# Human and zebrafish cross-species comparison on ligand affinity towards the aryl hydrocarbon receptor and Keap1 oxidative stress sensor: a molecular docking dataset

**DOI:** 10.1016/j.dib.2026.113037

**Published:** 2026-07-07

**Authors:** Sebastian Lungu-Mitea, Jana Horáčková, David Bednář, Klára Hilscherová

**Affiliations:** a RECETOX, Faculty of Science, Masaryk University (MUNI), CZ-62500 Brno, Czech Republic; b Department of Pharmaceutical Biosciences, Drug Safety and Toxicology, Uppsala University (UU), SE-75124, Uppsala, Sweden; c Department of Biology and Environmental Science, Linnaeus University (LNU), SE-39182, Kalmar, Sweden; d Loschmidt Laboratories, Department of Experimental Biology and RECETOX, Masaryk University, CZ-62500, Brno, Czech Republic; e International Clinical Research Centre, St. Anne’s University Hospital Brno, CZ-60200, Brno, Czech Republic; f Department of Animal Biosciences, Molecular Toxicology, Swedish University of Agricultural Sciences (SLU), SE-75007, Uppsala, Sweden

**Keywords:** Molecular docking, Receptor-ligand affinity, Aryl hydrocarbon receptor, Nrf2 Keap1 pathway, Cysteine code, Cross-species sensitivity assessment

## Abstract

Bioanalytical methods targeting adverse cellular outcomes are increasingly used in environmental toxicology, including receptor-mediated toxicity pathways relevant to high-throughput monitoring of drinking water, wastewater, and other environmental samples. However, despite their increasing regulatory acceptance, the representativeness of human tissue-derived bioassays for assessing toxicity toward aquatic species remains uncertain. Given these circumstances, this data article presents molecular docking and sequence-alignment datasets generated to compare human and zebrafish receptor/sensor variants associated with the oxidative stress response (Keap1/Nrf2) and xenobiotic metabolism (AhR/ARNT) pathways. The dataset includes docking data for hKeap1, zfKeap1a, and zfKeap1b, as well as hAhR, zfAhR1a, zfAhR1b, and zfAhR2 receptor/sensor variants, with selected reference ligands and environmental pollutants.

Receptor/sensor protein structures derived from AlphaFold predictions, X-ray crystallography, or cryo-electron microscopy were retrieved, prepared, and refined for molecular docking. Ligand structures included the reference agonists tetrachlorodibenzodioxin and tert‑butylhydroquinone, as well as the environmental pollutants climbazole, daidzein, thiabendazole, and metazachlor. Docking was performed using AutoDock Vina, generating the best-energy poses for each ligand-protein pair within defined docking grids. AlphaFold-predicted structures were evaluated by parallel docking into available partial X-ray crystal structures of the corresponding variants. For Keap1 variants, blind and site-directed docking approaches were applied, including grids covering reactive cysteine-associated binding regions, and potential effects of Keap1 dimerisation were considered. For AhR variants, docking focused on the PAS-B ligand-binding domain. Protein sequence alignment was conducted using the ClustalW algorithm to compare human and zebrafish receptor/sensor variants and support cross-species comparison of docking outputs.

The dataset comprises prepared protein and ligand files, representative docking poses, binding-affinity outputs, and sequence-alignment data. These files provide reusable input and output material for comparative toxicology, cross-species extrapolation, receptor-ligand interaction assessment, and benchmarking of molecular docking workflows. The data may inform scientists and regulators interested in molecular initiating events, toxicity-pathway conservation, and interspecies differences in receptor-mediated responses. This data article complements a related research article by providing supporting docking data, receptor/sensor variant sequence-alignments, and quality-assurance procedures for the molecular docking workflow.

Specifications Table

**Table:** 

Subject	Biology
Specific subject area	Computational Biology: Molecular docking of ligands and receptors/sensors of environmental toxicological concern
Type of data	Table Image Graph Figure Molecular docking illustration Raw - within the article and supplementary files Analysed - within the article and supplementary files Illustrative - within the article and supplementary files
Data collection	The dataset was entirely generated *in silico*. All generated and utilised PDBQT and FASTA-type files are given in the supplementary information (SI10). All acquired crystal and cryo-EM partial structures are mentioned in section 4 (experimental design, materials and methods). The following data sources were utilised: PubChem (https://pubchem.ncbi.nlm.nih.gov) UniProt (https://www.uniprot.org) RCSB Protein Data Bank (https://www.rcsb.org) National Center for Biotechnology Information (NCBI) (https://www.ncbi.nlm.nih.gov/)
Data source location	Institution: Swedish University of Agricultural Sciences (SLU) City, Region: Uppsala, Uppland Country: Sweden
Data accessibility	Repository name: Swedish National Data Service (SND): The SND Data Organisation and Information System (Doris) Data identification number: 2025–199; doi.org/10.5878/zwnq-qb88 Direct URL to data: https://doi.org/10.5878/zwnq-qb88
Related research article	DOI:10.1016/j.ecoenv.2026.120709

## 1 Value of the Data


 

• The dataset can be used to identify inter-species sensitivity differences to chemical pollutants that appear within the aquatic environment.
• Further, the dataset helps estimate the likelihood of the investigated chemicals to induce receptor pathways relevant to environmental toxicology.
• Scientists and regulators can potentially assess how inter-species sensitivities impact hazard and risk assessment or decision-making.
• Insight into the application of *in silico* molecular docking models.
• Assessment of the functionality of predicted protein 3D structures from given AA sequences compared to crystal and cryo-EM structures.

## 2 Background

High-throughput in vitro bioassays, also termed new approach methods, effect-based methods, or bioanalytical tools, have gained prominence in environmental toxicology over the last few years [1]. Endpoints which monitor specific adverse outcomes on the cellular level are commonly employed. However, most of the utilised bioassays are of mammalian origin. To what extent such represent protection goals within the environment, e.g., aquatic species, is still debated [2]. Elaborating on the latter, we conducted *in silico* molecular docking on human and zebrafish molecular receptors associated with adverse outcome pathways.

In this data article, we showcase the potential of chemicals which were identified within environmental water samples (CLB, DAI, MZC, TBDZ; fig. S2 in the supplementary manuscript (SM); abbreviations given in Table 1) to bind as ligands to the Keap1 sensor or AhR receptor *in silico* and potentially initiate respective toxicity pathways, the oxidative stress response and xenobiotic metabolism pathways. Additionally, we analysed two known pathway agonists (tBHQ, TCDD). Furthermore, we compared receptor susceptibility to these ligands between human and zebrafish variants. To this end, we either retrieved available crystal or cryo-EM structures or, if unavailable, we predicted 3D tertiary structures from AA sequences using AlphaFold. Molecular docking between ligands and receptors was conducted in AutoDock Vina.

**Table 1:** Summary of used abbreviations.

Abbreviation	Explanation
AA	Amino acid
AhR	Aryl hydrocarbon receptor
BTB	Broad complex, tramtrack, and bric a brac domain
CLB	Climbazole
cryo-EM	Cryogenic electron microscopy
CysX	Cysteine residue no X
DAI	Daidzein
Fig. Sx	Supplementary figure X
IVR	Intervening region domain
Keap1	Kelch-like ECH-associated protein 1; human (h) or zebrafish (zf) variant
Kelch domain	Five to seven kelch tandem repeats that form a β-propeller tertiary structure
MZC	Metazachlor
Nrf2	Nuclear factor erythroid-derived 2-like
PAS	Per-Arnt-Sim domain
PDB ID 1ZGK, 7EXI, 7ZUB	Protein database identifier of crystal or cryo-EM structures
SIx	Supplementary information, file no x
TBDZ	Thiabendazole
tBHQ	tert-Butylhydroquinone
TCDD	2,3,7,8-Tetrachlorodibenzodioxin

## 3 Data Description

To facilitate the understanding and readability of the manuscript, Table 1 provides a summary of the abbreviations used.

The raw and analysed data are either provided within this manuscript, in the supplementary information (SI), which is uploaded to the data-sharing repository (https://doi.org/10.5878/zwnq-qb88), or in the supplementary manuscript (SM). As computed by the AutoDock Vina algorithm, the raw data are provided in tables within this manuscript (Table 3, Table 4, Table 5, Table 6, Table 7, Table 8) and in SI9. The SI files are numbered SI9 to SI11; Table 2 summarises the SI. The enumeration starts with SI9 because other material originates from an overarching project within the data repository that relates to the original research article this manuscript accompanies (see also tab. S1 and Section 1 of the SM for the project’s concept and framework). All illustrations reference both the raw and analysed datasets to guide the reader and facilitate navigation. For brevity, detailed ligand poses and methodological descriptions were moved to the SM, which also serves as a link to the main article, in accordance with *Data in Brief* guidelines.

**Table 2:** Description of supplementary information (SI) within the data repository.

Name	Content
SI9	Summarised binding energies
SI10	PDBQT and FASTA-type files of ligands and receptors
SI11	TCDD-energy-equivalent calculations
SM	Supplementary manuscript

### 3.1 Molecular docking of tBHQ and MZC to keap1 - sensing of the oxidative stress response

Ligands (fig. S2), Keap1 receptor/sensor sequences (Fig. 6, Fig. 7), and 3D structures (fig. S3) were derived as described in the experimental design, materials, and methods 4.1, 4.2 and forwarded to *in silico* molecular docking using the AutoDock Vina algorithm. The known agonist tBHQ and MZC (fig. S2, panels A and B), a pollutant shown to induce the Nrf2-Keap1 pathway [[3], [4], [5]], were docked to different variants of human and zebrafish Keap1 to (A) gain insight into the potential binding location, (B) vicinity to known, active, canonical cysteine residues (Cys 151, 226, 273, 288, 613, 622, and 624; [6]), (C) to compare the predicted binding poses, and (D) to compare binding poses between crystallographic and AlphaFold-predicted structures.

In the following, all docking exercises are illustrated and described, starting with the blind docking and followed by the Cys151-site-directed docking for each compound. More detailed, zoomed-in docking poses are provided in the SM, section 3.1 (figs. S5 to S27). Discovered docking sites are described hierarchically, starting with domains that showed higher interaction affinities between the ligand and the receptor/sensor. The docking sites are colour-coded to facilitate orientation. The following images illustrate the computed binding poses for the identified Keap1 receptor/sensor site, followed by detailed poses for each domain. Ten poses were fitted per defined docking grid (”boxes”). Roughly, box1 was fitted to the beta-propeller region (Kelch domain, see fig. S3), whereas box2 was fitted to the alpha-helical-dominated region of Keap1 (BTB and IVR domains, see fig. S3). Box3 was fitted to the surroundings of Cys151 to conduct the site-directed docking. At the end of each section, tables summarise all binding sites and poses, along with their binding energies.

Additionally, AA sequences of all Keap1 variants have been aligned in Unipro Ugene utilising the ClustalW algorithm. Alignment was performed to compare primary protein structures and sequence homology with the molecular docking results obtained from tertiary protein structures. hKeap1 was chosen as the alignment template. All the following AA residue enumerations correspond to the latter. Beyond, alignment was conducted to the overall AA sequence (Fig. 6) and to the proximity of canonical cysteine 151 (Fig. 7). Multiple sequence alignment distance matrices are given for each comparison between variants.

#### 3.1.1 Molecular docking of tBHQ

##### 3.1.1.1 Blind docking of tBHQ: highest affinity - alpha-helical region (IVR domain)

**Fig. 1:**
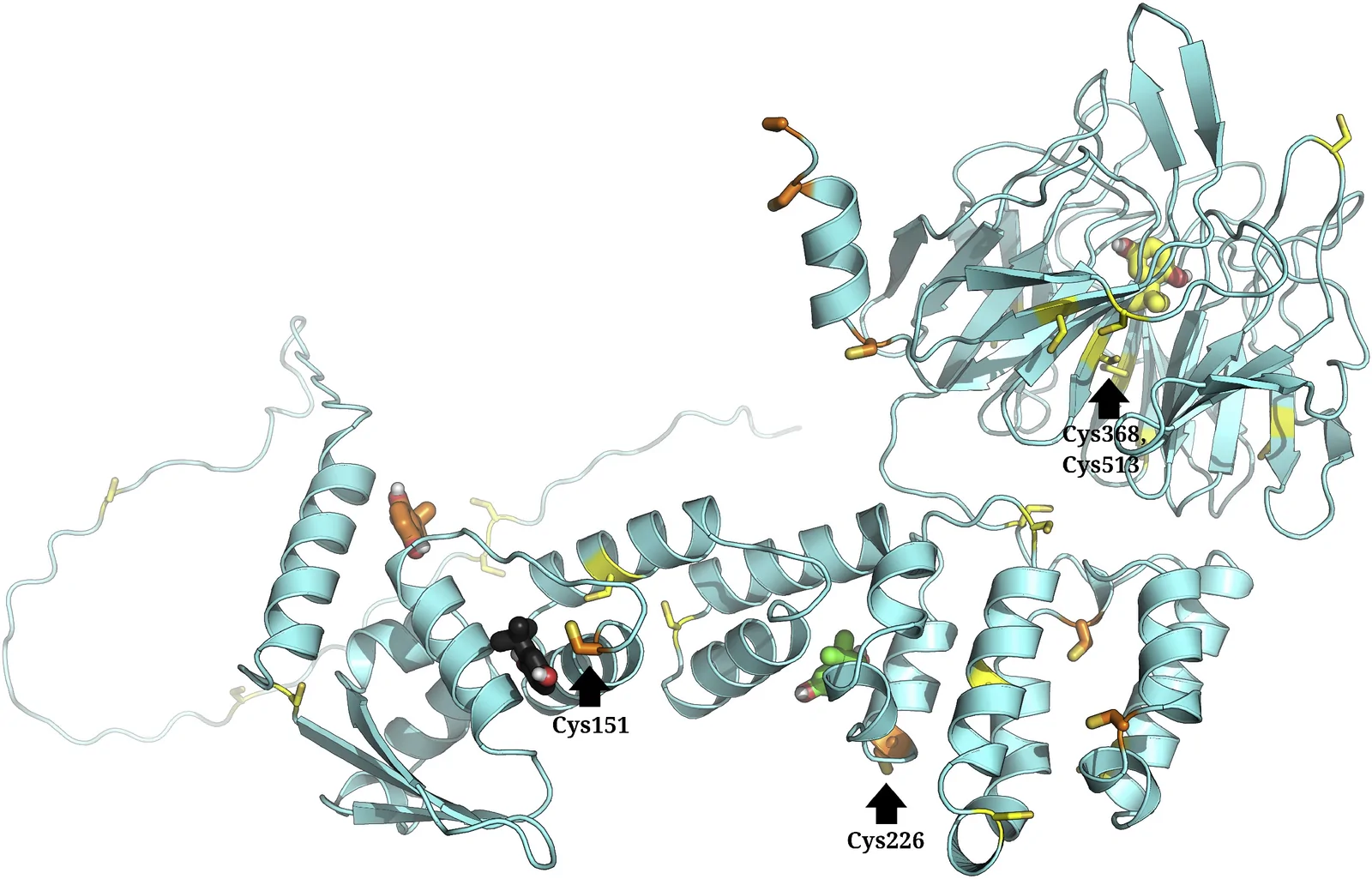
Best energy poses of tBHQ (coloured ligands) per site from blind and site-directed docking to the full-sequence AlphaFold-predicted structure of hKeap1 (cyan cartoon). The identified best ligand poses per binding site are colour-coded, with green (site A) corresponding to the best predicted binding energy, yellow (site B) to the medium, and orange (site C) to the worst. The black ligand (site D) corresponds to the site-directed docking. Canonical cysteine residues are highlighted as orange sticks branching from the protein’s backbone, while the remaining cysteines are in yellow. All illustrative data is summarised quantitatively in Table 3, Table 4.

**Fig. 2:**
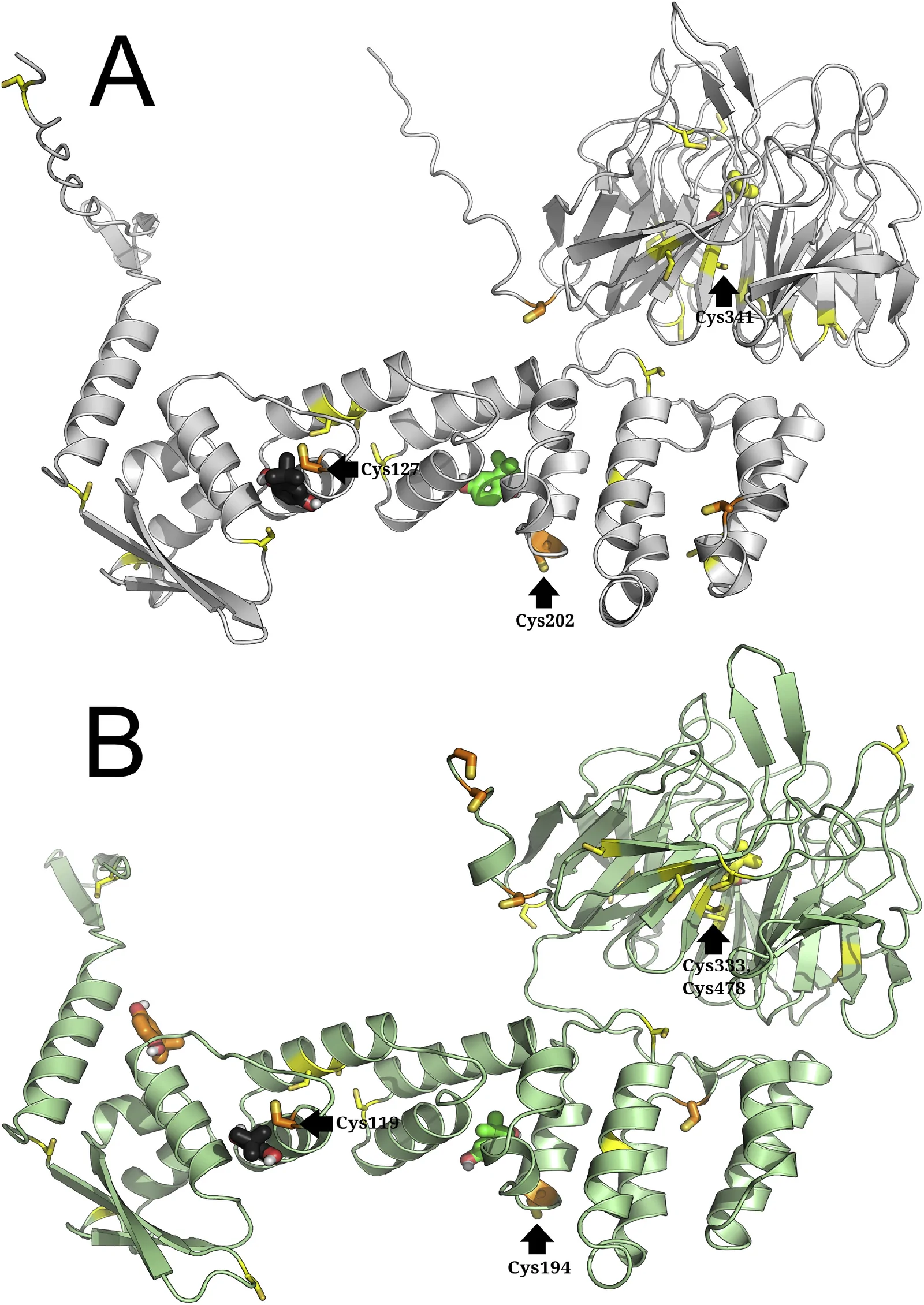
Best energy poses of tBHQ (coloured ligands) per site from blind and site-directed docking to the full-sequence AlphaFold-predicted structures of zfKeap1a (light grey cartoon, A) and zfKeap1b (light green cartoon, B). The identified best ligand poses per binding site are colour-coded, with green (site A) corresponding to the best predicted binding energy, yellow (site B) to the medium, and orange (site C) to the worst. The black ligand (site D) corresponds to the site-directed docking. Canonical cysteine residues are highlighted as orange sticks branching from the protein’s backbone, while the remaining cysteines are in yellow. All illustrative data is summarised quantitatively in Table 3, Table 4. Enumerated zebrafish cysteines correspond to Cys151, 226, 368, and 513 in hKeap1 (see also Fig. 6 for sequence alignment).

Docking of tBHQ to the AlphaFold-predicted full-sequence structure of hKeap1 (Fig. 1, Table 3, Table 4) revealed three binding sites with binding energies of −6.6 (green ligand, site A), −6.2 (yellow ligand, site B), and −5.5 kcal/mol (orange ligand, site C). The best-energy binding site (site A) is situated in the IVR domain of hKeap1, with Cys226 7.1 Å apart from this binding pose (the details of this pose are shown in fig. S5, SM). Similar binding was also observed in zebrafish variants zfKeap1a and zfKeap1b (green ligands in Fig. 2, details of best-energy poses in fig. S6, Table 3, Table 4) with binding energies of −6.3 and −6 kcal/mol, respectively.

**Table 3:** Summary of the blind (box1 and box2) and site-directed docking (box3) of tBHQ to Keap1 variants. The individual poses at the same binding site are highlighted in the same colour (as illustrated in the cartoons above). For the blind docking poses, green corresponds to the best predicted binding energy (site A), yellow to the medium (site B), and orange to the worst (site C). The poses from site-directed docking (box3) are shown against a black background (site D).

**Table 4:** Overview of best predicted binding energies regarding location (domain) and distance from potentially reactive cysteine residues. The colour coding corresponds to the discussion above and table 3.

##### 3.1.1.2 Blind docking of tBHQ: medium affinity - beta-propeller region (Kelch domain)

The second-best binding site (site B, −6.2 kcal/mol) in the hKeap1 predicted structure is situated inside the central cavity of the Kelch domain (yellow ligand in Fig. 1, details in fig. S7 (SM), Table 3, Table 4). There are two cysteine residues (368 and 513) at a distance of 5.7 to 8.3 Å from this binding site. Considering a rigid protein structure approximation during docking, this distance could be even lower in a real flexible protein and, therefore, more reactive. Similar binding poses were also observed in zfKeap1a and b (yellow ligands in Fig. 2, details in fig. S8, Table 3, Table 4) with binding energies of −6 and −6.1 kcal/mol, respectively.

**Fig. 3:**
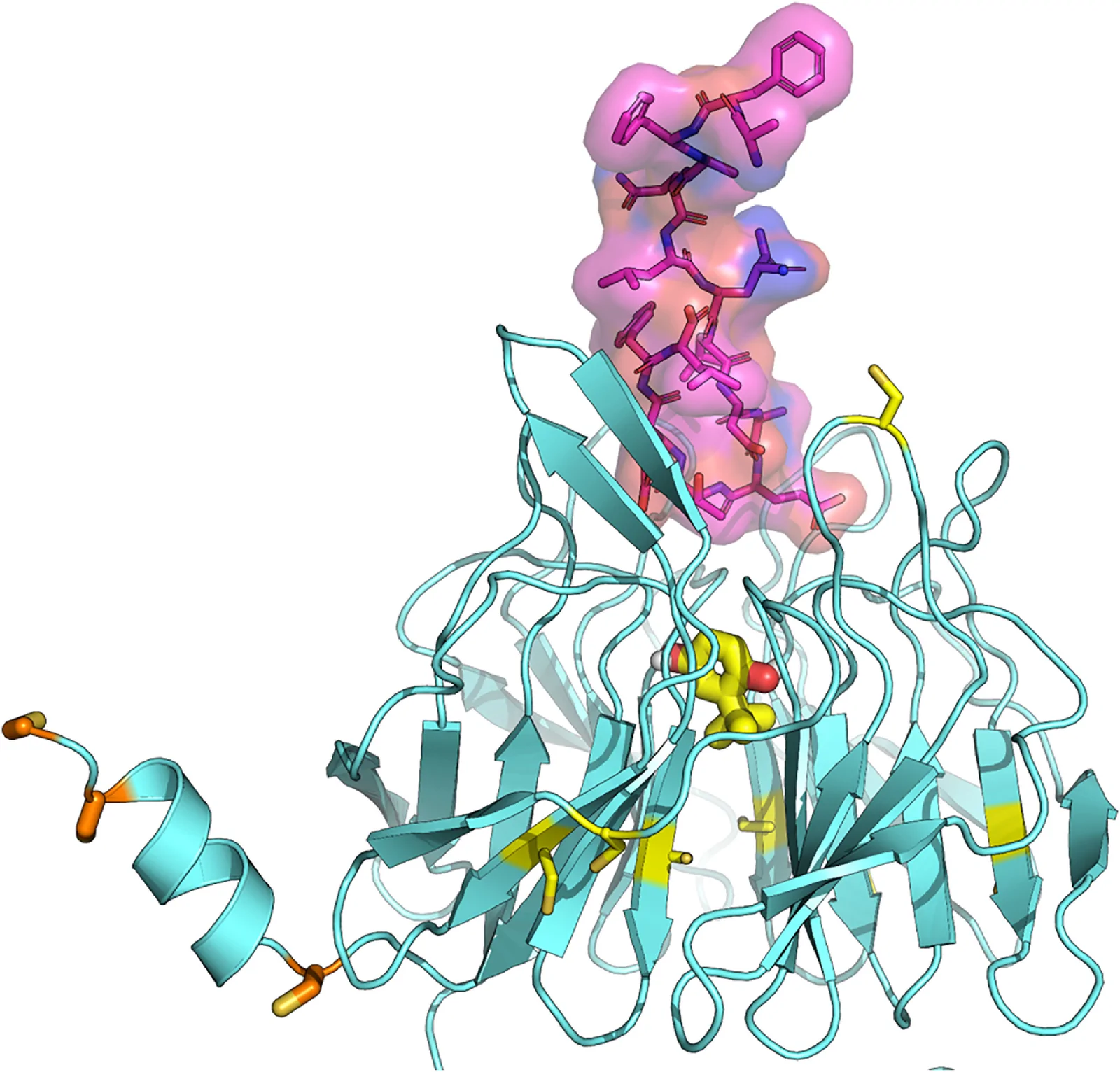
Docking of the partial crystal structure PDB ID 2FLU (ETGF motif of hNrf2) to the Kelch domain of hKeap1. There is no steric hindrance to ligand binding poses in the Cys368 and Cys513 proximity. Site B (yellow ligand) is given for orientation.

As Keap1 interacts with the associated transcription factor Nrf2 via the Kelch domain (background information given in the SM, Section 4.1), we simulated the respective docking of hNrf2’s ETGF motif by rendering the partial crystal structure PDB ID 2FLU (Fig. 3). No steric hindrance from the Keap1-Nrf2 interaction towards the ligand pose described here, and similar following poses, can be expected in this regard.

##### 3.1.1.3 Blind docking of tBHQ: worst affinity - BTB domain

The worst-energy predicted binding site (site C, −5.5 kcal/mol) of hKeap1 is located in the N-terminal region of its BTB domain (orange ligand in Fig. 1, details in fig. S9A (SM), Table 3, Table 4). However, there are no cysteine residues nearby. Again, similar binding poses were found in zfKeap1b (orange ligands in Fig. 2, fig. S9B, Table 3, Table 4) with a binding energy of −5.3 kcal/mol.

**Fig. 4:**
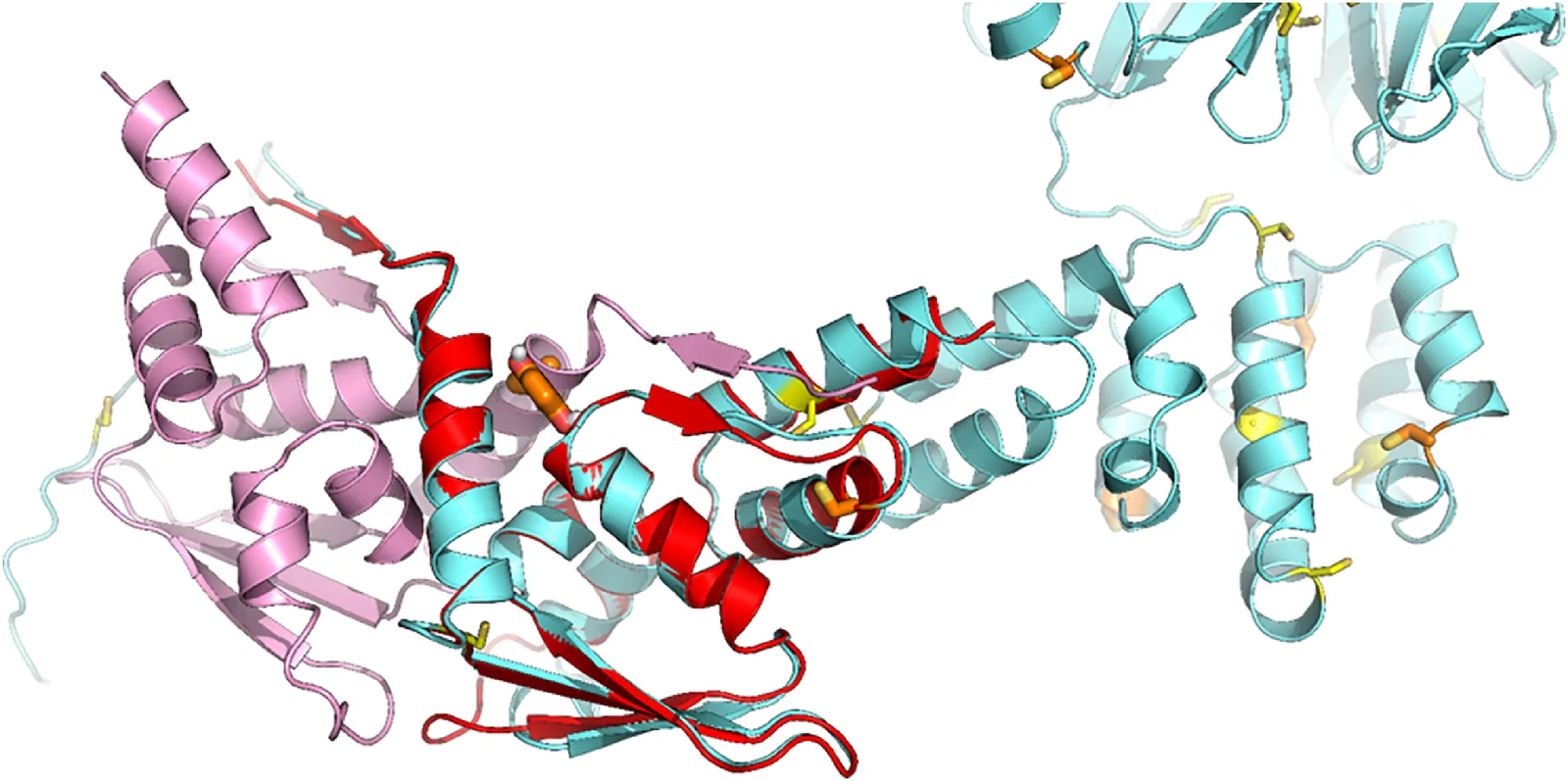
hKeap1 dimerisation via the BTB domain. Site C (orange ligand) is given for orientation. Dimer formation complex was retrieved from PDBe PISA v1.52 (https://www.ebi.ac.uk/pdbe) [7] .

Notably, site C is likely concealed, as Keap1 persists as a dimer in its active form, with the BTB domain being the location of protein-protein interaction (Fig. 4). The same is valid for the identical pose found for MZC (section 3.1.2.2). As this site might be disclosed during electrophilic exposure, we still list it here. However, as further discussion is beyond the scope of this manuscript, we refer the interested reader to section 4.1 of the SM, which provides a synopsis of Keap1 functionality, electrophilic exposure, and cysteine code interactions.

##### 3.1.1.4 Blind docking of tBHQ: verifying AlphaFold predictions via X-ray crystal structures

Docking was conducted on two partial crystal structures of hKeap1 to check whether the poses in the AlphaFold-predicted structures are consistent: PDB ID 1ZGK, partly covering the Kelch domain of hKeap1 (residues 322 to 609), and PDB ID 7EXI covering the BTB domain (residues 50 to 179, see also fig. S3). The best pose for tBHQ in the AlphaFold-predicted structure (green ligands, site A) could not be recalled since none of the X-ray structures resolves the region. However, the other two binding sites (yellow, site B, and orange ligands, site C) were found in the X-ray structures with similar affinities as in the hKeap1 model (fig. S10 (SM) and Table 3). Moreover, another possibly interesting tBHQ pose was found in the PDB ID 7EXI structure (site E, red ligand in fig. S10A, details in Table 3, Table 4). This pose has a binding energy of −5.1 kcal/mol and lies 4 Å from the Cys171 residue.

##### 3.1.1.5 Site-directed docking of tBHQ

It was reported that a Cys151Ser mutation significantly reduces the activity of mouse Keap1 in binding tBHQ [8]. However, no pose of tBHQ near Cys151 was found by the blind docking approach, suggesting worse binding energies than the obtained ones. Therefore, site-directed docking was attempted to generate poses near Cys151 (black ligands, site D, Fig. 1, Fig. 2, S11, and S12 (SM)) and compare their binding energies with the blind docking results. For hKeap1, the binding energy is only 0.3 kcal/mol worse than the poses obtained from blind docking. Interestingly, for zfKeap1 paralogs, this energy gap between Cys151-binding and other binding sites is more significant (2.0 and 1.1 kcal/mol for zfKeap1a and zfKeap1b, respectively; see also Table 3, Table 4).

##### 3.1.1.6 tBHQ molecular docking results - summary

**Fig. 5:**
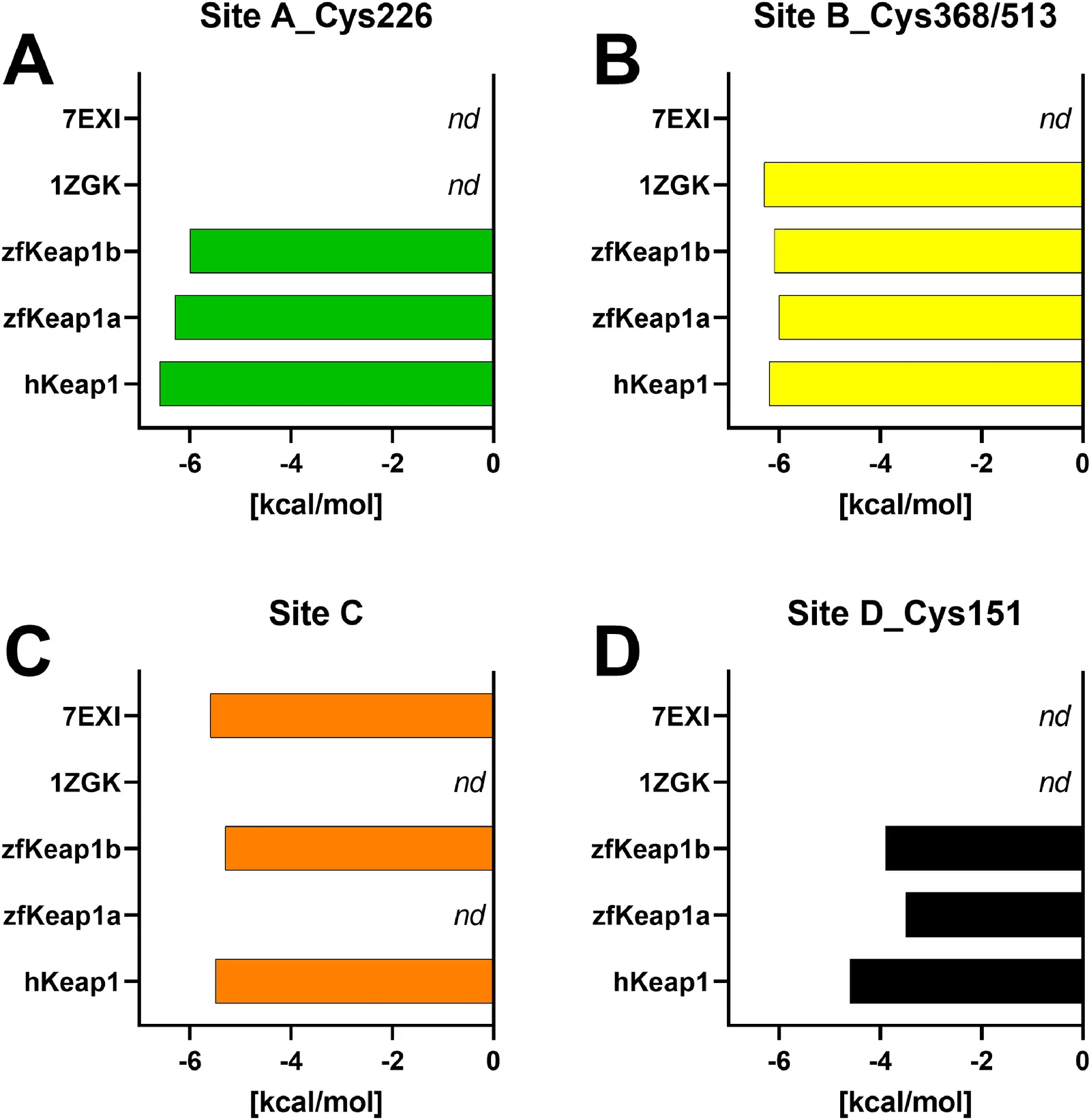
Illustration of the highest predicted binding energy poses of tBHQ regarding location (site), as derived from the blind and site-directed docking, for all investigated Keap1 variants. Colour coding corresponds to Table 3, Table 4.

All tBHQ docking results are summarised in Table 3, Table 4. Regarding blind docking, there are only minor qualitative differences in binding affinity between human and zebrafish (co-)orthologs if the zebrafish patterns are overlaid (Fig. 5A–C). However, by themselves, zfKeap1a and zfKeap1b differ in their binding affinity patterns (Table 3). zfKeap1a shows higher binding potential within the alpha-helical region (BTB and IVR domains), whereas zfKeap1b has more prominence within the beta-propeller region (Kelch domain). The binding site within the N-terminal BTB domain is found in zfKeap1b and hKeap1 within the first ten optimal poses (Fig. 5C) but not in zfKeap1a. As mentioned above, this site might not be directly accessible within the dimerised protein complex (Fig. 4). Differently, Nrf2-Keap1 interaction via the Kelch domain does not interfere with ligand binding site accessibility in the proximity of the residues Cys368 and 513 (Fig. 3).

**Fig. 6:**
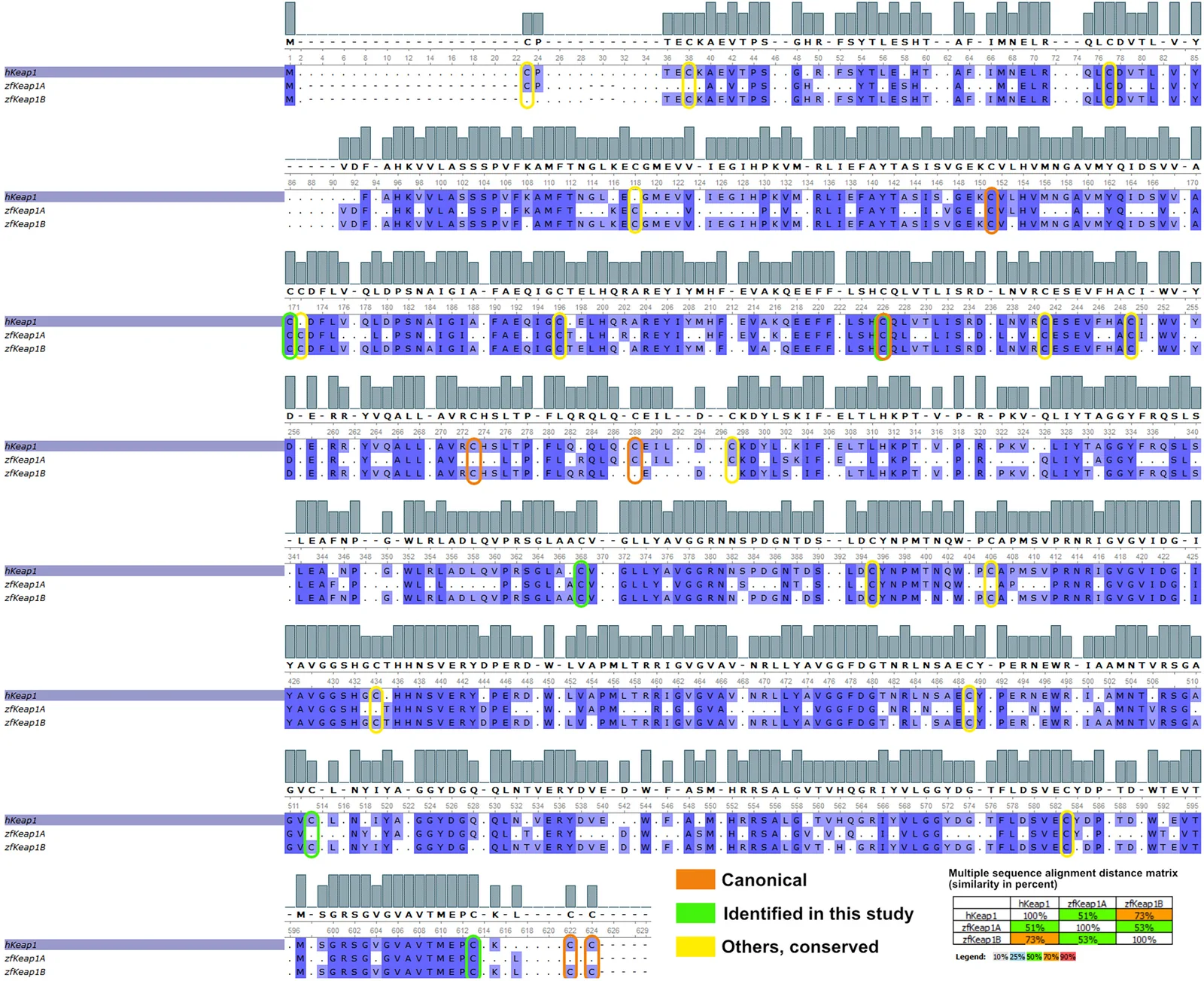
Keap1 amino acid (AA) sequence alignment. zfKeap1a and zfKeap1b were aligned to hKeap1 as a reference. AA similarity is indicated by grey bars and blue-shaded highlights. A multiple-sequence alignment distance matrix is shown in the lower-right corner. Cysteine residues are emphasised via colourful ovals, canonical reactive cysteines in orange, other conserved cysteines in yellow, and cysteines identified within this study in green. An AA sequence alignment was conducted in Ugene, using the ClustalW algorithm.

Overall, zfKeap1b seems closer to hKeap1 regarding qualitative susceptibility patterns towards tBHQ. As such, AA sequence homology is also more significant between hKeap1 and zfKeap1b (73% agreement) than zfKeap1a (51% agreement, Fig. 6). Nevertheless, tBHQ depicts higher absolute affinity within the IVR domain of zfKeap1a, in proximity to canonical Cys226, pointing towards a potential sensor diversification between the two zebrafish paralogs.

Moreover, AlphaFold-predicted structures are consistent with docking to partial X-ray structures of hKeap1, indicating that the predicted Keap1 models are relevant. Regarding the vicinity of cysteine residues, the best-energy pose of tBHQ (site A, green sticks) lies 7.1 Å from reactive residue Cys226. This distance could be even lower in a real flexible protein and, therefore, reactive. Also, a pose from the 7EXI structure shown as red ligands might be interesting, as it is within 4 Å of Cys171. No poses from blind docking were found near other prominent stress-sensor cysteines 151, 273, and 288 (see cysteine code in section 4.1 of the SM for explanation). Cys273 and 288 are missing within one zebrafish variant (Figs. 6 and S42). Therefore, their electrophile-sensing importance might hypothetically be reduced within zebrafish.

Site-directed docking of tBHQ to the Cys151 residue of Keap1 variants revealed somewhat lower absolute docking affinity than blind docking. However, for hKeap1, the difference was only 0.3 kcal/mol worse than the poses obtained from blind docking. Conversely, binding to Cys151 residues in zebrafish variants was significantly weaker, by 2.0 and 1.1 kcal/mol for zfKeap1a and 1b, respectively, than the poses predicted from blind docking. Overall, the differences in binding energies were qualitatively more pronounced between human and zebrafish Keap1 variants within the Cys151 vicinity (Fig. 5D).

**Fig. 7:**
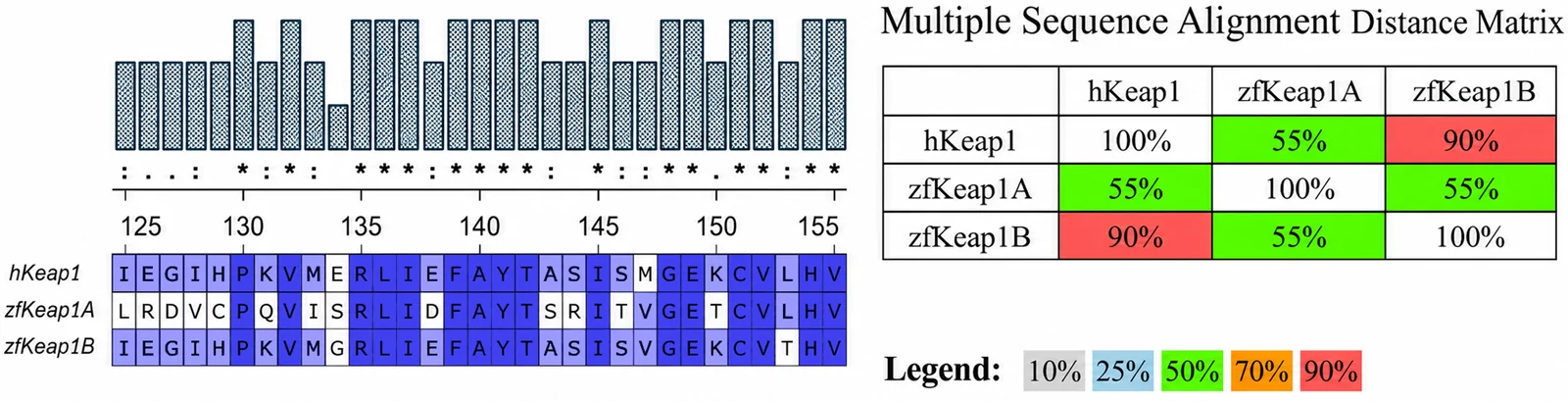
Sub-alignment of Keap1 AA sequence close to Cys151. AA residue enumeration corresponds to hKeap1. AA similarity is indicated by grey bars and blue-shaded highlights. A multiple-sequence alignment distance matrix is shown on the right. An AA sequence alignment was conducted in Ugene using the ClustalW algorithm.

Looking isolated at the Cys151 proximity (residues 125 to 155), hKeap1 shares 55% AA homology with zfKeap1a and 90% with zfKeap1b. Only 3 AA pairs differ between hKeap1 and zfKeap1b (Fig. 7). Nevertheless, this seemingly leads to a reduction in binding energy of 0.7 kcal/mol for the best energy pose. Nearby basic residues (H129, K131, K150, and H154) lower the pKa of Cys151, rendering it a thiolate anion even at physiological pH and thereby increasing its susceptibility to electrophilic attack.

In summary,

• When overlaying zebrafish Keap1 variants, tBHQ discloses qualitatively similar affinity patterns and binding energies to hKeap1 (blind-docking).
• Looking separately at the zebrafish variants, zfKeap1a shows absolutely higher susceptibility within the IVR domain, whereas, for zfKeap1b, susceptibility is most heightened within the Kelch domain.
• tBHQ binds with an absolute, higher affinity to the Cys151 proximity of hKeap1 compared to the zebrafish paralogs.
• Docking into crystal structures was consistent with AlphaFold-predicted structures.

#### 3.1.2 Molecular docking modelling of MZC

##### 3.1.2.1 Blind docking of MZC: highest affinity - beta-propeller region (Kelch domain)

**Fig. 8:**
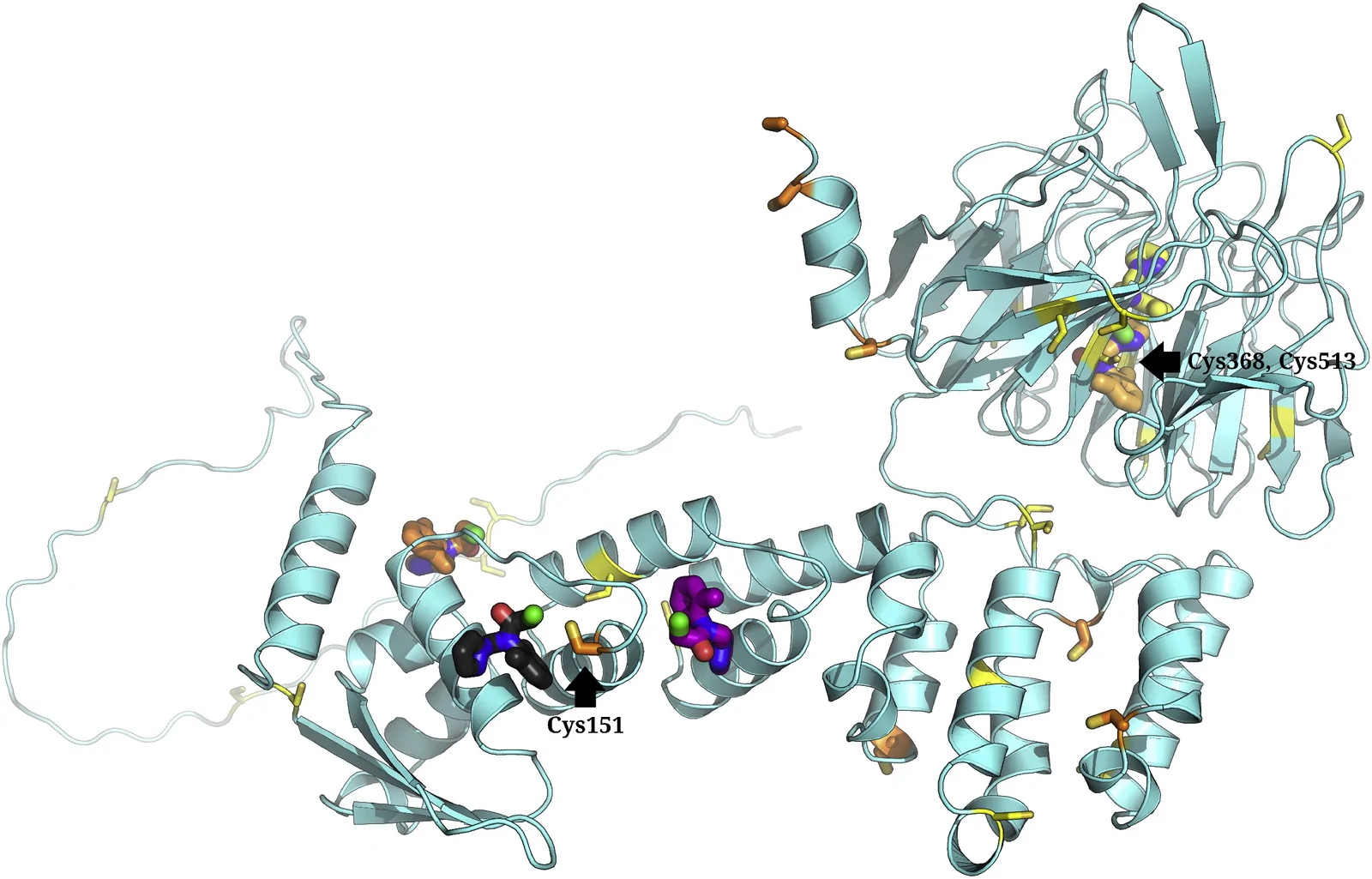
Best energy poses of MZC (coloured ligands) per site from blind docking to the full-sequence AlphaFold-predicted structure of hKeap1 (cyan cartoon). The identified best poses per binding site are colour-coded, with yellow (site A) corresponding to the best-predicted binding energy. Canonical cysteine residues are shown as orange sticks, while the remaining cysteines are yellow. All illustrative data is summarised quantitatively in Table 5, Table 6.

**Fig. 9:**
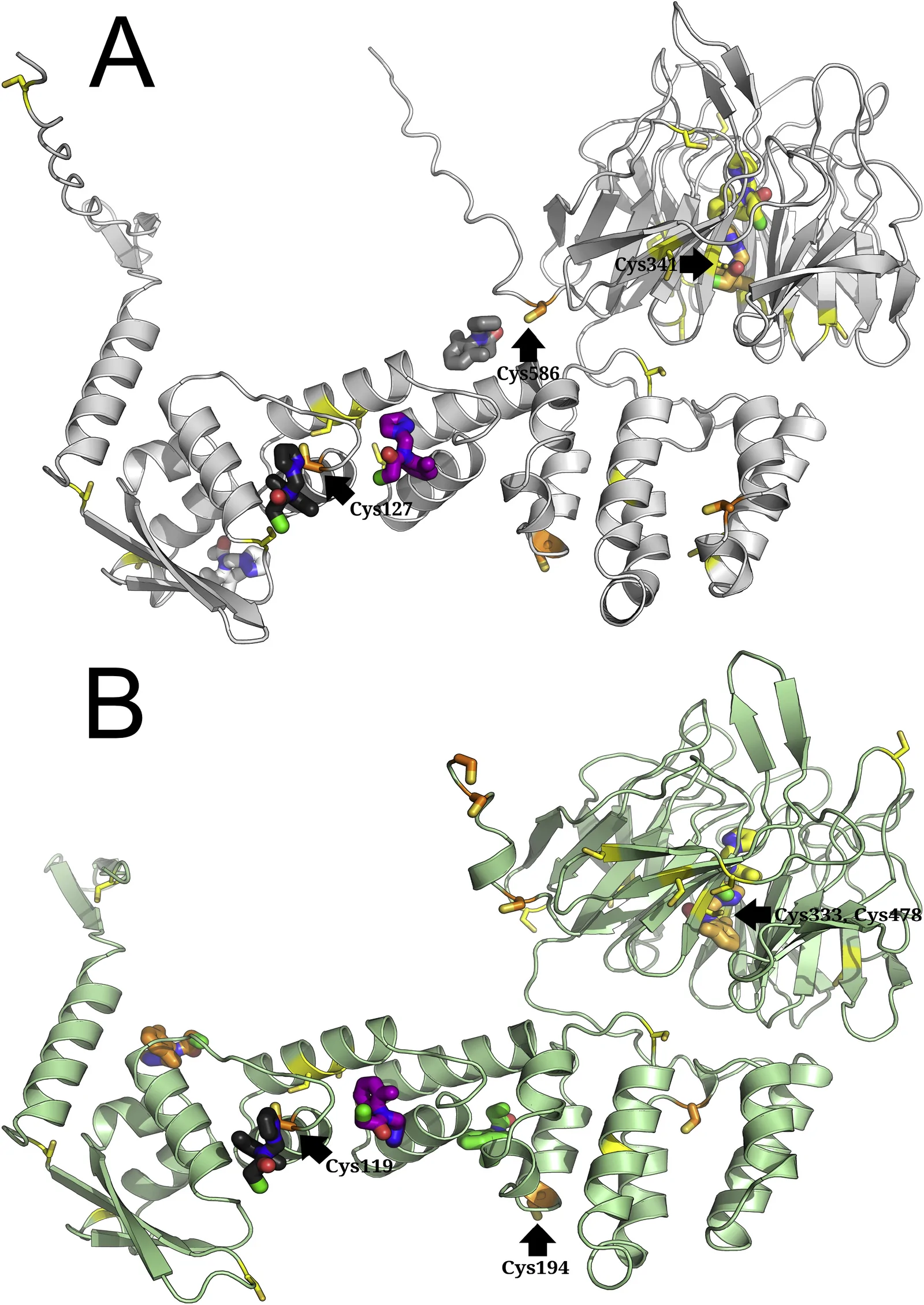
Best energy poses of MZC (coloured ligands) per site from blind docking to the full-sequence structure of zfKeap1a (light grey cartoon, A) and zfKeap1b (light green cartoon, B). The identified best poses per binding site are colour-coded, with yellow corresponding to the best-predicted binding energy (site A). Canonical cysteine residues are highlighted as orange sticks branching from the protein’s backbone, while the remaining cysteines are in yellow. All illustrative data is summarised quantitatively in Table 5, Table 6. Enumerated cysteine residues 119, 127, 194, 333, 341, 478, and 586 correspond to Cys151, 226, 368, 513, and 613 in hKeap1 (see also Fig. 6 for sequence alignment).

Docking of MZC to the AlphaFold-predicted structure of hKeap1 (Fig. 8) discovered five different binding sites with binding energies of −6.6 (site A, yellow ligand), −6.5 (site B, light orange ligand), −5 (site C, orange ligand), −5 (site D, violet ligand), and −4.8 kcal/mol (site E, black ligand). The best-energy binding site (site A, details in fig. S13 (SM), Table 5, Table 6) is situated in the middle of the central cavity of the Kelch domain of hKeap1. There are two cysteine residues (Cys368 and Cys513) at a distance of 5.5 and 7.1 Å from this binding site. Considering only rigid protein structure during the docking, this distance could be even lower in a real flexible protein and, therefore, reactive. As mentioned above, for tBHQ, this binding site is accessible despite the Nrf2-Keap1 interaction via the Kelch domain (section 3.1.1.2). Similar binding was also observed in zfKeap1a and zfKeap1b (yellow ligands in Fig. 9, details in fig. S14, Table 5, Table 6) with binding energies of −6.9 and −6.8 kcal/mol, respectively.

**Table 5:** Summary of the blind (box1 and box2) and site-directed docking (box3) of MZC to Keap1 variants. The individual blind-docked poses in the same binding site are highlighted by the same colour (as illustrated in the cartoons above), with yellow (site A) and light-orange (site B) corresponding to the best predicted binding energies, whereas orange (site C), violet (site D), green (site F), grey (site G), white (site H), and red (site I) representing intermediate to worst predicted energies. The poses from site-directed docking (box3) are shown against a black background (site E).

**Table 6:** Overview of best predicted binding energies regarding location (domain) and distance from potentially reactive cysteine residues for MZC. The colour coding corresponds to the discussion above and table 5.

The second-best binding site in hKeap1 (site B, −6.5 kcal/mol, light orange ligands) is also situated inside the Kelch domain but closer to the intramolecular interface with the IVR domain (Fig. 8, details in fig. S15, Table 5, Table 6). Two cysteine residues (Cys368 and Cys513) are located close to this binding site (3.7 and 3.9 Å). Again, similar binding poses were observed in zfKeap1a and zfKeap1b (light orange ligands in Fig. 9, details in fig. S16, Table 5, Table 6) with binding energies of −6.4 and −6.5 kcal/mol, respectively.

##### 3.1.2.2 Blind docking of MZC: medium to low affinity - alpha-helical region (BTB and IVR domains)

While the MZC docking poses in the Kelch domain are similar among the different Keap1 structures, in the alpha-helical region, they show more variation, as seen in Table 5, Table 6. MZC prefers to bind in the Kelch domain of Keap1 (∼1.5 kcal/mol difference), whereas tBHQ does not strongly prefer one part of the protein.

The best binding site of MZC in the alpha-helical region of the predicted structure of hKeap1, with a binding energy of −5 kcal/mol (site C, orange ligand in Fig. 8, details in fig. S17A (SM), Table 5, Table 6), is identical to the third-best binding site of tBHQ with no cysteines around, and, hence, located within the BTB domain. A similar pose with a similar binding energy was predicted for zfKeap1b (−5.1 kcal/mol; figs. 9B and S17B). As mentioned above, this site is not directly accessible within the dimeric protein complex (section 3.1.1.3).

The subsequent binding site within hKeap1 (site D, violet ligand in Fig. 8, details in fig. S18A, Table 5, Table 6) has a similar binding energy to site C, with no nearby cysteine residues. The site was recalled in the zfKeap1a and 1b variants (Fig. 9; details in fig. S18B+C, Table 5, Table 6), with somewhat higher binding energies of −5.6 and −5.1 kcal/mol, respectively. Binding site D is situated at the C-terminal end of the BTB domain, within a region called ”3-box” (not highlighted in fig. S3).

The last blindly predicted binding site for hKeap1, shown as black sticks in Fig. 8 (site E, details in fig. S19, Table 5, Table 6) with a binding energy of −4.8 kcal/mol, lies within 3.5 Å of the reactive residue Cys151. The other Keap1 structures did not recall this binding site via blind docking.

In zfKeap1b, the preferred binding site of MZC within the IVR domain is shown as a green ligand in Fig. 9B (site F, details in fig. S20, Table 5, Table 6), with a binding energy of −5.6 kcal/mol. This site is identical to the best-energy binding site of tBHQ. The MZC pose lies 6.5 Å from a reactive Cys194 (corresponding to Cys226 in hKeap1), which can be even closer in reality, given the flexibility of side chains.

Two other potential binding sites were found in zfKeap1a. First (site G, grey ligand in Fig. 9A, details in fig. S21A, Table 5, Table 6), with a binding energy of −5 kcal/mol. This binding site lies within 3.9 Å of canonical Cys613 and is very likely reactive. Second, with a binding energy of −4.9 kcal/mol and no adjacent cysteines (site H, white ligand in Figs. 9A and S21B).

##### 3.1.2.3 Blind docking of MZC: verifying alphafold predictions via X-ray crystal structures

Docking to partial crystal structures of hKeap1 (PDB ID 1ZGK and 7EXI) revealed similar poses (fig. S22 (SM)) as docking to the AlphaFold structure; the two binding sites in the Kelch domain (site A, yellow and site B, light-orange ligands) and the best-energy binding site in the alpha-helical region (site C, orange ligand). Moreover, a similar binding site as for tBHQ (site I, red ligand) was predicted in the 7EXI structure with a binding energy of −4.9 kcal/mol (fig. S22A). The MZC pose is within 4 Å of Cys171.

##### 3.1.2.4 Site-directed docking of MZC

Blind docking of MZC to hKeap1 already revealed a potential pose close to Cys151 (black ligand in Fig. 8, site E, −4.8 kcal/mol). However, no such sites could be recalled for the zebrafish variants within the first ten poses. Therefore, site-directed docking to Cys151 (box3) was performed for all predicted variants. The latter revealed best binding energies of −3.6 and −4.1 kcal/mol for zfKeap1a and 1b, respectively, which can be considered qualitatively lower than hKeap1 (black ligand in Fig. 9, details in fig. S23 (SM), Table 5, Table 6).

##### 3.1.2.5 MZC molecular docking results - summary

**Fig. 10:**
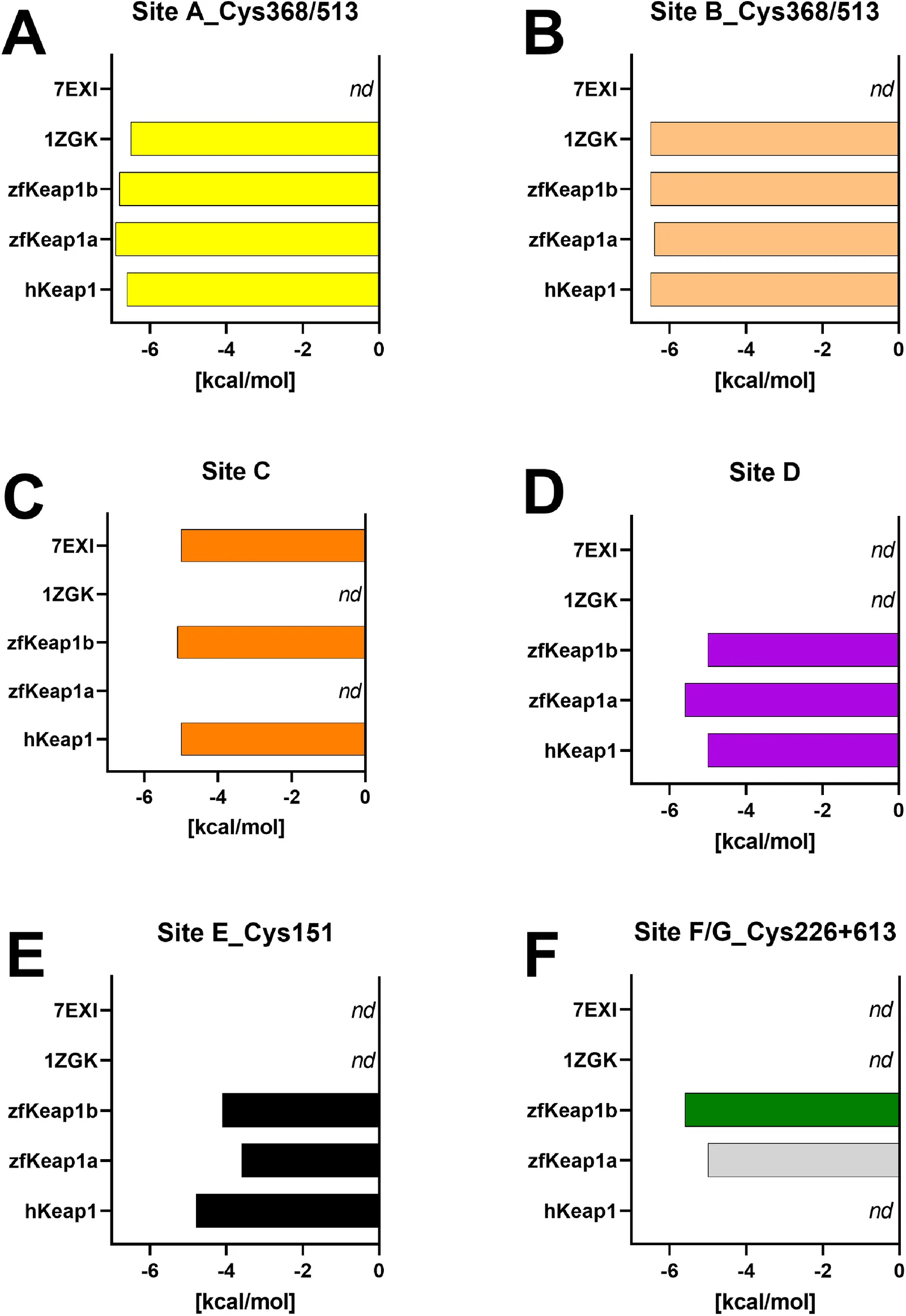
Illustration of the highest predicted binding energy poses of MCZ regarding location (site) as derived from the blind and site-directed docking for all investigated Keap1 variants. Colour-coding corresponds to Table 5, Table 6.

All MZC docking results are summarised in Table 5, Table 6. In general, MZC binds to the Kelch domain with higher affinity, proximal to the reactive cysteine residues 369 and 513. Notably, Cys513 is not conserved in the zfKeap1a variant. Within the Kelch domain’s central cavity, no significant predicted binding differences exist between human and zebrafish Keap1 variants (Fig. 10A+B). At the same time, within the alpha-helical region, MZC prefers different binding sites in each variant (Fig. 10C+D, Table 5, Table 6). As such, hKeap1 showed the highest binding affinity in site C (BTB domain), zfKeap1a in site D (3-box domain), and zfKeap1B in site F (IVR domain).

Regarding the vicinity of cysteine residues, the second-best-energy binding site of MZC (site B, light-orange sticks) was found in all Keap1 structures and lies within ∼4 Å of Cys368 and Cys513. Similar to tBHQ, the docking results are consistent between predicted and X-ray structures of hKeap1. Interestingly, the Cys171 binding site (site I) was recalled again for the 7EXI sequence. Two additional binding sites near canonical cysteine residues were identified for the two zebrafish paralogs. Site F, for zfKeap1b, within a vicinity of 6.5 Å from Cys226 (−5.6 kcal/mol, green ligand in Fig. 9B), and site G, for zfKeap1a, located within 3.9 Å from Cys613 (−5 kcal/mol, grey ligand in Fig. 9A). As such, MZC secondary binding, besides the interaction location within the primary Kelch cavity (sites A and B), is differently modulated for every variant. Regarding non-Kelch-associated cysteines, MZC binds with the highest affinity to site E (Cys151, −4.8 kcal/mol) in hKeap1, site G (Cys613, −5 kcal/mol) in zfKeap1a, and site F (Cys226, −5.6 kcal/mol) in zfKeap1b.

Contrary to tBHQ, for MZC, a potential binding site near the canonical Cys151 residue could already be recalled from blind docking (site E, −4.8 kcal/mol). The site was also found in zfKeap1a and 1b (black ligands in Fig. 9), but with significantly lower binding energies of −3.6 and −4.1 kcal/mol, respectively. Further, the binding energy differences between hKeap1, zfKeap1a, and 1b are identical within the Cys151 proximity across both investigated ligands (1.1/1.2 and 0.7 Δ kcal/mol, Table 7). As mentioned for the tBHQ docking, a three AA substitution between hKeap1 and zfKeap1b sequences (Fig. 7) is sufficient for qualitative susceptibility differences towards potential ligands. It can be deduced that binding affinities near Cys151 are qualitatively weaker in zebrafish variants than in human variants, with the following hierarchy: hKeap1 > zfKeap1b > zfKeap1a.

**Table 7:** Binding energy differences within the proximity of canonical cysteine 151 in Δ kcal/mol between investigated Keap 1 variants, deduced from blind and site-directed docking.

When comparing the aligned variants’ AA sequences (Figs. 6 and S42), 24 canonical and conserved cysteines can be identified, out of which 22 appear within hKeap1. Cys118 and Cys172 are zebrafish-exclusive and might warrant further scrutiny. However, none of the latter was identified in the blind docking exercise and may play a secondary sensing role. zfKeap1a bears only 16 conserved cysteines (missing Cys38, 273, 406, 434, 489, 513, 622, and 624), whereas zfKeap1b has 21 conserved cysteines (missing Cys23, 288, and 297). In both cases, zebrafish variants either lack canonical cysteines or cysteines identified within blind docking (for both ligands). This might indicate a shift in functionality and a propensity toward other ligand classes for the respective zebrafish Keap1 paralogs.

In summary,

• MZC preferably binds to the Kelch domain with nearby cysteines 368 and 513. Absolute binding energies are comparable between human and zebrafish variants.
• Outside the Kelch domain, MZC prefers different sites for each variant (hKeap1 -> Cys151, zfKeap1a -> Cys613, zfKeap1b -> Cys226).
• Like tBHQ, the susceptibility of Cys151 to MZC is higher in hKeap1 than in zebrafish variants (hKeap1 > zfKeap1b > zfKeap1a). Binding energy differences in Δ kcal/mol for the Cys151 proximity are conserved across the investigated ligands.
• Docking into crystal structures was consistent with AlphaFold-predicted structures.

### 3.2 Docking of various ligands to the AhR - initiation of xenobiotic metabolism

Ligands (fig. S2), AhR sequences (Fig. 13), and 3D structures (fig. S4) were derived as described in the experimental design, materials, and methods 4.1, 4.2 and used for molecular docking predictions in AutoDock Vina. The known reference compound and inducer TCDD (fig. S2F) was docked to different variants of human and zebrafish AhR to gain insight into the potential binding location and to compare the predicted binding energies. Additionally, the ligands CLB, DAI, and TBDZ (fig. S2C-E), identified via effect-directed environmental sample analysis within the main accompanying article, were subjected to the identical site-directed docking calculation. Docking was performed against the PAS-B domain, as the ligand-binding site is located within it (fig. S4, [9]).

The following sections illustrate and describe all docking exercises for each compound, starting with those identified in the environmental samples. Additional illustrations for every best energy pose per compound and PAS-B variant are given in the SM, section 3.1. The ligands and poses are colour-coded to facilitate orientation. Up to ten poses, where possible, were identified per the defined docking grid (18×18×18 Å box around the PAS-B domain). Throughout this section, tables and figures summarise all binding sites and poses, along with their binding energies.

AA sequences of all AhR variants have been aligned in Unipro Ugene using the ClustalW algorithm. Alignment was performed to compare primary protein structures and sequence homology with the molecular docking results obtained from tertiary protein structures. hAhR was chosen as the alignment template. All following AA residue enumerations correspond to the latter. Alignment was conducted first for the total AhR AA sequence (Fig. 13A) and second for the PAS-B ligand binding domain and its near vicinity (Fig. 13B). Multiple sequence alignment distance matrices are given for each comparison between variants.

#### 3.2.1 PAS-B docking results

**Fig. 11:**
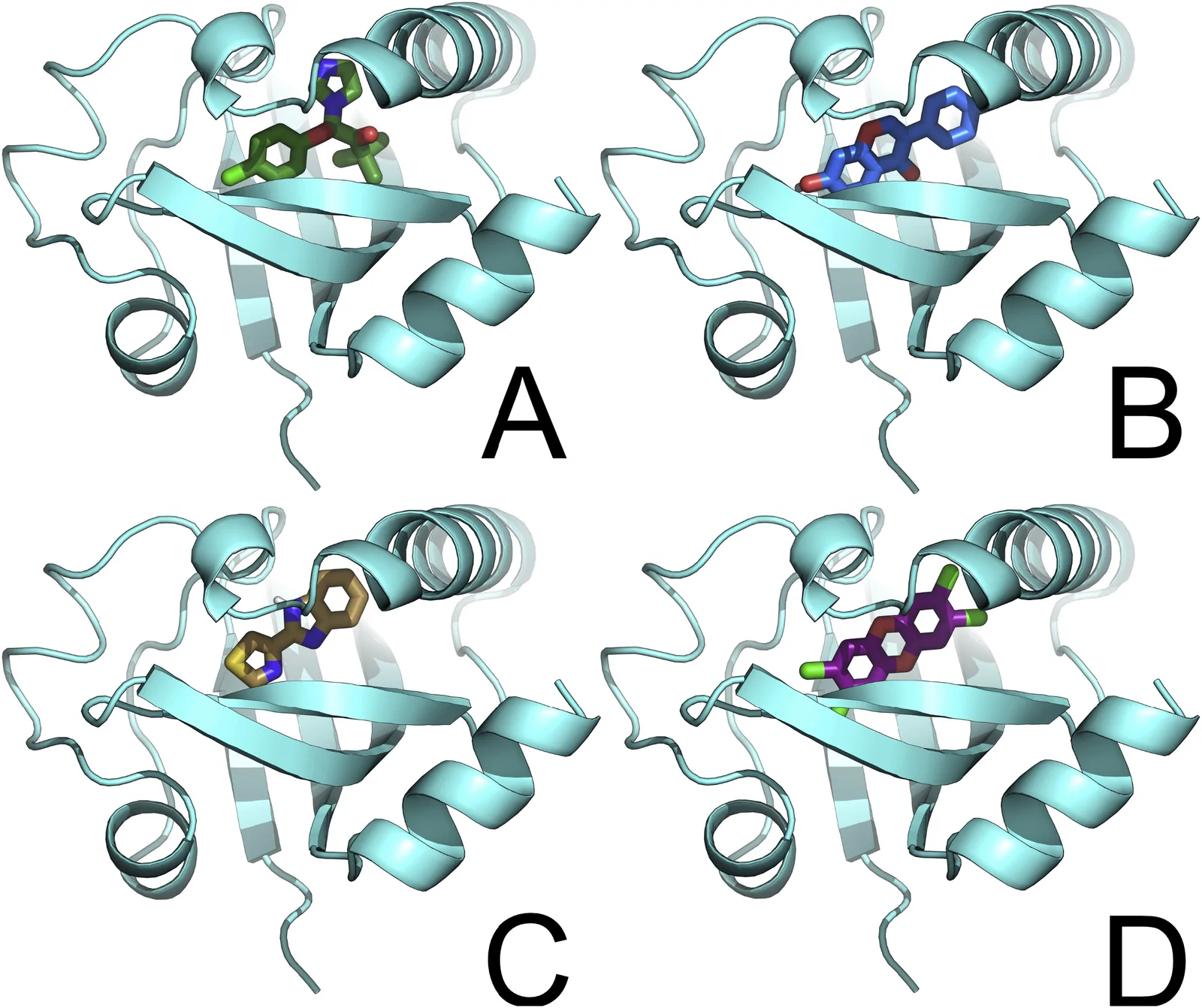
The best-energy poses of climbazole (CLB, green ligand, A), daidzein (DAI, blue ligand, B), thiabendazole (TBDZ, ochre ligand, C), and 2,3,7,8-Tetrachlorodibenzodioxin (TCDD, purple ligand, D) from site-directed docking to the PAS-B domain of human aryl hydrocarbon receptor shown as a cyan cartoon (hAhR, PDB ID 7ZUB). Up to ten best poses were computed, and the quantitative data are given in Table 8.

**Fig. 12:**
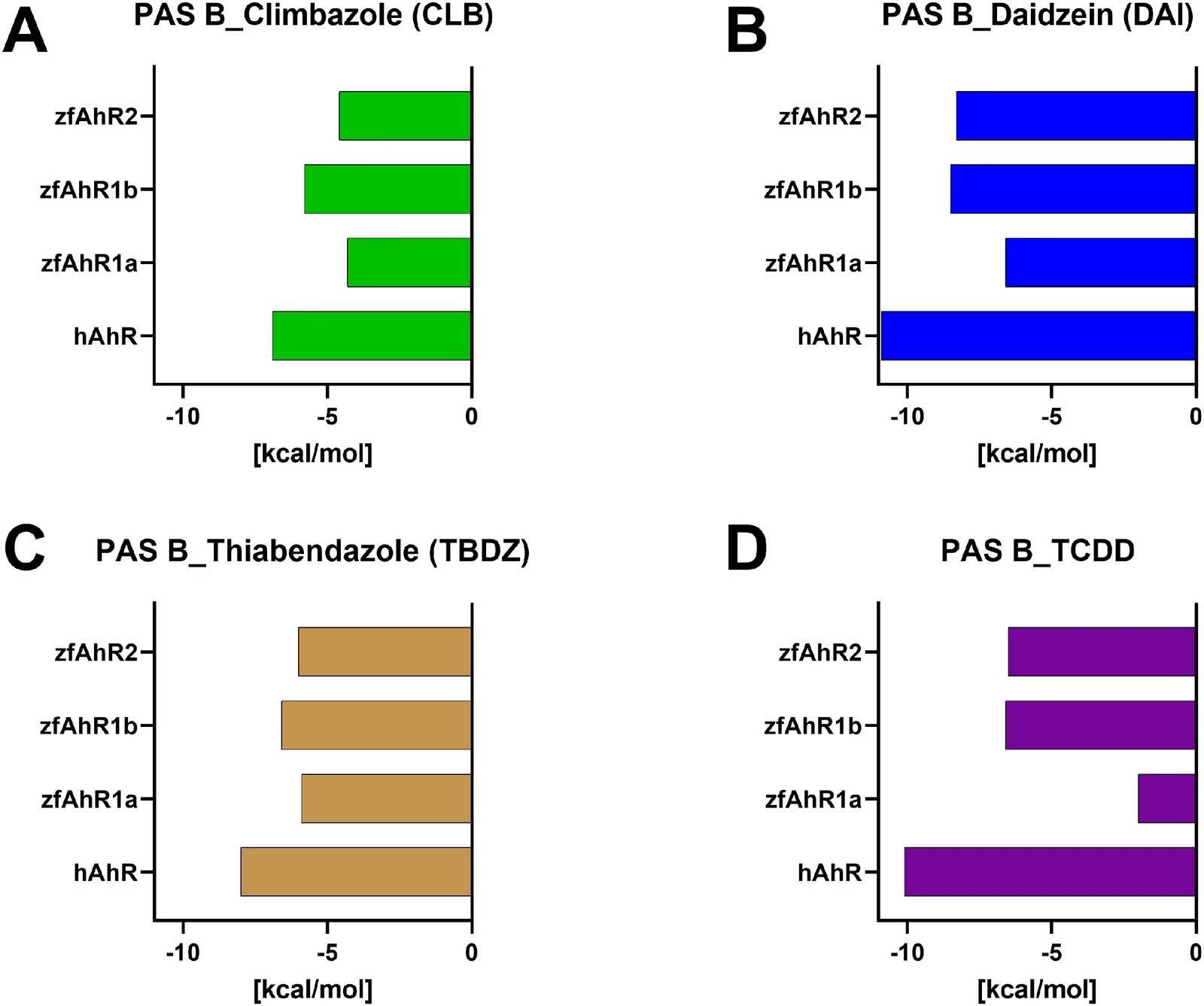
Illustration of the highest predicted binding energy poses per depicted compound derived from the PAS-B site-directed docking for all investigated AhR variants. Colour-coding corresponds to Table 8.

In our investigation of ligand-binding affinities, we assessed four distinct compounds: climbazole (CLB), daidzein (DAI), thiabendazole (TBDZ), and 2,3,7,8-Tetrachlorodibenzodioxin (TCDD). Fig. 11 illustrates the best energy binding pose per ligand within the hAhR PAS-B domain. The best energy poses of the remaining variants are given in Fig. 12 and in the SM (figs. S24–27). All computed binding poses per ligand and variant are summarised in Table 8. The resulting hierarchy of binding affinities across various AhR variants can be deduced as follows:

**Table 8:** Summary of the site-directed docking of Climbazole (CLB), Daidzein (DAI), Thiabendazole (TBDZ), and 2,3,7,8-Tetrachlorodibenzodioxin (TCDD) to the PAS-B domain of human (hAhR) and three zebrafish (zfAhR1a, zfAhR1b, zfAhR2) aryl hydrocarbon receptor variants.

Starting with CLB, we observed the following best binding energies: hAhR (−6.9 kcal/mol, fig. S24A), zfAhr1b (−5.8 kcal/mol, fig. S24C), zfAhR2 (−4.6 kcal/mol, fig. S24D), and zfAhR1a (−4.3 kcal/mol, fig. S24B). Second, for DAI, the best binding energies were: hAhR (−10.9 kcal/mol, fig. S25A), zfAhr1b (−8.5 kcal/mol, fig. S25C), zfAhR2 (−8.3 kcal/mol, fig. S25D), and zfAhR1a (−6.6 kcal/mol, fig. S25B). TBDZ exhibited the following best binding energies: hAhR (−8 kcal/mol, fig. S26A), zfAhr1b (−6.6 kcal/mol, fig. S26C), zfAhR2 (−6 kcal/mol, fig. S26D), and zfAhR1a (−5.9 kcal/mol, fig. S26B). Finally, for TCDD, the best binding energies were as follows: hAhR (−10.1 kcal/mol, fig. S27A), zfAhr1b (−6.6 kcal/mol, fig. S27C), zfAhR2 (−6.5 kcal/mol, fig. S27D), and zfAhR1a (−2 kcal/mol, fig. S27B).

Furthermore, the binding energy spans for each case can be summarised as: hAhR: CLB (−6.9 to −5.5 kcal/mol), DAI (−10.9 to −8.7 kcal/mol), TBDZ (−8 to −7.4 kcal/mol), TCDD (−10.1 to −9.6 kcal/mol); zfAhR1a: CLB (−4.3 to −2.7 kcal/mol), DAI (−6.6 to −4.4 kcal/mol), TBDZ (−5.9 to −4.2 kcal/mol), TCDD (−2 to −2 kcal/mol); zfAhr1b: CLB (−5.8 to −2.9 kcal/mol), DAI (−8.5 to −7 kcal/mol), TBDZ (−6.6 to −5.4 kcal/mol), TCDD (−6.6 to −5.5 kcal/mol); and zfAhR2: CLB (−4.6 to −2.1 kcal/mol), DAI (−8.3 to −7.8 kcal/mol), TBDZ (−6 to −5.4 kcal/mol), TCDD (−6.5 to −5.2 kcal/mol).

#### 3.2.2 Docking of various ligands to the ahr pas-b domain - summary

The hierarchy of the absolute best binding energy pose is preserved among variants, with hAhR showing the most significant susceptibility to all ligands, followed by zfAhR1b, zfAhR2, and, finally, zfAhR1a (Figs. 12 and S28A in the SM). Comparing between ligands, CLB shows the weakest affinity to all AhR variants, with one exception. For zfAhR1a, TCDD displays the lowest binding affinity to the receptor. CLB is followed by TBDZ, and finally, DAI. For zfAhR2 and zfAhR1b, the best energy poses of TBDZ are on par with TCDD. DAI shows the strongest affinity to all AhR variants in terms of the best pose binding energies. However, the span of recorded binding energies is more expansive than TCDD’s (fig. S28A).

**Fig. 13:**
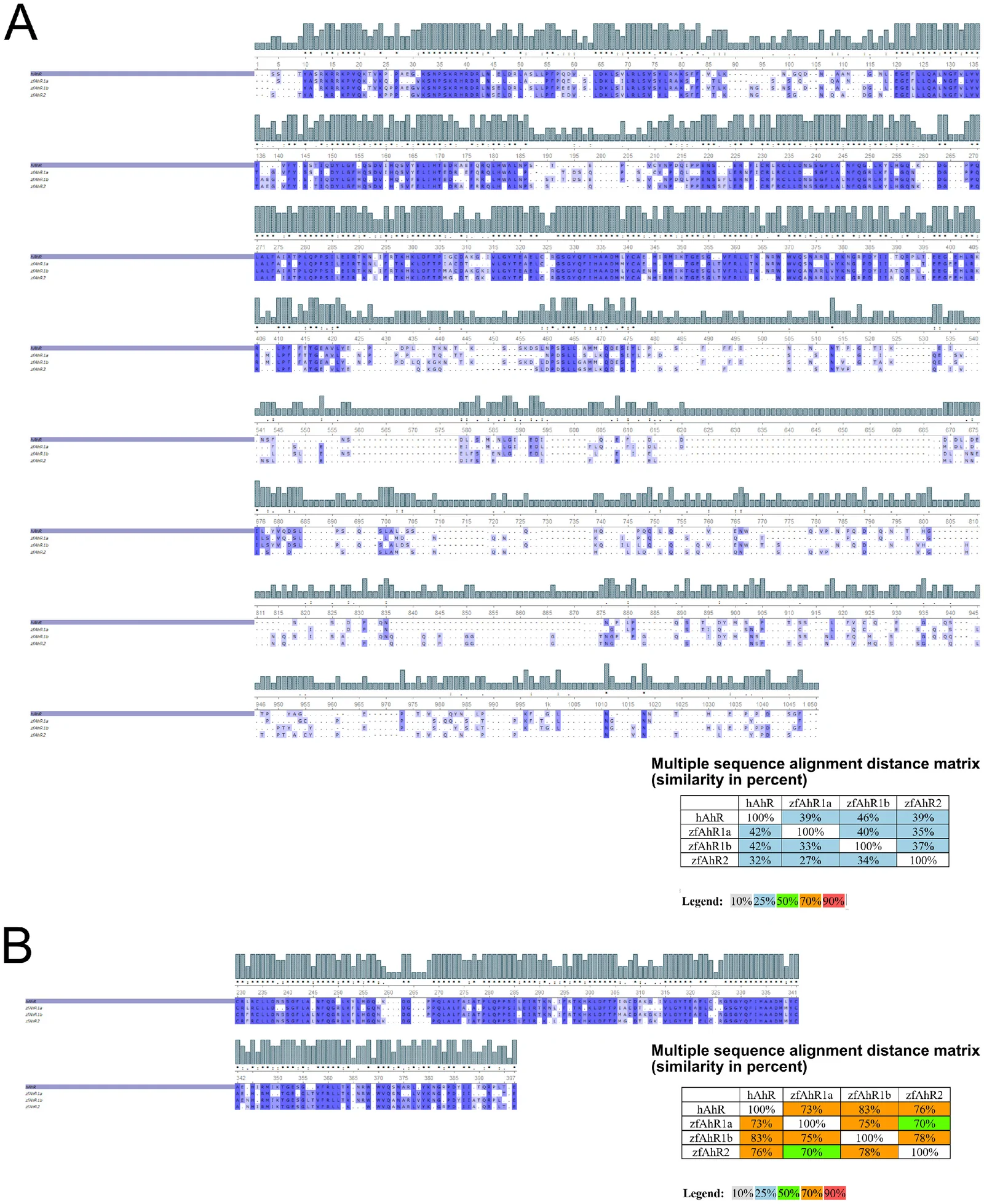
AhR amino acid (AA) sequence alignment. zfAhR1a, zfAhR1b, and zfAhR2 were aligned to hAhR as a reference. AA similarity is indicated by grey bars and blue-shaded highlights. (A) gives the entire sequence alignment; (B) is an excerpt of the proximity of the PAS-B ligand binding domain (residues 230–397). Multiple sequence alignment distance matrices are given in the respective corners. AA sequence alignment was conducted in Ugene, using the ClustalW algorithm.

When comparing AA sequence homology between hAhR and zebrafish variants, the overall sequence seems relatively less conserved, with zfAhR1a and 1b sharing 42% homology and zfAhR2 32% homology (Fig. 13A). However, when comparing the proximity of the ligand binding domain (residues 230 to 397), the numbers increase to 73% for zfAhr1a, 76% for zfAhR2, and 83% for zfAhr1b (Fig. 13B). Interestingly, the sequence homology coincides with the conserved binding hierarchy pattern (hAhR > zfAhr1b > zfAhR2 > zfAhR1a).

By plotting TCDD binding energy equivalents (dividing the ligand binding energy per pose by the average TCDD reference binding energy per receptor variant (see SI11 for calculations), one can compare susceptibility between receptors (fig. S28B+C). All ligands identified within the environmental samples are approximately 2 to 3 times more likely to bind to zfAhR1a than the reference ligand TCDD (fig. S28B). For zfAHR1b, all ligands show similar relative affinities as TCDD. In zfAhR2 and hAhR, binding energy equivalents are more diverse, with DAI showing higher, CLB and TBDZ lower affinity, than TCDD. The highest absolute binding energies were recorded for hAhR (DAI and TCDD, respectively). Therefore, when depicting hAhR TCDD binding energy equivalents, only DAI in hAhR shows similar affinity levels (fig. S28C).

In general, the highest absolute binding energies were found in hAhR. However, all receptor variants in zebrafish seem susceptible to the ligands identified within the environmental samples. Although the absolute binding energy hierarchy (hAhR > zfAhR1b > zfAhR2 > zfAhR1a) holds across all setups, in zebrafish, all receptor variants could potentially be activated within comparable margins.

In summary,

• All AhR variants showed susceptibility towards the identified environmental ligands and the reference compound TCDD.
• Except for zfAhR1a, the following susceptibility hierarchy was computed regarding the absolute best binding energy pose: DAI > TCDD > TBDZ > CLB.
• Regarding ligand affinity, the following hierarchy was computed for all receptor variants: hAhR > zfAhR1b > zfAhR2 > zfAhR1a. The latter also corresponds to AA-sequence homology in the vicinity of the PAS-B.

## 4 Experimental Design, Materials and Methods

For brevity and due to *Data in Brief* reference limitations, the experimental design, materials, and methods section provides only substantial information. For a more detailed overview, we refer to the SM, section 2.6. Additionally, cross-references to the SM within the following subchapters facilitate orientation.

### 4.1 Ligands and ligand preparation

Ligands were selected based on their ability to induce the investigated toxicity pathways, Keap1-Nrf2-ARE (oxidative stress response) and AhR-ARNT-XRE (xenobiotic metabolism), respectively. Commonly known reference compounds and previously identified environmental pollutants known to induce the latter pathways were chosen. As such, tert‑butylhydroquinone (tBHQ, CAS: 1948–33–0, fig. S2A in SM, section 2.6.1) and Metazachlor (MZC, CAS: 67,129-08–2, fig. S2B) were selected for the Keap1-Nrf2-ARE pathway, whereas 2,3,7,8-Tetrachlorodibenzodioxin (TCDD, CAS: 1746–01–6, fig. S2F), Climbazole (CLB, CAS: 38,083-17–9, fig. S2C), Daidzein (DAI, CAS: 486–66–8, fig. S2D), and Thiabendazole (TBDZ, CAS: 1746–01–6, fig. 2CE) were selected for the AhR-ARNT-XRE pathway. TCDD and tBHQ are considered to be reference inducers. The pesticide MZC has been identified in previous studies [[3], [4], [5]]. CLB (fungicide), DAI (naturally occurring isoflavone in food products), and TBDZ (fungicide and antiparasitic agent) were identified via effect-directed analysis of environmental samples within the main accompanying article.

We retrieved 3D structures of the above-named ligands from the PubChem database (https://pubchem.ncbi.nlm.nih.gov) and optimised them using the Avogadro 1.2.0 software (https://avogadro.cc) [10]. For parameter optimisation in Avogadro, see section 2.6.1 of the SM. All utilised PDBQT-type files are available in the supplementary folder SI10.

### 4.2 Receptor/Sensor selection, preparation, and sequence alignment

Keap1-sensor protein structures (oxidative stress toxicity pathway; fig. S3) were predicted by AlphaFold2 (https://alphafold.ebi.ac.uk) [11,12] through ColabFold (https://github.com/sokrypton/ColabFold) [13], from sequences obtained via UniProt (https://www.uniprot.org; hKeap1: Q14145, zfKeap1a: Q1ECZ2, zfKeap1b: A0A2R8Q1W5), as no experimental full-sequence protein structures were available. For more details on the hKeap1 structure fragments 7EXI and 1ZGK (fig. S3), which were employed in control predictions and sensor preparation, see section 2.6.2 in the SM.

For the human Ah-receptor protein structure (xenobiotic metabolism toxicity pathway, fig. S4), which encompasses the PAS-B ligand binding domain, the partial cryo-EM structure PDB ID 7ZUB, chain D [14] was used as a model. Zebrafish PAS-B ligand binding domains were predicted by AlphaFold2 from UniProt sequences (zfAhR1a: A0A8N7UZF6, zfAhR1b: Q4U3K9, and zfAhR2: Q9YGV3). For more details on the 7ZUB cryo-EM structure (fig. S4), human and zebrafish PAS-B domain localisation, and receptor preparation, consult the SM, section 2.6.2.

FASTA files (SI10) of the above-named Keap1 sensor and AhR receptor sequences were aligned and visualised in the Unipro Ugene software (https://ugene.net/) [15] using the ClustalW algorithm. Further alignment parameters are given in the SM, section 2.6.3.

### 4.3 Docking

Molecular docking was conducted with the AutoDock/Vina algorithm [16,17] in PyMOL (Schrödinger Inc., New York, US) by defining docking grids in proximity to the points of interest: the PAS-B domain of different species’ variants for AhR and several highly reactive cysteine residues for the Keap1 sensor variants. PyMOL was utilised to visualise the predicted binding poses, measure atomic distances, and render images. More details, especially on docking grid localisation, are given in the SM, section 2.6.5.

## Limitations

None.

## Ethics Statement

Not applicable; the manuscript does not contain clinical studies, patient data, or animal experiments. All authors acknowledged and followed the ethical requirements for publication in Data in Brief.

## Funding

This work was supported by the U-share collaboration grant for postdoctoral fellows and the support of RECETOX Research Infrastructure (ID LM2023069, MEYS CR, 2020-2022), European Union’s Horizon 2020 Research and Innovation Programme projects CETOCOEN (857560), and the Grant Agency of the Czech Republic (25-18233M). Computational resources were provided by e-INFRA CZ and Elixir CZ (90254 and LM2023055).

## CRediT authorship contribution statement

**Sebastian Lungu-Mitea:** Conceptualization, Methodology, Software, Validation, Formal analysis, Investigation, Data curation, Resources, Writing – original draft, Writing – review & editing, Visualization, Project administration, Funding acquisition. **Jana Horáčková:** Methodology, Software, Validation, Formal analysis, Investigation, Data curation, Writing – original draft, Writing – review & editing, Visualization. **David Bednář:** Conceptualization, Methodology, Software, Resources, Writing – review & editing, Funding acquisition. **Klára Hilscherová:** Resources, Writing – review & editing, Funding acquisition.

## Data Availability

Researchdata.se - Swedish National Data ServiceDataset connected to the project “Cross-species bioanalytical assessment of the oxidative stress and xenobiotic metabolism modes of action for environmental water samples” (Original data)

## Appendix Supplementary materials

Image, application 1
