# Supplementary material for: Human and zebrafish cross-species comparison on ligand affinity towards the aryl hydrocarbon receptor and Keap1 oxidative stress sensor: a molecular docking dataset
Source: Data Brief. 2026 Jul 7;68:113037. doi: 10.1016/j.dib.2026.113037 (PMC13571530; doi:10.1016/j.dib.2026.113037)
Supplement: Supplementary file 1 [file mmc1.pdf]

Supplementary manuscript relating to the project comprising:

Main article: Cross-species bioanalytical assessment of the oxidative stress and xenobiotic metabolism modes of action for environmental water samples – a multi-layered approach to testing and modelling

Sebastian Lungu-Mitea (a,b,c,d), Geeta Mandava (a), Jana Horackova (f,g), Oksana Golovko (e), Zuzana Tousova (b), David Bednar (f,g), Lutz Ahrens (e), Klara Hilscherova (b), Johan Lundqvist (a)

Data in Brief: A dataset on the multi-species and multi-endpoint bioanalytical assessment of environmental samples derived from Swedish wastewater treatment plants

Sebastian Lungu-Mitea (a,b,c,d), Geeta Mandava (a), Oksana Golovko (e), Kim Frieberg (a), Sara Alsayfi (a), Stefan Örn (a), Lutz Ahrens (e), Johan Lundqvist (a)

Data in Brief: Human and zebrafish cross-species comparison on ligand affinity towards the aryl hydrocarbon receptor and Keap1 oxidative stress sensor: a molecular docking dataset

Sebastian Lungu-Mitea (a,b,c,d), Jana Horackova (f,g), David Bednar (f,g), Klara Hilscherova (b)

Affiliations

(a) Department of Animal Biosciences, Molecular Toxicology, Swedish University of Agricultural Sciences (SLU), SE-75007 Uppsala, Sweden

(b) RECETOX, Faculty of Science, Masaryk University (MUNI), CZ-62500 Brno, Czech Republic

(c) Department of Pharmaceutical Biosciences, Drug Safety and Toxicology, Uppsala University (UU), SE-75124 Uppsala, Sweden

(d) Department of Biology and Environmental Science, Linnaeus University (LNU), SE-39182 Kalmar, Sweden

(e) Department of Aquatic Sciences and Assessment, Swedish University of Agricultural Sciences (SLU), SE-75007 Uppsala, Sweden

(f) Loschmidt Laboratories, Department of Experimental Biology and RECETOX, Masaryk University (MUNI), CZ-62500 Brno, Czech Republic

(g) International Clinical Research Centre, St. Anne's University Hospital Brno, CZ-60200 Brno, Czech Republic

*Corresponding author: sebastian.lungu@slu.se

ORCID IDs

DB: https://orcid.org/0000-0002-6803-0340

GM: https://orcid.org/0000-0003-2834-2259

JH: https://orcid.org/0000-0001-7119-121X

JL: https://orcid.org/0000-0001-5693-9007

KF: https://orcid.org/0000-0002-0043-4031

KH: https://orcid.org/0000-0001-6320-8093

LA: https://orcid.org/0000-0002-5430-6764

OG: https://orcid.org/0000-0003-3256-3054

SA: -

SLM: https://orcid.org/0000-0001-8192-9134

SÖ: https://orcid.org/0000-0002-0086-2124

ZT: https://orcid.org/0000-0001-7069-0471

INDEX

1. Concept, summary list, and abbreviations....5

Tab. S1: Description of supplementary information (SI) in the data repository....6

Tab. S2: List of abbreviations....7

2. Supplementary material and methods....9

2. 1 Environmental sample preparation and extraction....9

Fig. S1: A map indicating WWTP sites' A to C approximate locations in central and western Sweden....10

Tab. S3: Environmental sample and WWTP-related characteristics in detail....10

2.2 Instrumental chemical assessment....12

2.3 Bioanalytical assessment: reporter gene bioassays....12

2.3.1 Oxidative stress response reporter assay in zebrafish cell line....13

2.3.2 Oxidative stress reporter assay in human cell line....13

2.3.3 Xenobiotic metabolism response reporter assay in zebrafish cell line....14

2.3.4 Xenobiotic metabolism response reporter assay in human cell line....15

2.3.5 Xenobiotic metabolism response reporter assay in mouse cell line....16

2.3.6 Cell handling and plating....16

2.3.7 Transient transfection....16

2.3.8 Exposure....17

2.3.9 Viability and reporter-related readouts....18

2.3.10 MTS-based cellular viability assay....18

2.3.11 Luciferase-based reporter readouts....18

2.4 Bioanalytical assessment: the FET assay....19

2.5 In silico-mediated effect-directed analysis (ISEDA)....19

2.5.1 tcpl and MySQL R coding packages....19

2.5.2 OCHEM AhR-NR QSAR....20

2.6 Molecular docking: assessing receptor-ligand affinity....20

2.6.1 Ligands....20

Fig. S2: 2D representation of all selected ligands....21

2.6.2 Tertiary protein structure selection and prediction: Keap1 and AhR PAS-b domain....21

2.6.3 Amino acid sequence alignment....22

2.6.4 Topology, mapping, and nomenclature........22
Fig. S3: Topology of hKeap1........23
Fig. S4: Topology of hAhR........24
2.6.5 Molecular docking procedure........24
2.7 Quantification of bioavailable concentrations via mass-balance modelling........25
2.8 Data processing and statistical analyses........25
2.8.1 Reporter gene assays – raw data handling and normalisation........25
2.8.2 Reporter gene assays – regression analyses........26
2.8.3 Iceberg modelling........26
2.8.4 Removal efficiencies........27
2.8.5 Statistical analysis of molecular docking-derived data........27
2.8.6 Ratios........28
3. Supplementary results........28
3.1 Supplementary results: DIB_B co-submission........28
Fig. S5: Molecular docking (tBHQ)........28
Fig. S6: Molecular docking (tBHQ)........29
Fig. S7: Molecular docking (tBHQ)........30
Fig. S8: Molecular docking (tBHQ)........30
Fig. S9: Molecular docking (tBHQ)........31
Fig. S10: Molecular docking (tBHQ)........31
Fig. S11: Molecular docking (tBHQ)........32
Fig. S12: Molecular docking (tBHQ)........32
Fig. S13: Molecular docking (MZC)........33
Fig. S14: Molecular docking (MZC)........33
Fig. S15: Molecular docking (MZC)........34
Fig. S16: Molecular docking (MZC)........34
Fig. S17: Molecular docking (MZC)........35
Fig. S18: Molecular docking (MZC)........35
Fig. S19: Molecular docking (MZC)........36
Fig. S20: Molecular docking (MZC)........36
Fig. S21: Molecular docking (MZC)........37
Fig. S22: Molecular docking (MZC)........38
Fig. S23: Molecular docking (MZC)........38
Fig. S24: Molecular docking (CLB)........39
Fig. S25: Molecular docking (DAI)........40
Fig. S26: Molecular docking (TBDZ)........41

Fig. S27: Molecular docking (TCDD)....42
Fig. S28: Molecular docking summary....43
3.2 Supplementary results: Main article – Summarised cytotoxicity/cellular viability....44
Fig. S29: Cytotoxicity/cellular viability impact assessment of the environmental samples from WWTP sites A to C....44
Fig. S30: Cytotoxicity/cellular viability impact assessment of reference control and ISEDA-identified compounds....45
3.3 Supplementary results: Main article – Optimised data fitting strategies for the oxidative stress MoA....46
Fig. S31: Alternative data fitting (tBHQ, MZC)....48
Fig. S32: Alternative data fitting (tBHQ, MZC)....50
Fig. S33: Alternative data fitting (tBHQ, MZC)....51
3.4 Supplementary results: Main article – Optimised data fitting strategies for the xenobiotic metabolism MoA....52
Fig. S34: Alternative data fitting (TCDD)....53
Fig. S35: Alternative data fitting (TCDD)....54
Fig. S36: Alternative data fitting (TCDD)....55
Tab. S4: Effect estimates (EC10 or EC20) ratios, as derived from various DRE data model fits.. 56
Fig. S37: Alternative data fitting (TCDD)....57
Fig. S38: Alternative data fitting (TCDD)....59
Fig. S39: Alternative data fitting (TCDD)....60
3.5 Supplementary results: Main article – other supplementary results....61
Tab. S5: Summary of all effect point estimates ($EC_{IR1.5}$), their variation metrics, calculated BEQ values, and chemical removal efficiencies, as derived from the oxidative stress MoA assessment....61
Tab. S6: Summary of all effect point estimates (EC10), calculated BEQ values, and chemical removal efficiencies, as derived from the xenobiotic metabolism MoA assessment....61
Tab. S7: EC50 estimates computed herein vs. reported EC50 estimates within the literature. .62
Tab. S8: Percentage [%] of BEQ_Bio explained by BEQ_Chem....63
Tab. S9: Summary of all tested ISEDA compounds' concentration estimates (AC50 and EC10), including their variation metrics....63
Fig. S40: Alternative data fitting (ISEDA)....65
3.6 Supplementary results: Main article – Bioavailability assessment....66
Fig. S41: Outline of the bioavailability assessment....67
4. Supplementary discussion....67
4.1 The Nrf2-Keap1-ARE toxicity pathway – Canonical reactive cysteine residues and mechanistic insights on the "cysteine code"....67
4.1.1 Background and objectives of this project concerning Keap1....67

Fig. S42: Amount of total conserved cysteines within all Keap1 variants....69
4.1.2 The Nrf2-Keap1-ARE signalling pathway: nomenclature and mechanisms....69
4.1.3 Molecular docking results in the context of the “cysteine code” (see DIB_B for results)....71
4.2 Supplementary discussion: Comment on zfKeap1a/b functional divergence and structural variability....72
4.3 Supplementary discussion: “CALUX” assay nomenclature and identification....72
4.4 Supplementary discussion: Cross-species assessment in context with regulatory metrics – BEQs and EBTs....72
Fig. S43: Hypothetical TCDD-BEQ scenario....74
4.5 Supplementary discussion: TCDD as a reference compound, reasoning and scrutiny....74
4.6 Supplementary discussion: validity of molecular docking approach....76
4.7 Supplementary discussion: AhR crosstalk and non-canonical pathways....77
4.8 Supplementary discussion: outlook and further technicalities regarding multi-species reporter gene assays....77
Fig. S44: Outlook on short to long-term challenges....80
5. References....80

1. Concept, summary list, and abbreviations

The main article is supported by two *Data in Brief* co-submissions. As such, the co-submissions are self-reliant, but they supplement the primary article by providing raw data, specific methodologies, and first-tier analyses. Higher-tier analyses are exclusive to the main article. Readers are directed to these co-submissions for an in-depth review of datasets to avoid overburdening the main article. This supplementary manuscript at hand serves as a link between the main article and the co-submissions, describing the shared methodology in detail, as recommended by the *Data in Brief* guidelines. Moreover, the supplementary manuscript provides additional results, analyses, and discussions.

The first *Data in Brief* co-submission (hereafter, “DIB_A”; for abbreviations, see tab. S2) encompasses raw data and preliminary analysis (LOEC/NOEC) pertinent to the bioanalytical assessment of the investigated environmental samples. This includes additional samples beyond the scope of the main article and employs further test frameworks, specifically the fish embryo acute toxicity test (FET, OECD TG 236 [1]). The data in DIB_A are related to the SI files SI1-SI8.

The second *Data in Brief* co-submission (hereafter, “DIB_B”) incorporates comprehensive molecular docking models along with protein sequence alignment studies. It also features confirmatory analyses that compare AlphaFold-predicted structures with those derived from cryo-EM and X-ray

crystallography. DIB_B addresses the consequential implications and is associated with SI files SI9 through SI11.

All raw and computed metadata across the manuscripts are consolidated in a shared data repository (https://doi.org/10.5878/zwnq-qb88), which contains 16 Supplementary Information files (hereafter, "SI") and this supplementary manuscript (hereafter, "SM"). These files are numbered chronologically, starting with SI12, which contains data explicitly related to the main article (tab. S1).

Tab. S1: Description of supplementary information (SI) in the data repository. (https://doi.org/10.5878/zwnq-qb88)

Name	Content	Associated Article
SI1	Raw and analysed data of all experiments conducted with the ZF4 assay	DIB_A
SI2	Raw and analysed data of all experiments conducted with the MCF7/AREc32 assay	DIB_A
SI3	Raw and analysed data of all experiments conducted with the ZFL assay	DIB_A
SI4	Raw and analysed data of all experiments conducted with the HepG2 assay	DIB_A
SI5	Raw and analysed data of all experiments conducted with the DREcoScreen assay	DIB_A
SI6	Raw, analysed data, and statistical interference of the mFET assay	DIB_A
SI7	Transformation and statistical interference conducted in all cellular bioassays	DIB_A
SI8	Transformation and statistical interference conducted for all pairwise comparisons between assays/species	DIB_A
SI9	Summarised binding energies	DIB_B
SI10	PDBQT and FASTA-type files of ligands and receptors	DIB_B
SI11	TCDD-energy-equivalent calculations	DIB_B
SI12	Physico-chemical properties of all targeted compounds detected in chemical analysis; measured chemical concentration of targeted compounds per environmental sample; calculated REP values, extracted ToxCast data; OCHEM AhR QSAR outputs; bioanalytics for AhR-QSAR positive compounds; BEQ-Chem calculations.	Main article SM
SI13	Bioavailability (C_free concentrations) data generated via linear solvation energy relationships QSPRs from UFZ-LSER	Main article SM
SI14	Metazachlor reference compound standard curves, cytotoxicity data, and all respectively raw data generated	Main article SM
SI15	Raw and metadata from the bioanalytical assessment of ISEDA-identified chemical compounds	Main article SM
SI16	BEQ-Bio calculations relating to estimates derived via 3PNL regression analysis (main article) and all other applied data-fitting scenarios (supplementary manuscript)	Main article SM

Tab. S2: List of abbreviations	
2PNL	Two-parametric non-linear regression
30%SL	Refitted simple linear regression using the first 30% curve estimates of the 3PNL model
3PNL	Three-parametric non-linear regression
4PNL	Four-parametric non-linear regression
A/B/C_X	Coding of environmental samples
AA	Amino acid
AC50	Concentration at which a compound produces 50% of its maximal observed activity in a specific assay system
AhR	Aryl hydrocarbon receptor
ARE	Antioxidant response element
ARNT	Aryl hydrocarbon receptor nuclear translocator protein
BEQ	Bioanalytical equivalents
BTB	Broad complex, tramtrack, and bric-a-brac domain
CI	Confidence interval
CLB	Climbazole
CRC	Concentration-response curve
cryo-EM	Cryogenic electron microscopy
Ctrl	Solvent control
CysX	Cysteine residue no. X
DAI	Daidzein
DIB_A	Data in Brief co-submission A
DIB_B	Data in Brief co-submission B
DLC	Dioxin-like compounds
DLR	Dual luciferase assay
DMSO	Dimethyl sulfoxide
EBT	Effect-based trigger value
EC10	Effective concentration of a sample causing 10% of the effect in relation to the reference compound
EC50	Effective concentration of a sample causing 50% of the effect in relation to the reference compound

ECIR1.5	Effect concentration causing a 1.5x induction ratio (vs. Control)
EROD	Ethoxyresorufin-O-deethylase activity (CYP1A activity)
FBS	Fetal bovine serum
FET	Fish embryo acute toxicity test
Fig. Sx	Supplementary figure X
Fluc	Firefly luciferase
GSR	Glutathione reductase activity
hpf	Hours post-fertilisation
ISEDA	In silico-mediated effect-directed analysis
IVR	Intervening region domain
Keap1	Kelch-like ECH-associated protein 1; human (h) or zebrafish (zf) variant
Kelch domain	Five to seven kelch tandem repeats that form a β-propeller tertiary structure
LOEC	Lowest observed effect concentration
LR	(single) luciferase assay
mFET	Modified zebrafish embryo test
MoA	Mode of action
MTS	Dimethyl-thiazol-sulfophenyl tetrazolium-based cytotoxicity assay
MZC	Metazachlor
NC	Negative control (solvent control)
NOEC	No observed effect concentration
Nrf2	Nuclear factor erythroid 2-related factor 2
PAS	Per-Arnt-Sim domain
PDB ID 1ZGK, 7EXI, 7ZUB	Protein database identifier of crystal or cryo-EM structures
PPARγ	Peroxisome Proliferator-Activated Receptor gamma
PXR	Pregnane X receptor
REF	Relative enrichment factor
Rluc	Renilla luciferase
SEM	Standard error of mean

SI	Supplementary information
SL	simple linear regression
SM	Supplementary manuscript
TBDZ	Thiabendazole
tBHQ	tert-Butylhydroquinone
TCDD	2,3,7,8-tetrachlorodibenzodioxin
WWTP	Wastewater treatment plant
XRE	Xenobiotic response element, also known as DRE - dioxin response element

2. Supplementary material and methods

2. 1 Environmental sample preparation and extraction

Water samples were collected from three wastewater treatment plants (WWTPs) in central and western Sweden, with downstream catchments identified as areas of anthropogenic (drinking water production, leisure, and food consumption) and ecological (water protection areas and Natura 2000 sites) interest (fig. S1). Additionally, the three WWTP sites were selected based on a prior commercial bioanalytical screening campaign (unpublished data due to confidentiality), in which these samples showed comparatively strong induction of Nrf2/Keap1/ARE- and AhR/ARNT/XRE-mediated responses. Site selection was therefore intentionally targeted toward bioactive wastewater samples to enable potential quantitative cross-species assay comparison across both investigated MoAs, rather than to provide a generally representative survey of Swedish WWTPs.

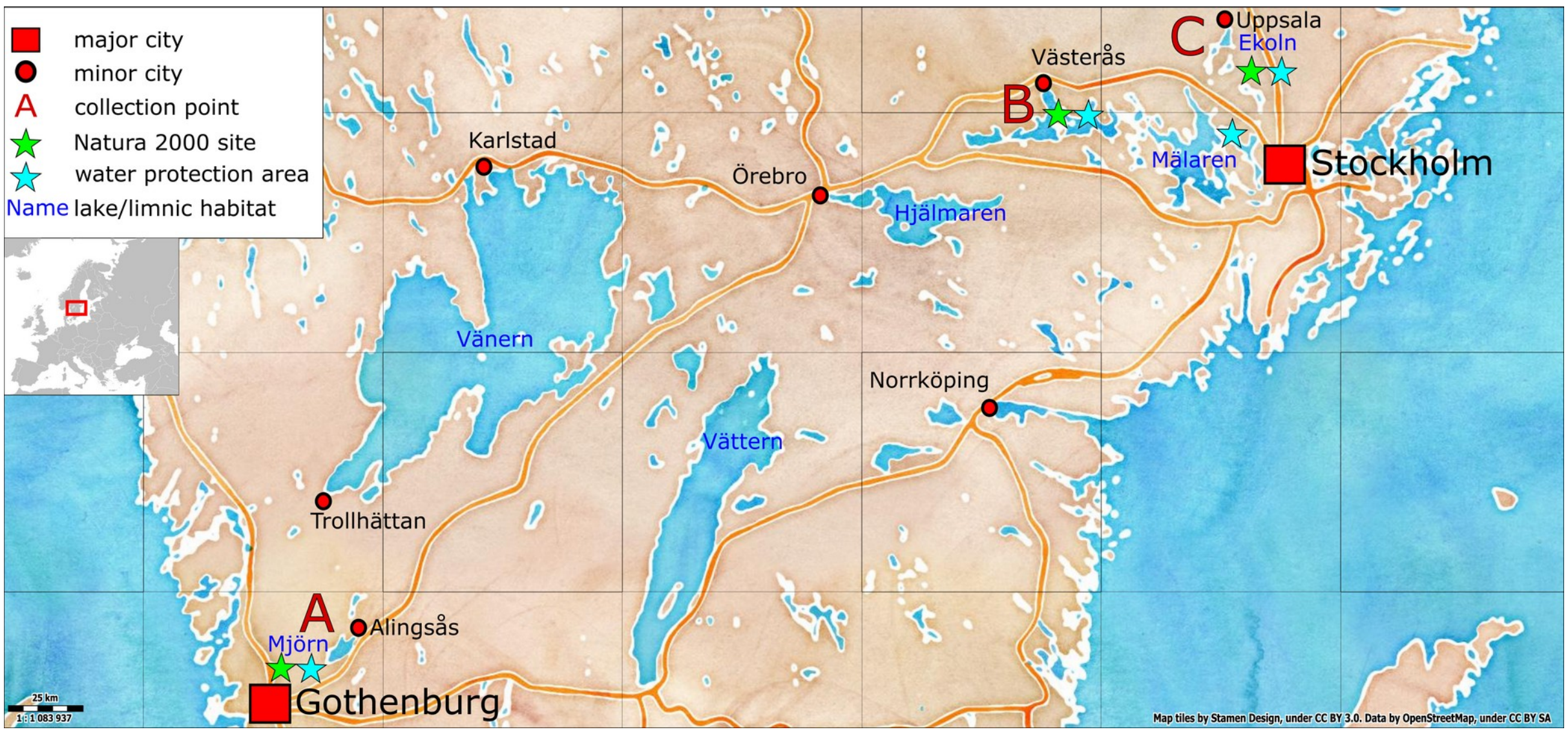

Fig. S1: A map indicating WWTP sites' A to C approximate locations in central and western Sweden, including ecological and anthropogenic concern protection areas.

Influent and effluent samples were collected as one- to seven-day composite samples during the summer (June). Surface water samples were collected as grab samples 10 cm below the water surface, contemporaneous with the WWTP samples. The latter samples were collected into 1 L high-density polyethene containers, snap-frozen, and stored until usage or analysis at -20°C. Tab. S3 gives further details on the respective environmental samples. The precise geographic locations are disclosed due to legal confidentiality.

Environmental sample extraction was performed using an automatic solid-phase extraction (SPE) system (SPE-DEX, Horizon Technology, Salem, NH, USA) with HLB extraction disks (Atlantic HLB-H Disks, diameter 47 mm; Horizon Technology, Salem, NH, USA). HLB extraction disks were conditioned with 280 mL of MeOH and 420 mL of Millipore water. After conditioning, approximately 1 L of the environmental samples was loaded onto the disks at a flow rate of 50 mL/min. Loaded HLB disks were washed twice with 24 mL of 5% (v/v) MeOH/95% Millipore water, dried under vacuum for 30 min, and eluted three times with 25 mL of MeOH, for a total volume of 75 mL. Furthermore, eluates were evaporated under a gentle nitrogen stream at 35 °C. The residue was dissolved in 0.5 mL of anhydrous dimethyl sulfoxide (DMSO;≥99.9% purity, sterile; Sigma-Aldrich, Steinheim, Germany), yielding a 2000-fold enrichment factor. The same volume of Millipore water was concentrated by solid-phase extraction to serve as an operational control.

The overall loading volume for the environmental samples was 1 L. However, for sample IDs A_I and B_I, only 900 and 980 mL could be recovered, respectively, resulting in slightly changed relative enrichment factors (REFs) in the later bioassays (tab. S3). Enrichment and dilution factors were calculated according to Escher et al. [2].

Relative enrichment factor (REF) = $\textit{enrichment factor}_{\text{SPE}}$ * $\textit{dilution factor}_{\text{bioassay}}$

Enrichment $\text{factor}_{\text{SPE}} = V_{\text{water}}/V_{\text{extract}}$

Dilution $\text{factor}_{\text{bioassay}} = V_{\text{extract in bioassay}}/V_{\text{total in bioassay}}$

A REF >1 implies that the water sample is concentrated, while a REF <1 implies that the water sample is diluted compared to the original environmental sample.

Tab. S3: Environmental sample and WWTP-related characteristics in detail. Samples were collected in June 2018.

ID	WWTP site	Sample type	Catchment	Sampling method	Waste source	Person equivalent	Inflow/outflow [m^3/day]	Treatment	SPE enrichment factor
A_RU	A	Recipient upstream	Mjörn (water protection area, Natura 2000 site)	Grab	90% households, 10 % industries	30000	-	-	2000
A_I	A	Influent	Mjörn (water protection area, Natura 2000 site)	5-day composite	90% households, 10 % industries	30000	400	-	1800
A_E	A	Effluent	Mjörn (water protection area, Natura 2000 site)	5-day composite	90% households, 10 % industries	30000	400	Roughing, 1st sand filter, 1st clarifier, aeration/bioreactor, 2nd clarifier, digester, reject water influent to WWTP	2000
A_RD	A	Recipient downstream	Mjörn (water protection area, Natura 2000 site)	Grab	90% households, 10 % industries	30000	-	-	2000
B_RU	B	Recipient upstream	Mälaren (water protection area, Natura 2000 site)	Grab	92% households, 8 % industries	111105	-	-	2000
B_I	B	Influent	Mälaren (water protection area, Natura 2000 site)	7-day composite	92% households, 8 % industries	111105	39888	-	1960
B_E	B	Effluent	Mälaren (water protection area, Natura 2000 site)	7-day composite	92% households, 8 % industries	111105	39888	Roughing, 1st sand filter, 1st clarifier, aeration/bioreactor, 2nd clarifier	2000

B_RD	B	Recipient downstream	Mälaren (water protection area, Natura 2000 site)	Grab	92% households, 8 % industries	111105	-	-	2000
C_RU	C	Recipient upstream	Ekoln (water protection area, Natura 2000 site)	Grab	Not defined	160000	-	-	2000
C_I	C	Influent	Ekoln (water protection area, Natura	1-day composite	Not defined	160000	37100	-	2000
C_E	C	Effluent	Ekoln (water protection area, Natura 2000 site)	1-day composite	Not defined	160000	37100	Roughing, 1st sand filter, 1st clarifier, aeration/bioreactor, 2nd clarifier, iron chloride treatment, eventually chlorine dosing	2000
C_RD	C	Recipient downstream	Ekoln (water protection area, Natura 2000 site)	Grab	Not defined	160000	-	-	2000

2.2 Instrumental chemical assessment

The environmental samples were analysed on a DIONEX UltiMate 3000 ultra-high pressure liquid chromatography (UPLC) system (Thermo Scientific, Waltham, MA, USA) coupled to a triple quadrupole mass spectrometer (MS/MS) (TSQ QUANTIVA, Thermo Scientific, Waltham, MA, USA). For method performance on linearity, limits of quantification, precision, blanks, and additional physicochemical properties of the targeted compounds, please consult Golovko et al. [3] and SI12.

Notably, technical replicates of the same environmental samples, amongst others, were spiked with isotopically labelled standards and target-analysed for 165 chemicals of emerging concern. The results have been previously published by Golovko et al. [3] and are described in detail there.

2.3 Bioanalytical assessment: reporter gene bioassays

From the three investigated WWTPs (A to C), influent and effluent samples were analysed by five reporter gene assays in cell cultures derived from three different species (zebrafish, human, and mouse). These assays cover two modes of action (MoA; activation of oxidative stress defence via Nrf2-Keap1-ARE interaction (see, e.g., [4]) and activation of the xenobiotic metabolism via AhR-ARNT-XRE interaction (consult, e.g., [5])). For the interested reader, section 4.1 of the SM (below) offers a comprehensive review of the Nrf2-Keap1-ARE-mediated cellular stress response in the context of the molecular docking results (see sections 2.6 and 3.1).

2.3.1 Oxidative stress response reporter assay in zebrafish cell line

The oxidative stress response assay is based on the embryonic zebrafish fibroblast cell line ZF4 (CVCL_3275) [6]), which is transiently transfected with the pGL4.37[luc2P/ARE/Hygro] (Promega, Madison, USA) and pRL-CMV plasmid vectors. The basic ZF4 cell line was purchased from ATCC (Manassas, USA). The pGL4.37[luc2P/ARE/Hygro] plasmid vector bears four copies of a consensus Nrf2-responsive antioxidant response element (ARE) driving transcription of the primary Firefly (*Photinus pyralis*) luciferase reporter gene luc2P. The pRL-CMV plasmid vector drives the constitutive expression of the secondary Renilla luciferase reporter gene Rluc (*Renilla reniformis*), under the control of a cytomegalovirus (CMV) promoter. The secondary RLuc reporter normalises transfection efficiency within the transient setup (DLR, “dual luciferase assay”). Detailed descriptions of the assay's development and format are given in previous publications [7], [8].

Transient transfection was carried out 24h after initial cell plating on 96-well plates after the cells reached a confluency of about 80% (see further details in the sections 2.3.6 and 2.3.7 below) at a 2 µg transfection reagent to 1 µg plasmid DNA ratio per reaction using FugeneHD (Promega, Madison, USA). The FugeneHD transfection reagent increases cellular competence via lipid fusion. Transfection preconditioning was performed in OptiMEM medium (Gibco, Paisley, UK) according to the manufacturer's instructions. Primary and secondary reporter plasmids were co-transfected in a 10:1 ratio (0.9 µg reporter plasmid, 0.1 µg control plasmid). Transfection optimisation experiments were conducted according to the manufacturer's guidelines and have been reported previously [7], [8].

ZF4 cells were cultured in Dulbecco's Modified Eagle's Medium/Ham's Nutrient Mixture F-12 (DMEM: F12, nr. 21331020) containing phenol red (Gibco, Paisley, UK), supplemented with 10% (v/v) fetal bovine serum (Gibco, Paisley, UK), 1% (v/v) penicillin-streptomycin 100 U/mL (Gibco, Paisley, UK), 2.5 mM L-glutamine (Lonza, Basel, Swiss), 15 mM HEPES (Gibco, Paisley, UK), 0.5 mM sodium pyruvate (Sigma-Aldrich, Steinheim, Germany), and 1200 mg/L sodium bicarbonate (Gibco, Paisley, UK). The cells were cultured in a humidified environment at 28°C and with 5% CO_2. The cells were passaged

weekly at a 1:10 subculture ratio, using phosphate-buffered saline (PBS; pH 7.4; Gibco, Paisley, UK) for washing and 0.25% (w/v) trypsin (Sigma-Aldrich, Steinheim, Germany) for detachment.

2.3.2 Oxidative stress reporter assay in human cell line

The MCF7/AREc32 (AREc32) reporter line (CVCL_1D32) was obtained from Ximbio (London, UK). The reporter line is based on the human mammary carcinoma cell line MCF7 [9] (CVCL_0031), which was stably transfected with the pGL3-8xARE plasmid vector and named AREc32 [10]. The plasmid vector drives the expression of a Firefly (*Photinus pyralis*) luciferase reporter gene luc+, controlled by eight copies of the rat GST gene's antioxidant response element (gstrARE).

MCF7 cells were cultured in high-glucose (4.5 g/L D-glucose) Dulbecco's Modified Eagle's Medium (DMEM GlutMAX-I, nr.32430027) (Gibco, Paisley, UK), supplemented with 10% (v/v) fetal bovine serum (Gibco, Paisley, UK), 1% (v/v) penicillin-streptomycin 100 U/mL (Gibco, Paisley, UK), and 0.8 mg/mL geneticin (G418) selective antibiotic (Invivogen, CA, USA). The cells were cultured in the above medium, and whilst plating, they were grown in the same composition medium without the antibiotic G418. The cells were maintained in an incubator at 37 °C under a humidified 5% CO2 atmosphere and passaged twice a week, with medium changes every three days. Phosphate-buffered saline (PBS; at pH 7.4; Gibco, Paisley, UK) was used for washing and 0.05% (w/v) trypsin-EDTA (Gibco) for detachment.

2.3.3 Xenobiotic metabolism response reporter assay in zebrafish cell line

The reporter assay is based on the zebrafish hepatocyte cell line ZFL [11] (CVCL_3276), transiently transfected with the pGudLuc7.5 and pRL-null[zfEF1aPro] plasmids [12]. The basic ZFL cell line was purchased from ATCC (Manassas, USA). The pGudluc7.5 plasmid vector was donated by Michael S. Denison (University of California, Davies, USA; *in memoriam*: https://toxchange.toxicology.org/blogs/pamela-lein1/2022/06/30/in-memory-of-michael-steven-denison). The latter consists of a pGL3 backbone, 20 xenobiotic response element (XRE) copies from the genomic promoter region of the mCyp1A1 gene upstream of an MMTV viral promoter, and the Firefly (*Photinus pyralis*) luciferase Fluc reporter gene [13], which was employed as the primary reporter. The zebrafish-optimised pRL-null[zfEF1aPro] plasmid vector drives the constitutive expression of the secondary Renilla luciferase reporter gene (*Renilla reniformis*) Rluc, regulated by the endogenous zebrafish translation elongation factor 1 alpha promoter (zfEF1aPro). The secondary RLuc reporter normalises transfection efficiency within the transient setup (DLR). Further details on the assay are provided by Lungu-Mitea et al. [12].

Transient transfection was carried out 24h after initial cell plating in 96-well plates, when the cells reached about 80% confluency (see further details in sections 2.3.6 and 2.3.7 below), at a 3 µg

transfection reagent to 1 µg plasmid DNA ratio per reaction using jetPRIME (Polyplus, Illkirch, France). The jetPRIME transfection reagent increases cellular competence via a proprietary disclosed mechanism. Transfection pre-conditioning was conducted in the kit-specific reaction buffer according to the manufacturer's instructions. Primary and secondary reporter plasmids were co-transfected in a 10:1 ratio (0.9 µg reporter plasmid, 0.1 µg control plasmid). Transfection optimisation experiments were conducted according to the manufacturer's guidance, as previously reported [7]. Note that we changed our original protocol from using a competitor's transfection reagent to jetPRIME, as the latter does not induce systemic toxicity in the affected cells (data not shown).

The ZFL cell line was cultured in a medium made from powder, consisting of 50% (v/v) Leibovitz's L-15 (Sigma-Aldrich, Steinheim, Germany), 35% (v/v) Dulbecco's modified Eagle's medium (Gibco, Paisley, UK), 15% (v/v) Ham's Nutrient Mixture F-12 (Gibco, Paisley, UK), and phenol red. Additionally, 150 mg/L sodium bicarbonate (Gibco, Paisley, UK), 15 mM HEPES (Gibco, Paisley, UK), 10 µg/mL bovine insulin (Sigma-Aldrich, Steinheim, Germany), 50 ng/mL mouse EGF (Sigma Aldrich, Steinheim, Germany), and 5% (v/v) fetal bovine serum (Gibco, Paisley, UK) were supplemented. The cells were cultured in a humid environment at 28°C and at atmospheric CO_2. The cells were passaged weekly at a 1:20 ratio, using PBS (pH 7.4; Gibco, Paisley, UK) for washing and 0.25% (w/v) trypsin-EDTA (Sigma-Aldrich, Steinheim, Germany) for detachment.

2.3.4 Xenobiotic metabolism response reporter assay in human cell line

The reporter assay is based on the human hepatoma cell line HepG2 [14] (CVCL_0027), transiently transfected with the pGudLuc7.5 (see section 2.3.3 above for details) and the pGL4.70 (Promega, Madison, USA) plasmid vectors. As such, the transient reporter assay is identical to the stably transfected human AhR CALUX-type clone HG2L7.5c1 [15], with 2,3,7,8-tetrachlorodibenzodioxin (TCDD)-induced EC50 within the same order of potency (2e-10 and 5e-10 M, respectively). Note that several versions of the AhR CALUX-type assays are derived from clones of different species (rat, mouse, human, and guinea pig). The Tox21 dataset is based on the human clone HG2L7.5c1 (https://tripod.nih.gov/tox/assays). The pGL4.70 plasmid vector drives the constitutive expression of the secondary "humanised" Renilla luciferase reporter gene (*Renilla reniformis*) hRluc, which is optimised for expression within human cell lines and regulated via a minimal artificial promoter (minP). The secondary hRLuc reporter normalises transfection efficiency within the transient setup (DLR).

Transient transfection was carried out 48h after initial cell plating on 96-well plates when the cells reached a confluency of about 50% (see details in the sections 2.3.6 and 2.3.7 below) at a 0.3 µl transfection reagent to 100 ng plasmid DNA ratio per reaction using Lipofectamine 2000 (Invitrogen,

Thermo Fisher Scientific, USA). The Lipofectamine 2000 transfection reagent increases cellular competence via lipid fusion. Transfection preconditioning was performed in OptiMEM medium (Gibco, Paisley, UK) according to the manufacturer's instructions. Primary and secondary reporter plasmids were co-transfected in a 10:1 ratio (0.9 µg reporter plasmid, 0.1 µg control plasmid). Transfection optimisation experiments were conducted according to the manufacturer's guidance, as reported previously [16].

HepG2 cells were cultured in high-glucose Dulbecco's Modified Eagle Medium (DMEM, nr.31053028) (Gibco, Paisley, UK), supplemented with 10% (v/v) fetal bovine serum (Gibco, Paisley, UK), 1% (v/v) penicillin-streptomycin 100 U/mL (Gibco, Paisley, UK), and 2 mM L-glutamine (Gibco, Paisley, UK). The cells were maintained in an incubator at 37 °C in a humidified atmosphere of 5% CO_2. The medium was changed every 3 days, and cells were passaged twice a week. PBS (pH 7.4; Gibco, Paisley, UK) was used for washing, and 0.05% (w/v) trypsin-EDTA (Gibco) for detachment.

2.3.5 Xenobiotic metabolism response reporter assay in mouse cell line

The stably transfected reporter line DREcoScreen (DRE) [17] (CVCL_EG66) was obtained from Hiro Biotech (Singapore) via the Japanese Collection of Research Bioresources (Ibaraki, Osaka, Japan) and is based on the mouse hepatoma cell line Hepa1c1c7 [18] (CVCL_0328). DRE has been stably transfected with the pIND-DCDR7 plasmid vector driving the expression of a Firefly (*Photinus pyralis*) luciferase reporter gene luc+, under the control of seven copies of the mouse CYP1A1 gene's xenobiotic response element (cypmXRE).

DRE cells were cultivated in α-Minimum Essential Media (α-MEM, nr.12561) (Gibco, Paisley, UK), supplemented with 5% (v/v) fetal bovine serum (Gibco, Paisley, UK), 1% (v/v) penicillin-streptomycin 100 U/mL (Gibco, Paisley, UK), and 150 µg/mL Hygromycin B gold (InvivoGen, USA). The cells cultured in flasks were grown in the above medium, while cells plated for assays were grown in the composition medium without the antibiotic Hygromycin. The cells were maintained in an incubator at 37 °C in a humidified atmosphere of 5% CO_2. The medium was changed every three days, and cells were passaged twice a week. PBS (pH 7.4; Gibco, Paisley, UK) was used for washing, and 0.05% (w/v) trypsin-EDTA (Gibco) for detachment.

2.3.6 Cell handling and plating

Cells of running cultures were seeded on the first day of the experiment onto white, clear-bottom 96-well microtiter plates (Corning, New York, USA), in 100 µL/well culture medium, at different cell concentrations, regarding the used cell line; ZF4, $2.5*10^5$ cells/mL; AREc32, $1.5*10^5$ cells/mL; ZFL, $1.5*10^5$ cells/mL; HepG2, $1.5*10^5$ cells/mL; DRE, 10^5 cells/mL. Afterwards, plated cells were returned to the incubator for 24h.

2.3.7 Transient transfection

After 24h, cultures of ZF4 and ZFL reached approximately 80% confluency, whereas 48h cultures of HepG2 reached 50% confluency and were subjected to transient transfection according to the manufacturer's instructions and specific optimisations (see sections above for particular assays). After assay-specific pre-transfection treatment, 10 µL/well transfection mix in reduced medium was added to the cells, containing 100 ng/well pooled plasmid DNA and the respective ratio amount of transfection reagent. Subsequently, the cells were returned to the incubator for 24 h. Stably transfected lines AREc32 and DRE did not follow this procedure.

2.3.8 Exposure

The following day, the culture medium was removed, and the cells were exposed to the respective regimen for 24 h at 100 µL/well. After 24h of transfection in ZFL, ZF4, and HepG2 cells, the cells were exposed, whereas DRE and AREc32 cells were treated directly after 24h of plating. For the oxidative stress assays (ZF4 and AREc32), cells were exposed to four concentrations of the respective environmental samples at 1:1 dilutions, starting at REF 20 (REF 20, 10, 5, and 2.5). For the xenobiotic metabolism assays (ZFL, HepG2, and DRE), cells were exposed to four concentrations of the respective environmental samples at 1:1 dilution ratios, starting at REF 2 (REF 2, 1, 0.5, and 0.25). Cells were exposed in triplicate or quadruplicate per concentration. Each experiment was replicated at least three times. DRE cells were exposed only to influent samples from the three WWTP sites of interest. Note that A_I and B_I sample concentrations differed slightly due to initially varying SPE enrichment factors.

Additionally, a standard reference compound control was applied to every plate. tert-butylhydroquinone (tBHQ; CAS 1948-33-0; 97% purity; 100 µM stock in DMSO) (Sigma-Aldrich, St. Louis, USA) was utilised as a reference compound of the oxidative stress MoA, whereas 2,3,7,8-tetrachlorodibenzodioxin (TCDD; CAS 1746-01-6; 10 µg/ mL in toluene; Supelco analytical standard; 32 µM stock in DMSO) (Sigma- Aldrich, Steinheim, Germany) for the xenobiotic metabolism. Stock solutions were stored at -20 °C in a desiccator. For every assay, cells were exposed to five increasing concentrations of the reference compounds. tBHQ was diluted in 1:1 titration steps, starting at 100 µM (100, 50, 25, 12.5, and 6.25 µM). The exposure concentrations were identical for both assays (ZF4 and AREc32). TCDD was titrated in half-log steps of different potency ranges in regards to the used assay, giving differences in species-specific sensitivity; ZFL: 30, 10, 3, 1, and 0.3 nM; HepG2: 3, 1, 0.3, 0.1, and 0.03 nM; DRE: 300, 100, 30, 10, 3, 1, 0.3, 0.1 pM. Cells were exposed in triplicate or quadruplicate at each concentration. Each experiment was replicated at least three times.

The exposure medium was spiked with 0.1% (v/v) DMSO as the solvent vehicle, and the highest exposure concentrations across the specific setups were adjusted to yield the same final solvent concentration. A negative control (vehicle control) consisting of six to nine well-replicates was tested on every plate. Among the latter, a triplicate control was performed without solvent and pooled. Non-solvent controls consisted of wells including cells but non-spiked medium. A potential statistically significant difference between the two controls was assessed using two-sided Mann-Whitney U tests (alpha level = 0.05). Pilot studies, the first conducted experiments (see SI1), and wherever plate space was provided (see SI5), also included background controls. Background controls consisted of empty wells to determine background noise levels for the specific lumino- or colourimetric endpoint measurements at the end of each assay. Pilot and historical data from multiple high-throughput screenings did not show statistically significant differences between solvent and non-solvent controls. Background measures were historically stable, comprising approximately 70 raw luminescence units for the luciferase readout and 0.05 raw absorbance units for the MTS readout. The latter comprises 0.01% (luminescence) and 5% (absorbance) of the control values. To save plate space, background controls were omitted, and historical values were subtracted instead. Thus, we could fit all environmental samples, reference compounds, and vehicle controls onto one 96-well plate per assay and per experiment (e.g., raw data in SI1-5 for plate setup). We considered reducing inter-plate variability to be the better trade-off, given the exhaustive historical experience with the previously mentioned controls.

2.3.9 Viability and reporter-related readouts

After 24h exposure, cellular viability and the reporter-related luciferase readouts were both acquired from the same plate by multiplexing a [3-(4,5-dimethyl thiazol-2-yl)-5-(3-carboxymethoxyphenyl)-2-(4-sulfophenyl)-2H-tetrazolium]-based (“MTS”; CellTiter 96® AQueous One Solution Cell Proliferation Assay; Promega, Madison, USA) viability assay with a luciferase or Dual-Luciferase® (“DLR”; Promega, Madison, USA) reporter assay. The MTS assay was conducted according to the manufacturer's protocol. The latter measures the NADPH-dependent turnover of a formazan-salt product in a rapid, colourimetric reaction, thereby indirectly revealing the activity of viable cells. Because the tetrazolium salt is water-soluble, cell lysis is not required. Furthermore, the reagent is non-toxic to cells and enables subsequent readout of the luciferase signal from the same cell population.

2.3.10 MTS-based cellular viability assay

After 24 h of exposure to environmental samples and controls, the exposure medium was discarded, the cells were washed once with 200 µL/well of PBS, and 100 µL/well of Earle's Balanced Salt Solution (EBSS; Gibco, Paisley, UK) was added. For ZFL cells cultured at atmospheric CO_2 levels, 10 mM HEPES was added to the EBSS medium. To start the viability readout, 17% (v/v) MTS reagent was added to

every well. Given their specific cultivation needs, the plates were returned to the incubator for 30 min (mammalian cell lines) or 60 min (fish cell lines). The absorbance of formazan product turnover was measured at 490 nm using a Tecan Spark (Tecan, Männedorf, Switzerland) plate reader.

2.3.11 Luciferase-based reporter readouts

Following the MTS viability readout, cells were washed once in 200 µL/well PBS and lysed in 20 µL/well passive lysis buffer ("PLB"; Promega, Madison, USA) for 30 min on an orbital plate shaker at room temperature. Total raw luminescence units were measured on a Tecan Spark plate reader using a flash luminescence protocol and an auto-injection setup. For stably transfected, single-luciferase reporter assays (AREc32 and DRE), 10 µL/well Fluc substrate (Luciferase Assay System; Promega, Madison, USA) was injected, and the reaction was recorded for 2s. For the DLR reporter assays (ZF4, ZFL), 50 µL/well and (HepG2) 45µL/well Fluc substrate were injected, the reaction was recorded for 2s, quenched by addition of 50/45 µL/well, respectively, Rluc "stop&glow" substrate (Dual-Luciferase®; Promega, Madison, USA), and the secondary signal was recorded for 2s.

2.4 Bioanalytical assessment: the FET assay

Further bioanalytical assessment of the environmental samples has been conducted using a modified version (mFET [19]) of the FET (OECD TG 236 [1]), which also includes sublethal endpoints up to 144 hpf, such as movements per minute, heart rate, and hatching time. In this setup, we also investigated the upstream and downstream recipient samples associated with the respective WWTPs (refer to DIB_A).

2.5 In silico-mediated effect-directed analysis (ISEDA)

2.5.1 tcpl and MySQL R coding packages

The R package ToxCast (TM) Analysis Pipeline (tcpl) [20], in connection with the MySQL ToxCast database interface [21] was utilised in an *in silico*-mediated effect-directed analysis (ISEDA) to discover the primary drivers of toxicity pathway induction for the xenobiotic metabolism MoA with regard to the compounds identified in the targeted chemical analysis.

As a reference, the total AhR CALUX-type [15] concentration-response data ("TOX21_AhR_LUC_Agonist"; human AhR-CALUX clone HG2L7.5c1) were extracted using the following parameters in the command line: assay IDs 543 and 544 (acid), assay acronyms "TOX21_AhR_LUC_Agonist" and "TOX21_AhR_LUC_Agonist_viability" (acnm/aenm), and data IDs 806 and 807 (aeid). This assay was chosen because it is technically closely related to the HepG2 reporter assays used in this study. EC_{20} point estimates were derived from the extracted concentration-response relationships within the constructed database (see SI12). Given the scenario that multiple datasets were available per compound and assay, the geometric mean of the derived

EC_{20}s per compound was computed. Relative effect potencies (REPs) were derived as a ratio of EC_{20} of TCDD to the EC_{20} of the inquired compound.

Additionally, using the extracted cytotoxicity data ("TOX21_AhR_LUC_Agonist_viability"), cytotoxicity thresholds were set either above 50 µM for each compound or at each compound's specific cut-off level. Such compounds were flagged within the database for further analysis. Finally, the total database was compared with the identified compounds from the targeted chemical analysis using the "VLOOKUP" function in MS Excel to retrieve CAS numbers and names. Given that a chemically detected compound could not be found in the extracted ToxCast data, the process was repeated for its respective salt.

For compounds flagged as cytotoxic, the data extraction process was independently repeated to ensure accuracy using the "CLD_CYP1A1_24hr", "CLD_CYP1A2_24hr", "CLD_CYP1A1_48hr", and "CLD_CYP1A2_48hr" command line parameters (see details in SI12). Given the scenario in which EC_{20} estimates below the initial cytotoxicity cut-off could be obtained, these values were forwarded for further analysis.

2.5.2 OCHEM AhR-NR QSAR

All positive hits and cytotoxicity-flagged compounds were forwarded to the OCHEM AhR nuclear receptor QSAR [22], [23], where potential receptor interference was estimated from SMILES inputs. This step aimed to infer potential receptor activation based on SMILES inputs, as depicted in SI12. Compounds flagged as "cytotoxic but active" underwent further testing as pure compounds in the DRE reporter assay, as outlined in section 2.3. Only compounds depicting activity with the DRE assay were forwarded to subsequent analysis.

2.6 Molecular docking: assessing receptor-ligand affinity

2.6.1 Ligands

As employed in the bioanalytical assessment (see section 2.3, above), the reference compounds tBHQ, MZC, TCDD, and the three identified compounds from ISEDA (climbazole, daidzein, thiabendazole) were utilised as *in silico* ligands for molecular docking. Ligand chemical structures and PubChem chemical identifiers (CIDs) are depicted below in Figure S2.

3D structures of all ligands were downloaded from the PubChem database (https://pubchem.ncbi.nlm.nih.gov [24], [25]) and optimised using the Avogadro 1.2.0 software (https://avogadro.cc [26]). Optimisation was conducted as follows. The multiplicity of bonds and hydrogen atoms was checked, and the structures were minimised using the steepest descent algorithm in the "Auto Optimise tool" of Avogadro, utilising the Universal Force Field (UFF)

parameter set. Next, the ligands were uploaded to the RESP ESP charge Derive (RED) Server Development 2.0 [27] to derive the restrained electrostatic potential (RESP) charges. Finally, AutoDock atom types were added, and PDBQT-type files were generated via MGLTools (https://ccsb.scripps.edu/mgltools [28]–[30]). All PDBQT-type files are available in the supplementary folder SI10.

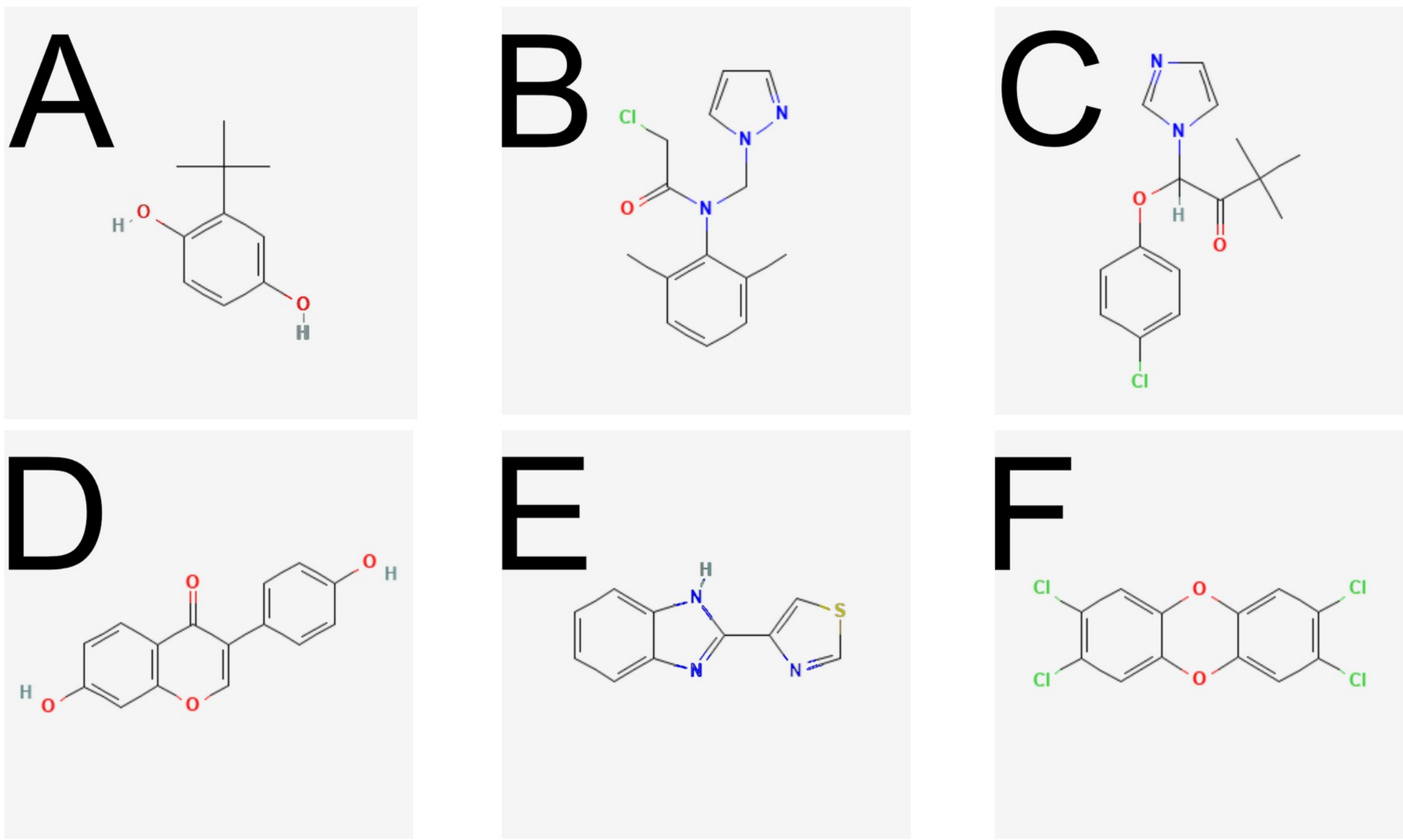

Fig. S2: 2D representation of all selected ligands. Structures were retrieved from the PubChem database and modified. (A) tert-butylhydroquinone (tBHQ, CID: 16043), (B) Metazachlor (MZC, CID: 49384), (C) Climbazole (CLB, CID: 37907), (D) Daidzein (DAI, CID: 5281708), (E) Thiabendazole (TBDZ, CID: 5430), (F) 2,3,7,8-Tetrachlorodibenzodioxin (TCDD, CID: 15625).

2.6.2 Tertiary protein structure selection and prediction: Keap1 and AhR PAS-b domain

As there are no experimental full-sequence protein structures available for Keap1 (fig. S3), the structures were predicted by the AlphaFold2 tool (https://alphafold.ebi.ac.uk [31], [32]) from human and zebrafish Keap1 protein sequences available at UniProt (https://www.uniprot.org [33]; hKeap1: Q14145, zfKeap1a: Q1ECZ2, zfKeap1b: A0A2R8Q1W5). For comparison, two experimental hKeap1 structure fragments were retrieved from the RCSB Protein Data Bank (https://www.rcsb.org [34], [35]). PDB ID 7EXI covers residues 50 to 179 with a resolution of 1.82 Å, and PDB ID 1ZGK covers residues 322 to 609 with a resolution of 1.35 Å.

For AhR (fig. S4), there are no full-sequence experimental structures either. For the human AhR variant (hAhR), a partial cryo-EM structure, PDB ID 7ZUB chain D [36], was selected as the receptor and retrieved from the RCSB Protein Data Bank. This structure covers residues 271 to 427 of hAhR, the PAS-b ligand-binding domain, with a resolution of 2.85 Å. For zebrafish AhR (zfAhR), there are three variants available at UniProt (zfAhR1a: A0A8N7UZF6, zfAhR1b: Q4U3K9, and zfAhR2: Q9YGV3).

Since there are no experimental structures available for these sequences, the PAS-b domains of the three zebrafish AhR variants were modelled with AlphaFold2, corresponding to residues 271 to 385 in zfAhR1a, 281 to 395 in zfAhR1 b, and 289 to 403 in zfAhR2.

The predicted full protein structures of Keap1 were retrieved from the AlphaFold database (https://alphafold.ebi.ac.uk [32]) via UniProt, the PAS-b domains of zfAhR variants were predicted using AlphaFold2 in ColabFold (https://colab.research.google.com/github/sokrypton/ColabFold [37]), and the X-ray and cryo-EM structures were downloaded from RCSB PDB. All zebrafish structures were aligned to the predicted full-length human sequence structure in PyMOL (PyMOL Molecular Graphics System, v. 1.8.4.) and stripped of all HETATM records (non-protein atoms). All positions mentioned throughout the project correspond to the hKeap1 and hAhR amino acid sequences (see figs. 6, 7, and 13 in DIB_B). The structure PDB ID 1ZGK contains seven modified selenomethionine residues, which were converted to methionine residues by replacing the selenium atoms with sulphur. The structures were protonated using the H++ web server v. 4.0 (http://newbiophysics.cs.vt.edu/H++/ [38], [39]) with pH = 7.0, salinity = 0.1 M, internal dielectric = 10, and external dielectric = 80. AutoDock atom types and Gasteiger charges were added to the receptors by MGLTools, and the corresponding PDBQT files were generated (supplementary folder SI10). All receptors were prepared without any ligands.

2.6.3 Amino acid sequence alignment

FASTA files of the human and zebrafish sequences named in section 2.6.2 above were acquired from the National Centre for Biotechnology Information's (NCBI) webpage (https://www.ncbi.nlm.nih.gov/ [25]) and imported into the Unipro Ugene alignment software (https://ugene.net/ [40]). All FASTA-type files used are provided in SI10. AA multiple sequence alignment was conducted via the ClustalW algorithm, utilising the following parameters: gap open penalty 10, gap extension penalty 0.2, weight matrix BLOSUM, gap separation distance 4, weight transition no, hydrophilic gaps yes. Sequence alignment and distance matrices were computed in Unipro Ugene, exported, and graphically aligned in Affinity Designer (Serif, Nottingham, UK). Simplified, linear domain maps were designed in IBS Illustrator (https://ibs.biocuckoo.org/ [41]).

2.6.4 Topology, mapping, and nomenclature

This section gives 3D structures and domain maps for Keap1 (Fig. S3) and AhR (Fig. S4) to facilitate orientation. Section 4.1 below provides a summary of the Nrf2-Keap1-ARE pathway for the interested reader, in relation to the docking modelling conducted. We refer to relevant literature for more details about the AhR-ARNT-XRE pathway (e.g., [5]).

Notably, humans have only one receptor/sensor variant, whereas fish have multiple ones due to the teleost genome duplication event [42].

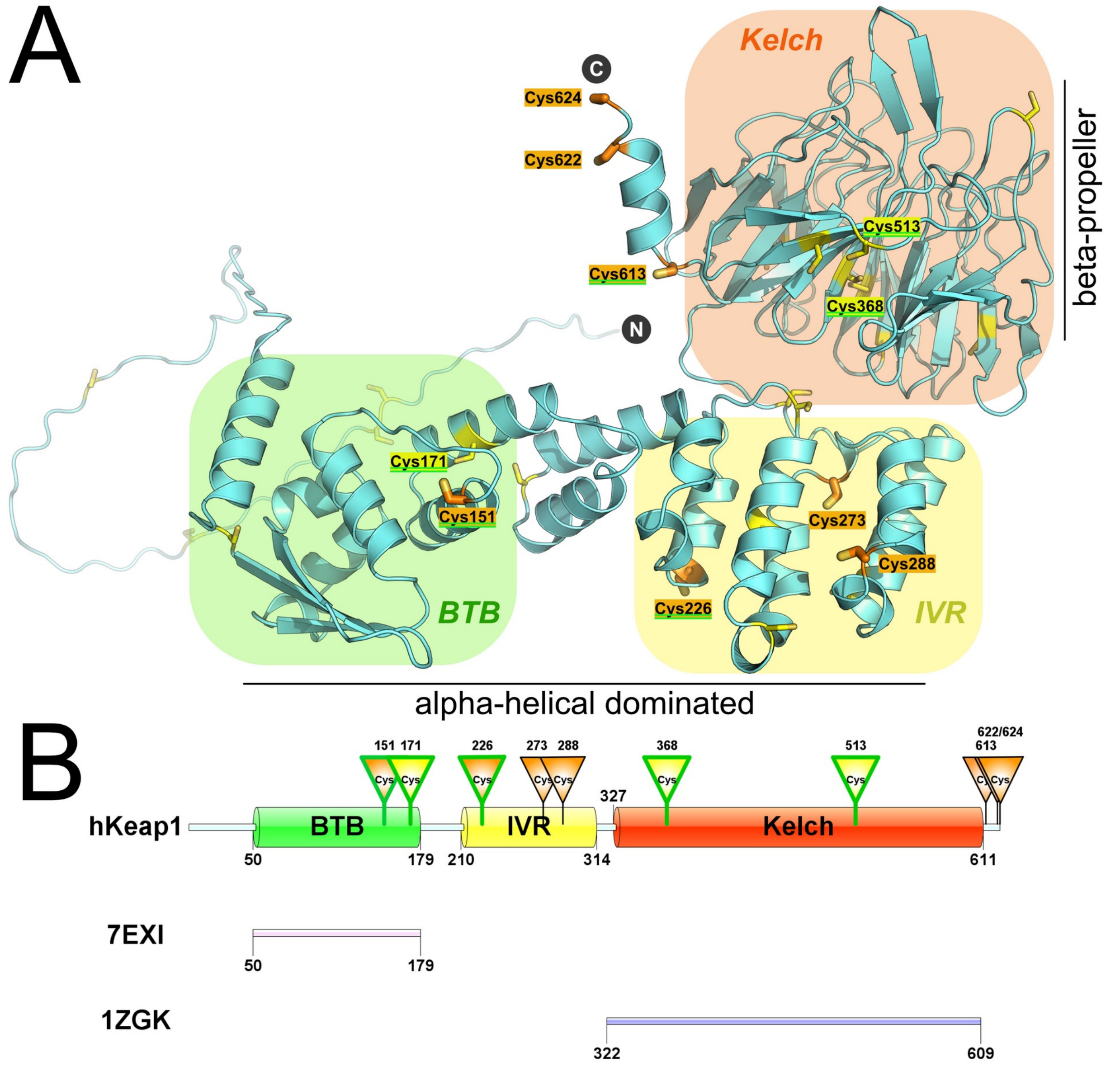

Fig. S3: Topology of hKeap1. The 3D structure is shown as a cyan cartoon (panel A). A simplified sequence structure is given below (panel B), to which partial crystal structures 7EXI and 1ZGK were aligned. Canonical reactive cysteines are highlighted in orange, and other cysteines are highlighted in yellow. Cysteines identified within this study are marked in green. Domains are highlighted as rectangles concerning their region. Alpha-helical dominated region: Broad complex, tram-track, and Bric a brac domain (BTB, residues 50 to 179, green) and the intervening region (IVR, residues 210 to 314, yellow). Beta-propeller region: Kelch domain (residues 327 to 611, orange). The N- and C-termini are illustrated as black-and-white dots labelled N and C, respectively. Illustrations were created in PyMOL and the IBS Illustrator.

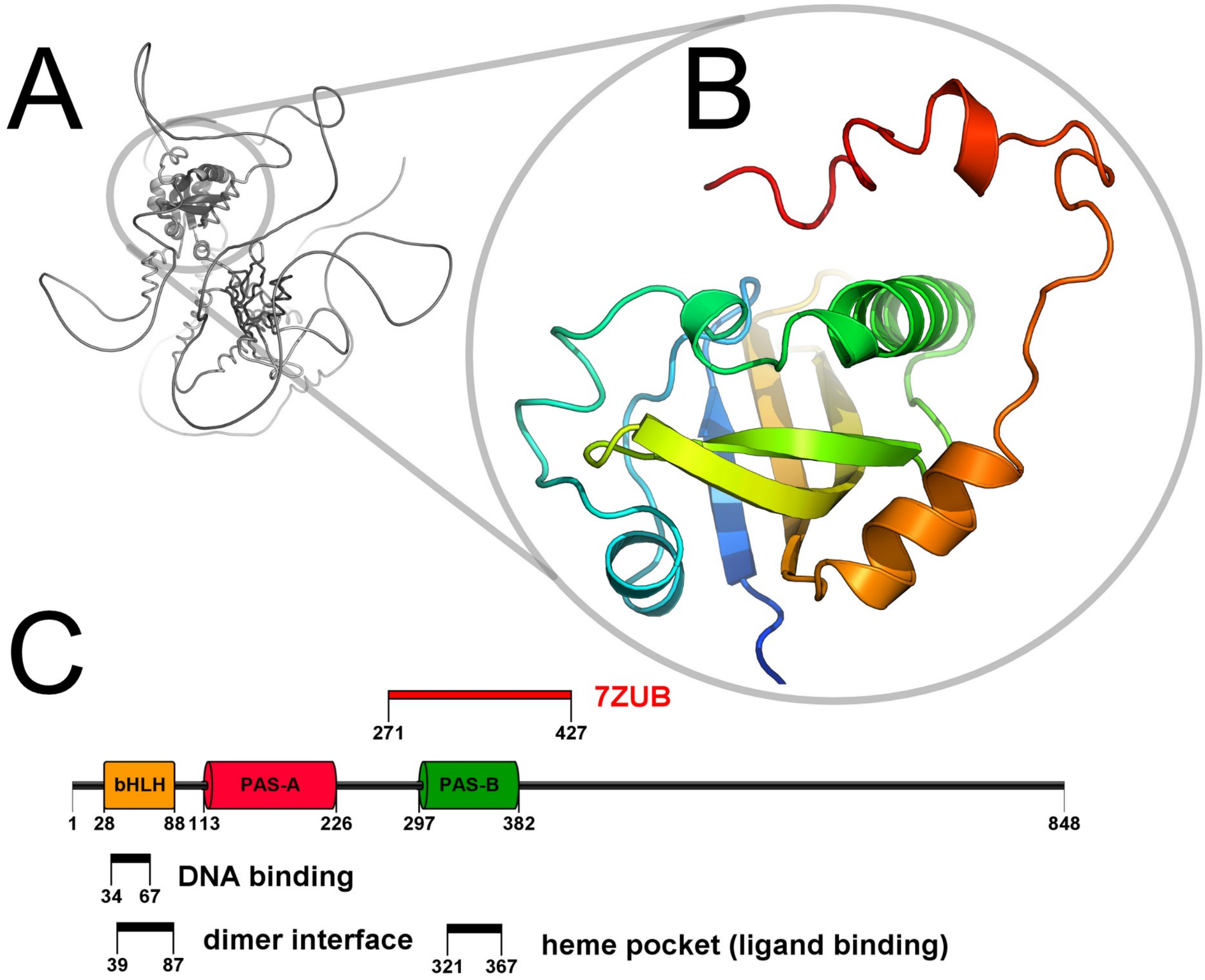

Fig. S4: Topology of hAhR. (A) The 3D structure of the entire hAhR, as predicted by AlphaFold, is illustrated in grey. (B) Zoom in on the PAS-B ligand binding domain of hAHR as a rainbow-coloured cartoon. (C) A simplified sequence structure is given in the lower left corner, to which the partial cryo-EM structure 7ZUB is aligned. Domains are illustrated as rectangles and cylinders with regard to their location within the AA sequence: basic helix-loop-helix (bHLH, residues 28 to 58), PAS-A (Per-Arnt-Sim-A, residues 113 to 226), and PAS-B (Per-Arnt-Sim-B, residues 297 to 382). The respective protein features are given below the simplified sequence. Illustrations were created in PyMOL and the IBS Illustrator.

2.6.5 Molecular docking procedure

The AutoDock Vina 1.1.2 algorithm [43] was utilised for molecular docking. As Keap1 is a large protein with distinct domains, two overlapping grid boxes covering the whole protein were set up in PyMOL (Schrödinger Inc., New York, US) using the Autodock/Vina plugin [44]. The first docking grid dimensions were x = 65 Å, y = 68 Å, z = 72 Å, with the center at x = −10.74, y = −32.27, z = −7.88 (Box1). The second docking grid was 53 x 102 x 61 Å in size, with the centre at x = −24.74, y = −49.27, z = −57.88 (Box2). Regarding site-directed docking to the reactive Cys151 residue, a 15 × 15 × 15 Å box was used, centred at x = −19, y = −35, and z = −57 (Box3). For AhR, site-directed docking to the PAS-b domain was set up by placing a docking grid to cover the binding site, with dimensions 18 x 18

x 18 Å and centre coordinates at x = 13, y = 6, and z = −24. The flag "exhaustiveness = 100" was used to thoroughly sample the possible conformational space. The number of output conformations of the docked ligand was set to 10 poses. Finally, PyMOL was used to visualise the predicted poses, measure distances, and render pictures. The raw data are presented in section 3 of the DIB_B article and in SI9. Additional box plots of binding energies were created in GraphPad Prism 9 (GraphPad Software, Boston, Massachusetts, USA).

2.7 Quantification of bioavailable concentrations via mass-balance modelling

Bioavailable (or free cellular concentrations, C_{free}) of the utilised reference compounds and the three compounds identified via ISEDA were computed via the UFZ-LSER QSPR [45], employing a method by Fischer et al. [46] that is based on several mass-balance-equation models [47], [48]. For experimental and calculated descriptors, mass-balance equations, biomaterials, model output, summary, and further references, please consult SI13.

The following input parameters were chosen: I) Concentration of analyte, 3 µM for tBHQ and MZC, 30 nM for TCDD and the compounds identified via ISEDA (additionally, AC_{50} point estimates of the ISEDA-identified compounds, as derived via log-logistic regression (see the following section 2.8.1), were transformed into free cellular concentrations, as well). II) Cell culture medium volume: 100 µL. III) Type of base medium: DMEM. IV) Fraction of base medium: 95% for ZFL and DRE, 90% for ZF4, MCF7AREc32, and HepG2. V) Type of serum: FBS. VI) Fraction of serum: 5% for ZFL and DRE, 10% for ZF4, MCF7AREc32, and HepG2 cell lines. VII) Cell number: according to concentrations named in section 2.3.6 above, in 100 µL.

The C_{free} geometric mean was calculated and forwarded for further analysis in the scenario where we obtained multiple free cellular concentration predictions from various mass-balance equations, descriptors, and database queries.

2.8 Data processing and statistical analyses

2.8.1 Reporter gene assays – raw data handling and normalisation

For the initial raw data handling and normalisation procedures, please consult SI1 to SI8, SI14, and SI15. To summarise, in the first step, raw luminescence units from the single-luciferase reporter assay (LR) were used for analysis, as measured by the AREc32 and DRE assays. Similarly, raw absorbance units recorded in the MTS viability assay were not further normalised in the first step. DLR raw luminescence units were normalised to cell number and transfection efficiency by dividing the primary Firefly luciferase (Fluc) signal by the constitutive Renilla luciferase (Rluc) signal for every technical replicate. In the second step, technical replicates within each exposure group were pooled

to obtain experimental means, which were then forwarded to subsequent data transformation and analysis procedures, as described below.

2.8.2 Reporter gene assays – regression analyses

Data-fitting techniques for simple linear and nonlinear regression were conducted as described by Escher et al. [49], [50], with modifications by Weimer et al [51]. Alternative data-fitting procedures are discussed further below in the SM sections 3.3 and 3.4, along with their respective supplementary results.

For the main article, values obtained from the oxidative stress MoA bioassays (ZF4 and MCF7/AREc32) were expressed as induction ratios (IR) in relation to the negative controls. Both environmental sample and reference compound values were fitted to simple linear regressions, anchored at the origin (f(x)=1). $ECIR_{1.5}$ point estimates for calculating BEQs were derived from the line fit.

Values obtained from the xenobiotic MoA bioassays (ZFL, HepG2, and DRE) were expressed as a percentage of maximum TCDD induction (%min.max.). Data obtained from the environmental samples were fitted to a simple linear regression, anchored at the origin (f(x)=0). In contrast, TCDD-reference and ISEDA-compound concentration-response curves were fitted using Hill or log-logistic regression with different parameterisations. Alternative parametrisation and curve fit are further discussed in sections 3.3 and 3.4. The 3PNL model was applied throughout the main article, as it aligns with the guidance provided by Escher et al [50]. EC_{10} values for BEQ calculations were derived from the line and curve fits, respectively.

To compare general effect differences between reporter lines within the same exposure setup, the above-stated values were log10- or Box-Cox-transformed and subjected to a 2-way ANOVA with “reporter line” and “exposure concentration” as factors. For tests involving three different reporter lines (xenobiotic metabolism MoA, all influent samples), the 2-way ANOVA was followed by Tukey's multiple comparison test. Normality of residuals via Shapiro-Wilk and Kolmogorov-Smirnov tests, homoscedasticity via Spearman's rank correlation test, and optical inspection of residuals via QQ-plot and residual plot (predicted vs. fitted residuals) were conducted to ensure that ANOVA criteria were met. A $p<0.05$ was considered statistically significant, while a $p<0.1$ indicated a trend. All calculations in the main article were performed using GraphPad Prism 9 (GraphPad Software, Boston, USA).

2.8.3 Iceberg modelling

Iceberg modelling was conducted as described by Neale et al. [52]–[54]. Relative effect potencies (REPs) of the identified compounds within the targeted chemical analysis were calculated by dividing the EC_{20} of TCDD by the geometric mean point estimates (EC_{20}) derived from the ISEDA, as obtained

from ToxCast AhR CALUX-type bioassay (TOX21_AhR_LUC_Agonist; human AhR-CALUX clone HG2L7.5c1) data (see section 2.5).

$$REP = \frac{EC_{20;RC}}{\prod_{i=1}^{n} EC_{20;i}}$$

With RC: reference compound (here TCDD), i: compound identified in ISEDA.

Chemical equivalents (BEQ_{chem}) were calculated by summing up the respective positive ISEDA hits for every WWTP sample (see SI12).

$$BEQ_{Chem} = \sum_{i=1}^{n} \frac{REP_i * c_i * MW_{RC}}{MW_i}$$

With RC: reference compound (here TCDD), i: compound identified by chemical analysis, MW: molecular weight, c: concentration, REP: relative effect potency.

Biological equivalents (BEQ_{bio}) were calculated by dividing the computed effect point estimates of the reference compound ($EC_{IR1.5}$ for tBHQ and MZC, EC_{10} for TCDD) by the point estimates derived from the WWTP sample regression fitting (see SI16).

$$BEQ_{Bio;ox.stress} = \frac{EC_{IR1.5;RC}}{EC_{IR1.5;i}}$$

$$BEQ_{Bio;xen.met.} = \frac{EC_{10;RC}}{EC_{10;i}}$$

With RC: reference compound (here tBHQ, MZC, and TCDD), i: environmental sample.

Percentage contribution of BEQchem to BEQbio:

$$[\%] = \frac{BEQ_{Chem}}{BEQ_{Bio}} * 100$$

2.8.4 Removal efficiencies

Removal efficiency (%) from chemical analyses:

$$[\%] = \frac{\sum_{i=1}^{n;influent} c_i - \sum_{i=1}^{n;effluent} c_i}{\sum_{i=1}^{n;influent} c_i} * 100$$

Removal efficiency (%) from bioanalytical assessment:

$$[\%] = \frac{BEQ_{inf} - BEQ_{eff}}{BEQ_{inf}} * 100$$

2.8.5 Statistical analysis of molecular docking-derived data

To compare differences between receptor/sensor binding energies for all investigated zebrafish and human variants, up to ten binding poses (where possible) per binding site were subjected to non-parametric Kruskal-Wallis tests, followed by Dunn's multiple comparison test. A $p<0.05$ was regarded as statistically significant. All calculations were conducted in GraphPad Prism 9.

2.8.6 Ratios

To compare regression fit estimates, BEQ values, and bioavailable concentrations for species sensitivity, the zebrafish estimates were divided by the corresponding human or mouse estimates.

3. Supplementary results

3.1 Supplementary results: DIB_B co-submission

More detailed and zoomed-in docking poses from blind and site-directed docking to the investigated ligands

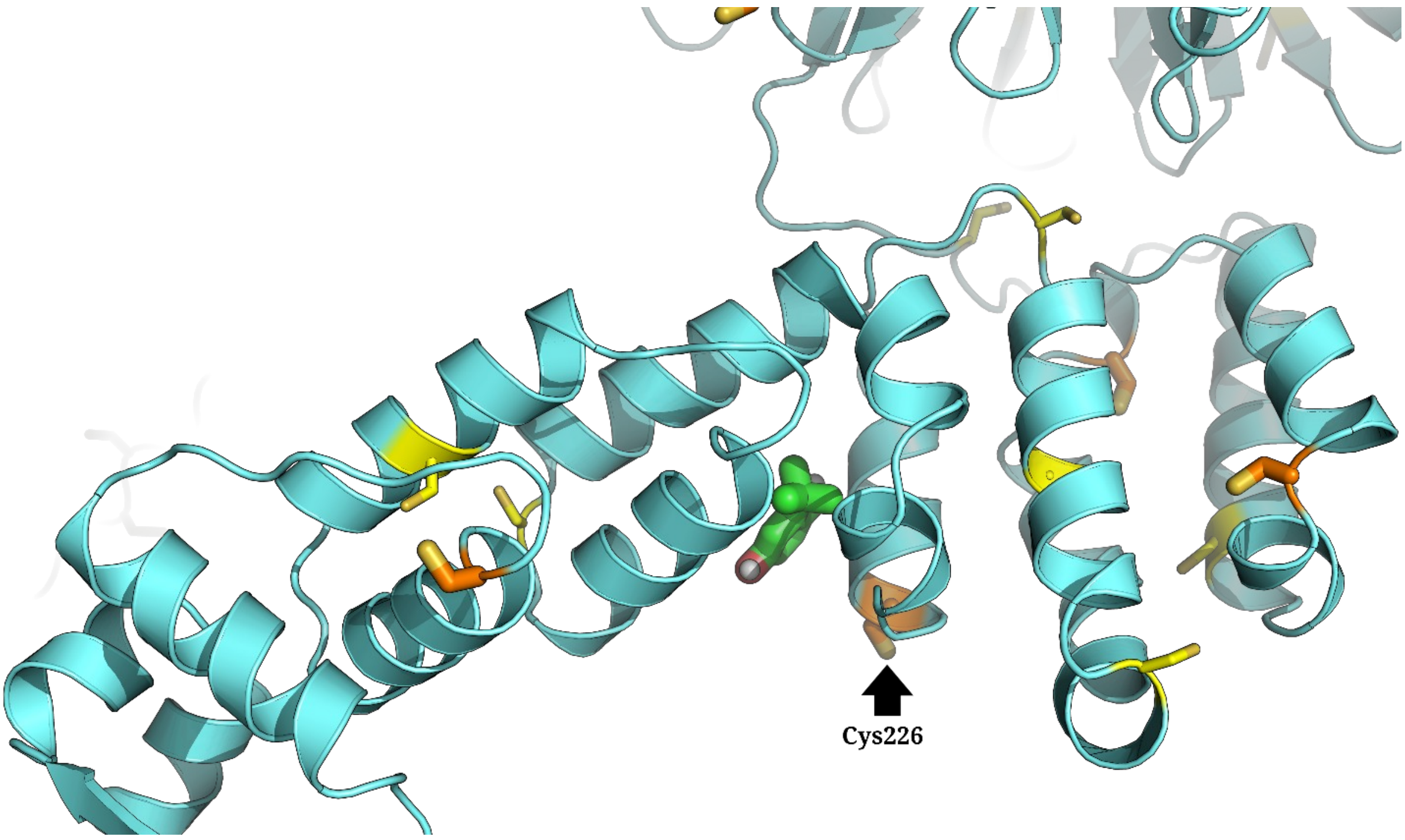

Fig. S5: Molecular docking (tBHQ). Best energy pose (site A, -6.6 kcal/mol) of tBHQ (green ligand) from blind docking to a predicted full-sequence structure of hKeap1 (cyan cartoon). Here, the alpha-helical region (IVR domain) is emphasised. Canonical cysteine residues 151, 226, 273, and 288 are highlighted as orange sticks; the remaining ones are in yellow.

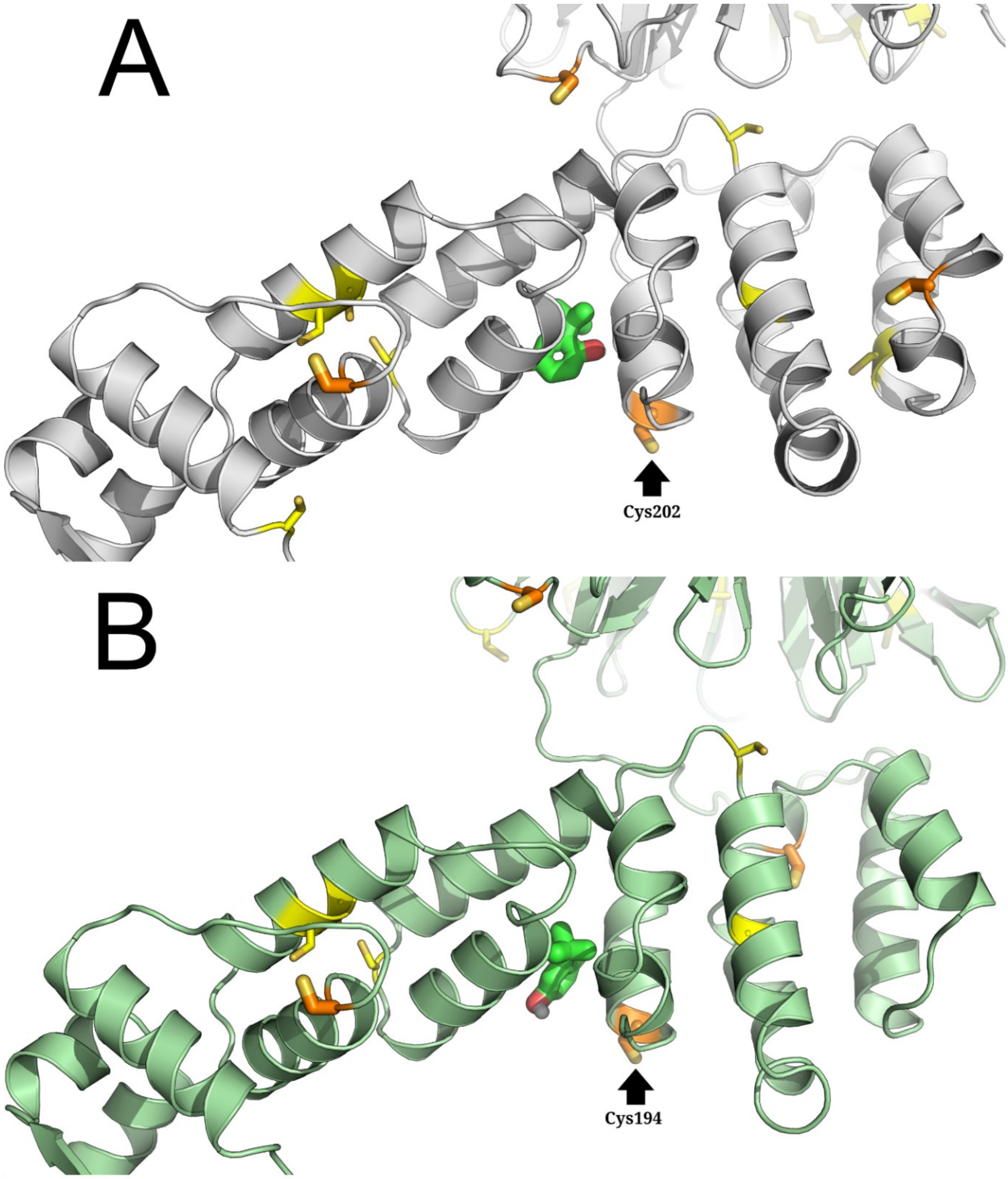

Fig. S6: Molecular docking (tBHQ). Best binding pose (site A, -6.3 and -6 kcal/mol, respectively) of tBHQ (green ligands) in the alpha-helical region (IVR domain) of predicted structures of zfKeap1a (light grey cartoon, A) and zfKeap1b (light green cartoon, B). Canonical cysteine residues are shown as orange sticks, and the remaining cysteine residues are shown as yellow lines. The enumerated zebrafish cysteines correspond to Cys226 in hKeap1.

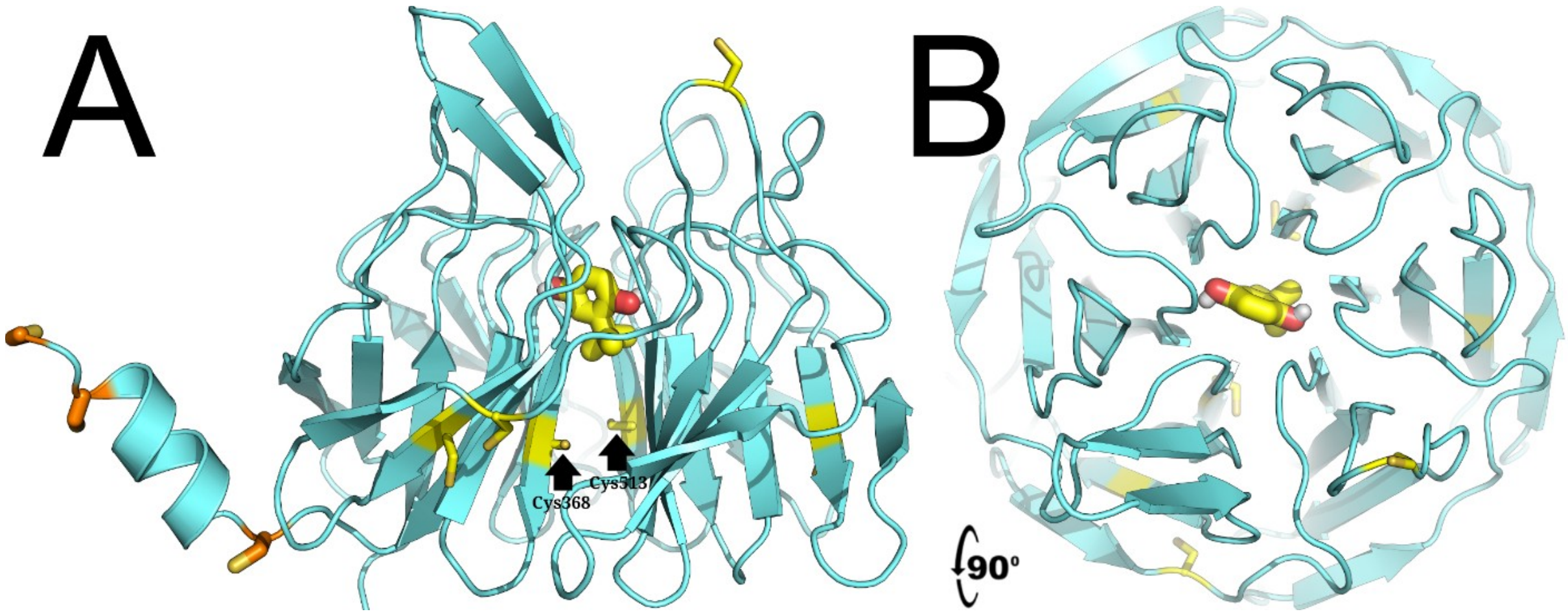

Fig. S7: Molecular docking (tBHQ). Two different views of the second-best pose (site B, −6.2 kcal/mol) of tBHQ (yellow ligand) situated in the central cavity of the Kelch domain from blind docking to the AlphaFold-predicted structure of hKeap1 (cyan cartoon). Canonical cysteine residues are highlighted as orange sticks branching from the protein's backbone, while the remaining cysteines are in yellow.

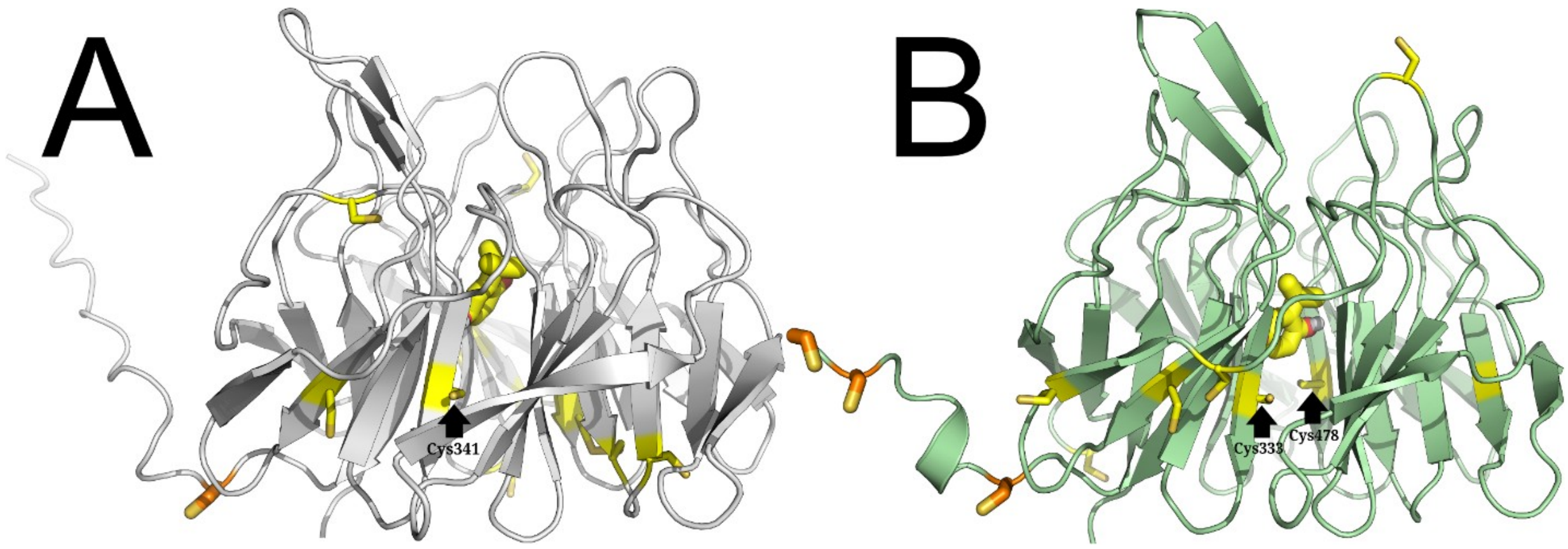

Fig. S8: Molecular docking (tBHQ). The poses of tBHQ (yellow ligands) inside the Kelch domain (site B) of zfKeap1a (light grey cartoon, −6 kcal/mol, A) and zfKeap1b (light green cartoon, −6.1 kcal/mol, B). Canonical cysteine residues are highlighted as orange sticks branching from the protein's backbone, while the remaining cysteines are in yellow. The enumerated zebrafish cysteines correspond to Cys368 and 513 in hKeap1.

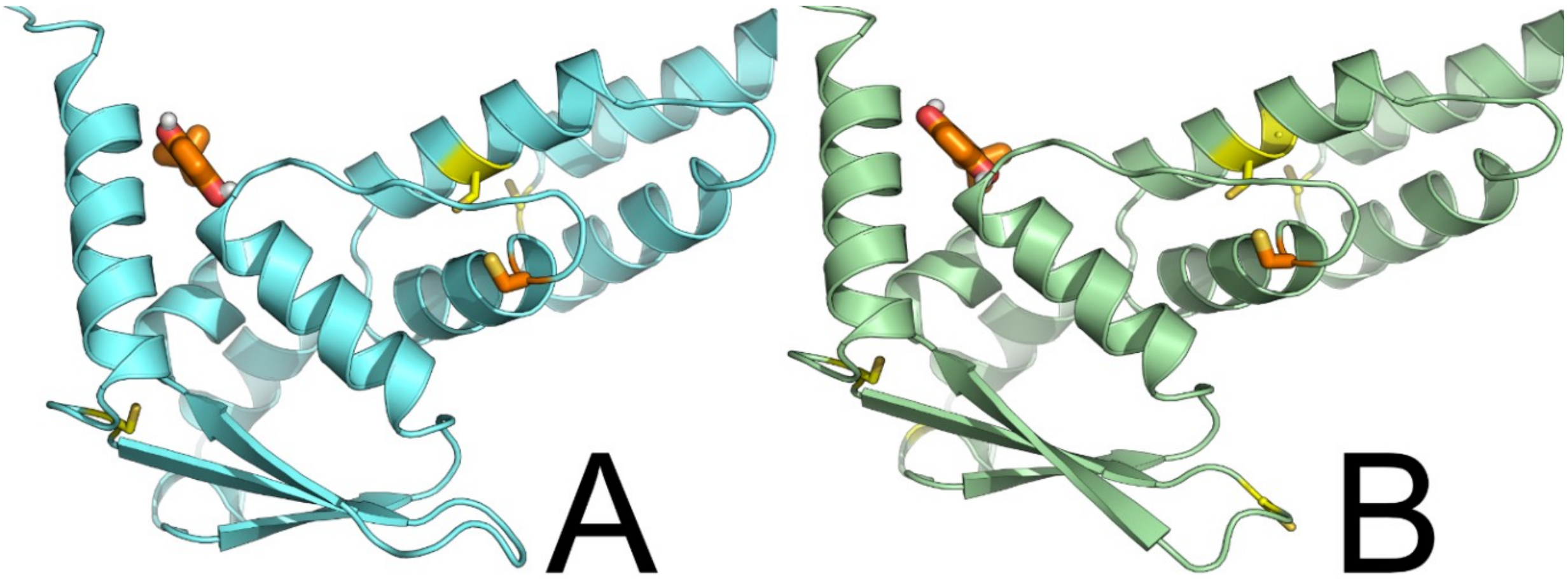

Fig. S9: Molecular docking (tBHQ). tBHQ (orange ligands) in the third-best binding site (site C) situated in the BTB domain from blind docking to predicted structures of hKeap1 (cyan cartoon, -5.5 kcal/mol, A) and zfKeap1b (light green cartoon, −5.3 kcal/mol, B). Canonical cysteine residues are shown as orange sticks, the remaining ones as yellow lines.

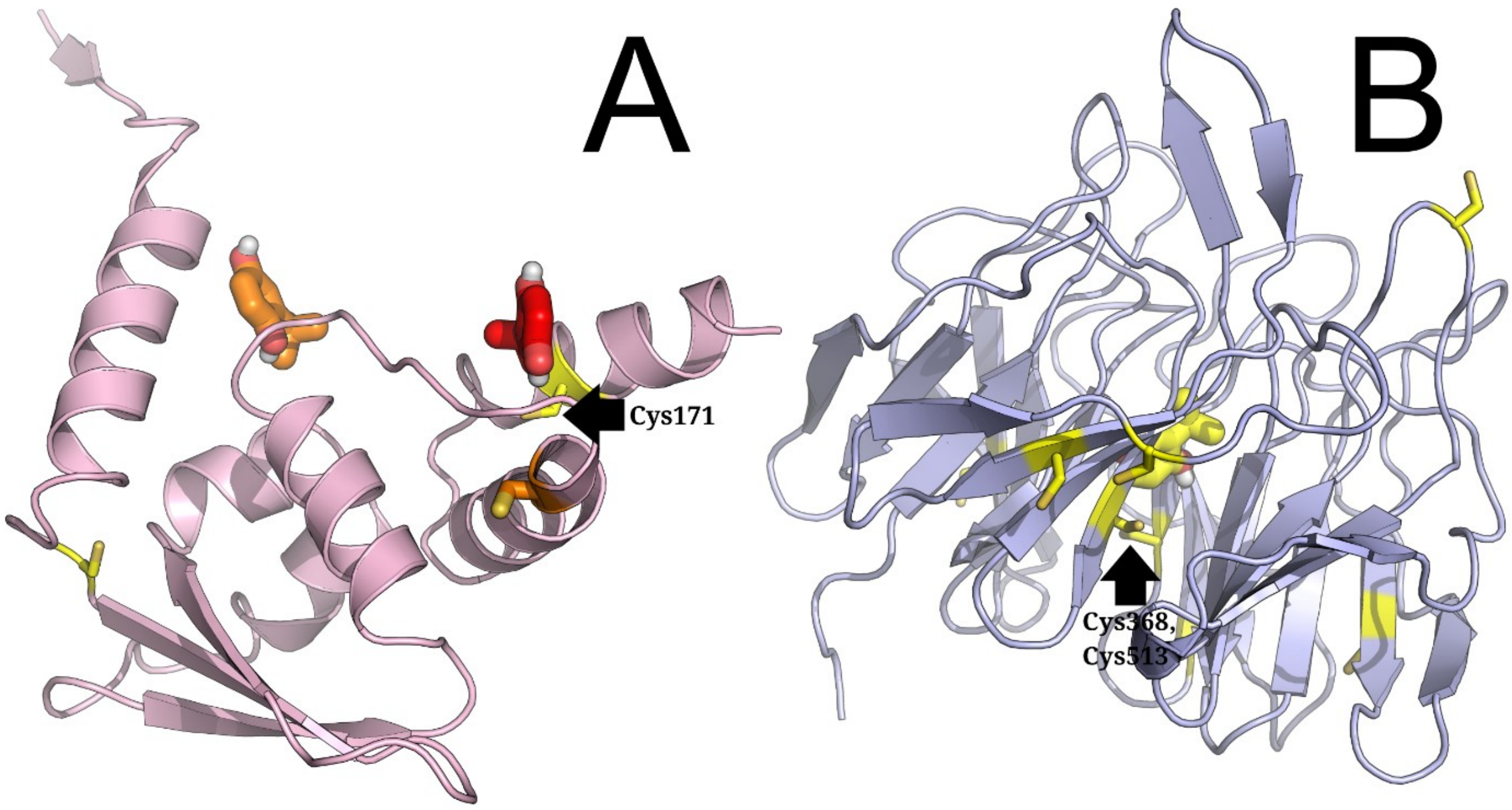

Fig. S10: Molecular docking (tBHQ). Best energy poses of tBHQ (coloured ligands) per site from blind docking to X-ray structures PDB ID 7EXI (light pink cartoon, A) and 1ZGK (light purple cartoon, B). The ligand poses are colour-coded, with yellow corresponding to the best predicted binding energy (site B, -6.3 kcal/mol) and red to the worst (site E, -5.1 kcal/mol). Canonical cysteine residues are highlighted as orange sticks branching from the protein's backbone, while the remaining cysteines are in yellow. All illustrative data is summarised quantitatively in tabs. 3 and 4 (DIB_B article).

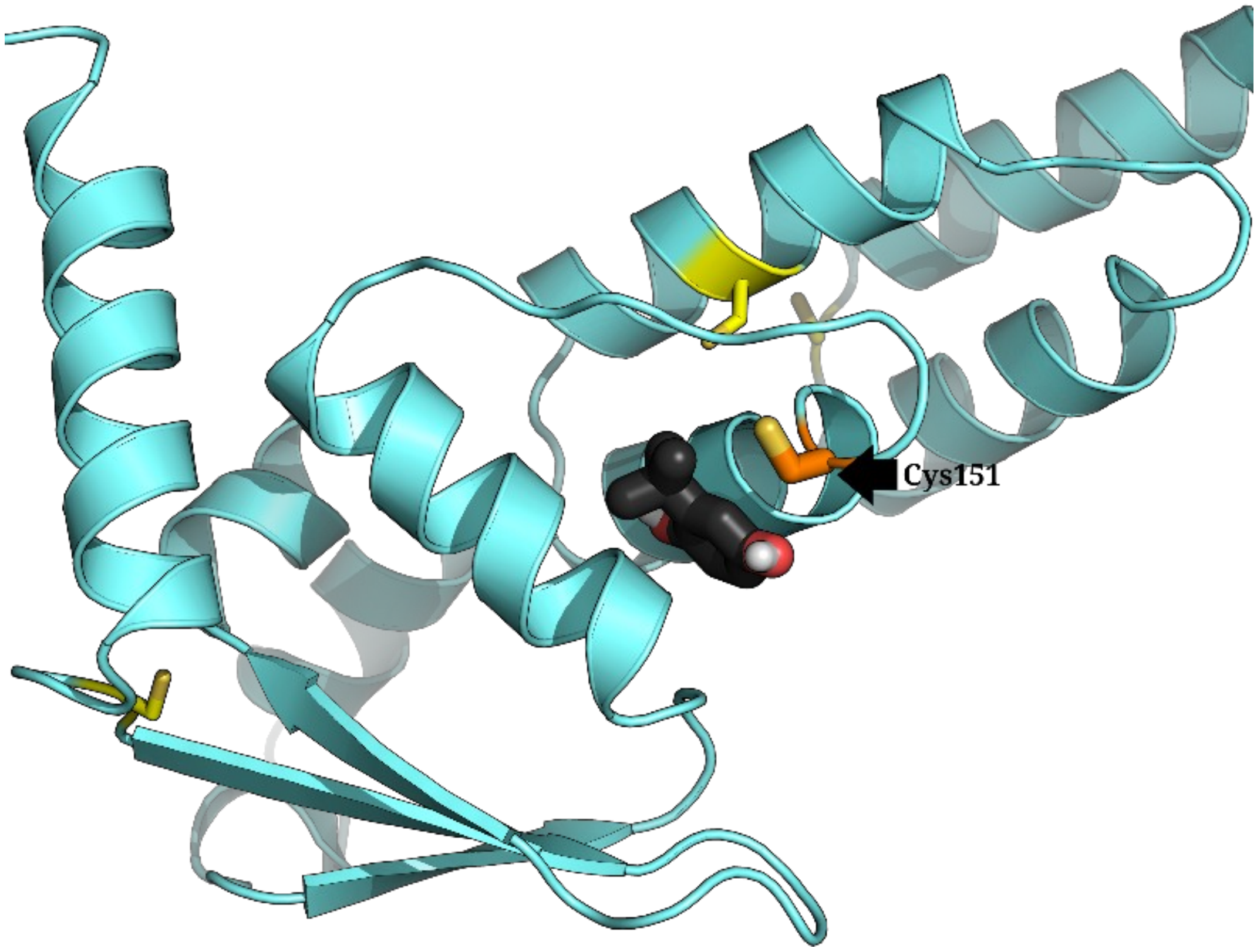

Fig. S11: Molecular docking (tBHQ). The best-energy pose for tBHQ (site D, black ligand, -4.6 kcal/mol) from site-directed docking to the proximity of reactive residue Cys151 in hKeap1 (cyan cartoon). Canonical cysteine 151 is shown as orange sticks, while the remaining cysteines are in yellow. All illustrative data is summarised quantitatively in tabs. 3 and 4 (DIB_B article).

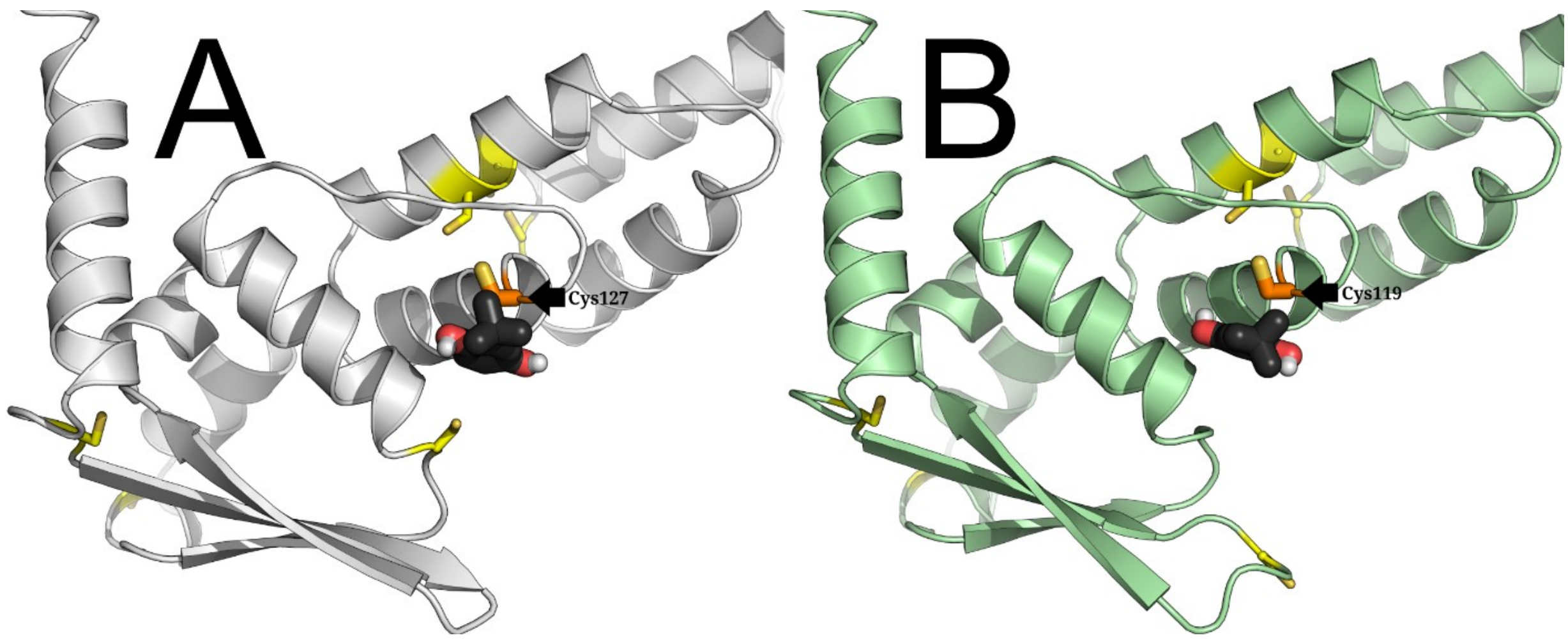

Fig. S12: Molecular docking (tBHQ). The best-energy poses of tBHQ (site D, black ligands) from site-directed docking to the proximity of the canonical Cys151 residue of AlphaFold-predicted structures of zfKeap1a (light grey cartoon, -3.5 kcal/mol, A) and zfKeap1b (light green cartoon, -3.9 kcal/mol, B). Reactive cysteines 127 and 119 (corresponding to Cys151 in hKeap1) are shown as orange sticks, while the remaining cysteines are yellow. All illustrative data is summarised quantitatively in tabs. 3 and 4 (DIB_B article).

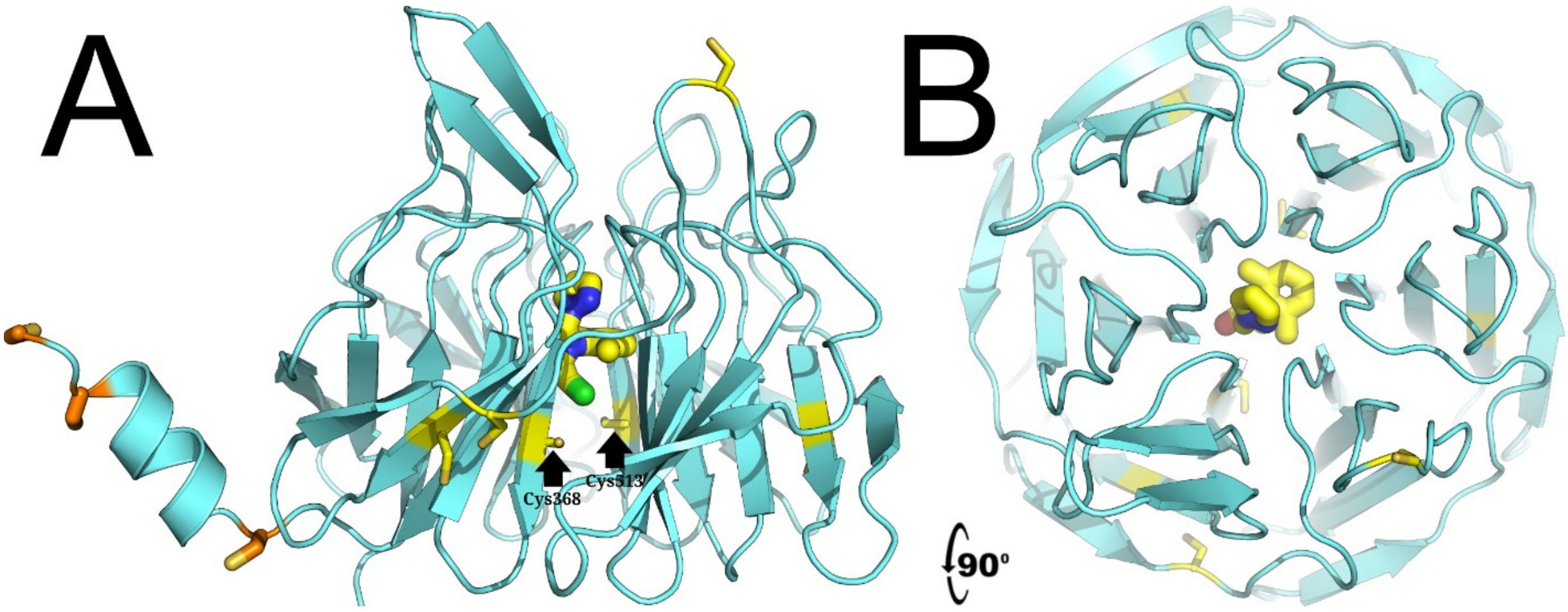

Fig. S13: Molecular docking (MZC). Two different views of the best-energy pose (site A, -6.6 kcal/mol) of MZC (yellow ligands) situated in the central cavity of the Kelch domain of hKeap1 from blind docking to AlphaFold-predicted structure (cyan cartoon). Canonical cysteine residues are highlighted as orange sticks branching from the protein's backbone, while the remaining cysteines are in yellow.

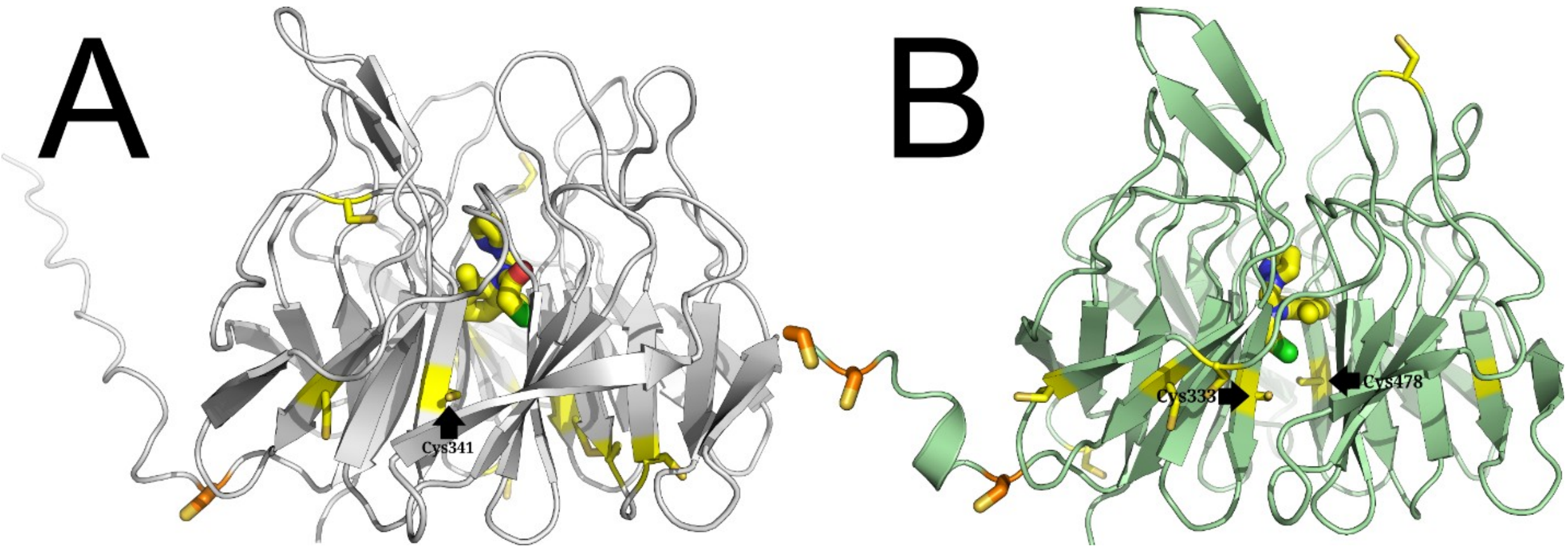

Fig. S14: Molecular docking (MZC). Best-energy poses (site A) of MZC (yellow ligands) inside the Kelch domain of zfKeap1a (light grey cartoon, −6.9 kcal/mol, A) and zfKeap1B (light green cartoon, −6.8 kcal/mol, B). Canonical cysteine residues are highlighted as orange sticks branching from the protein's backbone, while the remaining cysteines are in yellow. Enumerated cysteine residues 333, 341, and 478 correspond to Cys 368 and 513 in hKeap1.

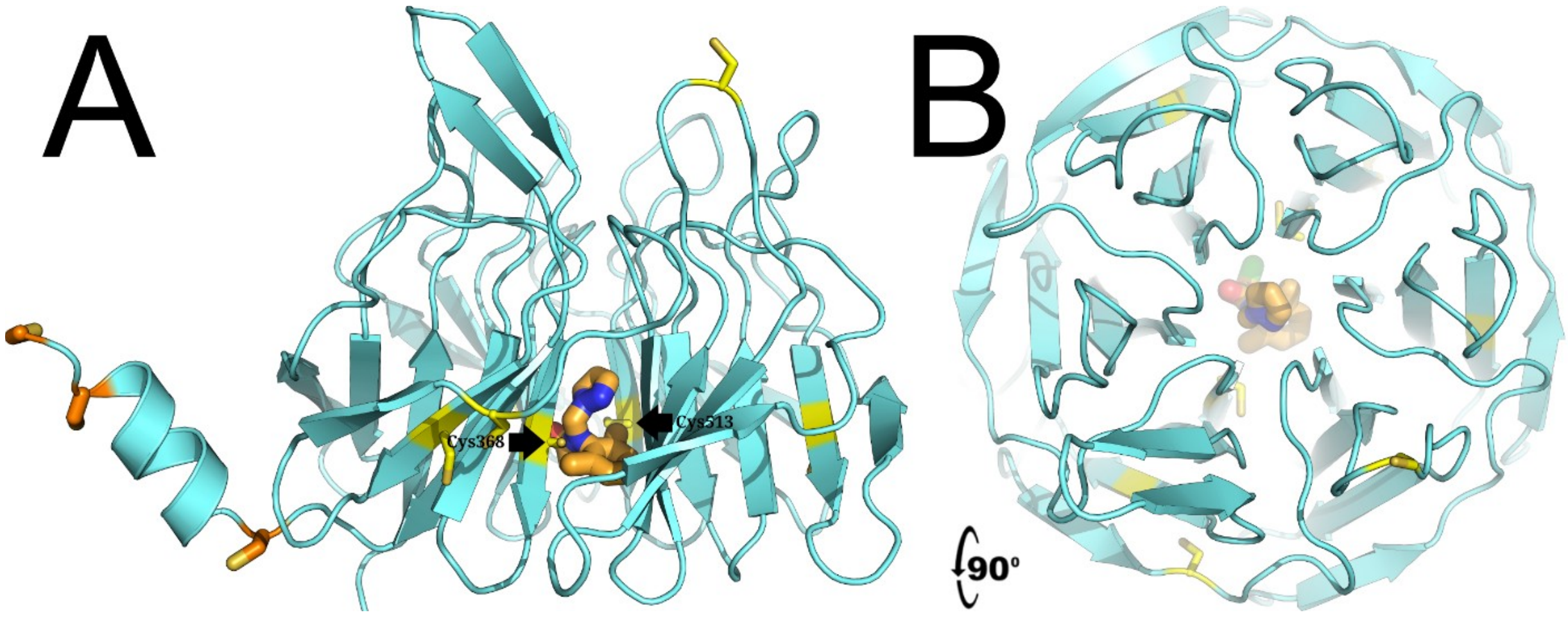

Fig. S15: Molecular docking (MZC). Two different views of the second best-energy site (site B, -6.5 kcal/mol), including a pose of MZC (light orange ligand) situated in the central cavity of the Kelch domain of hKeap1 from blind docking to the AlphaFold-predicted structure (cyan cartoon). Canonical cysteine residues are highlighted as orange sticks branching from the protein's backbone, while the remaining cysteines are in yellow.

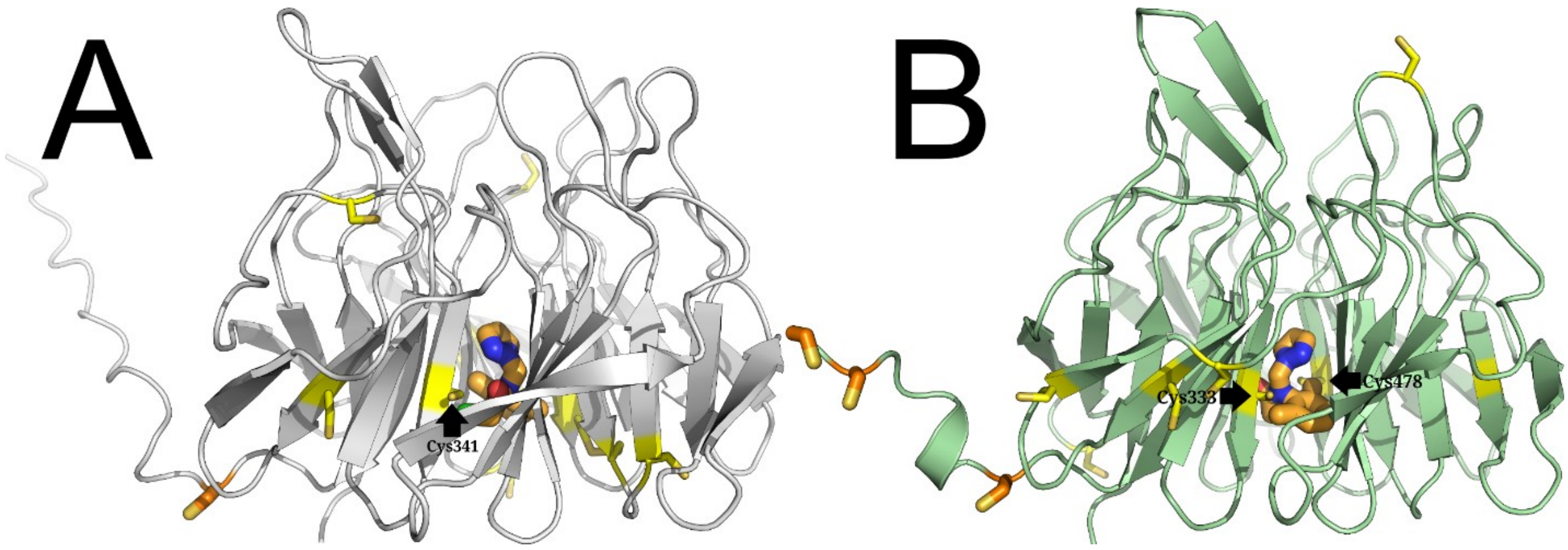

Fig. S16: Molecular docking (MZC). Best-energy poses (for site B) of MZC (light orange ligands) inside the Kelch domain of zfKeap1a (light grey cartoon, −6.4 kcal/mol, A) and zfKeap1b (light green cartoon, −6.5 kcal/mol, B). Canonical cysteine residues are highlighted as orange sticks branching from the protein's backbone, while the remaining cysteines are in yellow. Enumerated cysteine residues 333, 341, and 478 correspond to Cys368 and 513 in hKeap1.

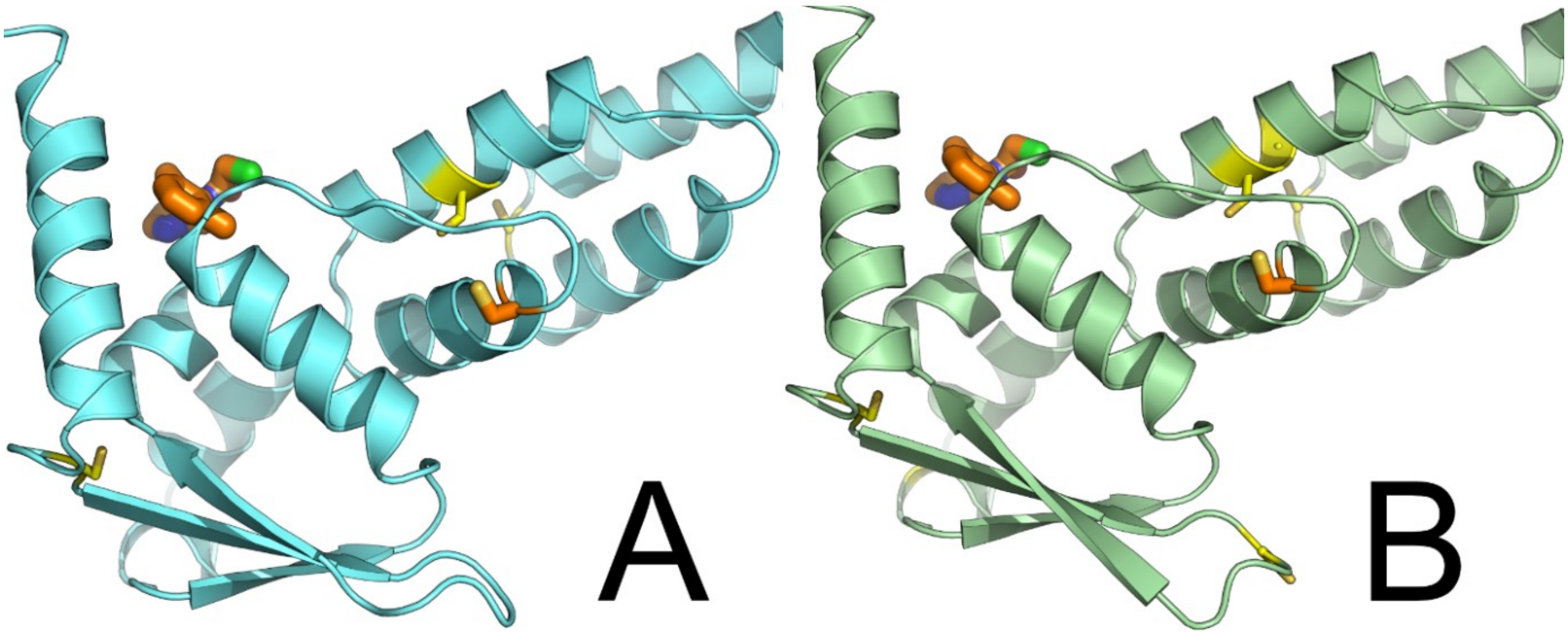

Fig. S17: Molecular docking (MZC). MZC poses (orange ligands) in the third best binding site situated within the BTB domain (site C) of hKeap1 (cyan cartoon, -5 kcal/mol, A) and zfKeap1b (light green cartoon, -5.1 kcal/mol, B) predicted structures. Canonical cysteine residues are shown as orange sticks, the remaining ones as yellow lines.

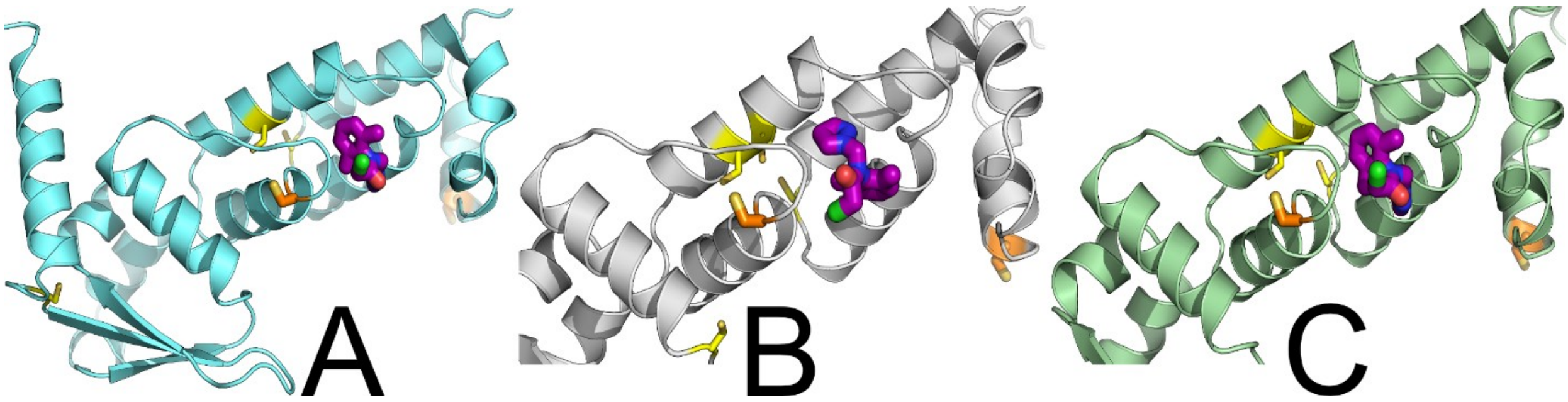

Fig. S18: Molecular docking (MZC). MZC poses (purple ligands) in the fourth best binding site of the alpha-helical region (site D) of hKeap1 (cyan cartoon, -5 kcal/mol, A), zfKeap1a (light grey cartoon, -5.6 kcal/mol, B), and zfKeap1b (light green cartoon, -5.1 kcal/mol, C) predicted structures. Canonical cysteine residues are shown as orange sticks, the remaining ones as yellow lines.

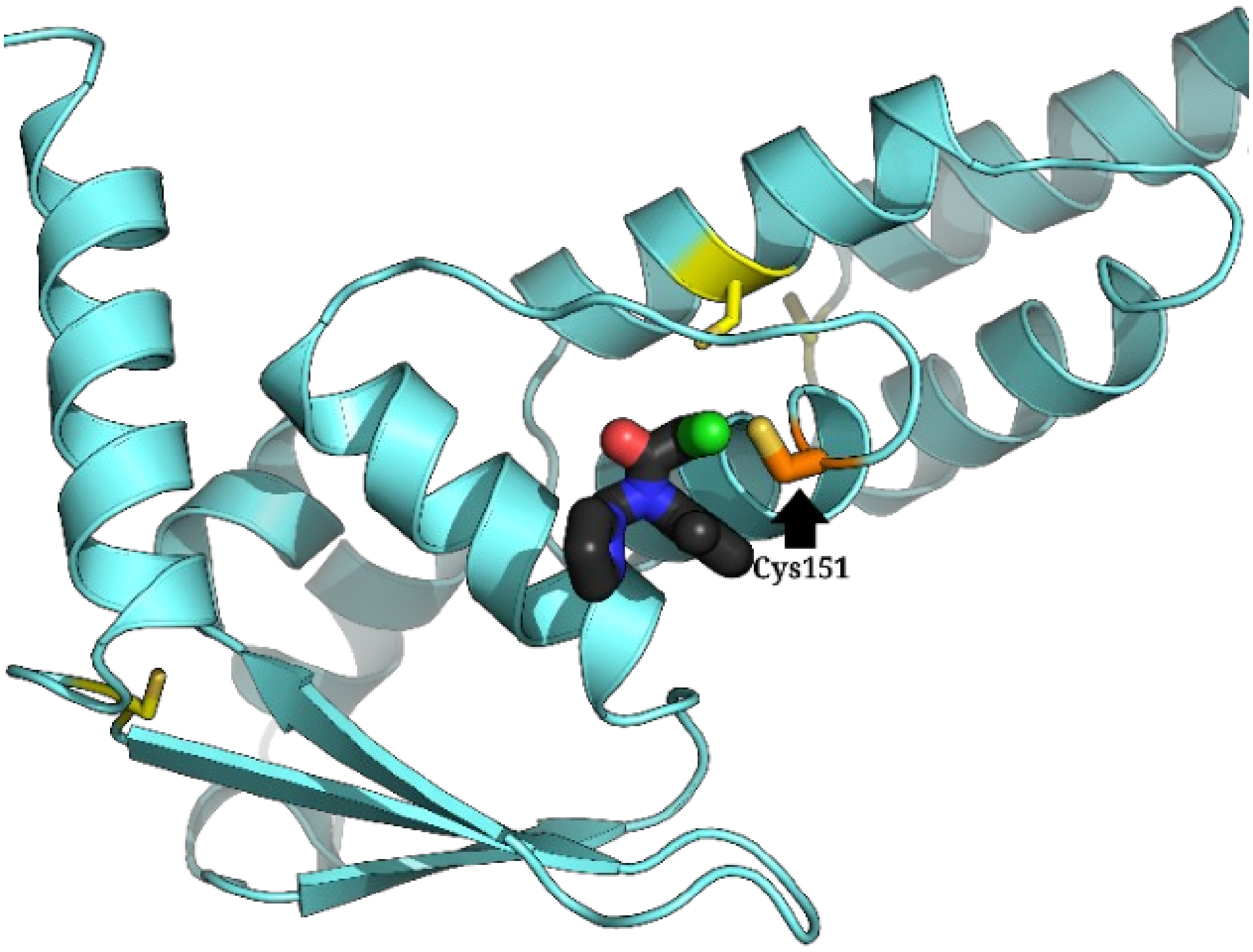

Fig. S19: Molecular docking (MZC). A MZC pose (black ligand) within the fifth best binding site inside the BTB domain (site E, -4.8 kcal/mol) of hKeap1 predicted structure (cyan cartoon). Canonical cysteine residues are shown as orange sticks, the remaining ones as yellow lines.

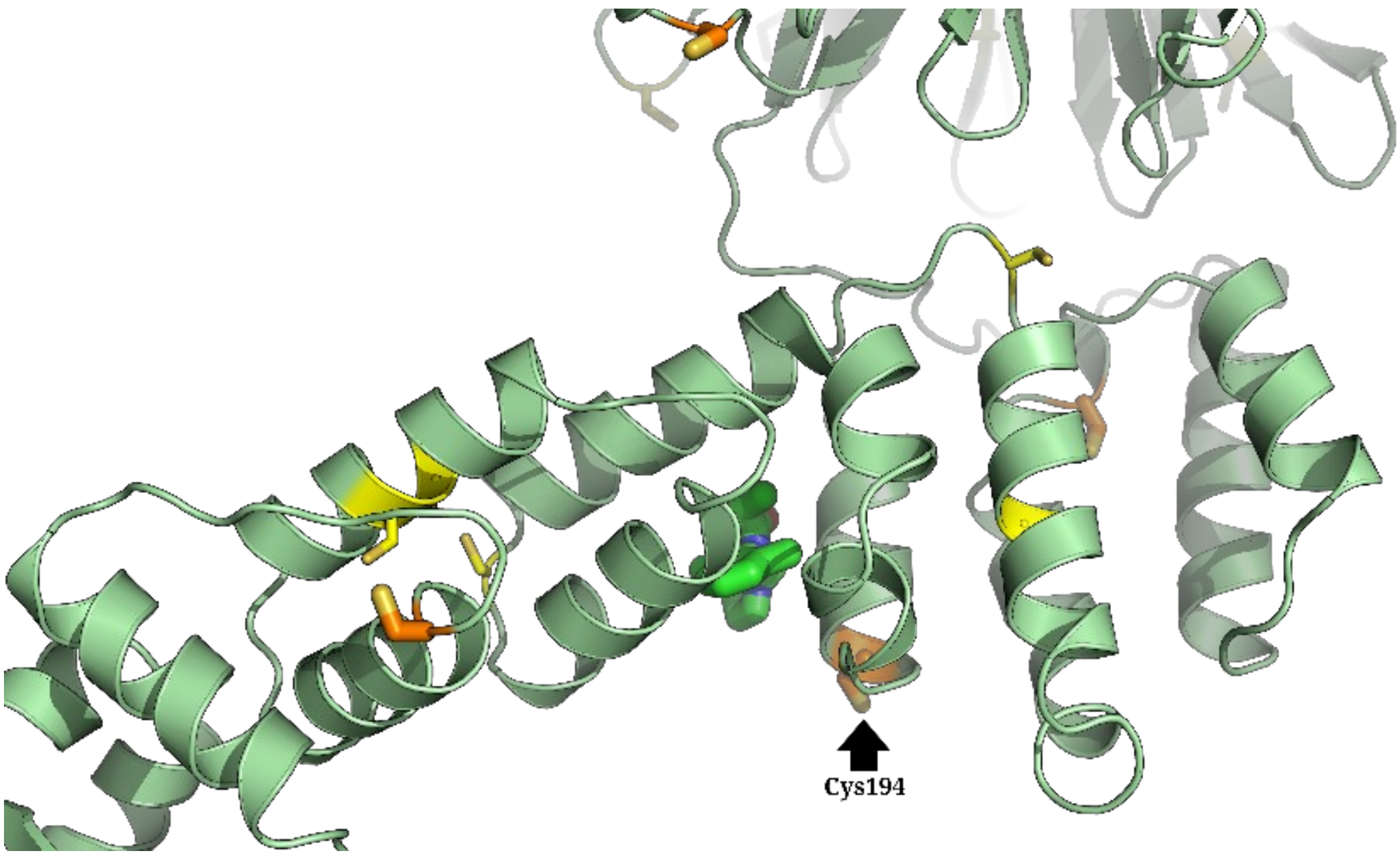

Fig. S20: Molecular docking (MZC). MZC (green ligand) within the best-energy binding site (site F, -5.6 kcal/mol) of the zfKeap1b IVR domain (light grey cartoon). Canonical cysteine residues are shown as orange sticks, the remaining ones as yellow lines. The enumerated cysteine 194 corresponds to Cys226 in hKeap1.

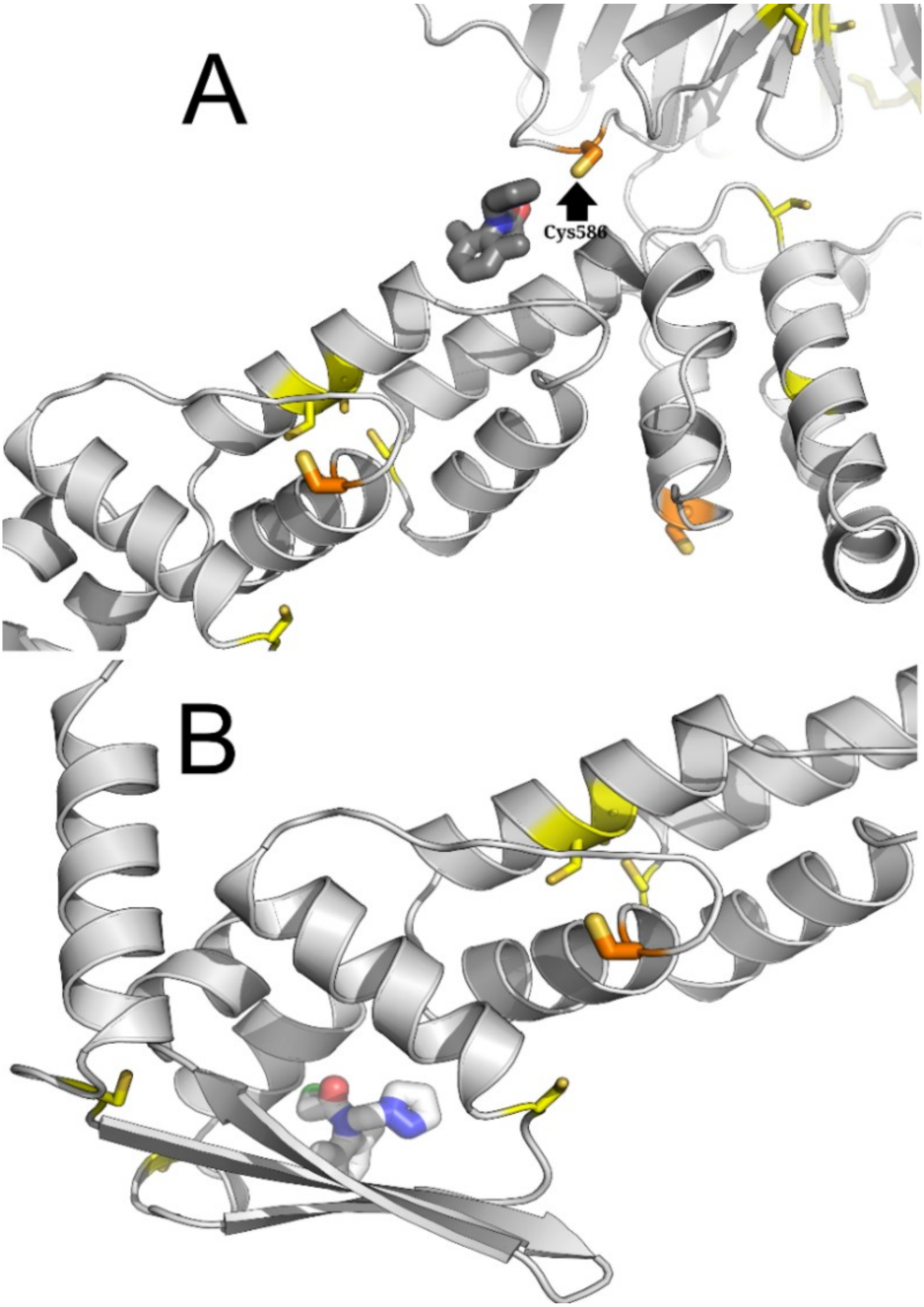

Fig. S21: Molecular docking (MZC). MZC (coloured ligands) in other binding sites (site G, -5 kcal/mol, A, and site H, -4.9 kcal/mol, B) of zfKeap1a (light grey cartoon). Canonical cysteine residues are shown as orange sticks, the remaining ones as yellow lines. The enumerated cysteine 588 corresponds to Cys613 in hKeap1

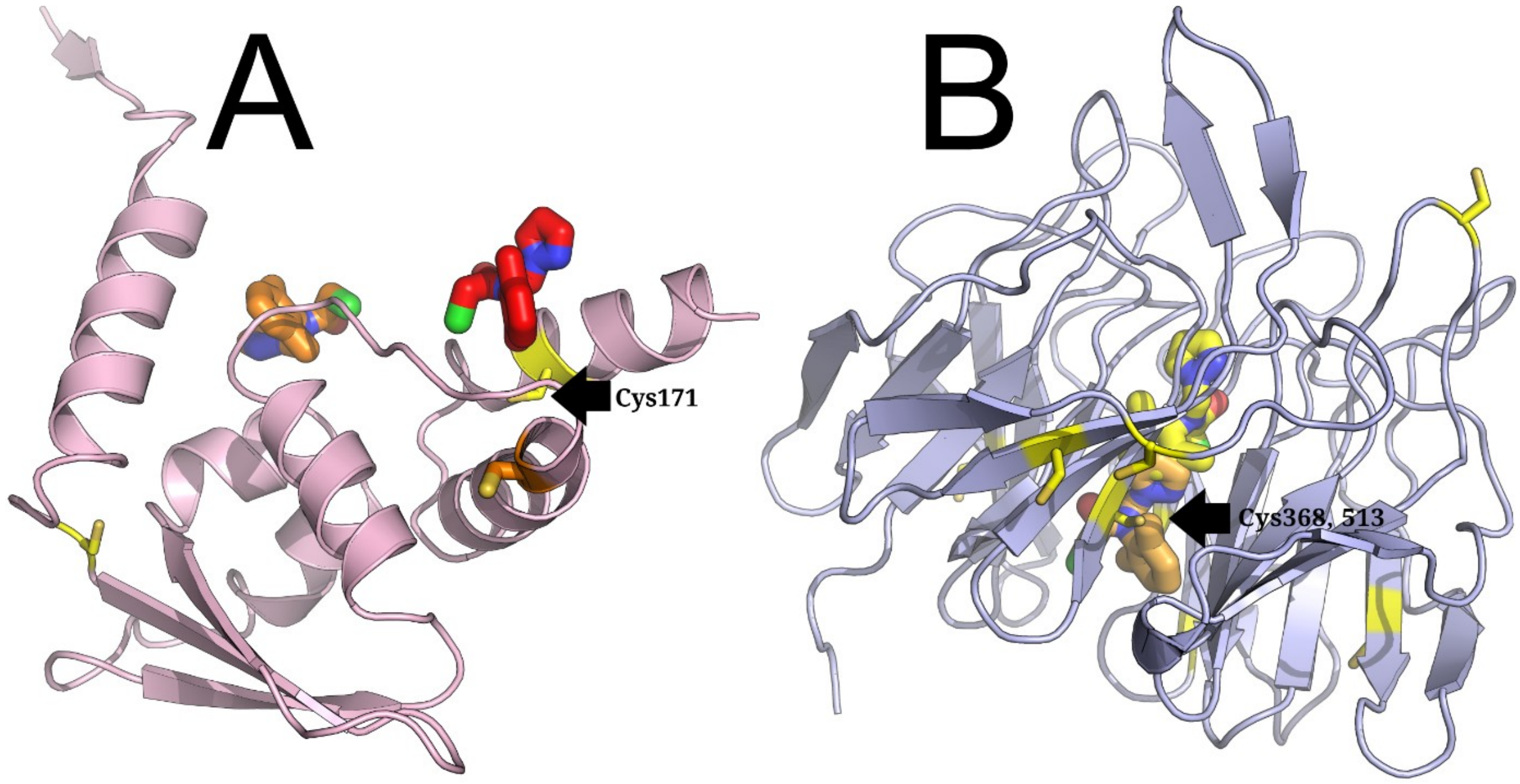

Fig. S22: Molecular docking (MZC). Best energy poses of MZC (coloured ligands) per site from blind docking to X-ray structures PDB ID 7EXI (light pink cartoon, A) and 1ZGK (light purple cartoon, B). The poses and binding sites are highlighted in colour, with yellow indicating the best-predicted binding energy (site A, -6.3 kcal/mol) and red the worst (site I, -4.9 kcal/mol). Canonical cysteine residues are highlighted as orange sticks branching from the protein's backbone, while the remaining cysteines are in yellow. All illustrative data is summarised quantitatively in tabs. 5 and 6 (DIB_B article).

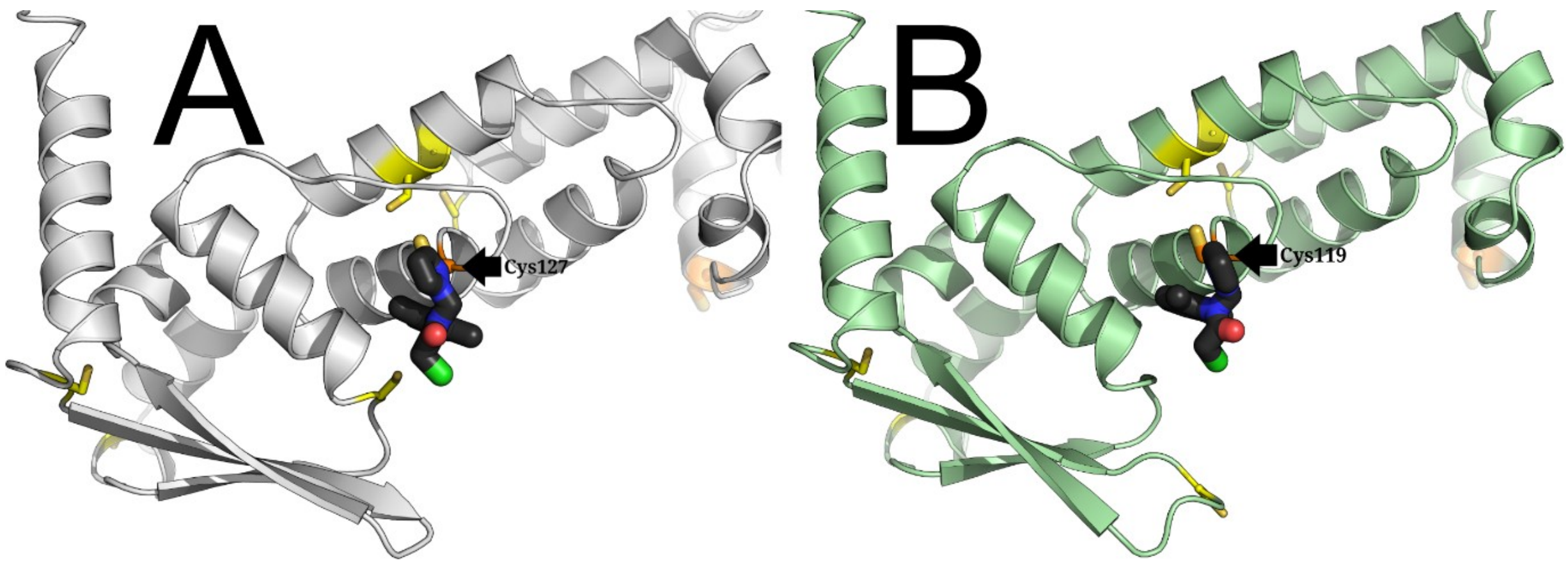

Fig. S23: Molecular docking (MZC). The best-energy poses of MZC (site E, black ligands) from site-directed docking to the proximity of the canonical Cys151 residue of AlphaFold-predicted structures of zfKeap1a (light grey cartoon, -3.6 kcal/mol, A) and zfKeap1b (light green cartoon, -4.1 kcal/mol, B). Reactive cysteines 127 and 119 (corresponding to Cys151 in hKeap1) are shown as orange sticks, while the remaining cysteines are in yellow. All illustrative data is summarised quantitatively in tabs. 5 and 6 (DIB_B article).

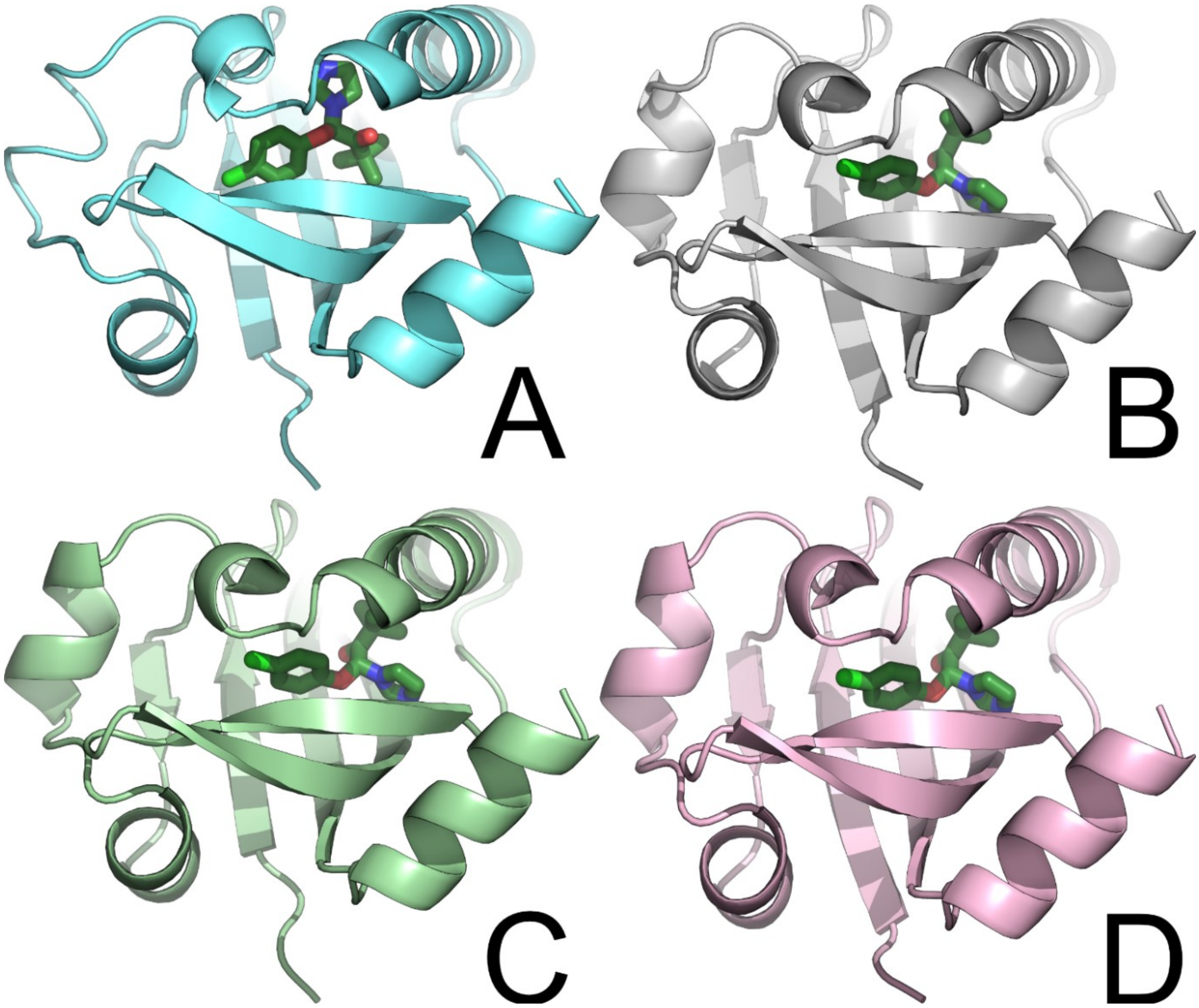

Fig. S24: Molecular docking (CLB). The best-energy poses of climbazole (CLB, green ligands) from site-directed docking to the PAS-B domain of human (hAhR, A) and zebrafish (zfAhR1a (B), zfAhR1b (C), zfAhR2 (D)) aryl hydrocarbon receptor variants. Up to ten best poses were computed; the quantitative data are given in tab. 8 (DIB_B article).

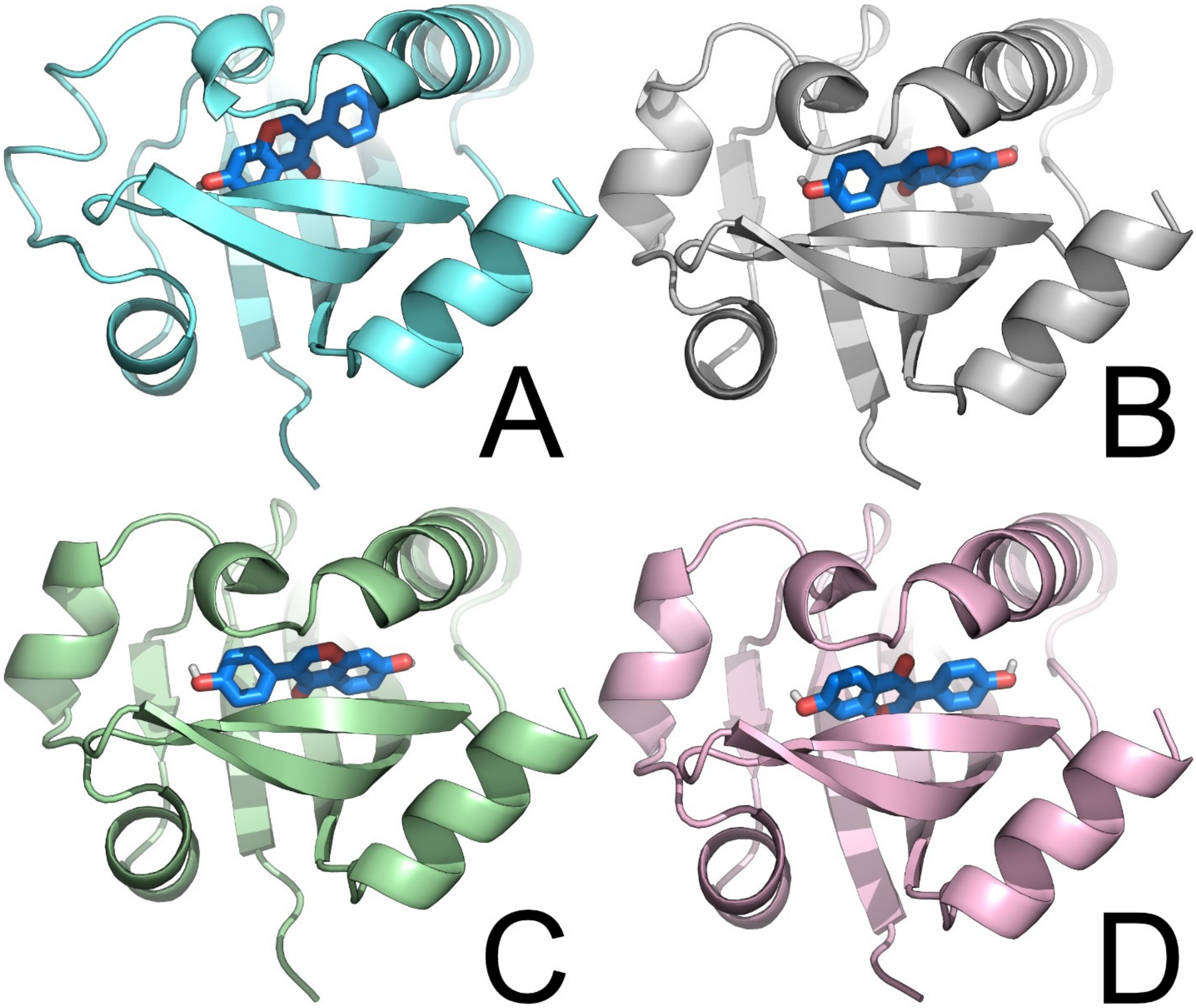

Fig. S25: Molecular docking (DAI). The best-energy poses of daidzein (DAI, blue ligands) from site-directed docking to the PAS-B domain of human (hAhR, A) and zebrafish (zfAhR1a (B), zfAhR1b (C), zfAhR2 (D)) aryl hydrocarbon receptor variants. Up to ten best poses were computed; the quantitative data are given in tab. 8 (DIB_B article).

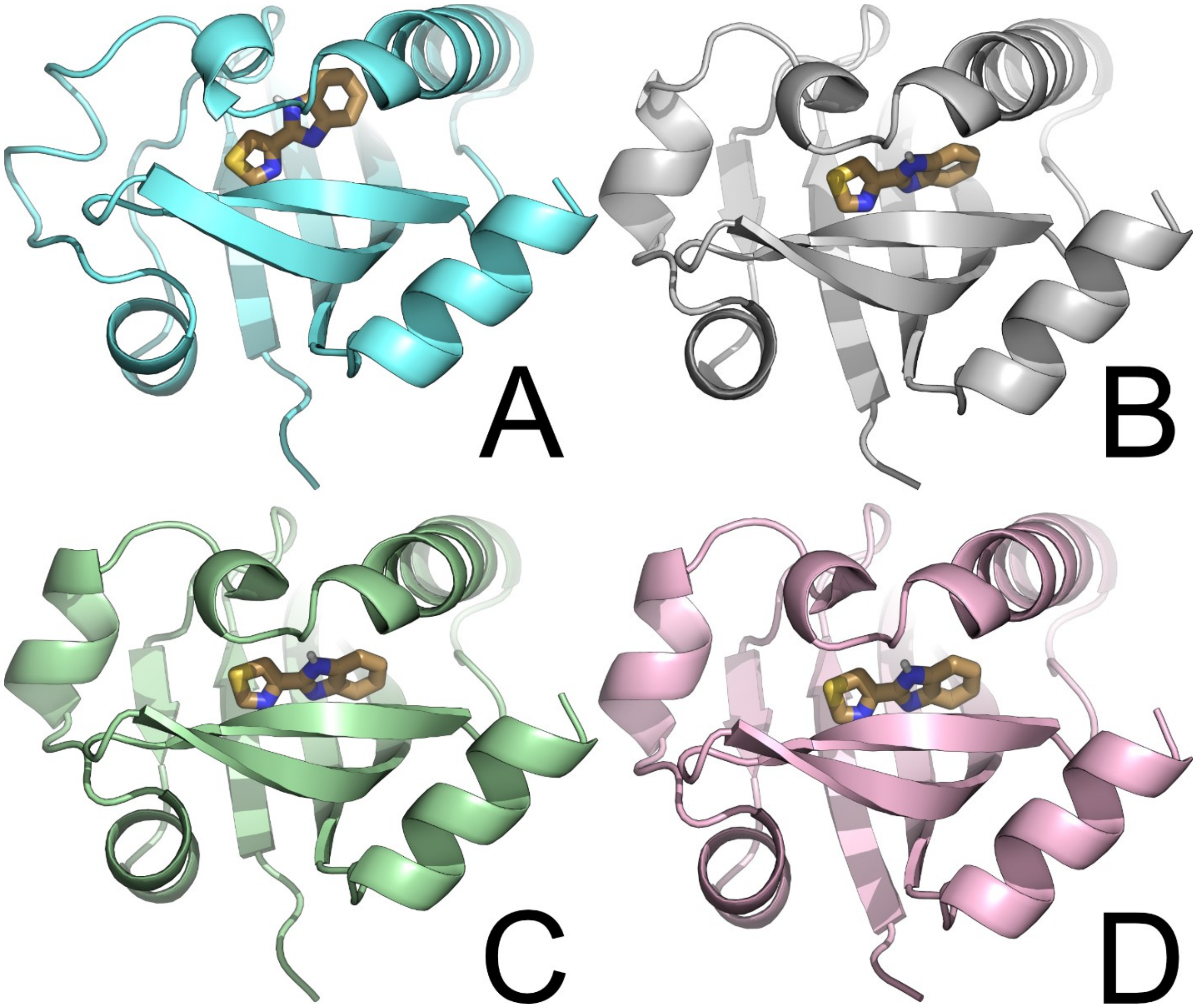

Fig. S26: Molecular docking (TBDZ). The best-energy poses of thiabendazole (TBDZ, ochre ligands) from site-directed docking to the PAS-B domain of human (hAhR, A) and zebrafish (zfAhR1a (B), zfAhR1b (C), zfAhR2 (D)) aryl hydrocarbon receptor variants. Up to ten best poses were computed; the quantitative data are given in tab. 8 (DIB_B article).

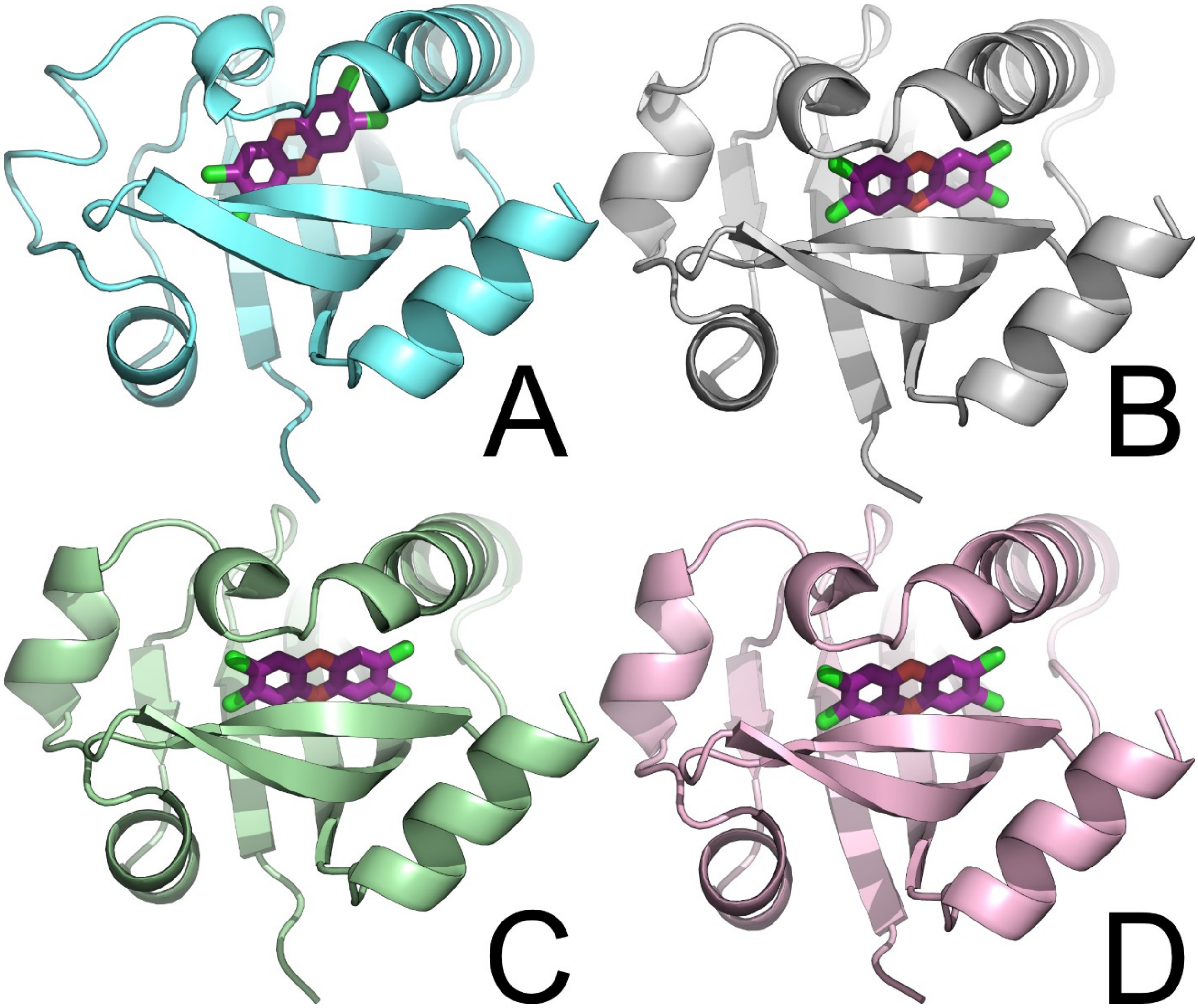

Fig. S27: Molecular docking (TCDD). The best-energy poses of 2,3,7,8-Tetrachlorodibenzodioxin (TCDD, purple ligands) from site-directed docking to the PAS-B domain of human (hAhR, A) and zebrafish (zfAhR1a (B), zfAhR1b (C), zfAhR2 (D)) aryl hydrocarbon receptor variants. Up to ten best poses were computed; the quantitative data are given in tab. 8 (DIB_B article).

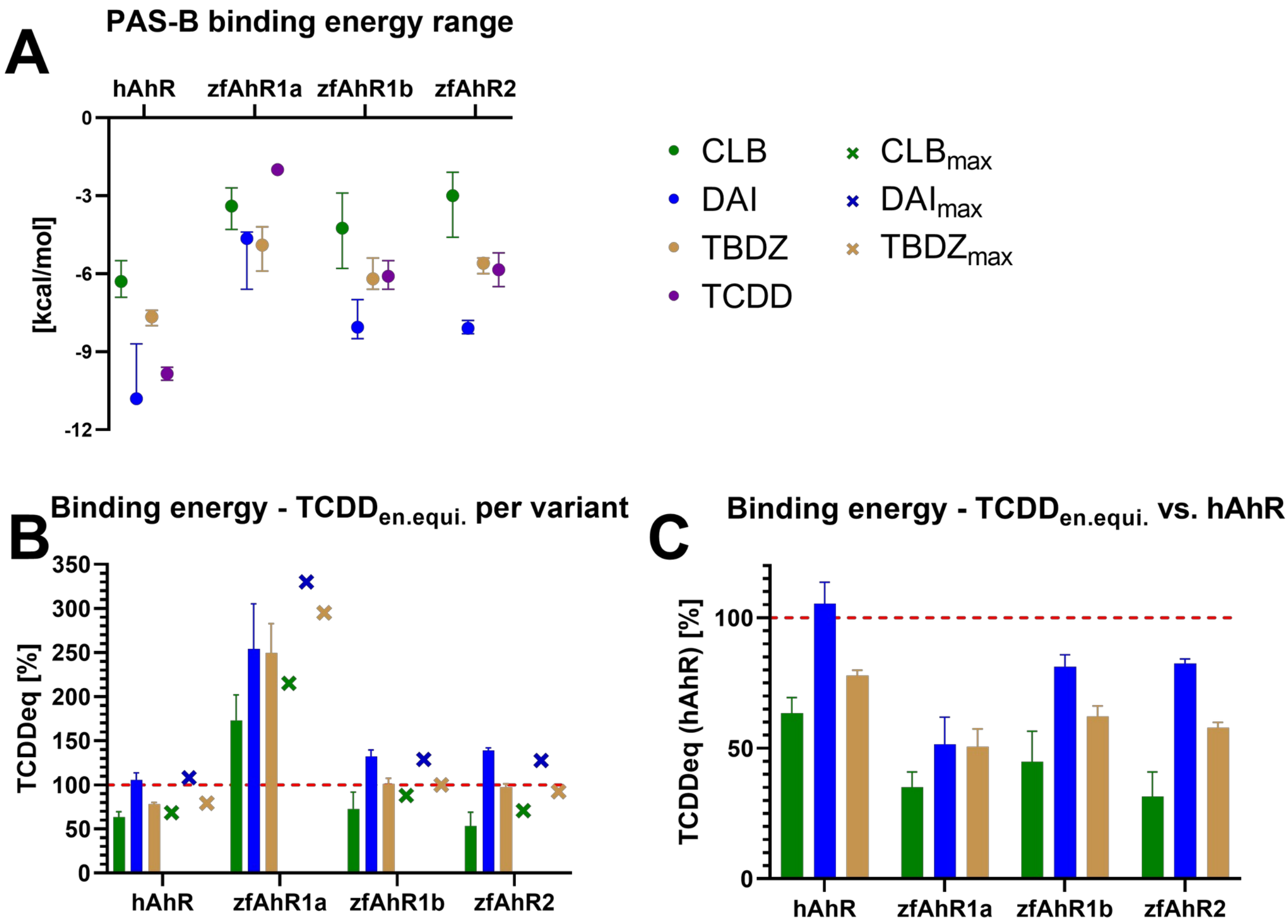

Fig. S28: Molecular docking summary. (A) Range of derived binding energies in kcal/mol per compound (Climbazole (CLB), Daidzein (DAI), Thiabendazole (TBDZ), and 2,3,7,8-tetrachlorodibenzodioxin (TCDD)) for every respective receptor variant. Dots indicate the medians, and whiskers extend to 10 poses. (B) TCDD binding energy equivalents in per cent, normalised to the respective variant's mean TCDD binding energy value. Boxes indicate the mean energy equivalents with standard deviations. Crosses illustrate the equivalent of the best energy pose of the respective compound, normalised to the best energy pose of TCDD. (C) TCDD binding energy equivalents in per cent, normalised to the mean hAhR TCDD binding energy value. Boxes indicate the mean energy equivalents with standard deviations. All data and calculations are given in SI11.

3.2 Supplementary results: Main article – Summarised cytotoxicity/cellular viability

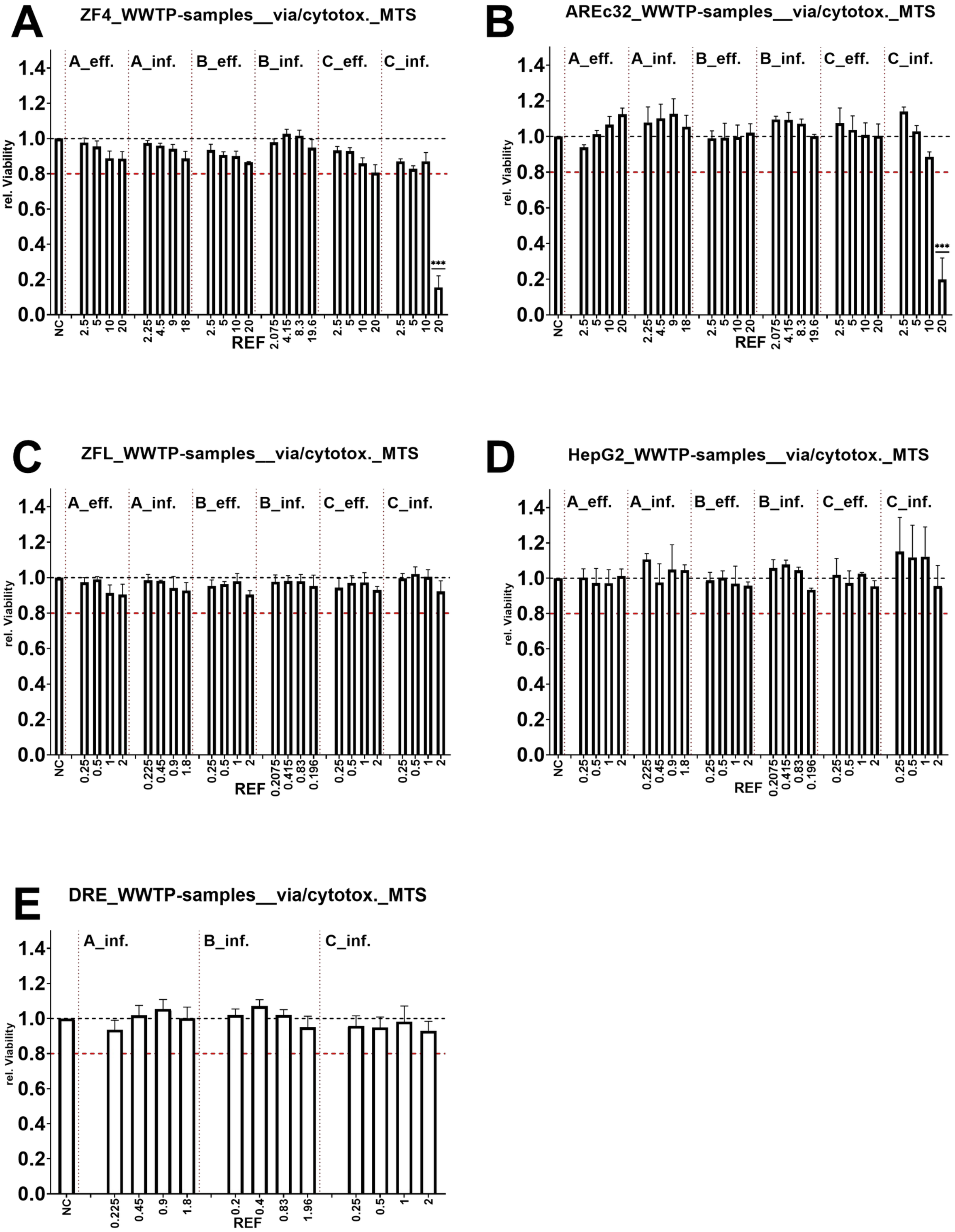

Fig. S29: Cytotoxicity/cellular viability impact assessment of the environmental samples from WWTP sites A to C, conducted in the zebrafish (ZF4 and ZFL), human (MCF7/AREc32 (AREc32) and HepG2), and mouse (DRE) reporter lines. Viability corresponds to control-normalised mitochondrial metabolism, as measured by the MTS assay. Each bar represents the mean, including SEM (experimental units n = 3-4; observational units N = 9-12). All statistical comparisons versus the

negative vehicle control (NC) were conducted using one-sided Dunnett's tests (alpha level = 0.05; ***p<0.001) on pooled raw data (see SI1 to SI8 for raw data and details on calculations).

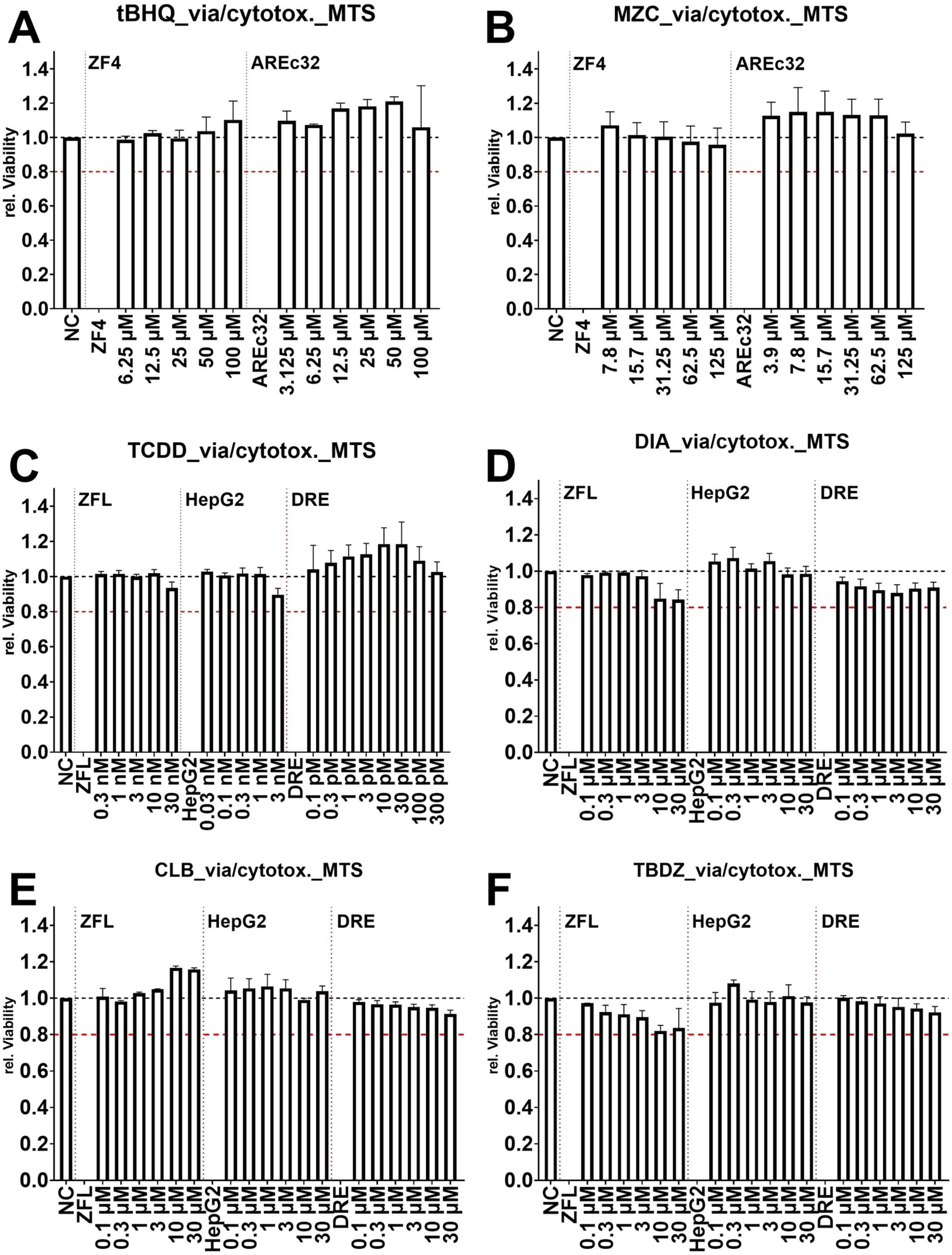

Fig. S30: Cytotoxicity/cellular viability impact assessment of reference control and ISEDA-identified compounds, conducted in the zebrafish (ZF4 and ZFL), human (MCF7AREc32 (AREc32) and HepG2), and mouse (DRE) reporter lines. Viability corresponds to control-normalised mitochondrial metabolism, as measured by the MTS assay. Each bar represents the mean, including SEM (experimental units n = 3-4; observational units N = 9-12). All statistical comparisons versus the

negative vehicle control (NC) were conducted using one-sided Dunnett's tests (alpha level = 0.05) on pooled raw data (see SI1 to SI8, SI14, and SI15 for raw data and details of calculations).

3.3 Supplementary results: Main article – Optimised data fitting strategies for the oxidative stress MoA

In the main article (section 3.1), we recognised that simple linear regression is suboptimal for modelling the CRC relationship of the MZC reference compound due to its hyperbolic profile (fig. S31B). Conversely, a linear model adequately described the tBHQ data (fig. S31A). To achieve an optimal fit for both datasets, we implemented a four-parameter log-logistic nonlinear regression (4PNL) with modifications described by Weimer et al. [51]. The formulation of the 4PNL model is delineated in section 3.4, below.

However, calculating BEQ values from nonlinear regressions is problematic due to variable slope values [55]–[57], potentially leading to significant inaccuracies [57]. Escher et al. recommended estimating parameters only from the initial 30% of the response curve, where the function is approximately linear. This guidance is considered the canonical approach and has been adopted within the main article. Despite this, enhanced parametric flexibility (four parameters and beyond) provided by the 4PNL model could invalidate this assumption. A data-driven, multi-parametric model might be more robust, particularly for the oxidative stress MOA, without a receptor-saturation plateau, where effects likely transition into cytotoxicity. An alternative solution to this challenge is proposed herein, as follows.

Both tBHQ and MZC reference compound data were fitted to the named 4PNL model (fig. S31C+D). Most statistical software, such as GraphPad Prism or respective R packages, generates a curve consisting of many estimates that can be interrogated and retrieved. We retrieved the 4PNL curve estimates for tBHQ and MZC up to 30% of the maximum response, which showed no cytotoxicity (see SI16, tab “Nrf2-30%fit”), and utilised them to recalibrate a simple linear regression (designated “30%SL” hereafter; see fig. S31E+F).

This revised method rectifies the inadequate fits of the MZC data by linear regression (fig. S31F), maintaining mathematical integrity for BEQ estimations and conserving mechanistic induction discrepancies. While the fit-adjustment processes slightly alter the ratios between zebrafish and human estimates (fig. S33), the ratios for tBHQ consistently exceed unity (4PNL: 1 to 2; 30%SL: 1.6 to 3.4), suggesting mechanistic discrepancies. Moreover, differences in response to tBHQ are less noticeable at an induction level of 1.5 after normalisation to vehicle control.

For the main article, all non-cytotoxic concentrations were retained for regression fitting, provided that the corresponding concentration-response data exhibited approximately semi-linear behaviour

within the tested exposure range. Thus, no fixed IR4/IR5 (induction ratio 4 or 5) cut-off was applied to the oxidative stress MoA datasets. Instead, cytotoxicity was assessed in parallel (see above), and cytotoxic concentrations were excluded from subsequent analyses, consistent with recent AREc32-based applications [58]. Similarly, for the xenobiotic metabolism MoA, concentrations exceeding 30% of the maximum TCDD-induced response were not automatically excluded if the corresponding concentration-response relationship remained approximately semi-linear and non-cytotoxic within the tested range (one incidence, influent sample C at REF 2 in the HepG2 assay).

This approach was chosen because the study's primary aim was to compare mechanistic response patterns across species and assays, rather than to optimise each individual concentration-response relationship using a canonical fitting procedure. Retaining all valid, non-cytotoxic data points maintained a consistent data-analysis workflow across samples and assays, avoiding the need for sample- and assay-specific exclusion criteria that would require separate downstream statistical procedures.

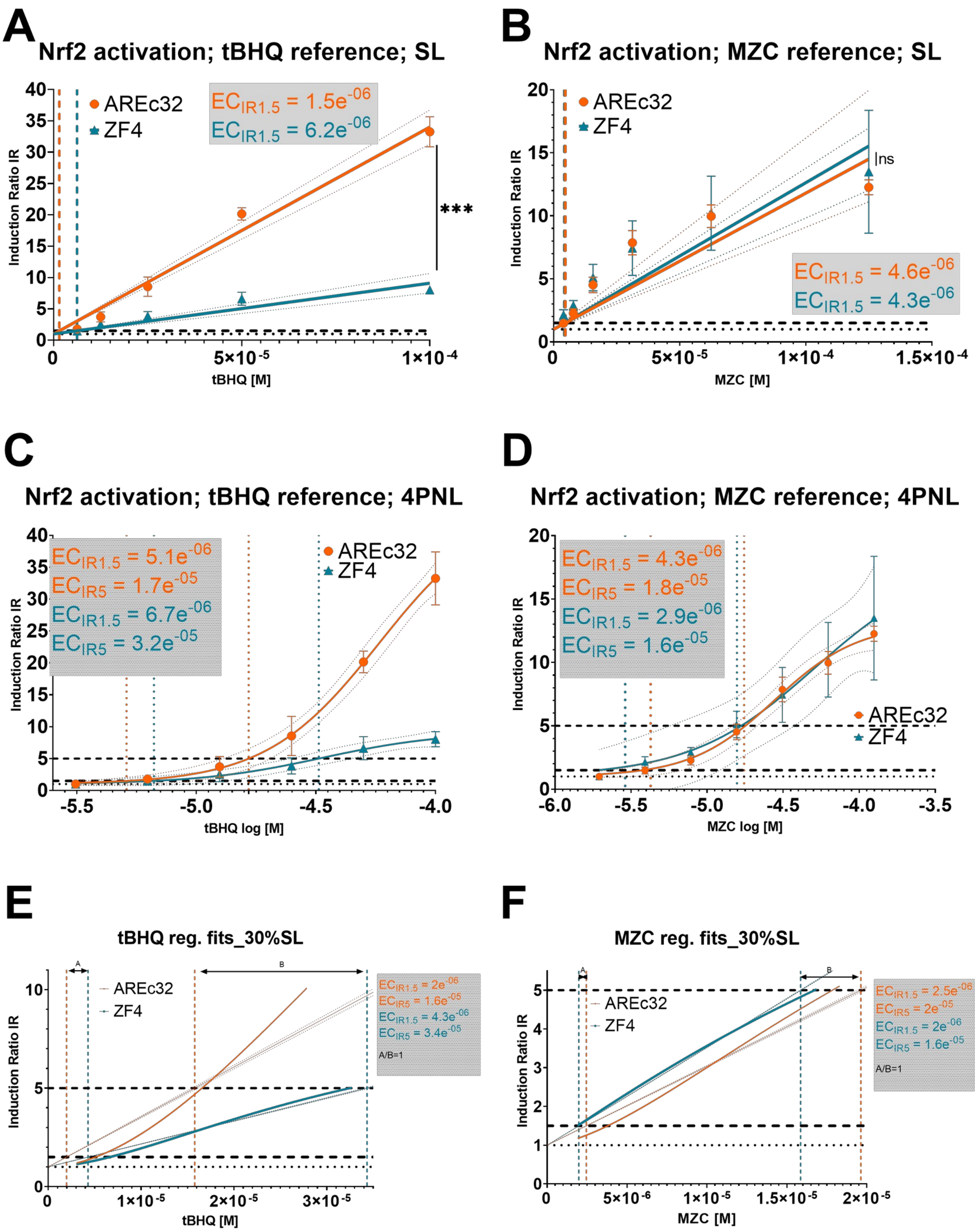

Fig. S31: Alternative data fitting (tBHQ, MZC). Activation of the cellular oxidative stress response (Nrf2-Keap1-mediated toxicity pathway), depicted as induction ratio (IR) relative to the solvent control, in human (MCF7AREc32 (AREc32, orange) and zebrafish (ZF4, turquoise) reporter gene assays exposed for 24h to the reference compounds tert-Butylhydroquinone (tBHQ, panel A) and metazachlor (MZC, panel B). Points indicate the experimental mean (experimental units n = 3, observational units N = 9-12), with the SEM marked by whiskers. Samples were analysed in a linear serial dilution series, with 100 µM (tBHQ) and 125 µM (MZC) as the highest concentrations. CRCs were fitted using simple linear regression (SL; panels A and B) and are shown in their respective colours, with 95% CIs shown as faded dotted lines. Effect point estimates ($EC_{IR1.5}$, coloured, vertical, and dotted lines) were derived from exposure concentrations inducing an IR of 1.5 (horizontal,

dotted line). The respective values are depicted in the graphs. A two-way ANOVA was conducted to compute differences between effect sizes of the respective reporter line pairs (ns = statistically non-significant, ***p < 0.001). The respective samples' cellular viability/cytotoxicity data are given in the SM, figs. S29 and S30, and detailed in DIB_A. The respective raw and metadata are provided in SI1, SI2, and SI14. Alternatively, CRCs were fitted to a non-linear, log-logistic regression model (4PNL, panels C and D; see equations in section 3.4 below). EC_{IR5} estimates were also derived. Total CRC estimates from the 4PNL model were retrieved up to the point that induced 30% of the maximum effect, and the model was refitted using a simple linear regression (30%SL model; panels E and F; see sections 3.3 and 3.4 below for details).

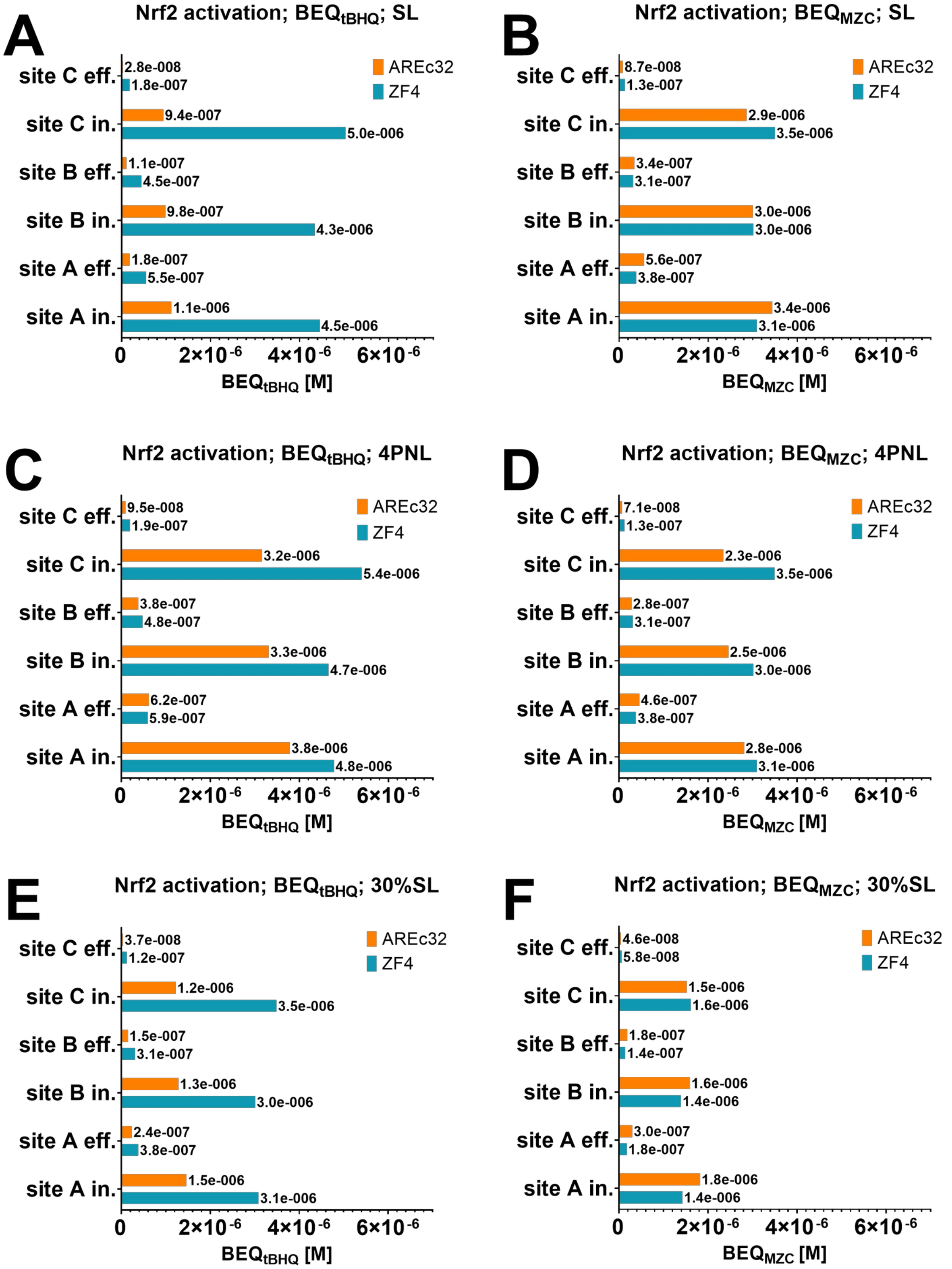

Fig. S32: Alternative data fitting (tBHQ, MZC). Outline of derived tBHQ-BEQ standardisations (panels A, C, and E) and MZC-BEQ standardisations (panels B, D, and F), regarding the used regression model (SL, 4PNL, or 30%SL, see sections 3.3 and 3.4 below).

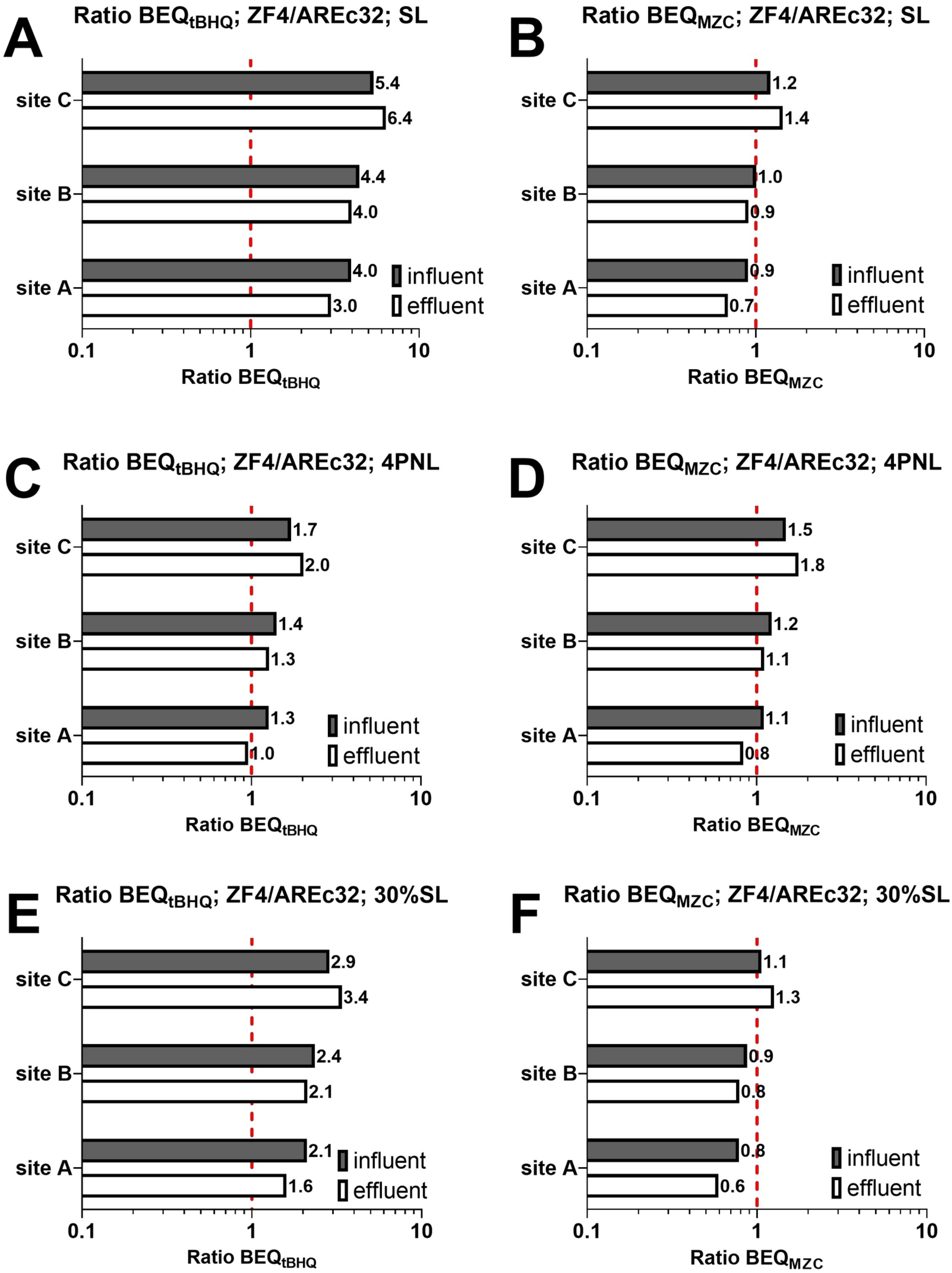

Fig. S33: Alternative data fitting (tBHQ, MZC). Outline of derived zebrafish-to-human BEQ ratios, regarding the used regression model: SL (panels A and B), 4PNL (C and D), and 30%SL (E and F); see details in sections 3.3 and 3.4 below. The unity ratio is illustrated as a dotted red line.

3.4 Supplementary results: Main article – Optimised data fitting strategies for the xenobiotic metabolism MoA

Similar to section 3.3 above, we explored several multi-parameter nonlinear regression models for data fitting. Direct interpolation of BEQ values from sigmoidal curves with variable slopes is deemed incorrect [49], [56], [57], a scenario particularly likely for environmental samples. The alternative is to fit a simple linear regression to the environmental data, given that the response is unlikely to exceed a high percentage increase relative to the utilised reference compound's maximum response. A point estimate from the CRC within the initial 30% is selected, where the response exhibits a quasi-linear trend. Nevertheless, the choice of model and the nature of the empirical data ultimately determine the CRC's form, and the above scenario might not always hold. We fitted xenobiotic metabolism MoA data to elaborate on our point, as delineated in the main article, with the following models:

2PNL:

$$f(x) = min \frac{+max - min}{1 + 10^{(logEC50 - x)}}$$

With upper (max.) and lower (min) asymptotes defined by normalisation to vehicle controls (min.) and maximum TCDD response (max.)

3PNL:

$$f(x) = min \frac{+max - min}{1 + 10^{HillSlope*(logEC50 - x)}}$$

With upper (max.) and lower (min) asymptotes defined by normalisation to vehicle controls (min.) and maximum TCDD response (max.). As such, the model aligns with the recommendations made by Escher et al [50].

4PNL:

$$f(x) = d \frac{+a - d}{1 + 10^{HillSlope*(logEC50 - x)}}$$

With upper (a) and lower (d) asymptotes refitted to the data by the model, as recommended by Weimer et al. [51] .

30%SL: Modelled according to section 3.3, using the first 30% of data from the 3PNL model.

The inadequacy of a simple linear regression for deriving EC_{10} values for BEQ calculation is evident from the fig. S36B, D, and F, necessitating a sigmoidal fit. Application of the 2PNL, 3PNL, and 4PNL models to ZFL and HepG2 data yielded satisfactory fits for all models (fig. S34). However, the 2PNL

model inaccurately estimates the upper and lower bounds of the empirical curve for DRE data (fig. S34B), leading to a substantial underestimation of EC_{10}. For example, the EC_{10} estimate in question is misjudged by almost half an order of magnitude.

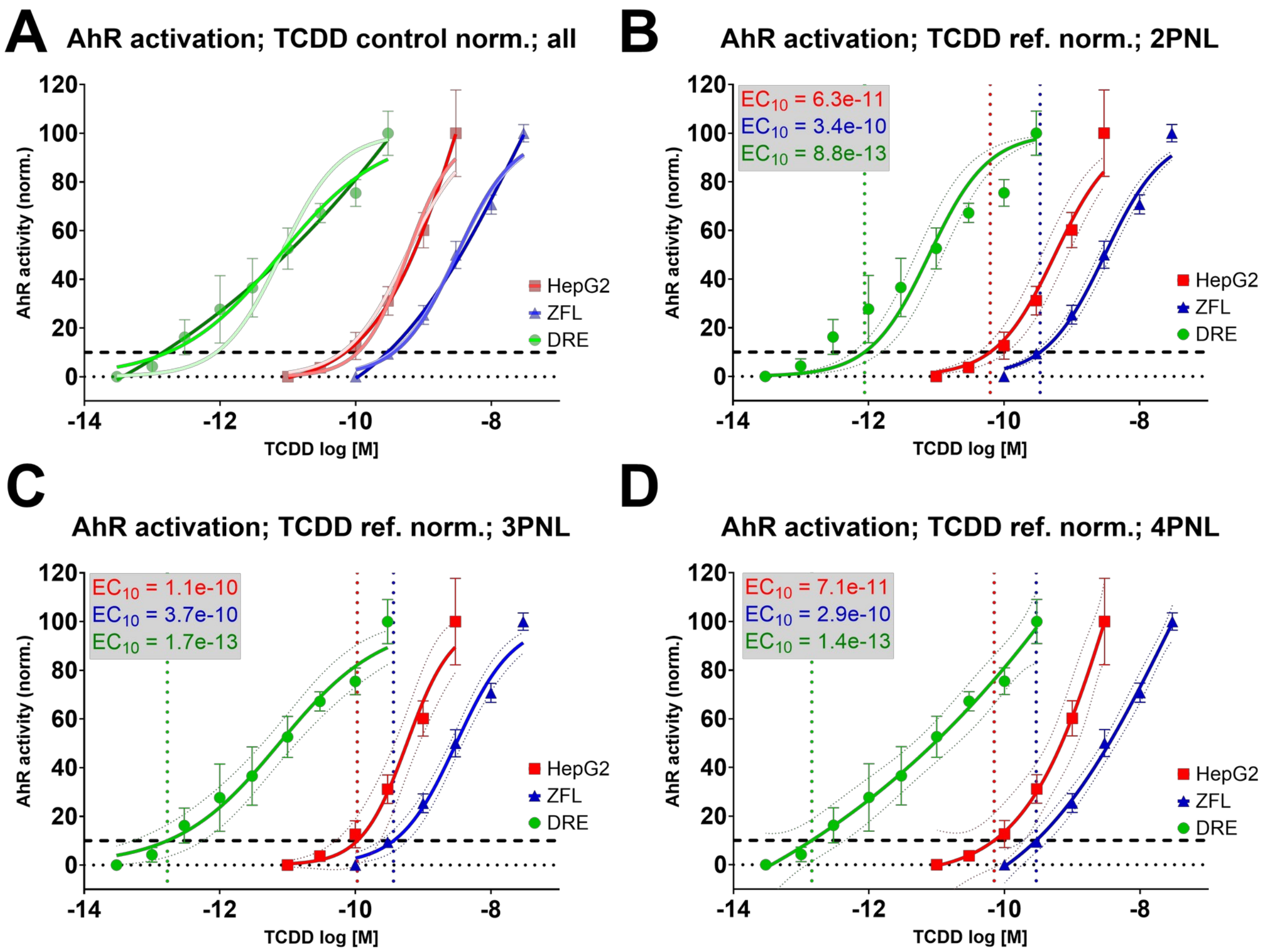

Fig. S34: Alternative data fitting (TCDD). Activation of the cellular xenobiotic metabolism (AhR-ARNT-mediated toxicity pathway), depicted as a minimum (solvent control background) to maximum TCDD-induced response normalisation, in human (HepG2, red), zebrafish (ZFL, blue), and mouse (DRE, green) reporter gene assays exposed for 24 h to the reference compound TCDD. Points indicate the experimental mean (experimental units n = 3-5, observational units N = 9-22), with the SEM marked by whiskers. The data were analysed via various non-linear, log-logarithmic regression models (panel B: 2PNL, panel C: 3PNL, panel D: 4PNL, panel A: merge of the latter) and are given in their respective colours, with 95% CIs as faded, dotted lines. Effect point estimates (EC_{10}; coloured, vertical, and dotted lines) were derived from the concentrations that cause 10% of the maximum response (horizontal dotted line). The respective values are depicted within the graphs.

Examining the lower 30% of the DRE reporter line response across 2PNL, 3PNL, and 4PNL models on logarithmic and linear scales revealed differing behaviours within these bounds (fig. S35). Only the 2PNL model exhibited a semi-linear characteristic. At the same time, the 3PNL and 4PNL fits were relatively hyperbolic (fig. S35B), presenting a dilemma between choosing a suboptimal 2PNL fit or disregarding the linearity assumption for BEQ extrapolation. Instead, we adopted the 30%-refitting approach from the 3PNL data, indicating a near-linear response up to a 10% threshold.

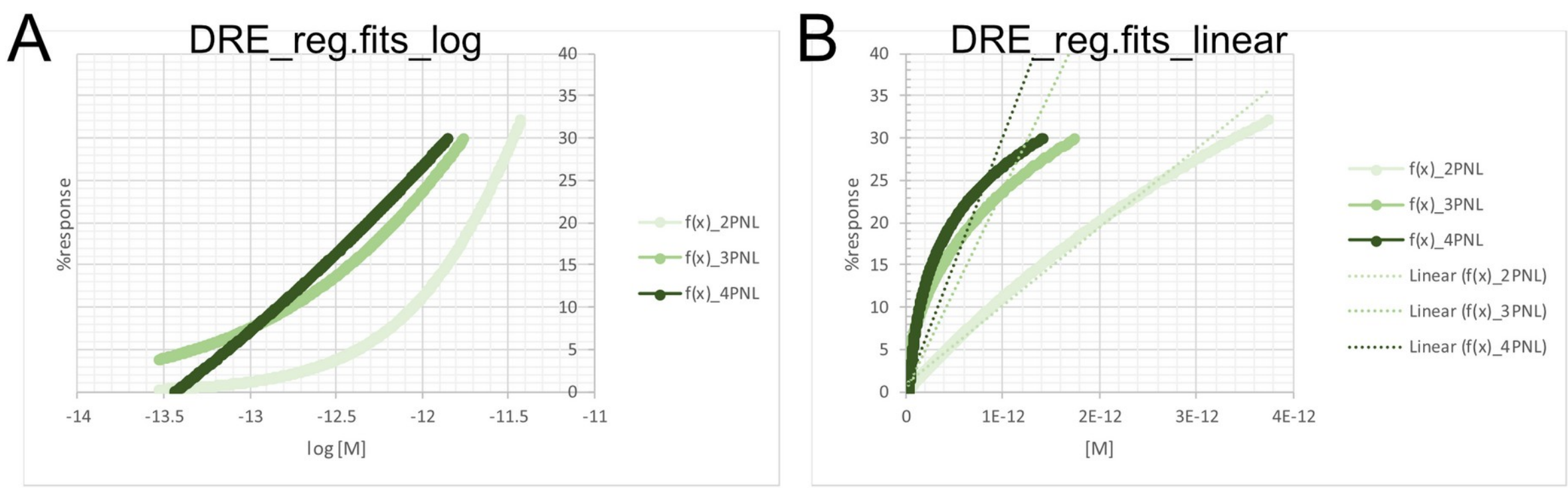

Fig. S35: Alternative data fitting (TCDD). The lower 30% of the 2PNL, 3PNL, and 4PNL model fits; displayed on a logarithmic (panel A) and linear (panel B) scale. The data were taken from the DRE assay results.

Figures S36C and E show that the 30%SL refitting yields estimates comparable to those of the 3PNL model. While unnecessary in this instance, as the data already exhibited a semi-linear character, it suggests that this approach does not introduce distortion. Although not ideal, the refitting of DRE data (fig. S36A) reduces the error inherent in the 2PNL model by more than half and closely matches the 3PNL at EC_{20} (see fig. S36A).

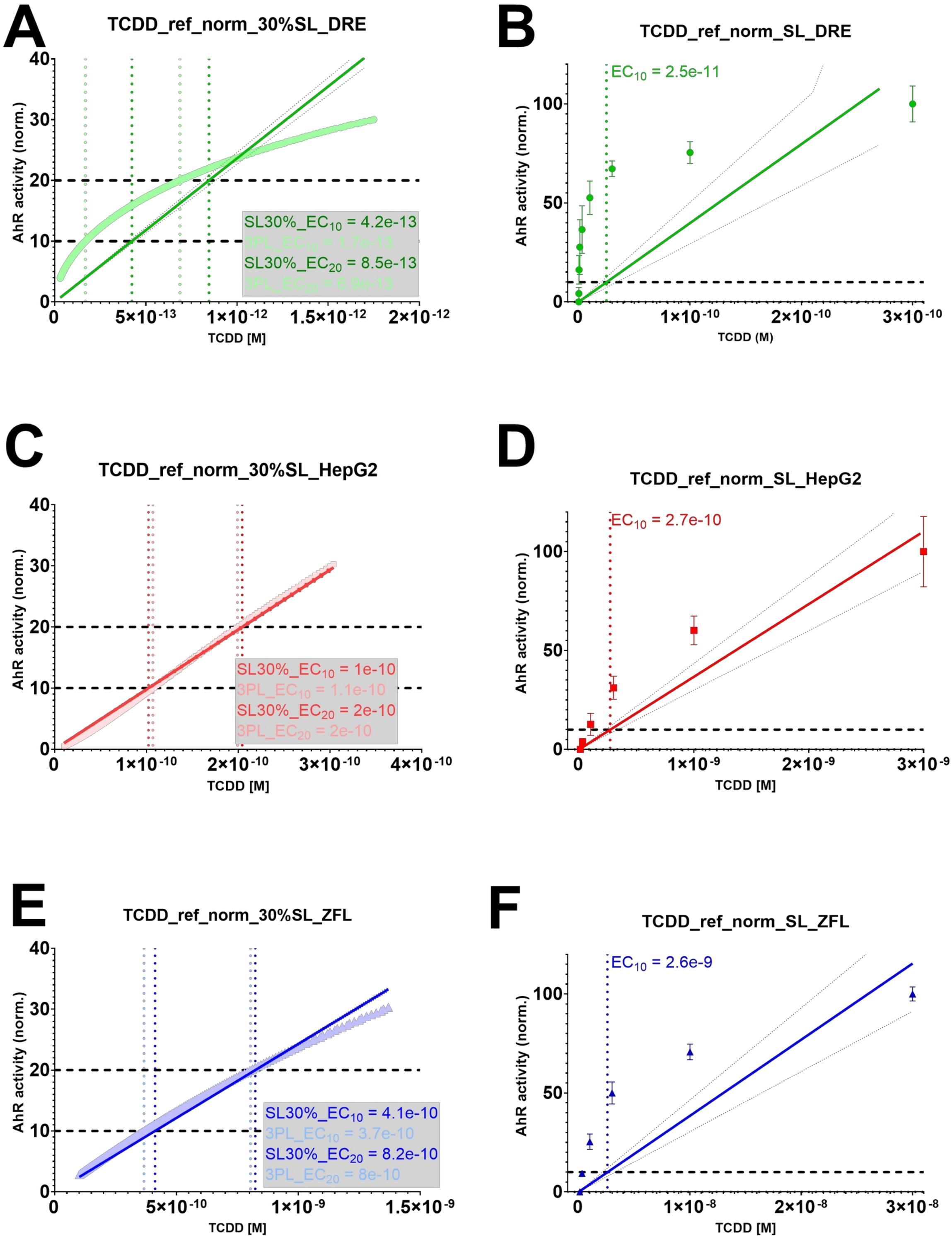

Fig. S36: Alternative data fitting (TCDD). Activation of the cellular xenobiotic metabolism (AhR-ARNT-mediated toxicity pathway), depicted as a minimum (solvent control background) to maximum TCDD-induced response normalisation, in human (HepG2, red), zebrafish (ZFL, blue), and mouse (DRE, green) reporter gene assays exposed for 24 h to the reference compound TCDD. Points indicate the experimental mean (experimental units n = 3-5, observational units N = 9-22), with the SEM marked by whiskers. CRCs were fitted using simple linear regression (panels B, D, and E) and are shown in their respective colours, with 95% CIs shown as faded dotted lines. Alternatively, total curve estimates derived from the 3PNL

model were refitted to a simple linear regression (panels A, C, and E). Effect point estimates (EC_{10} and EC_{20}; coloured, vertical, and dotted lines) were derived from the concentrations that cause 10 or 20% of the maximum response (horizontal dotted line).

Tab. S4: Effect estimates (EC10 or EC20) ratios, as derived from various DRE data model fits.

**Pair**	**Ratio**
2PNL/3PNL	5.2
2PNL/4PNL	6.3
2PNL/30%SL	2.1
30%SL/3PNL	2.5
30%SL/4PNL	3
30%SL/3PNL at EC20	1.2

Comparing different model-derived CRC estimates (EC_{10}, $_{20}$, and $_{50}$), the 30%-refitting method appears consistent with standard sigmoidal fits (fig. S37), both when data naturally aligns with semi-linearity (fig. S37B+C) and when the sigmoidal curve inaccurately characterises the empirical data or breaches the semi-linearity assumption (fig. S37A). Therefore, this novel strategy merits consideration as a standard evaluative procedure, given its safeguard against misinterpretation and its preservation of linearity. Nonetheless, more extensive, additional validation is required before drawing definitive conclusions about its broader applicability.

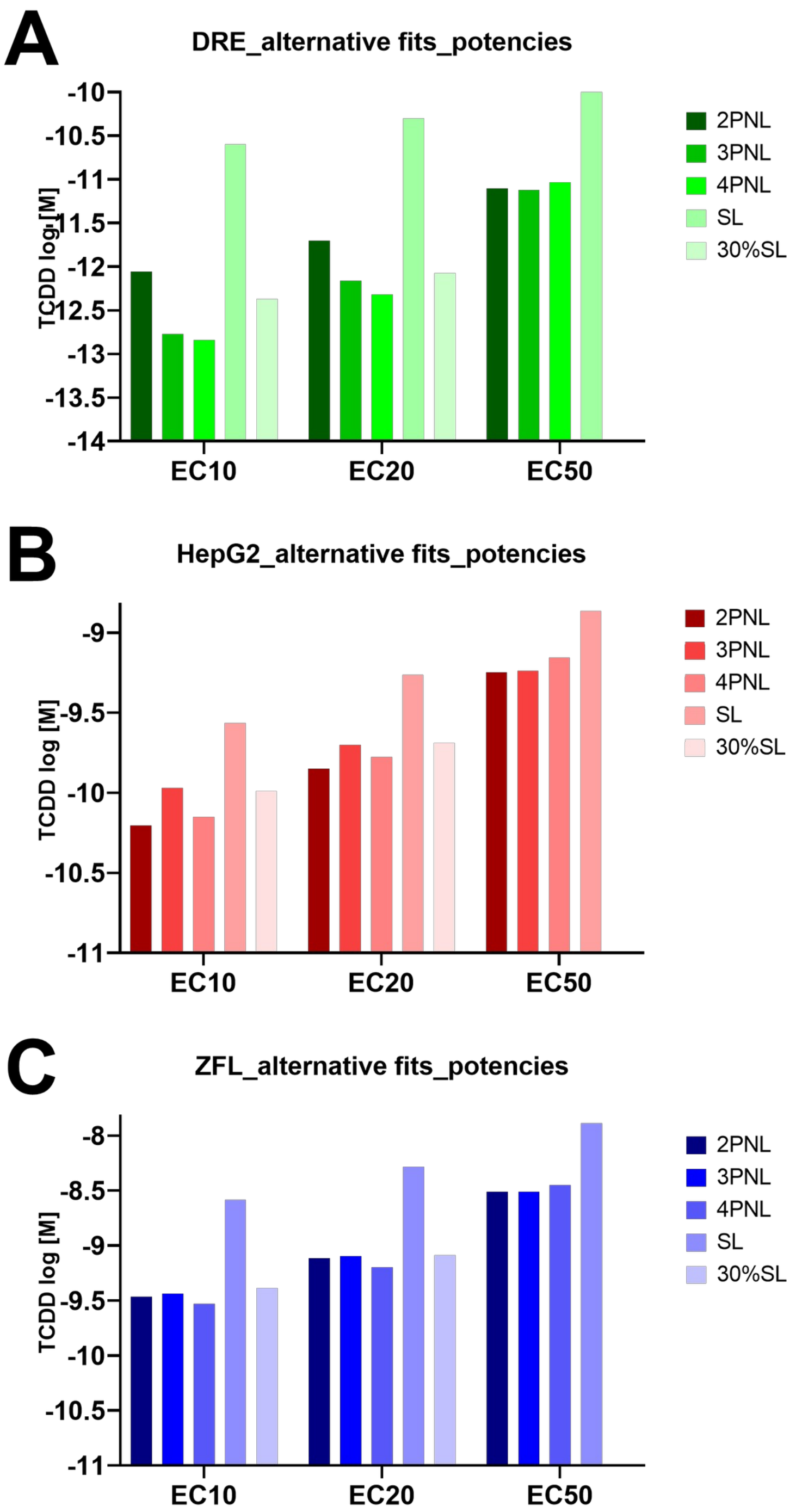

Fig. S37: Alternative data fitting (TCDD). Comparison of point-effect estimate performance among the indicated models. The applied regression models are described in section 3.4.

Subsequent meta-analyses mirrored those in the main article, converting model estimates to TCDD-BEQ values and comparing zebrafish and mammalian estimates and ratios (figs. S38 and S39). A simple linear regression (SL) distorts the data, exaggerating differences between ZFL and HepG2 while underestimating those between ZFL and DRE (fig. S39A). Except for the site C influent, all nonlinear regression models (2PNL, 3PNL, 4PNL) predict a half- to an entire-order-of-magnitude difference in TCDD-BEQs between the zebrafish and the human reporter line. We conclude that,

except for the site C influent sample, the TCDD-BEQ standardisation procedure does not sufficiently account for differences in species sensitivity.

Zebrafish-to-mouse estimates and potency differences depend on the chosen model. For 2PNL, the observed ratios were the lowest, ranging from 319 (site A influent) to 784 (site B influent); keeping in mind that the respective estimate was derived from a semi-linear curve but insufficiently represented empirical data (fig. S39B). Both 3PNL and 4PNL yielded satisfactory fits yet breached the semi-linearity premise, resulting in a threefold disparity in potencies (3PNL ratios: 1773 to 4359; 4PNL: 1675 to 4118; fig. S39C+D). The 30%SL adjustment aligns potencies more closely, although a significant magnitude difference persists (ratios 795 to 1952, fig. S39E). In conclusion, for environmental mixture effects, particularly between zebrafish (ZFL) and mouse (DRE) reporter lines, the TCDD-BEQ normalisation does not adequately correct for species-specific differences in sensitivity.

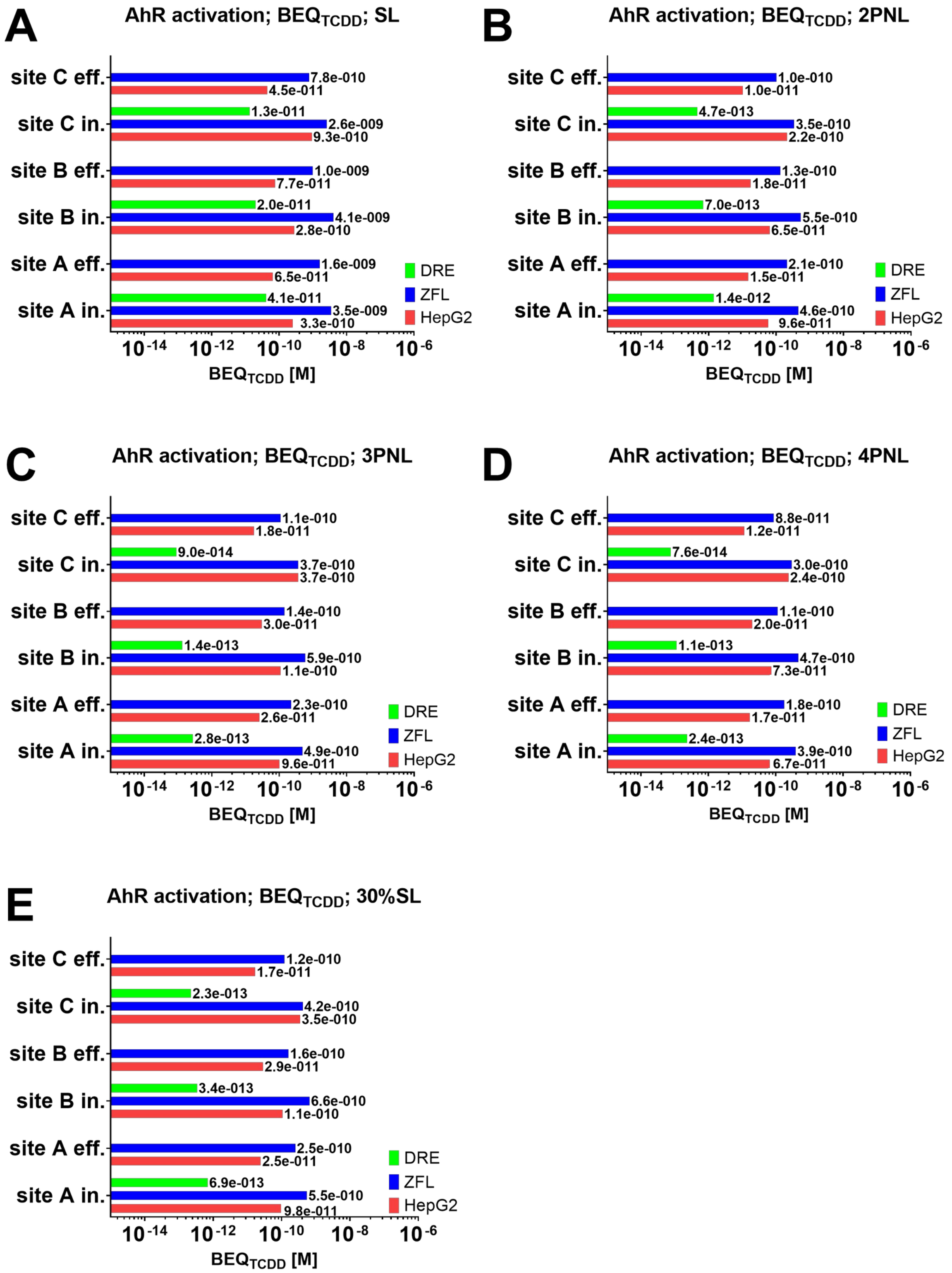

Fig. S38: Alternative data fitting (TCDD). Outline of derived TCDD-BEQ standardisations, regarding the used regression model (panel A: SL, panel B: 2PNL, panel C: 3PNL, panel D: 4PNL, and panel E: 30%SL, see details in section 3.4).

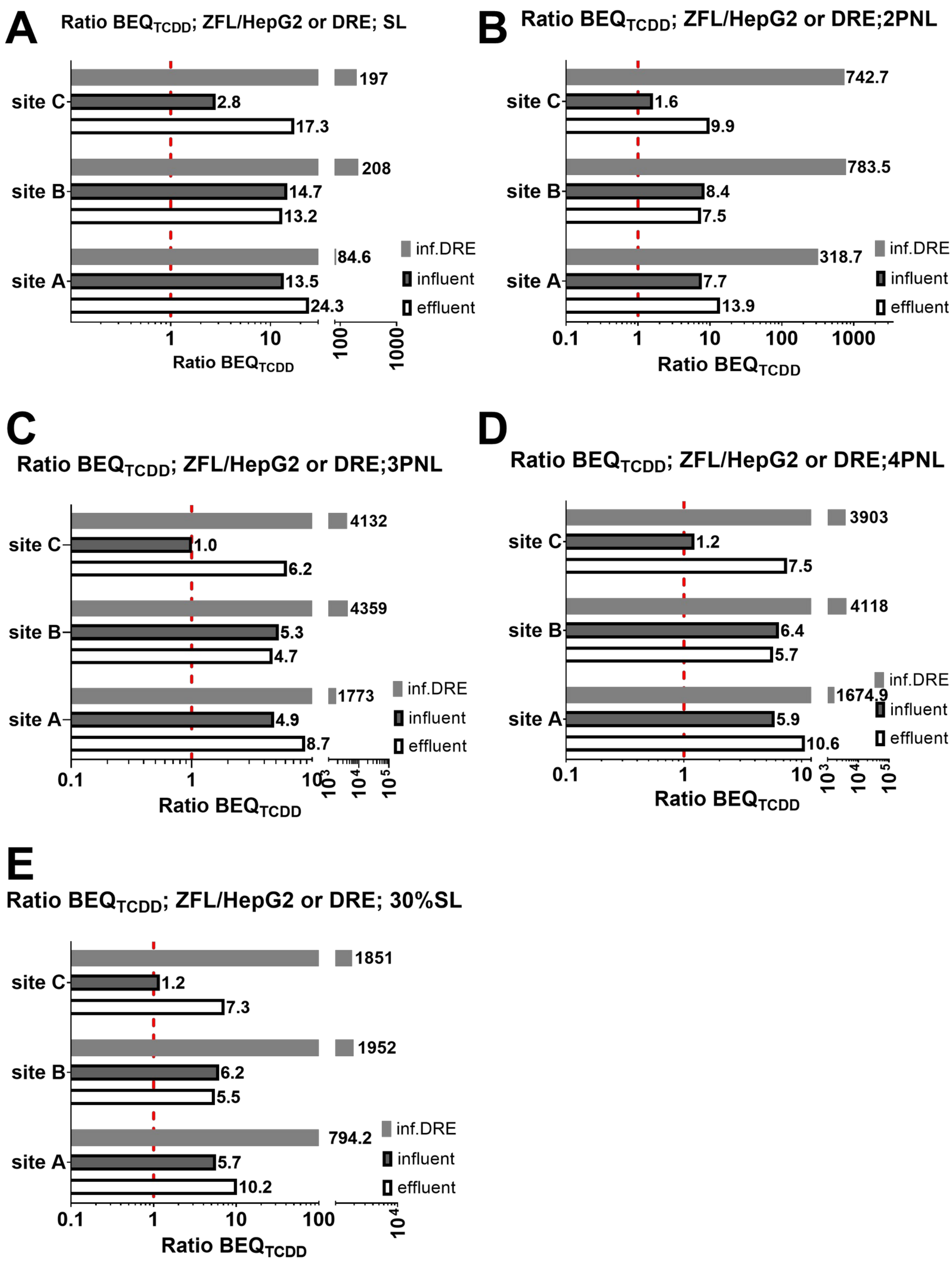

Fig. S39: Alternative data fitting (TCDD). Outline of derived zebrafish-to-mammal BEQ ratios, regarding the used regression model: SL (panel A), 2PNL (panel B), 3PNL (panel C), 4PNL (panel D), and 30%SL (panel E), see details in section 3.4. The unity ratio is illustrated as a dotted red line.

3.5 Supplementary results: Main article – other supplementary results

Tab. S5: Summary of all effect point estimates ($EC_{IR1.5}$), their variation metrics, calculated BEQ values,

and chemical removal efficiencies, as derived from the oxidative stress MoA assessment (Ctrl = reference control, inf = influent, eff = effluent, end = endpoint, hu = human, zf = zebrafish, SE = standard error, CIs = confidence intervals, Ch. = removal efficiency from chemical assessment data (SI12, tab. "REP_BEQ_calculations") given in parentheses). Effect concentration estimates computed beyond the empirically tested exposure concentrations, as well as consequential BEQ values and biological removal efficiency, are marked with a double-cross (‡).

Site	Sample	Spec	Cell line	$EC_{IR1.5}$ [REF] [M] Including SE	95% CIs [REF] [M]	BEQ_tBHQ [M]	BEQ_MZC [M]	Removal [%] (Ch.)
A	inf	hu	AREc32	1.35 ± 0.15	1.02;1.68	1.12E-06	3.43E-06	
A	eff	hu	AREc32	8.27 ± 1.45	5.08;11.45	1.83E-07	5.60E-07	84 (73)
A	inf	zf	ZF4	1.39 ± 0.10	1.18;1.61	4.45E-06	3.09E-06	
A	eff	zf	ZF4	11.25 ± 2.53	5.68;16.82	5.49E-07	3.82E-07	88 (73)
B	inf	hu	AREc32	1.54 ± 0.07	1.40;1.69	9.83E-07	3.00E-06	
B	eff	hu	AREc32	13.48 ± 4.00	4.70;22.28	1.12E-07	3.44E-07	89 (85)
B	inf	zf	ZF4	1.43 ± 0.14	1.13:1.73	4.34E-06	3.02E-06	
B	eff	zf	ZF4	13.82 ± 3.72	5.65;22.00	4.47E-07	3.11E-07	90 (85)
C	inf	hu	AREc32	1.62 ± 0.30	0.93;2.30	9.38E-07	2.87E-06	
C	eff	hu	AREc32	53.48 ± 48.32‡	-52.9;160	2.83E-08‡	8.66E-08‡	97‡ (92)
C	inf	zf	ZF4	1.23 ± 0.14	0.90;1.56	5.03E-06	3.50E-06	
C	eff	zf	ZF4	34.35 ± 21.59‡	-13.2;81.9	1.80E-07‡	1.25E-07‡	96‡ (92)
Ctrl	tBHQ	hu	AREc32	1.52E-06 ± 5.95E-08	1.39E-06; 1.64E-06			
Ctrl	tBHQ	zf	ZF4	6.18E-06 ± 5.55E-07	4.99E-06; 7.37E-06			
Ctrl	MZC	hu	AREc32	4.63E-06 ± 4.01E-07	3.78E-06; 5.48E-06			
Ctrl	MZC	zf	ZF4	4.30E-06 ± 6.12E-07	2.99E-06; 5.61E-06			

Tab. S6: Summary of all effect point estimates (EC10), calculated BEQ values, and chemical removal efficiencies, as derived from the xenobiotic metabolism MoA assessment (Ctrl = reference control, inf = influent, eff = effluent, end = endpoint, hu = human, zf = zebrafish, m = mouse, SE = standard error, CIs = confidence intervals, Ch. = removal efficiency from chemical assessment data (SI12, tab. "REP_BEQ_calculations") given in parentheses). Effect concentration estimates computed beyond the empirically tested exposure concentrations, as well as consequential BEQ values and biological removal efficiency, are marked with a double-cross (‡).

Site	Sample	Spec.	Cell line	EC_{10} [REF] [M]	95% CIs [REF] [M]	BEQ_TCDD	Removal

				Including SE		[M]	[%] (Ch.)
A	inf	hu	HepG2	1.06 ± 0.19	0.76; 1.72	1.01E-10	
A	eff	hu	HepG2	4.13 ± 0.45‡	3.14; 5.11	2.59E-11‡	74‡ (73)
A	inf	zf	ZFL	0.75 ± 0.08	0.57; 0.93	4.90E-10	
A	eff	zf	ZFL	1.62 ± 0.23	1.11; 2.14	2.26E-10	54 (73)
A	inf	m	DRE	0.61 ± 0.07	0.46; 0.76	2.77E-13	
B	inf	hu	HepG2	0.96 ± 0.13	0.68; 1.24	1.11E-10	
B	eff	hu	HepG2	3.53 ± 0.73‡	1.91; 5.15	3.03E-11‡	73‡ (85)
B	inf	zf	ZFL	0.62 ± 0.05	0.52; 0.72	5.88E-10	
B	eff	zf	ZFL	2.57 ± 0.28‡	1.95; 3.19	1.43E-10‡	76‡ (85)
B	inf	m	DRE	1.25 ± 0.12	1.00; 1.51	1.35E-13	
C	inf	hu	HepG2	0.29 ± 0.03	0.22; 0.36	3.67E-10	
C	eff	hu	HepG2	6.01 ± 1.61‡	2.48; 9.54	1.78E-11‡	95‡ (92)
C	inf	zf	ZFL	0.99 ± 0.11	0.75; 1.22	3.71E-10	
C	eff	zf	ZFL	3.33 ± 0.79‡	1.59; 5.06	1.10E-10‡	70‡ (92)
C	inf	m	DRE	1.88 ± 0.31	1.23; 2.52	8.99E-14	
Ctrl	TCDD	hu	HepG2	1.07E-10 ± 4.43E-11	1.52E-11; 1.99E-10		
Ctrl	TCDD	zf	ZFL	3.66E-10 ± 8.45E-11	1.91E-10; 5.42E-10		
Ctrl	TCDDl	m	DRE	1.69E-13 ± 1.06E-13	4.58E-14; 3.83E-13		

Tab. S7: EC50 estimates computed herein vs. reported EC50 estimates within the literature for identical assays or reporter constructs.

	EC_{50} measured [M] ± SE	EC_{50} reported [M] ± SE	Reference	Ratio
HepG2	5.77E-10 ± 1.10E-10	2.2E-10 ± 3E-11	[15]	2.64
ZFL	3.08E-09 ± 3.21E-10	1.2E-09 ± 4.7E-12	[12]	2.58
DRE	7.58E-12 ± 4.36E-12	2.8E-12 (SE not given)	[17]	2.71

Tab. S8: Percentage [%] of BEQ_Bio explained by BEQ_Chem, as derived from the iceberg modelling approach (see SI12, tab. "REP_BEQ_calculations"), regarding the utilised regression model (see section 3.4 herein).

Reg. Mod.	Cell line	site A inf.	site A eff.	site B inf.	site B eff.	site C inf.	site C eff.

SL	HepG2	0.0017	0.0038	0.0022	0.0040	0.0011	0.0067
	ZFL	0.0001	0.0002	0.0001	0.0003	0.0004	0.0004
	DRE	0.0109		0.0309		0.0752	
2PNL	HepG2	0.008	0.017	0.009	0.017	0.005	0.029
	ZFL	0.001	0.001	0.001	0.002	0.003	0.003
	DRE	0.310		0.881		2.142	
**3PNL**	HepG2	0.004	0.010	0.006	0.010	0.003	0.017
	ZFL	0.001	0.001	0.001	0.002	0.003	0.003
	DRE	1.610		4.571		11.121	
4PNL	HepG2	0.007	0.015	0.008	0.015	0.004	0.026
	ZFL	0.001	0.001	0.001	0.003	0.003	0.003
	DRE	1.894		5.377		13.084	
30%SL	HepG2	0.005	0.010	0.006	0.011	0.003	0.018
	ZFL	0.001	0.001	0.001	0.002	0.002	0.002
	DRE	0.642		1.822		4.433	

Tab. S9: Summary of all tested ISEDA compounds' concentration estimates (AC50 and EC10), including their variation metrics. Effect concentration estimates computed beyond the empirically tested exposure concentrations are marked with a double-cross (‡). Non-computable estimates and respective variation metrics are marked with "na" (not applicable).

AC_{50}			
ISEDA-identified compound	Reporter cell line	AC_{50} [M]	SE [M]
Daidzein (DAI)	HepG2	9.19E-06	±4.51E-06
	DRE	3.69E-06	±1.19E-06
	ZFL	na	na
Climbazole (CLB)	HepG2	3.70E-05	±1.85E-05
	DRE	2.93E-06	±2.08E-06
	ZFL	na	na
Thiabendazole (TBDZ)	HepG2	1.01E-06	±8.23E-07
	DRE	5.87E-06	±2.11E-06
	ZFL	na	na

EC_{10}			
ISEDA-identified compound	Reporter cell line	EC_{10} [M]	SE [M]
Daidzein (DAI)	HepG2	2.39E-05	±3.76E-06
	DRE	4.09E-06	±9.20E-07
	ZFL	na	na
Climbazole (CLB)	HepG2	1.59E-05	±1.09E-06
	DRE	5.75E-06	±3.62E-06
	ZFL	na	na
Thiabendazole (TBDZ)	HepG2	2.14E-04‡	±2.61E-04‡
	DRE	1.62E-06	±4.18E-07
	ZFL	na	na

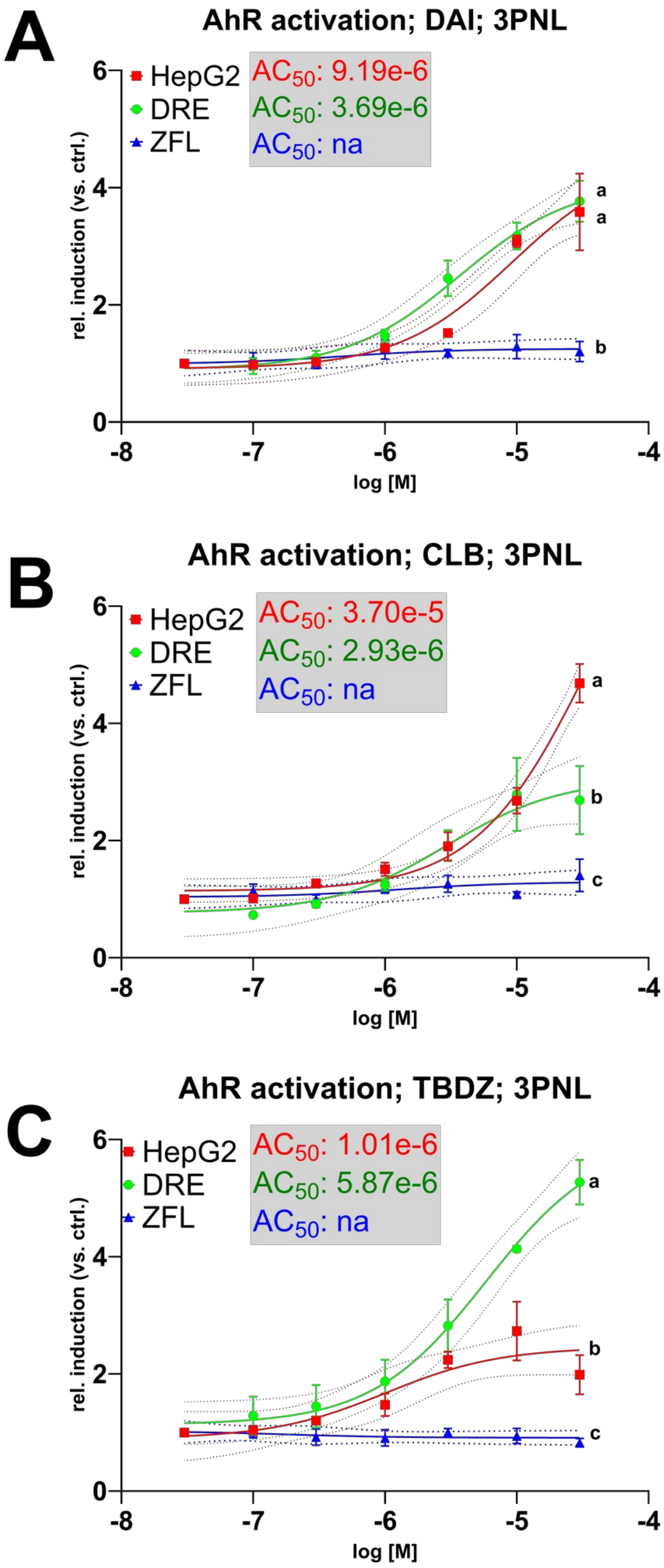

Fig. S40: Alternative data fitting (ISEDA). Activation of the cellular xenobiotic metabolism (AhR-ARNT-mediated toxicity pathway), depicted as relative induction in relation to the solvent control, in human (HepG2, red), zebrafish (ZFL, blue), and mouse (DRE, green) reporter gene assays exposed for 24 h to the ISEDA-identified compounds daidzein (DAI, panel A), climbazole (CLB, panel B), and thiabendazole (TBDZ, panel C). Points indicate the experimental mean (experimental units n = 3-5, observational units N = 9-15), with the SEM marked by whiskers. Samples were analysed in a logarithmic dilution series with 30 µM as the highest concentration. CRCs were fitted using non-linear, log-log regressions (model "3PNL"; see SM, section 3.4) and are shown in their respective colours, with 95% CIs as faded, dotted lines. Effect point estimates (AC_{50}; coloured, vertical, and dotted lines) were derived from the concentrations causing 50% of the response. The respective values are depicted in the graphs. A two-way ANOVA, followed by Tukey's test for multiple comparisons, was conducted to compute differences between effect sizes of the respective reporter line comparisons (alternating lowercase letters indicate statistical significance, $p < 0.05$). The respective samples' cellular viability/cytotoxicity data are given in the SM, fig. S30. The raw data and metadata are provided in SI15.

3.6 Supplementary results: Main article – Bioavailability assessment

To evaluate the potential impact of bioavailability differences within the *in vitro* test systems, the UFZ-LSER QSPR tool [45] was applied, employing mass-balance equation models to estimate free cellular concentrations [46]–[48]. DMEM was chosen as the standard culture medium input parameter because all cultures rely on DMEM or a variant thereof, and mass-balance parameters are not defined for more specific cultivation media. Similarly, HepG2 was chosen as the sole cellular parameter in the mass-balance equation, given that none of the other cell lines used in this study has established parameters. As such, only the FBS amount and cellular concentrations (at seeding) were accounted for in the model. From previous experience, these parameters describe most of the compartmentalisation, and specific differences introduced by the utilised cellular systems are relatively minuscule [46], [59].

First, the reference compounds tBHQ and MZC were assessed at a nominal concentration of 3 µM, close to the computed $EC_{IR1.5}$ estimates. Because the same amount of FBS was used in both the ZF4 and AREc32 assays and the number of cells was comparable, we also calculated free cellular concentrations, which were nearly identical (fig. S41A).

Second, free cellular concentrations of the reference compound TCDD and the three identified ISEDA compounds (CLB, DAI, and TBDZ) were computed for the ZFL, HepG2, and DRE bioassays. A fixed nominal concentration of 30 nM was chosen as a standard of comparison, and the nominal EC/AC_{50}s were transformed into free EC/AC_{50}s. For the fixed concentration, free cellular concentrations were consistent across the ZFL and DRE assays, whereas the HepG2 assay showed lower concentrations, reflecting its higher FBS content (fig. 41B).

Finally, further comparison of TCDD-relative effect potency (REP) values of the ISEDA compounds between nominal and free cellular concentrations was performed for the HepG2 and DRE assays (fig. 41C). ZFL-specific calculations were also derived but are not presented within the articles due to the instability of the point estimates (see fig. S40, or fig. 8 in the main article). The latter values and all raw data are given in SI13. The comparison (fig. S41D) indicated negligible differences in TCCD-REP nominal or free cellular concentration ratios between HepG2 and DRE, suggesting that bioavailability differences were minuscule and unlikely to account for the observed discrepancies in activation, reinforcing the conclusion that the observed effect differences are predominantly mechanistic in nature.

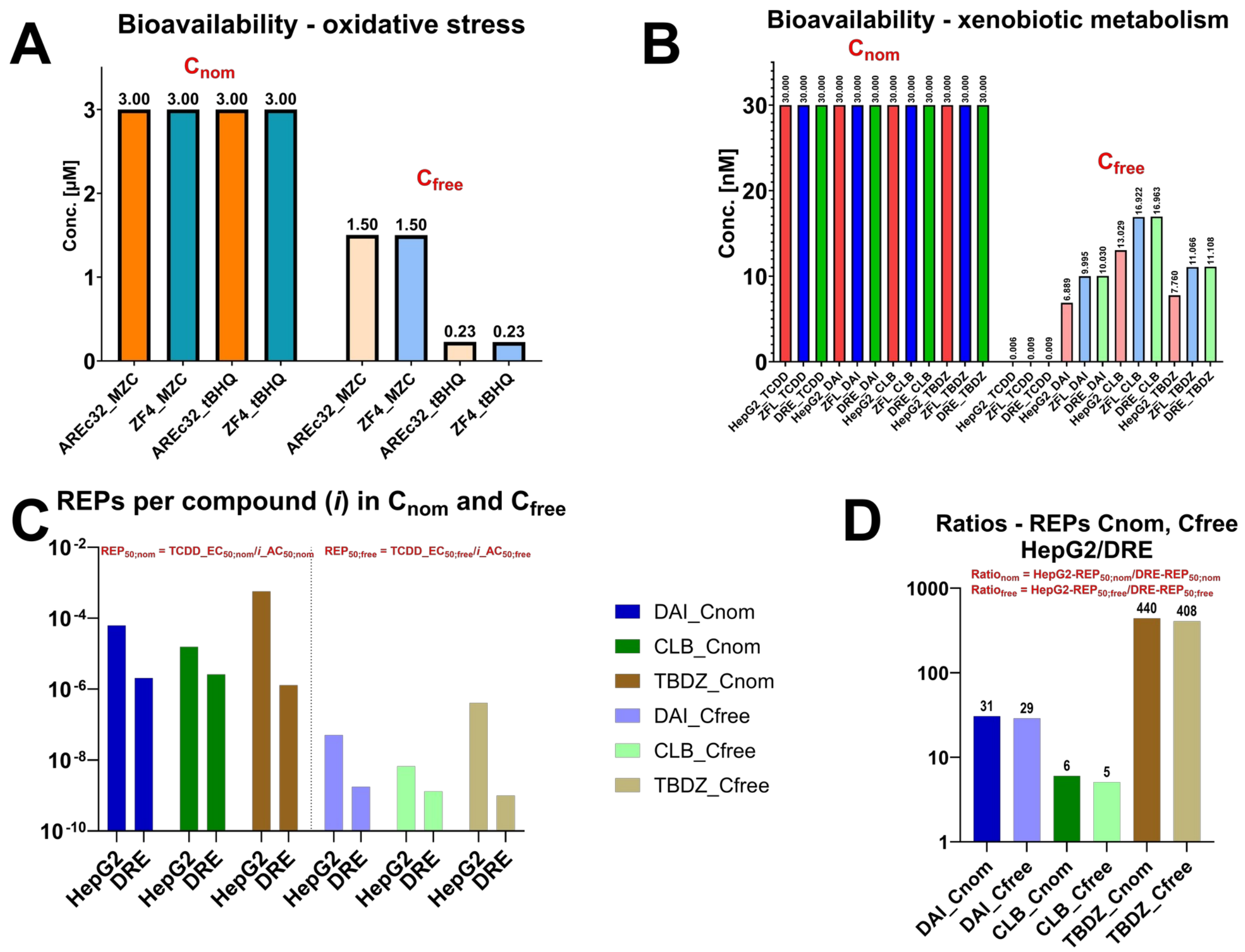

Fig. S41: Outline of the bioavailability assessment regarding exposure concentrations and derived effect point estimates. Free cellular concentrations (C_{free}) were derived from nominal concentrations (C_{nom}) utilising the mass-balance models in the UFZ-LSER QSPR tool. Panel A: bioavailability assessment for the oxidative stress MoA exposure concentrations. Panel B: bioavailability assessment for the xenobiotic metabolism MoA exposure concentrations. Panel C: relative effect potencies (REPs) derived from nominal and free cellular concentrations. Panel D: potency ratios between HepG2 and DRE-derived nominal and free cellular concentration estimates. Abbreviations: tert-Butylhydroquinone (tBHQ), metazachlor (MZC), 2,3,7,8-tetrachlorodibenzodioxin (TCDD), daidzein (DAI), climbazole (CLB), and thiabendazole (TBDZ). Additional data are given in SI13 and SI16.

4. Supplementary discussion

4.1 The Nrf2-Keap1-ARE toxicity pathway – Canonical reactive cysteine residues and mechanistic insights on the “cysteine code”

4.1.1 Background and objectives of this project concerning Keap1

Keap1 (kelch-like ECH-associated protein 1) is an oxidative stress sensor protein. Electrophiles react with the cysteine residues of this protein, most prominently Cys151, 226, 273, 288, 613, 622, and 624, triggering a conformational change and the release of the antioxidant transcription factor Nrf2 (nuclear factor erythroid-derived 2-like), which regulates the cellular response to oxidative stress. In zebrafish, there are two paralogs of the protein – zfKeap1a and zfKeap1b with slightly different functionality [60].

Also, the canonical cysteine residues are alternatively distributed between the two paralogs; 1a is missing Cys273, 622, and 624, whereas 1b lacks Cys288 (beyond other non-canonical cysteines). See also fig. S42 below for a comparison of conserved cysteine residues among the investigated zebrafish (zfKeap1a, zfKeap1b) and human (hKeap1) sensor variants.

The interaction of Keap1 with two reference electrophiles, tert-butylhydroquinone (tBHQ) and metazachlor (MZC), was studied in both biological (*in vitro* reporter gene assays, main article) and computational (*in silico* molecular docking, “data in brief” co-submission, DIB_B) terms. While tBHQ interacts primarily with Cys151 [60], [61] and is thereby considered a Cys151-dependent activator, the reaction mechanism with MZC is unknown. Within the main article, tBHQ was initially chosen as a reference compound to derive bioequivalent values (BEQs) for environmental samples, given its previous utilisation in that role [57]. We decided on the environmental pollutant MZC as an alternative, as others have associated it with the induction of oxidative stress [62], and we have previously demonstrated its ability to induce the Nrf2-Keap1 pathway [7], [8].

The goals of docking the reference electrophilic compounds to different structures of human and zebrafish Keap1 were: (i) to gain insight into where binding might occur, (ii) to observe if the poses are near any cysteine residues, especially around canonical ones, e.g. Cys151, (iii) to compare the predicted poses in the human variant and two zebrafish paralogous variants, and (iv) to compare binding poses in crystallographic and AlphaFold-predicted structures.

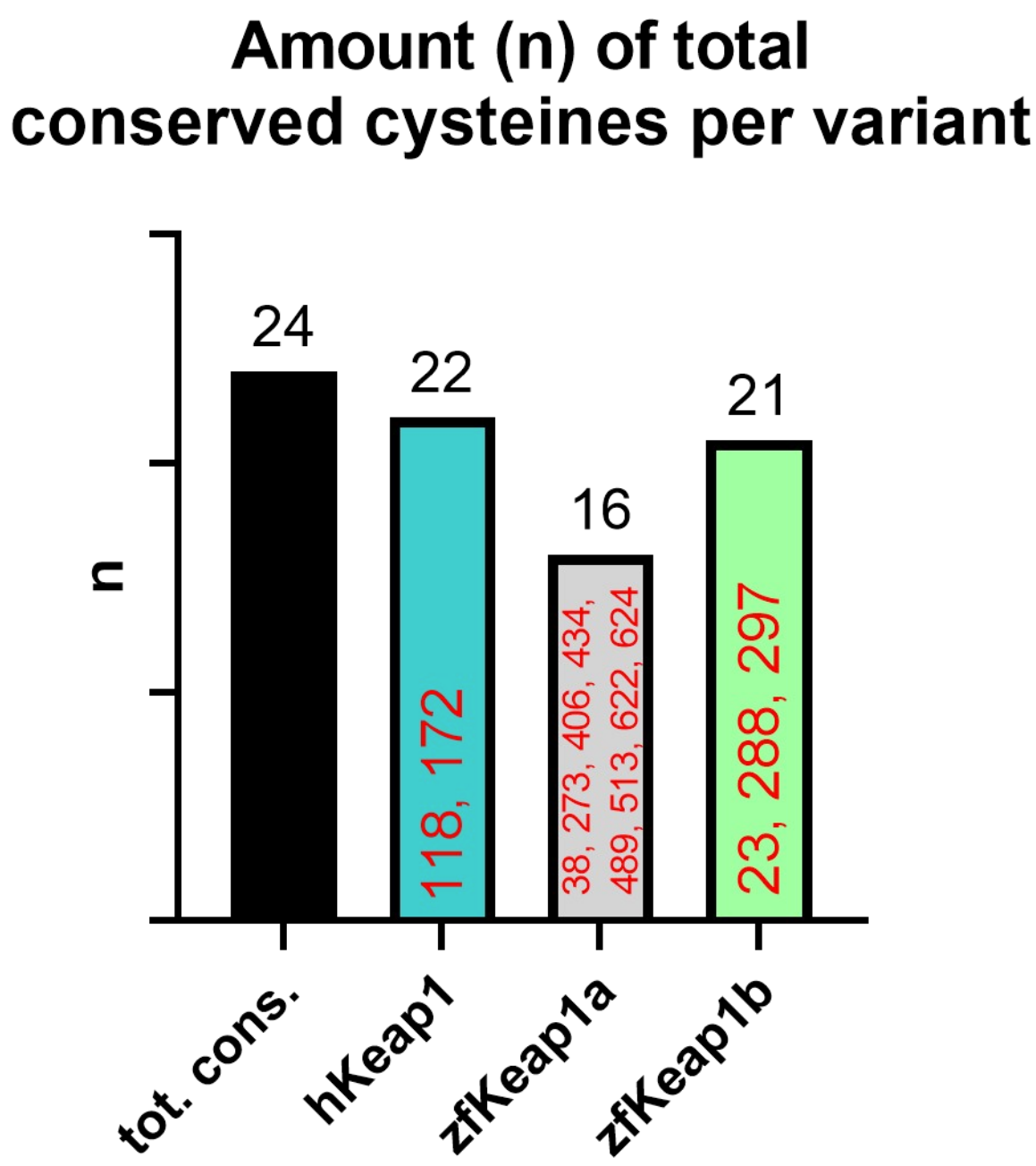

Fig. S42: Amount of total conserved cysteines within all Keap1 variants. The total number of conserved cysteine residues per variant is given on top of each bar. Missing conserved cysteines per variant are provided inside the bar, highlighted in red. Residue enumeration corresponds to the hKeap1 AA sequence (see DIB_B, fig. 6).

4.1.2 The Nrf2-Keap1-ARE signalling pathway: nomenclature and mechanisms

The Nrf2-Keap1-ARE signalling pathway is one principal regulator of oxidative stress detoxification, metabolism, and induction of related phase-II enzymes (e.g., glutathione S-transferase) [4], [63], [64]. Further, it plays a principal role in lipid, carbohydrate, nucleotide, and AA metabolism (reviewed in [65]). Crosstalk and reciprocal regulation between ARE-Nrf2-Keap1 and other central signalling cascades, such as MAPK/ERK, etc., have been reported [66]–[69].

The antioxidant-responsive element (ARE) sequence [70], [71] is found in the regulatory region of the mentioned phase-II metabolising enzymes, often coinciding with the xenobiotic response element (XRE) of the AhR-ARNT signalling pathway [72].

The nuclear factor erythroid-derived 2-like (Nrf2) is a basic region leucine zipper (bZip)-type transcription factor of the "Cap'n'Collar" (CNC) family that binds to the ARE and transactivates expression of related genes [63], [64], [73]–[75]. Nrf2 occurs ubiquitously in the cytosol under normal physiological conditions, bound to its negative regulator, Kelch-like ECH-associated protein 1 (Keap1).

Keap1, an actin-bound protein, serves as an adaptor for the Cullin 3-based E3-ligase ubiquitin complex (CUL-E3) and thereby marks Nrf2 for further ubiquitination and subsequent degradation [73]. Via the broad complex, tramtrack, and bric a brac (BTB) domain, Keap1 homodimerises with itself and also binds to CUL-E3 (see fig. 4 in DIB_B and fig. S3 for domain mapping). Nrf2 attachment is modulated via the Kelch domain (fig. 3 in DIB_B) [76], [77]. One of each Keap1 homodimers' Kelch domains anchors to DLG and ETGF motifs within Nrf2 (reviewed in [65], [78]). The Nrf2-Keap1 heteromer complex is described by a so-called "hinge-and-latch" apparatus, in which the DLG/Kelch interaction represents the latch and the ETGF/Kelch interaction the hinge. Thereby, the binding affinity of the latter complex is approximately two orders of magnitude higher [77].

Turnover and half-life of Nrf2 are short-term under unstressed conditions [79]. However, upon exposure to oxidative stress, Nrf2 is released from Keap1, thereby avoiding proteolysis. It translocates and accumulates within the nucleus, initiating gene expression [80].

Electrophilic interactions mediate Nrf2 release. As a common attribute, electrophiles can covalently modify sulfhydryl groups by alkylation, oxidation, or reduction [81], [82]. As such, 22 cysteine residues can be identified within the hKeap1 amino acid backbone. Some are more susceptible to reaction (Cys151, 226, 273, 288, 613, 622, and 624) and can be termed "canonical cysteines" [82]–[84]. Thus, certain classes of compounds seem to prefer interactions with specific cysteine residues and can be

categorised accordingly [60]. However, the overall recruitment of a cysteine pattern appears unique for every compound, a phenomenon termed “cysteine code” (reviewed in [85]).

In vivo and *in vitro* analyses revealed Cys151, 226, and 288 as the primary sensors due to increased reactivity (reviewed in [78]). Especially Cys151 is considered highly reactive, given that nearby basic residues (H129, K131, K150, and H154) lower its pKa, rendering it a thiolate anion even at physiological pH. Further research has shown that certain compounds are Cys151-preferable inducers, whereas others are Cys151-independent [61]. As such, common inducers tBHQ and sulforaphane are Cys151-preferable, implying that a deletion/mutation of Cys151 still renders Keap1 activation by these compounds, just to a lesser extent. Hence, the Nrf2-Keap1 interplay cannot be reduced to a single cysteine interaction but depends on multiple sensors (“cysteine-code”).

Cys151 is located in the previously mentioned BTB domain, which coordinates the interaction with the CUL-E3. Cys273 and Cys288 are situated within the intervening region (IVR) domain, which links BTB to Kelch. The geometry of the polypeptide chain changes when electrophiles covalently bind to these cysteine residues. Regarding location, interaction with CUL-E3 is prevented, or Kelch dissociation is mediated by the respective DLG and ETGF motifs, leading to Nrf2 release or, at least, Keap1 inactivation. More recent studies suggest a cycling mechanism (discussed in [78]). Per se, Nrf2 is not released from Keap1 once bound, even upon conformational changes within Keap1 due to electrophilic interactions [86]. Moreover, ETGF/Kelch interactions remain persistent, prevalent Keap1 is only rendered non-functional, and the oxidative stress response is mediated by *de-novo* synthesised Nrf2. Additionally, the latest results indicate an additional role of protein-protein interaction inhibitors, such as p62, regarding the hinge and latch mechanisms. Apparently, DLG/Kelch latch dissociation is also dependent on p62-mediated Nrf2 activation and not solely on electrophilic interactions [87].

The Nrf2-Keap1-ARE signalling pathway is highly conserved within vertebrates and beyond [75], [88], [89]. However, structural differences occur in fish due to the teleost genome duplication [42]. For both Nrf2 and Keap1, duplicated co-orthologs (Nrf2a/Nrf2b and Keap1a/Keap1b, respectively) were identified in zebrafish [90], [91]. Beyond duplication, subfunction partitioning occurred with differences in expression and spatiotemporal characteristics [91]. Structural differences were reported, with Nrf2b lacking certain transactivation domains and Keap1a and Keap1b being Cys273- or Cys288-deficient (beyond other cysteine residues). Nevertheless, both variants retain functionality [88]. In more detail, studies have pointed out that zfKeap1b is an authentic ortholog of mammalian Keap1 [92]. As such, Cys151-preferable compounds show minor mitigation of the oxidative stress response for zfKeap1a compared to zfKeap1b.

4.1.3 Molecular docking results in the context of the "cysteine code" (see DIB_B for results)

We concluded from the molecular docking analyses that tBHQ showed qualitatively similar affinity patterns and binding energies between human and zebrafish Keap1 variants regarding blind docking (DIB_B, section 3.1.1). Notably, there were minuscule interaction differences, with zfKeap1a preferring binding to the IVR (Cys226) and zfKeap1b to the Kelch domain (Cys368/513). Site-directed docking to the Cys151 proximity revealed cognisable binding energy differences for tBHQ between variants with the following hierarchy: hKeap1 > zfKeap1b > zfKeap1a.

For MZC (DIB_B, section 3.1.2), we found comparable best-energy binding poses across all three variants within the Kelch domain (Cys368/513). However, for intermediate- to low-energy binding poses, MZC affinities diverged across specific variants. As such, MZC preferred binding to Cys151 in hKeap1, Cys613 in zfKeap1a, and Cys226 in zfKeap1b. Site-directed docking to the Cys151 proximity revealed the same binding energy hierarchy as for tBHQ: hKeap1 > zfKeap1b > zfKeap1a.

Comparison of AA sequence homology between variants solidifies the above observations (DIB_B, section 3.1.1.6, figs. 6 and 7), as hKeap1 and zfKeap1b share higher overall sequence homology (73%), especially in the vicinity of Cys151 (90%). Nevertheless, substituting only 3 AA residues between hKeap1 and zfKeap1b within the Cys151 proximity leads to a binding energy reduction of 0.7 Δ kcal/mol. As such, sequence homology coincides with the encountered Cys151 binding energy hierarchy: hKeap1 > zfKeap1b > zfKeap1a.

The biological data presented in this project, the literature, and the molecular docking results support the concept that tBHQ is a Cys151-dependent inducer. According to the docking data, the primary interactions occur at Cys226, 368, and 513. However, Cys151 recruitment is necessary for complete engagement ("cysteine code"). Because zebrafish variants show lower susceptibility to tBHQ in the Cys151 vicinity, this reduces the cellular stress response. For tBHQ, the cysteine code seems to be conserved across variants.

Conversely, MZC is likely a Cys151-independent inducer that interacts via the Kelch domain (Cys368/513) in all variants. Differences in Cys151 interaction do not impact the measured *in vitro* endpoint. Interestingly, however, beyond the Kelch domain, MZC preferentially interacts with different cysteine residues in the observed Keap1 variants. The latter might indicate a diversion in functionality and susceptibility within the zebrafish variants to compensate for missing cysteines (compared to the human AA sequence).

4.2 Supplementary discussion: Comment on zfKeap1a/b functional divergence and structural variability

Multiple sequence alignments (see DIB_B, fig. 7) resolved 55% and 90% homology for zfKeap1a and zfKeap1b, respectively, to hKeap1 near cysteine 151 (amino acid residues 125-156). Despite minimal amino acid differences for zfKeap1b, tertiary structural changes affect tBHQ affinity at this site. In line with these results, studies have pointed out that zfKeap1b is an authentic ortholog of mammalian Keap1 [92]. As such, cysteine 151-preferable ligands show minor mitigation of the oxidative stress response for zfKeap1a compared to zfKeap1b. Nevertheless, both variants retain functionality [88]. Structural differences within fish variants occur due to the teleost genome duplication [42], resulting in duplicated co-orthologs (Nrf2a/Nrf2b and Keap1a/Keap1b) in zebrafish [90], [91]. Structural differences were reported: zfNrf2b lacks certain transactivation domains, whereas zfKeap1a and zfKeap1b are deficient in specific cysteine residues (see fig. S42). However, in our experimental setup, it remains unclear how signal transduction is sequestered among different zebrafish Nrf2 and Keap1 variants, as we can only measure the final interaction with the ARE regulatory unit. The extent to which stochastic processes between zebrafish Nrf2 and Keap1 variants impact transactivation in a cysteine 151-dependent manner remains enigmatic and warrants further investigation.

4.3 Supplementary discussion: “CALUX” assay nomenclature and identification

As the main article's discussion suggests, we want to highlight a significant gap in methodological reporting in the literature, particularly regarding the AhR/DRE CALUX reporter bioassays. Iterations of this assay arise from different generations of the reporter construct (pGudLuc1 to 7.5 [13], [93]–[95]) and their transfection into diverse species' cell lines [15], [96], [97], leading to numerous AhR/DRE CALUX clones with varied sensitivities to TCDD [15]. Eichbaum et al. [98] reviewed the use of AhR-responsive reporter bioassays for detecting DLCs and noted difficulties in identifying specific clones used in studies. Consistent with our findings, we recommend explicitly specifying the cell line and clone used when referencing the AhR/DRE CALUX-type assay. Noteworthy are the complications associated with extrapolating AhR-mediated effects across different species using reporter assays, which have been noted since their inception in environmental testing [99].

4.4 Supplementary discussion: Cross-species assessment in context with regulatory metrics – BEQs and EBTs

In the main article’s discussion, we elaborate on the complexities involved in BEQ normalisation, particularly how it skews cross-species comparisons. This issue becomes apparent with TCDD-BEQ normalisation, due to species- and ligand-specific modulation of the AhR-ARNT-XRE pathway. To address these concerns, we conducted a thought experiment (fig. S43) comparing all our derived

TCDD-BEQ values from different species reporter assays with published interim tier 1 effect-based trigger (EBT) values.

Rat TCDD-BEQs ("H4L7.5c2" in fig. S43) were computed for comparison. The AhR CALUX-type bioassay clone H4L7.5c2 is based on the rat hepatocellular carcinoma (H4-II-E, CVCL_0284) cell line, and is often employed in the bioanalytical assessment of environmental samples. Given that the human HepG2-based CALUX-type assay clone (HG2L7.5c1) can be easily confused with the rat clone (sometimes clone definitions are even completely omitted in the literature; only "CALUX" is mentioned) and that the two clones differ by one order of magnitude in terms of responsiveness to TCDD potency [15] we wanted to depict potential implications on the derivation of safety levels. Rat TCDD-BEQs were computed from the geometric means of our data. Given the variability in our biological estimates and the H4L7.5c2 CALUX-type assay's responsiveness to TCDD, we deemed this approach a feasible compromise. The EC_{50} value for H4L7.5c2 was derived from the literature [15], while the EC_{10} was computed assuming a Hill slope of one.

Further, we consulted interim EBTs, as presented and discussed in Neale et al. [100], [101] to assess our data from a hypothetical regulatory perspective. The ecological EBT-BEQ was initially derived from van der Oost et al. [102] and the human EBT-BEQ from the State of California water monitoring program [103]. The EBT-BEQs by Van der Oost were derived from *in vivo* exposure studies on the rare minnow (*Gobiocypris rarus*) and employ a species-sensitivity distribution approach. However, the source of the State of California's EBT-BEQs could not be found. These interim EBTs are intended to be broadly applicable (cross-species and -assay); however, their limitations are acknowledged. According to Carere et al. [104], these interim values constitute tier 1 EBTs. At the same time, previous work by fellow researchers and co-authors already demonstrated the need for assay-specific EBTs [105], and the hypothetical cross-species scenario presented here further illustrates why such values will be important for future regulatory applications, as broadly applied interim EBTs may shift interpretation depending on assay-specific reference-compound responsiveness rather than on environmental-sample bioactivity alone.

Our hypothetical, comparative scenario, as illustrated in fig. S43, indicates that mouse DRE assay-derived BEQs are generally lower than the ecological and human EBT-BEQ. Rat AhR CALUX-type-derived BEQs in effluent samples are below the human EBT-BEQ, while human HepG2 and zebrafish ZFL BEQs exceed these thresholds. Interestingly, this is not necessarily due to increased sensitivity of the bioassays to environmental samples, but rather to their lower sensitivity to TCDD compared with rodent models. This suggests that general, broad-application, interim EBTs of the AhR response can

lead to either overestimation or underestimation of effects, depending on the assessment perspective and assay used.

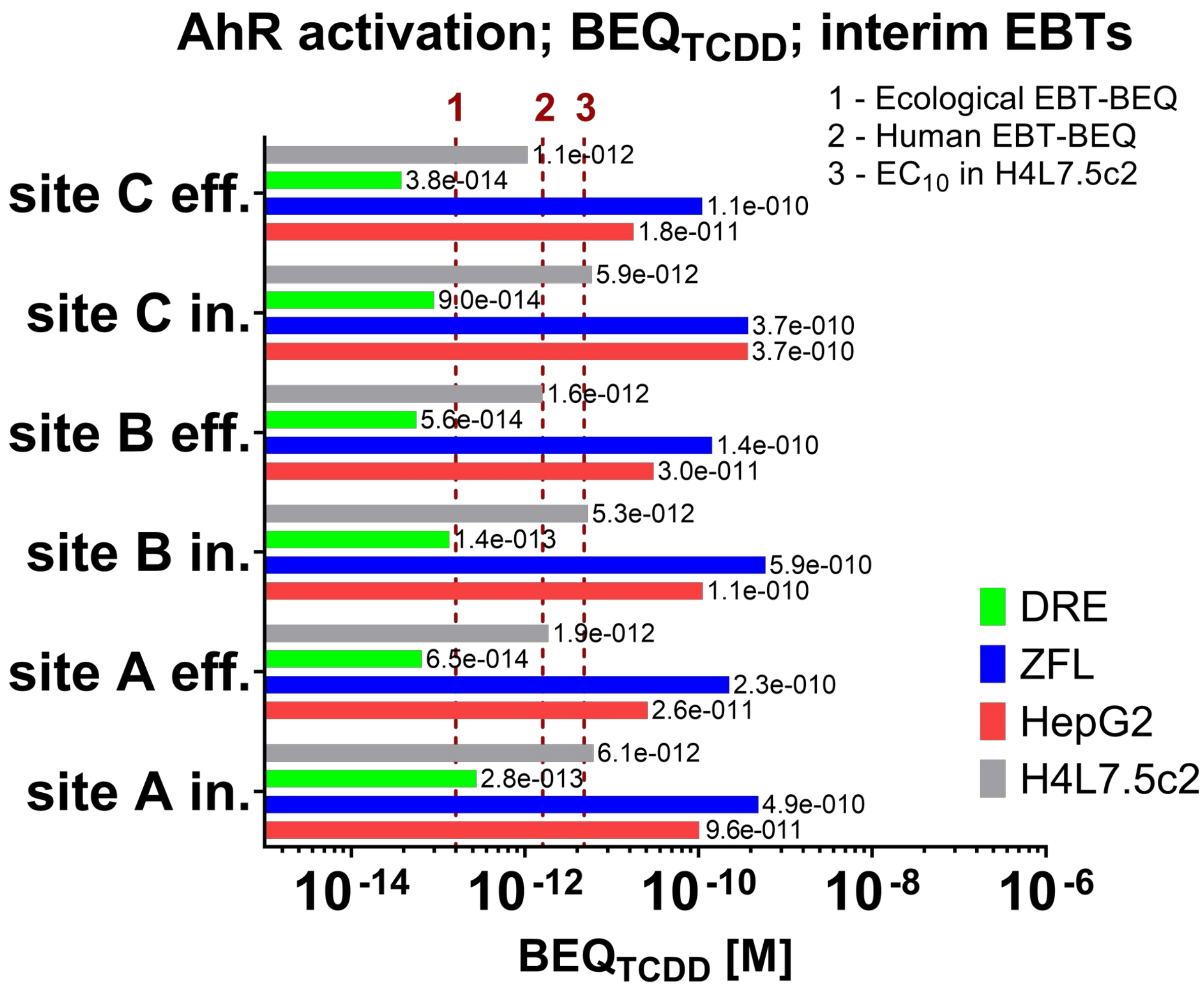

Fig. S43: Hypothetical TCDD-BEQ scenario derived from the cross-species bioanalytical assessment. Published tier 1/interim AhR EBT benchmark values are illustrated as dotted red lines for comparison; these values were not recalculated as assay-specific EBTs for each reporter assay.

4.5 Supplementary discussion: TCDD as a reference compound, reasoning and scrutiny

In their comprehensive research, Escher et al. [106], [107] delineated tier 2 EBTs, emphasising environmental quality standards (EQS) as a point of departure. These EQS inherently necessitate an inter-species sensitivity assessment. However, a limitation arises in their application: these tier 2 EBTs are explicitly defined only for the reference compound benzo[a]pyrene (BaP). Our choice of TCDD as the reference compound in this study is based on several factors. While PAHs are more commonly found internationally due to their origin in combustion processes, in our geographical context, DLCs appear more relevant. This significance is due to the legacy contamination from paper mills and trash incineration in Scandinavia [108], [109].

While analysing DLCs and PAHs might have been more effective for explaining a larger fraction of the biological response, we aimed for a broader chemical analysis targeting emerging pollutants. We regard this scenario as more realistic within a standard screening approach. Following the usual modus operandi, we employed TCDD as a reference compound. Regarding iceberg modelling and the subsequent ISEDA, our objectives were threefold: to assess the cross-species applicability of iceberg modelling, to identify critical drivers of AhR-ARNT-XRE pathway induction from the ISEDA, and to evaluate specific compounds across species, potentially revealing variations in sensitivity to these pollutants.

Moreover, DL-PCBs are known for their propensity to accumulate in the hydrosphere [110], [111]. Another crucial aspect is that TCDD is a more widely used reference compound, enabling the application of a standard modus operandi. The choice of TCDD was further influenced by the complexities of using BaP. The majority of PAHs require biotransformation to trigger AhR-mediated toxicity [112]–[114]. The extent and comparability of these biotransformation capacities in stable hepatocyte cell lines, like those used in our study, remain poorly documented and understood. We will address the issue of biotransformation in a section below (4.8).

When considering the environmental behaviour of these compound classes, both PAHs and DLCs exhibit poor water solubility [115]. The aqueous solubility of PAHs decreases logarithmically with increasing molecular mass, but the smaller, two- and three-ringed PAHs are more soluble [115]. In contrast, AhR-mediated toxicity is primarily associated with PAHs that have three or more aromatic rings [116]. TCDD and BaP have been shown to interact similarly with the AhR PASb domain [117], emphasising the need for a careful selection of reference compounds in environmental toxicology studies. Ultimately, the decision for the reference compound hinges on identifying the most potent inducer or the primary driver of the observed biological effect in the environment [50], [104].

However, how representative is AhR activation by TCDD or BaP of this specific MoA in an environmental water context? Both are unlikely contaminants in running water [118]–[120], whereas other classes of compounds are more likely to activate the AhR [121]. This activation acts more as a signal modulator, especially in fish with multiple AhR variants. As discussed in the main article, mixtures might elicit a general cellular stress response mediated by the AhR. The observed differences in BEQ values are primarily due to variability in the potency of the reference compound and to the BEQ calculation method. Many AhR-related effects in environmental mixture samples may be facilitated not only by receptor-mediated mechanisms but also by multidimensional pathway crosstalk.

Generally, for bioassays indicative of integrative effects (non-specific, reactive, and adaptive stress responses), known and identified chemicals explain only a small to intermediate fraction of the effect [49]. In contrast, for bioassays indicative of hormonal receptor-mediated effects, known and identified chemicals can more often explain a larger percentage of the effect [50], [122], [123], with some deviations observed between human and yeast-based assays . Contextually, the choice of the reference compound is critical, with TCDD and BaP being common choices due to their strong induction of AhR-related effects. Yet one must consider whether these are the most representative compounds of the MoA in aquatic contexts. Sediment, biota, and feed/food samples may be more suitable for targeting PAHs and DLCs due to the tendency of hydrophobic compounds to bind to organic matter, bioaccumulate, and biomagnify, as previously demonstrated by others [124]–[130]. In light of the results and discussion, it may be worthwhile to consider alternative reference compounds for assessing the MoA of xenobiotic metabolism in environmental water samples.

The BEQ concept (see also section 4.4 above) is based on toxic equivalency factors (TEFs) or toxic equivalents (TEQs) approaches [131]–[133]. TEFs index structurally related compounds to a well-studied reference compound based on shared characteristics, thereby hypothetically aggregating signals of a specific MoA. This approach requires meeting seven criteria: demonstrated need, defined chemical grouping, a broad toxicological database, endpoint consistency, additivity of response, a common MoA, and consensus [131]. This approach has recently been reevaluated for TCDD in the context of dioxin-like polychlorinated dibenzo-p-dioxins, polychlorinated dibenzofurans, and polychlorinated biphenyls [134]. Notably, this comparison was made between several fish species, using TCDD-TEFs derived from rainbow trout. Given the multidimensional modulation of AhR-related toxicity, it is questionable whether a single compound, such as TCDD or BaP, can meet these criteria. Similarly, van Duursen et al. [135] noted that the AHR-mediated risk for DL-PCBs in humans is likely overestimated in the current TEF concept when extrapolated from rodent data. Given that we aim to extend environmental monitoring to encompass the entire biosphere, this exercise becomes increasingly challenging.

4.6 Supplementary discussion: validity of molecular docking approach

An earlier cross-species molecular docking study [136] reported findings that differ from ours: mAhR and zfAhR2 were modelled at approximately -4 kcal/mol, and hAhR's affinity towards TCDD was estimated at only -2 kcal/mol (compared to approximately -10 kcal/mol in our study). However, Bisson *et al.*'s study was not corroborated by biological data, unlike ours, as elaborated in the main article. Moreover, as discussed by Tagliablue et al. [117], this earlier study had limitations, particularly its reliance on apo-template structures for homology modelling and its oversight to account for the ligand-induced fit effect on protein conformation.

In contrast, we utilised the most recent cryo-EM structure of the human PASb domain [36] for molecular docking. Additionally, recent advancements in computational tools, such as AlphaFold and AutoDock Vina, have significantly improved the accuracy of protein structure prediction and ligand docking [31], [137]. While not a substitute for experimental validation, these methods now serve as powerful complements to traditional techniques. The agreement between our docking results and biological data further supports this.

4.7 Supplementary discussion: AhR crosstalk and non-canonical pathways

As mentioned in the main article, AhR crosstalk and the induction of non-canonical pathways may be due to several reasons:

Firstly, the existence of multiple XRE regulatory units has been established [138]; some of which are located near genes involved in the oxidative stress response, such as NQO1. This suggests a complex interaction between the AhR and cellular stress defence pathways [139], [140], where AhR may regulate genes downstream of the Nrf2-Keap1-ARE pathway, necessitating coordinated activation [72], [141], [142] or, conversely, being influenced by these pathways [143], [144].

Secondly, various non-canonical pathways have been identified apart from the well-known ligand to AhR-ARNT-XRE pathway [5], [145]. In these pathways, AhR forms dimers with various translocators and transcriptional co-factors, leading to the activation of distinct downstream genes. Specific promiscuous ligands are known to activate these non-canonical pathways [146]. A microarray analysis revealed that only about one-third of the genes differentially expressed by AhR activation were associated with XRE binding, suggesting the involvement of mechanisms beyond the canonical pathway [147]. Unlike typical nuclear receptors, such as hormonal receptors, AhR uniquely responds to a wide range of structurally diverse chemicals, producing varied ligand-selective toxicological and biological effects through multiple AhR-dependent mechanisms.

The non-canonical involvement of AhR can manifest at various levels of pathway regulation, including the ligand-binding domain (LBD), sequences adjacent to the LBD that potentially influence PAS-PAS motif interactions, variations in signal transduction through non-canonical translocators and co-activators, and differential interactions with varying genomic response elements [148], [149].

4.8 Supplementary discussion: outlook and further technicalities regarding multi-species reporter gene assays

From the synopsis of the main article's discussion, we provide an outlook and propose further implementation steps to enhance the regulatory acceptance of reporter bioassays as a standard tool

in environmental monitoring (fig. S44). While multi-species reporter bioassays boast accessibility, technical know-how, and scalability [150], other fields still need improvement.

From a short- to mid-term perspective, improving technical aspects of bioavailability and species sensitivity in receptor-ligand interaction modelling is most promising. As elaborated in this study, the computation of actual bioavailable concentrations in *in vitro* test systems via mass-balance equations [47], [48] has been shown to be accurate [46], [59], at least for certain compound classes, and is now supported by tools like the UFZ-LSER [45]. Based on our experience, an empirical assessment of non-standard reporter assays and cell culture medium compositions, in the context of their partition coefficient parameters, would be highly beneficial for making these applications more robust and less variable across multiple species.

Additionally, molecular docking has demonstrated good accuracy [43], [151] in reproducing experimental ligand-binding poses, such as the recently revealed hAhR cryo-EM structure [36], as well as in predicting AlphaFold2-predicted structures [137]. Extending the state of the science to environmentally or technically relevant species, such as trout, zebrafish, mice, and rats, would further enrich our understanding.

Long-term challenges include addressing limited biotransformation capacities in *in vitro* systems, a persistent issue in *in vitro* toxicology [152], [153]. As such, all employed hepatocyte cell lines, including ZFL [154], [155], HepG2 [156], [157], and Hepa-1c1c7/DRE [158], [159], have been reported to preserve at least some biotransformation capacity, either directly or indirectly. On the other hand, we are unaware of any retained biotransformation capacities in the cell lines used for the oxidative stress MoA, ZF4, and MCF-7. As such, this aligns with general accounts indicating that non-hepatic, stable cell lines completely lose biotransformation capabilities over prolonged culture.

Indeed, the observed variance in induction patterns for the xenobiotic metabolism MoA could be attributed to differences in biotransformation capacity. Understanding these disparities is crucial, as they can significantly affect induction patterns and activity. Regrettably, there is no universally accepted standard for comparing biotransformation capacities across different cell lines [160], [161]. Furthermore, many stable hepatocyte-like cell lines have not been evaluated in this context. Unfortunately, conducting a thorough assessment of the biotransformation capacities of the cell lines utilised in this study would have exceeded its scope and necessitated a dedicated investigation. However, we emphasise the importance of establishing standardised parameters for such assessments, particularly for future cross-species evaluations of hepatocyte-like cultures.

Another aspect we discussed throughout the article and supplementary material is signal transduction, especially in fish, with a focus on non-specific toxicity pathways. Primarily due to

genome duplication events [42], multiple transcription factors, translocators, co-factor isoforms, and the resulting stochastic sequestration of the ligand, the details of ligand-to-signal modulation remain an enigma. Potentially, CRISPR-mediated knockout studies could provide insights into these intricate mechanisms.

Finally, the aspect of the chimeric reporter gene assays. Fundamentally, none of the commonly deployed reporter gene bioassays can be purely defined as a single-species assay, since all reporter constructs contain chimeric elements. Typically, reporter gene design employs one of two strategies. The first utilises the cell's endogenous signal transduction, transcription, and translation machinery, introducing only a foreign reporter construct. The second approach involves using cell lines deficient in specific components of these machineries, which are subsequently supplemented (e.g., [162]–[164]). Regardless of the strategy, the resulting reporter lines are chimeric.

All reporter gene assays in this study leverage the cells' innate signal transduction, transcription, and translation mechanisms. However, the interaction between the transcription factor complex and the reporter gene construct is facilitated by chimeric gene-regulatory elements. Specifically, the pGudLuc7.5 reporter construct [13], [95] is regulated by 20 XRE sequences from the murine CYP1A1 gene inserted into a modified viral MMTVLTR promoter. Similarly, our ZFL assay, although incorporating a zebrafish-derived genomic promoter (zfEF1aPro) to enhance performance, remains chimeric [12]. The DRE assay, driven by seven XREs from the C57BL/6 mouse strain's CYP1A1 gene, utilises a minimal heat shock promoter (mHSP) of uncertain origin [17], traditionally sourced from Drosophila or *E. coli* [165], [166]. The ZF4 assay employs a synthetic ARE consensus sequence and a synthetic minimal promoter in the pGL4.37[luc2P/ARE/Hygro] reporter vector. The AREc32-related construct, PGL8x-ARE, combines eight AREs from rat gsta2 and mouse gsta1 genes, driven by a viral SV40 promoter [10].

We refer to the existing literature for a detailed discussion on the issues and pitfalls associated with transient or stable chimeric reporter constructs [8], [12], [167]. While some reports show the functional interchangeability of chimeric constructs across species due to mechanistic conservation [97], [168], others document specific experimental failures [163]. Determining the impact of chimeric constructs on the validity of species-related data remains challenging, particularly given the indistinct origins of some cloning components. Instead, we propose reevaluating the concept of species-specific reporter bioassays and advocating the adoption of novel tools for assay development. One such promising approach is CRISPR-Cas-mediated knock-ins, which allow site-directed integration of luminescent or fluorescent reporters adjacent to target genes (Lungu-Mitea et al., in preparation). This method ensures that reporter gene activation is entirely endogenous, potentially circumventing

unintended effects caused by classical transgenesis and by random integration of reporter constructs into the host genome via bacterial plasmid vectors.

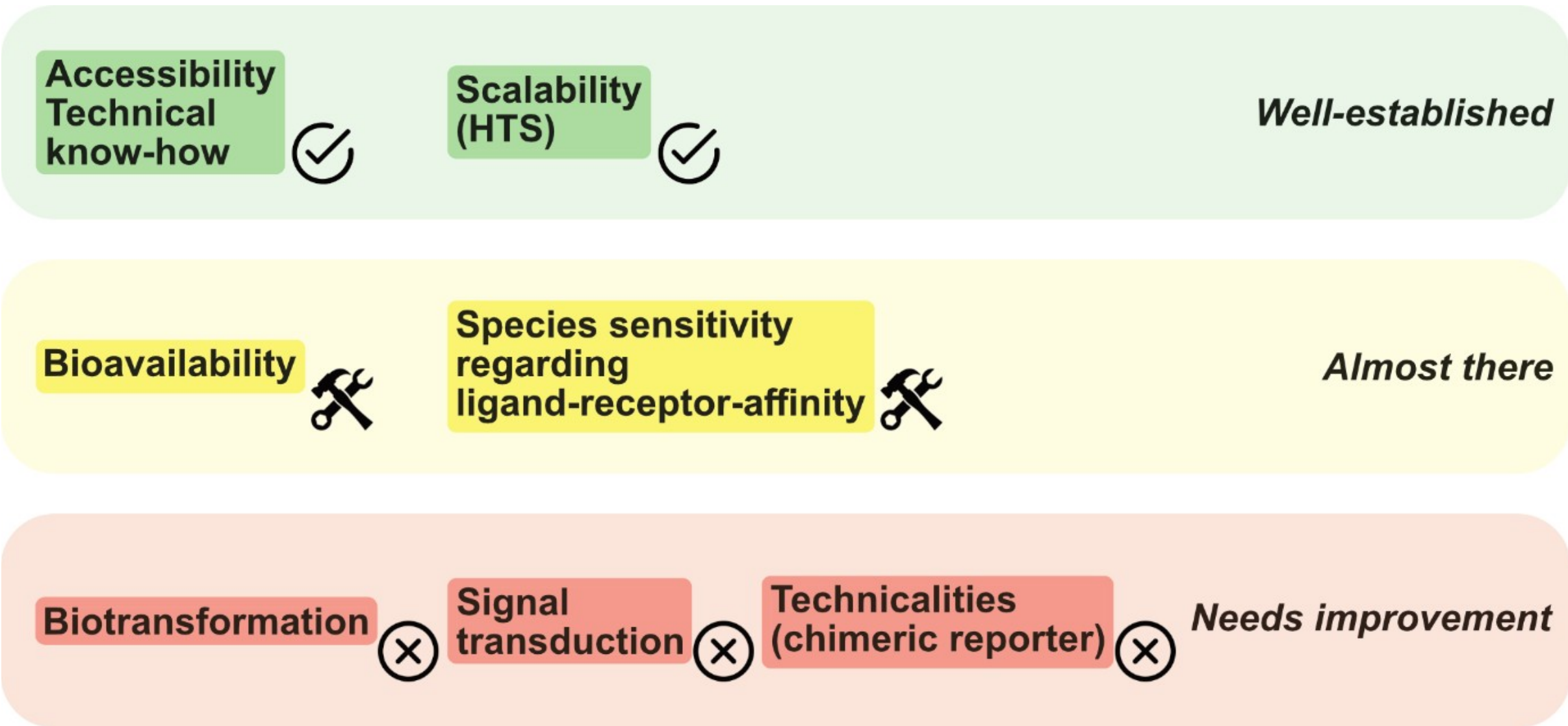

Fig. S44: Outlook on short to long-term challenges for fully integrating multi-species reporter bioassays into environmental monitoring.

5. References

[1] OECD, *Test No. 236: Fish Embryo Acute Toxicity (FET) Test*, no. July. OECD Publishing, 2025.

[2] B. I. Escher *et al.*, "Benchmarking Organic Micropollutants in Wastewater, Recycled Water and Drinking Water with In Vitro Bioassays," *Environ. Sci. Technol.*, vol. 48, no. 3, pp. 1940–1956, Feb. 2014, doi: 10.1021/es403899t.

[3] O. Golovko *et al.*, "Occurrence and removal of chemicals of emerging concern in wastewater treatment plants and their impact on receiving water systems," *Sci. Total Environ.*, vol. 754, p. 142122, Feb. 2021, doi: 10.1016/j.scitotenv.2020.142122.

[4] K. Itoh, K. I. Tong, and M. Yamamoto, "Molecular mechanism activating Nrf2-Keap1 pathway in regulation of adaptive response to electrophiles.," *Free Radic. Biol. Med.*, vol. 36, no. 10, pp. 1208–13, May 2004, doi: 10.1016/j.freeradbiomed.2004.02.075.

[5] E. J. Wright, K. Pereira De Castro, A. D. Joshi, and C. J. Elferink, "Canonical and non-canonical aryl hydrocarbon receptor signaling pathways," *Curr. Opin. Toxicol.*, vol. 2, pp. 87–92, Feb. 2017, doi: 10.1016/j.cotox.2017.01.001.

[6] W. Driever and Z. Rangini, "Characterization of a cell line derived from zebrafish (Brachydanio rerio) embryos.," *In Vitro Cell. Dev. Biol. Anim.*, vol. 29A, no. 9, pp. 749–54, Sep. 1993, [Online]. Available: http://www.ncbi.nlm.nih.gov/pubmed/8407719.

[7] S. Lungu-Mitea, A. Oskarsson, and J. Lundqvist, "Development of an oxidative stress in vitro assay in zebrafish (Danio rerio) cell lines," *Sci. Rep.*, vol. 8, no. 1, p. 12380, Dec. 2018, doi: 10.1038/s41598-018-30880-1.

[8] S. Lungu-Mitea and J. Lundqvist, "Potentials and pitfalls of transient in vitro reporter bioassays: interference by vector geometry and cytotoxicity in recombinant zebrafish cell lines," *Arch. Toxicol.*, no. 123456789, May 2020, doi: 10.1007/s00204-020-02783-6.

[9] H. D. Soule, J. Vazquez, A. Long, S. Albert, and M. Brennan, “A Human Cell Line From a Pleural Effusion Derived From a Breast Carcinoma 2,” *JNCI J. Natl. Cancer Inst.*, vol. 51, no. 5, pp. 1409–1416, Nov. 1973, doi: 10.1093/jnci/51.5.1409.

[10] X. J. Wang, J. D. Hayes, and C. R. Wolf, “Generation of a Stable Antioxidant Response Element–Driven Reporter Gene Cell Line and Its Use to Show Redox-Dependent Activation of Nrf2 by Cancer Chemotherapeutic Agents,” *Cancer Res.*, vol. 66, no. 22, pp. 10983–10994, Nov. 2006, doi: 10.1158/0008-5472.CAN-06-2298.

[11] C. Ghosh, Y. L. Zhou, and P. Collodi, “Derivation and characterization of a zebrafish liver cell line,” *Cell Biol. Toxicol.*, vol. 10, no. 3, pp. 167–176, 1994, doi: 10.1007/BF00757560.

[12] S. Lungu-Mitea, Y. Han, and J. Lundqvist, “Development, scrutiny, and modulation of transient reporter gene assays of the xenobiotic metabolism pathway in zebrafish hepatocytes,” *Cell Biol. Toxicol.*, vol. 39, no. 3, pp. 991–1013, Jun. 2023, doi: 10.1007/s10565-021-09659-0.

[13] G. He *et al.*, “Third-Generation Ah Receptor–Responsive Luciferase Reporter Plasmids: Amplification of Dioxin-Responsive Elements Dramatically Increases CALUX Bioassay Sensitivity and Responsiveness,” *Toxicol. Sci.*, vol. 123, no. 2, pp. 511–522, Oct. 2011, doi: 10.1093/toxsci/kfr189.

[14] D. P. Aden, A. Fogel, S. Plotkin, I. Damjanov, and B. B. Knowles, “Controlled synthesis of HBsAg in a differentiated human liver carcinoma-derived cell line,” *Nature*, vol. 282, no. 5739, pp. 615–616, Dec. 1979, doi: 10.1038/282615a0.

[15] J. C. Brennan *et al.*, “Development of Species-Specific Ah Receptor-Responsive Third Generation CALUX Cell Lines with Enhanced Responsiveness and Improved Detection Limits,” *Environ. Sci. Technol.*, vol. 49, no. 19, pp. 11903–11912, Sep. 2015, doi: 10.1021/acs.est.5b02906.

[16] A. K. Rosenmai *et al.*, “In vitro bioanalysis of drinking water from source to tap,” *Water Res.*, vol. 139, pp. 272–280, 2018, doi: 10.1016/j.watres.2018.04.009.

[17] S. Takeuchi, M. Iida, H. Yabushita, T. Matsuda, and H. Kojima, “In vitro screening for aryl hydrocarbon receptor agonistic activity in 200 pesticides using a highly sensitive reporter cell line, DR-EcoScreen cells, and in vivo mouse liver cytochrome P450-1A induction by propanil, diuron and linuron,” *Chemosphere*, vol. 74, no. 1, pp. 155–165, Dec. 2008, doi: 10.1016/j.chemosphere.2008.08.015.

[18] H. P. Bernhard, G. J. Darlington, and F. H. Ruddle, “Expression of liver phenotypes in cultured mouse hepatoma cells: Synthesis and secretion of serum albumin,” *Dev. Biol.*, vol. 35, no. 1, pp. 83–96, Nov. 1973, doi: 10.1016/0012-1606(73)90008-0.

[19] G. Carlsson, J. Patring, J. Kreuger, L. Norrgren, and A. Oskarsson, “Toxicity of 15 veterinary pharmaceuticals in zebrafish (Danio rerio) embryos,” *Aquat. Toxicol.*, vol. 126, pp. 30–41, Jan. 2013, doi: 10.1016/j.aquatox.2012.10.008.

[20] D. L. Filer, P. Kothiya, R. W. Setzer, R. S. Judson, and M. T. Martin, “tcpl: the ToxCast pipeline for high-throughput screening data,” *Bioinformatics*, vol. 33, no. 4, pp. 618–620, Feb. 2017, doi: 10.1093/bioinformatics/btw680.

[21] O. Jeroen, D. James, S. DebRoy, H. Wickham, and J. Horner, “RMySQL: Database Interface and ‘MySQL’ Driver for R.” 2025, [Online]. Available: https://github.com/r-dbi/rmysql.

[22] A. Abdelaziz, H. Spahn-Langguth, K.-W. Schramm, and I. V. Tetko, “Consensus Modeling for HTS Assays Using In silico Descriptors Calculates the Best Balanced Accuracy in Tox21 Challenge,” *Front. Environ. Sci.*, vol. 4, no. FEB, pp. 1–12, Feb. 2016, doi:

10.3389/fenvs.2016.00002.

[23] I. Sushko *et al.*, “Online chemical modeling environment (OCHEM): web platform for data storage, model development and publishing of chemical information,” *J. Comput. Aided. Mol. Des.*, vol. 25, no. 6, pp. 533–554, Jun. 2011, doi: 10.1007/s10822-011-9440-2.

[24] S. Kim *et al.*, “PubChem in 2021: new data content and improved web interfaces,” *Nucleic Acids Res.*, vol. 49, no. D1, pp. D1388–D1395, Jan. 2021, doi: 10.1093/nar/gkaa971.

[25] E. W. Sayers *et al.*, “Database resources of the national center for biotechnology information,” *Nucleic Acids Res.*, vol. 50, no. D1, pp. D20–D26, Jan. 2022, doi: 10.1093/nar/gkab1112.

[26] M. D. Hanwell, D. E. Curtis, D. C. Lonie, T. Vandermeersch, E. Zurek, and G. R. Hutchison, “Avogadro: an advanced semantic chemical editor, visualization, and analysis platform,” *J. Cheminform.*, vol. 4, no. 1, p. 17, Dec. 2012, doi: 10.1186/1758-2946-4-17.

[27] E. Vanquelef *et al.*, “R.E.D. Server: a web service for deriving RESP and ESP charges and building force field libraries for new molecules and molecular fragments,” *Nucleic Acids Res.*, vol. 39, no. suppl_2, pp. W511–W517, Jul. 2011, doi: 10.1093/nar/gkr288.

[28] G. M. Morris *et al.*, “AutoDock4 and AutoDockTools4: Automated docking with selective receptor flexibility,” *J. Comput. Chem.*, vol. 30, no. 16, pp. 2785–2791, Dec. 2009, doi: 10.1002/jcc.21256.

[29] M. F. Sanner, “Python: a programming language for software integration and development.,” *J. Mol. Graph. Model.*, vol. 17, no. 1, pp. 57–61, Feb. 1999, [Online]. Available: http://www.ncbi.nlm.nih.gov/pubmed/10660911.

[30] M. F. Sanner, A. J. Olson, and J.-C. Spehner, “Reduced surface: An efficient way to compute molecular surfaces,” *Biopolymers*, vol. 38, no. 3, pp. 305–320, Mar. 1996, doi: 10.1002/(SICI)1097-0282(199603)38:3<305::AID-BIP4>3.0.CO;2-Y.

[31] J. Jumper *et al.*, “Highly accurate protein structure prediction with AlphaFold,” *Nature*, vol. 596, no. 7873, pp. 583–589, Aug. 2021, doi: 10.1038/s41586-021-03819-2.

[32] M. Varadi *et al.*, “AlphaFold Protein Structure Database: massively expanding the structural coverage of protein-sequence space with high-accuracy models,” *Nucleic Acids Res.*, vol. 50, no. D1, pp. D439–D444, Jan. 2022, doi: 10.1093/nar/gkab1061.

[33] A. Bateman *et al.*, “UniProt: the universal protein knowledgebase in 2021,” *Nucleic Acids Res.*, vol. 49, no. D1, pp. D480–D489, Jan. 2021, doi: 10.1093/nar/gkaa1100.

[34] H. M. Berman, “The Protein Data Bank,” *Nucleic Acids Res.*, vol. 28, no. 1, pp. 235–242, Jan. 2000, doi: 10.1093/nar/28.1.235.

[35] S. K. Burley *et al.*, “RCSB Protein Data Bank: powerful new tools for exploring 3D structures of biological macromolecules for basic and applied research and education in fundamental biology, biomedicine, biotechnology, bioengineering and energy sciences,” *Nucleic Acids Res.*, vol. 49, no. D1, pp. D437–D451, Jan. 2021, doi: 10.1093/nar/gkaa1038.

[36] J. Gruszczyk *et al.*, “Cryo-EM structure of the agonist-bound Hsp90-XAP2-AHR cytosolic complex,” *Nat. Commun.*, vol. 13, no. 1, p. 7010, Nov. 2022, doi: 10.1038/s41467-022-34773-w.

[37] M. Mirdita, K. Schütze, Y. Moriwaki, L. Heo, S. Ovchinnikov, and M. Steinegger, “ColabFold: making protein folding accessible to all,” *Nat. Methods*, vol. 19, no. 6, pp. 679–682, Jun. 2022, doi: 10.1038/s41592-022-01488-1.

[38] R. Anandakrishnan, B. Aguilar, and A. V. Onufriev, “H++ 3.0: automating pK prediction and the preparation of biomolecular structures for atomistic molecular modeling and simulations,” *Nucleic Acids Res.*, vol. 40, no. W1, pp. W537–W541, Jul. 2012, doi: 10.1093/nar/gks375.

[39] J. C. Gordon, J. B. Myers, T. Folta, V. Shoja, L. S. Heath, and A. Onufriev, “H++: a server for estimating pKas and adding missing hydrogens to macromolecules,” *Nucleic Acids Res.*, vol. 33, no. Web Server, pp. W368–W371, Jul. 2005, doi: 10.1093/nar/gki464.

[40] K. Okonechnikov, O. Golosova, and M. Fursov, “Unipro UGENE: a unified bioinformatics toolkit,” *Bioinformatics*, vol. 28, no. 8, pp. 1166–1167, Apr. 2012, doi: 10.1093/bioinformatics/bts091.

[41] W. Liu *et al.*, “IBS: an illustrator for the presentation and visualization of biological sequences,” *Bioinformatics*, vol. 31, no. 20, pp. 3359–3361, Oct. 2015, doi: 10.1093/bioinformatics/btv362.

[42] S. M. K. Glasauer and S. C. F. Neuhauss, “Whole-genome duplication in teleost fishes and its evolutionary consequences,” *Mol. Genet. Genomics*, vol. 289, no. 6, pp. 1045–1060, Dec. 2014, doi: 10.1007/s00438-014-0889-2.

[43] O. Trott and A. J. Olson, “AutoDock Vina: Improving the speed and accuracy of docking with a new scoring function, efficient optimization, and multithreading,” *J. Comput. Chem.*, vol. 31, no. 2, pp. 455–461, Jan. 2010, doi: 10.1002/jcc.21334.

[44] D. Seeliger and B. L. de Groot, “Ligand docking and binding site analysis with PyMOL and Autodock/Vina,” *J. Comput. Aided. Mol. Des.*, vol. 24, no. 5, pp. 417–422, May 2010, doi: 10.1007/s10822-010-9352-6.

[45] Ulrich *et al.*, “UFZ-LSER database v 3.2 [Internet].” Leipzig, Deutschland, Helmholtz Zentrum für Umweltforschung - UFZ, 2017, [Online]. Available: http://www.ufz.de/lserd.

[46] F. C. Fischer *et al.*, “Modeling Exposure in the Tox21 in Vitro Bioassays,” *Chem. Res. Toxicol.*, vol. 30, no. 5, pp. 1197–1208, May 2017, doi: 10.1021/acs.chemrestox.7b00023.

[47] S. Endo, B. I. Escher, and K.-U. Goss, “Capacities of Membrane Lipids to Accumulate Neutral Organic Chemicals,” *Environ. Sci. Technol.*, vol. 45, no. 14, pp. 5912–5921, Jul. 2011, doi: 10.1021/es200855w.

[48] S. Endo and K.-U. Goss, “Serum Albumin Binding of Structurally Diverse Neutral Organic Compounds: Data and Models,” *Chem. Res. Toxicol.*, vol. 24, no. 12, pp. 2293–2301, Dec. 2011, doi: 10.1021/tx200431b.

[49] B. I. Escher, C. van Daele, M. Dutt, J. Y. M. Tang, and R. Altenburger, “Most Oxidative Stress Response In Water Samples Comes From Unknown Chemicals: The Need For Effect-Based Water Quality Trigger Values,” *Environ. Sci. Technol.*, vol. 47, no. 13, pp. 7002–7011, Jul. 2013, doi: 10.1021/es304793h.

[50] B. I. Escher, P. Neale, and F. Leusch, *Bioanalytical tools in water quality assessment Beate Escher, Peta Neale and Frederic Leusch*, 2nd editio. London: IWA Publishing, 2021.

[51] M. Weimer, X. Jiang, O. Ponta, S. Stanzel, A. Freyberger, and A. Kopp-Schneider, “The impact of data transformations on concentration–response modeling,” *Toxicol. Lett.*, vol. 213, no. 2, pp. 292–298, Sep. 2012, doi: 10.1016/j.toxlet.2012.07.012.

[52] P. A. Neale *et al.*, “Linking in Vitro Effects and Detected Organic Micropollutants in Surface Water Using Mixture-Toxicity Modeling,” *Environ. Sci. Technol.*, vol. 49, no. 24, pp. 14614–14624, Dec. 2015, doi: 10.1021/acs.est.5b04083.

[53] P. A. Neale *et al.*, “Integrating chemical analysis and bioanalysis to evaluate the contribution of wastewater effluent on the micropollutant burden in small streams,” *Sci. Total Environ.*, vol. 576, pp. 785–795, Jan. 2017, doi: 10.1016/j.scitotenv.2016.10.141.

[54] P. A. Neale *et al.*, “Assessing the Mixture Effects in In Vitro Bioassays of Chemicals Occurring in Small Agricultural Streams during Rain Events,” *Environ. Sci. Technol.*, vol. 54, no. 13, pp. 8280–8290, Jul. 2020, doi: 10.1021/acs.est.0c02235.

[55] B. I. Escher *et al.*, “Water quality assessment using the AREc32 reporter gene assay indicative of the oxidative stress response pathway,” *J. Environ. Monit.*, vol. 14, no. 11, pp. 2877–2885, 2012, doi: 10.1039/c2em30506b.

[56] B. I. Escher, P. A. Neale, and D. L. Villeneuve, “The advantages of linear concentration-response curves for in vitro bioassays with environmental samples,” *Environ. Toxicol. Chem.*, vol. 37, no. 9, pp. 2273–2280, Sep. 2018, doi: 10.1002/etc.4178.

[57] M. Wagner, E. L. M. Vermeirssen, S. Buchinger, M. Behr, A. Magdeburg, and J. Oehlmann, “Deriving bio-equivalents from in vitro bioassays: Assessment of existing uncertainties and strategies to improve accuracy and reporting,” *Environ. Toxicol. Chem.*, vol. 32, no. 8, pp. 1906–1917, Aug. 2013, doi: 10.1002/etc.2256.

[58] E. Hommel *et al.*, “Following the Mixtures of Organic Micropollutants with In Vitro Bioassays in a Large Lowland River from Source to Sea,” *ACS Environ. Au*, vol. 5, no. 4, pp. 363–375, 2025, doi: 10.1021/acsenvironau.4c00059.

[59] S. Lungu-Mitea *et al.*, “Modeling Bioavailable Concentrations in Zebrafish Cell Lines and Embryos Increases the Correlation of Toxicity Potencies across Test Systems,” *Environ. Sci. Technol.*, vol. 55, no. 1, pp. 447–457, Jan. 2021, doi: 10.1021/acs.est.0c04872.

[60] M. Kobayashi *et al.*, “The antioxidant defense system Keap1-Nrf2 comprises a multiple sensing mechanism for responding to a wide range of chemical compounds.,” *Mol. Cell. Biol.*, vol. 29, no. 2, pp. 493–502, Jan. 2009, doi: 10.1128/MCB.01080-08.

[61] K. Takaya *et al.*, “Validation of the multiple sensor mechanism of the Keap1-Nrf2 system,” *Free Radic. Biol. Med.*, vol. 53, no. 4, pp. 817–827, Aug. 2012, doi: 10.1016/j.freeradbiomed.2012.06.023.

[62] A. Slaninova, M. Smutna, H. Modra, and Z. Svobodova, “A review: oxidative stress in fish induced by pesticides.,” *Neuro Endocrinol. Lett.*, vol. 30 Suppl 1, no. April, pp. 2–12, 2009, doi: NEL300709R01 [pii].

[63] K. Itoh, K. Igarashi, N. Hayashi, M. Nishizawa, and M. Yamamoto, “Cloning and characterization of a novel erythroid cell-derived CNC family transcription factor heterodimerizing with the small Maf family proteins.,” *Mol. Cell. Biol.*, vol. 15, no. 8, pp. 4184–93, Aug. 1995, [Online]. Available: http://www.ncbi.nlm.nih.gov/pubmed/7623813.

[64] P. Moi, K. Chan, I. Asunis, A. Cao, and Y. W. Kan, “Isolation of NF-E2-related factor 2 (Nrf2), a NF-E2-like basic leucine zipper transcriptional activator that binds to the tandem NF-E2/AP1 repeat of the beta-globin locus control region.,” *Proc. Natl. Acad. Sci. U. S. A.*, vol. 91, no. 21, pp. 9926–30, Oct. 1994, doi: 10.1073/pnas.91.21.9926.

[65] J. D. Hayes and A. T. Dinkova-Kostova, “The Nrf2 regulatory network provides an interface between redox and intermediary metabolism.,” *Trends Biochem. Sci.*, vol. 39, no. 4, pp. 199–218, Apr. 2014, doi: 10.1016/j.tibs.2014.02.002.

[66] K. Itoh, P. Ye, T. Matsumiya, K. Tanji, and T. Ozaki, “Emerging functional cross-talk between the Keap1-Nrf2 system and mitochondria.,” *J. Clin. Biochem. Nutr.*, vol. 56, no. 2, pp. 91–7,

Mar. 2015, doi: 10.3164/jcbn.14-134.

[67] M. Kobayashi and M. Yamamoto, “Nrf2-Keap1 regulation of cellular defense mechanisms against electrophiles and reactive oxygen species.,” *Adv. Enzyme Regul.*, vol. 46, no. 1, pp. 113–40, 2006, doi: 10.1016/j.advenzreg.2006.01.007.

[68] T. Nguyen, P. J. Sherratt, and C. B. Pickett, “Regulatory mechanisms controlling gene expression mediated by the antioxidant response element.,” *Annu. Rev. Pharmacol. Toxicol.*, vol. 43, no. 1, pp. 233–60, 2003, doi: 10.1146/annurev.pharmtox.43.100901.140229.

[69] A. K. Sharma, G. Taneja, D. Khanna, and S. K. Rajput, “Reactive oxygen species: friend or foe?,” *RSC Adv.*, vol. 5, no. 71, pp. 57267–57276, 2015, doi: 10.1039/C5RA07927F.

[70] T. H. Rushmore, M. R. Morton, and C. B. Pickett, “The antioxidant responsive element. Activation by oxidative stress and identification of the DNA consensus sequence required for functional activity.,” *J. Biol. Chem.*, vol. 266, no. 18, pp. 11632–9, Jun. 1991, [Online]. Available: http://www.ncbi.nlm.nih.gov/pubmed/1646813.

[71] W. W. Wasserman and W. E. Fahl, “Functional antioxidant responsive elements.,” *Proc. Natl. Acad. Sci. U. S. A.*, vol. 94, no. 10, pp. 5361–6, May 1997, [Online]. Available: http://www.ncbi.nlm.nih.gov/pubmed/9144242.

[72] C. Köhle and K. W. Bock, “Coordinate regulation of Phase I and II xenobiotic metabolisms by the Ah receptor and Nrf2.,” *Biochem. Pharmacol.*, vol. 73, no. 12, pp. 1853–62, Jun. 2007, doi: 10.1016/j.bcp.2007.01.009.

[73] K. Itoh *et al.*, “Keap1 represses nuclear activation of antioxidant responsive elements by Nrf2 through binding to the amino-terminal Neh2 domain.,” *Genes Dev.*, vol. 13, no. 1, pp. 76–86, Jan. 1999, doi: 10.1101/gad.13.1.76.

[74] K. Itoh, T. Ishii, N. Wakabayashi, and M. Yamamoto, “Regulatory mechanisms of cellular response to oxidative stress.,” *Free Radic. Res.*, vol. 31, no. 4, pp. 319–24, Oct. 1999, doi: 10.1080/10715769900300881.

[75] M. Kobayashi *et al.*, “Identification of the interactive interface and phylogenic conservation of the Nrf2-Keap1 system.,” *Genes Cells*, vol. 7, no. 8, pp. 807–20, Aug. 2002, doi: 10.1046/j.1365-2443.2002.00561.x.

[76] M. Xiang, A. Namani, S. Wu, and X. Wang, “Nrf2: bane or blessing in cancer?,” *J. Cancer Res. Clin. Oncol.*, vol. 140, no. 8, pp. 1251–9, Aug. 2014, doi: 10.1007/s00432-014-1627-1.

[77] T. Yamamoto *et al.*, “Physiological significance of reactive cysteine residues of Keap1 in determining Nrf2 activity.,” *Mol. Cell. Biol.*, vol. 28, no. 8, pp. 2758–70, Apr. 2008, doi: 10.1128/MCB.01704-07.

[78] A. T. Dinkova-Kostova, R. V. Kostov, and P. Canning, “Keap1, the cysteine-based mammalian intracellular sensor for electrophiles and oxidants,” *Arch. Biochem. Biophys.*, vol. 617, pp. 84–93, Mar. 2017, doi: 10.1016/j.abb.2016.08.005.

[79] S. Fourquet, R. Guerois, D. Biard, and M. B. Toledano, “Activation of NRF2 by nitrosative agents and H2O2 involves KEAP1 disulfide formation.,” *J. Biol. Chem.*, vol. 285, no. 11, pp. 8463–71, Mar. 2010, doi: 10.1074/jbc.M109.051714.

[80] K. Igarashi, K. Kataokat, K. Itoh, N. Hayashi, M. Nishizawa, and M. Yamamoto, “Regulation of transcription by dimerization of erythroid factor NF-E2 p45 with small Maf proteins,” *Nature*, vol. 367, no. 6463, pp. 568–572, Feb. 1994, doi: 10.1038/367568a0.

[81] a T. Dinkova-Kostova, M. a Massiah, R. E. Bozak, R. J. Hicks, and P. Talalay, “Potency of

Michael reaction acceptors as inducers of enzymes that protect against carcinogenesis depends on their reactivity with sulfhydryl groups.," *Proc. Natl. Acad. Sci. U. S. A.*, vol. 98, no. 6, pp. 3404–9, Mar. 2001, doi: 10.1073/pnas.051632198.

[82] N. Wakabayashi *et al.*, "Protection against electrophile and oxidant stress by induction of the phase 2 response: fate of cysteines of the Keap1 sensor modified by inducers.," *Proc. Natl. Acad. Sci. U. S. A.*, vol. 101, no. 7, pp. 2040–5, Feb. 2004, doi: 10.1073/pnas.0307301101.

[83] R. Saito *et al.*, "Characterizations of Three Major Cysteine Sensors of Keap1 in Stress Response.," *Mol. Cell. Biol.*, vol. 36, no. 2, pp. 271–84, Nov. 2015, doi: 10.1128/MCB.00868-15.

[84] D. D. Zhang and M. Hannink, "Distinct cysteine residues in Keap1 are required for Keap1-dependent ubiquitination of Nrf2 and for stabilization of Nrf2 by chemopreventive agents and oxidative stress.," *Mol. Cell. Biol.*, vol. 23, no. 22, pp. 8137–51, Nov. 2003, doi: 10.1128/MCB.23.22.8137-8151.2003.

[85] K. Taguchi, H. Motohashi, and M. Yamamoto, "Molecular mechanisms of the Keap1-Nrf2 pathway in stress response and cancer evolution," *Genes to Cells*, vol. 16, no. 2, pp. 123–140, Feb. 2011, doi: 10.1111/j.1365-2443.2010.01473.x.

[86] H. K. Bryan, A. Olayanju, C. E. Goldring, and B. K. Park, "The Nrf2 cell defence pathway: Keap1-dependent and -independent mechanisms of regulation," *Biochem. Pharmacol.*, vol. 85, no. 6, pp. 705–717, Mar. 2013, doi: 10.1016/j.bcp.2012.11.016.

[87] Y. Horie *et al.*, "Molecular basis for the disruption of Keap1–Nrf2 interaction via Hinge & Latch mechanism," *Commun. Biol.*, vol. 4, no. 1, pp. 1–11, 2021, doi: 10.1038/s42003-021-02100-6.

[88] Y. Fuse and M. Kobayashi, "Conservation of the Keap1-Nrf2 System: An Evolutionary Journey through Stressful Space and Time.," *Molecules*, vol. 22, no. 3, pp. 1–22, Mar. 2017, doi: 10.3390/molecules22030436.

[89] M. Kobayashi and M. Yamamoto, "Molecular mechanisms activating the Nrf2-Keap1 pathway of antioxidant gene regulation.," *Antioxid. Redox Signal.*, vol. 7, no. 3–4, pp. 385–94, 2005, doi: 10.1089/ars.2005.7.385.

[90] L. Li, M. Kobayashi, H. Kaneko, Y. Nakajima-Takagi, Y. Nakayama, and M. Yamamoto, "Molecular evolution of Keap1. Two Keap1 molecules with distinctive intervening region structures are conserved among fish.," *J. Biol. Chem.*, vol. 283, no. 6, pp. 3248–55, Feb. 2008, doi: 10.1074/jbc.M708702200.

[91] A. R. Timme-Laragy *et al.*, "Nrf2b, novel zebrafish paralog of oxidant-responsive transcription factor NF-E2-related factor 2 (NRF2).," *J. Biol. Chem.*, vol. 287, no. 7, pp. 4609–27, Feb. 2012, doi: 10.1074/jbc.M111.260125.

[92] V. T. Nguyen *et al.*, "Generation and characterization of keap1a- and keap1b-knockout zebrafish," *Redox Biol.*, vol. 36, no. July, p. 101667, Sep. 2020, doi: 10.1016/j.redox.2020.101667.

[93] P. M. GARRISON, K. TULLIS, J. M. M. J. G. AARTS, A. BROUWER, J. P. GIESY, and M. S. DENISON, "Species-Specific Recombinant Cell Lines as Bioassay Systems for the Detection of 2,3,7,8-Tetrachlorodibenzo- p -dioxin-like Chemicals," *Toxicol. Sci.*, vol. 30, no. 2, pp. 194–203, 1996, doi: 10.1093/toxsci/30.2.194.

[94] P. M. Garrison, K. Tullis, J. M. Aarts, A. Brouwer, J. P. Giesy, and M. S. Denison, "Species-specific recombinant cell lines as bioassay systems for the detection of 2,3,7,8-tetrachlorodibenzo-p-dioxin-like chemicals.," *Fundam. Appl. Toxicol.*, vol. 30, no. 2, pp. 194–

203, Apr. 1996, [Online]. Available: http://www.ncbi.nlm.nih.gov/pubmed/8812265.

[95] D. Han, S. R. Nagy, and M. S. Denison, "Comparison of recombinant cell bioassays for the detection of Ah receptor agonists," *BioFactors*, vol. 20, no. 1, pp. 11–22, 2004, doi: 10.1002/biof.5520200102.

[96] A. Murk, "Chemical-Activated Luciferase Gene Expression (CALUX): A Novelin VitroBioassay for Ah Receptor Active Compounds in Sediments and Pore Water," *Fundam. Appl. Toxicol.*, vol. 33, no. 1, pp. 149–160, Sep. 1996, doi: 10.1006/faat.1996.0152.

[97] C. A. Richter, V. L. Tieber, M. S. Denison, and J. P. Giesy, "An in vitro rainbow trout cell bioassay for aryl hydrocarbon receptor- mediated toxins," *Environ. Toxicol. Chem.*, vol. 16, no. 3, pp. 543–550, 1997, doi: 10.1897/1551-5028(1997)016<0543:AIVRTC>2.3.CO;2.

[98] K. Eichbaum *et al.*, "In vitro bioassays for detecting dioxin-like activity — Application potentials and limits of detection, a review," *Sci. Total Environ.*, vol. 487, no. 1, pp. 37–48, Jul. 2014, doi: 10.1016/j.scitotenv.2014.03.057.

[99] M. H. El-Fouly, C. Richter, J. P. Giesy, and M. S. Denison, "Production of a novel recombinant cell line for use as a bioassay system for detection of 2,3,7,8-tetrachlorodibenzo- P -dioxin-like chemicals," *Environ. Toxicol. Chem.*, vol. 13, no. 10, pp. 1581–1588, Oct. 1994, doi: 10.1002/etc.5620131006.

[100] P. A. Neale *et al.*, "Application of Effect-Based Methods to Water Quality Monitoring: Answering Frequently Asked Questions by Water Quality Managers, Regulators, and Policy Makers," *Environ. Sci. Technol.*, vol. 57, no. 15, pp. 6023–6032, Apr. 2023, doi: 10.1021/acs.est.2c06365.

[101] P. A. Neale, B. I. Escher, M. L. de Baat, J. Enault, and F. D. L. Leusch, "Effect-Based Trigger Values Are Essential for the Uptake of Effect-Based Methods in Water Safety Planning," *Environ. Toxicol. Chem.*, vol. 42, no. 3, pp. 714–726, Mar. 2023, doi: 10.1002/etc.5544.

[102] R. van der Oost, G. Sileno, M. Suárez-Muñoz, M. T. Nguyen, H. Besselink, and A. Brouwer, "SIMONI (Smart Integrated Monitoring) as a novel bioanalytical strategy for water quality assessment: Part I–model design and effect-based trigger values," *Environ. Toxicol. Chem.*, vol. 36, no. 9, pp. 2385–2399, Sep. 2017, doi: 10.1002/etc.3836.

[103] State Water Resources Control Board, "Water Quality Control Policy for Recycled Water State Water Resources Control Board California Environmental Protection Agency," 2018. [Online]. Available: http://www.waterboards.ca.gov.

[104] M. Carere, A.-S. Wernersson, T. Lettieri, and S. Buchinger, "Technical Proposal for Effect-Based Monitoring and Assessment under the Water Framework Directive," 2021. doi: 10.13140/RG.2.2.19954.27847.

[105] B. I. Escher *et al.*, "Effect-based trigger values for in vitro and in vivo bioassays performed on surface water extracts supporting the environmental quality standards (EQS) of the European Water Framework Directive," *Sci. Total Environ.*, vol. 628–629, pp. 748–765, 2018, doi: 10.1016/j.scitotenv.2018.01.340.

[106] B. I. Escher, P. A. Neale, and F. D. L. Leusch, "Effect-based trigger values for in vitro bioassays: Reading across from existing water quality guideline values," *Water Res.*, vol. 81, pp. 137–148, Sep. 2015, doi: 10.1016/j.watres.2015.05.049.

[107] B. I. Escher *et al.*, "Effect-based trigger values for in vitro and in vivo bioassays performed on surface water extracts supporting the environmental quality standards (EQS) of the European Water Framework Directive," *Sci. Total Environ.*, vol. 628–629, pp. 748–765, Jul. 2018, doi:

10.1016/j.scitotenv.2018.01.340.

[108] F. Tamaddon and W. Hogland, “Characterization and coincineration of waste in a paper milla case study,” *Linnaeus Eco-Tech*, pp. 699–708, Dec. 2007, doi: 10.15626/Eco-Tech.2007.076.

[109] M. Almemark, G. Finnveden, and B. Frostell, “Treatment Technologies for Organochlorine-Containing Sludges and Concentrates from External Treatment of Pulp and Paper Wastewaters,” *Water Sci. Technol.*, vol. 24, no. 3–4, pp. 319–329, Aug. 1991, doi: 10.2166/wst.1991.0488.

[110] L. Morales *et al.*, “Oceanic Sink and Biogeochemical Controls on the Accumulation of Polychlorinated Dibenzo- p -dioxins, Dibenzofurans, and Biphenyls in Plankton,” *Environ. Sci. Technol.*, vol. 49, no. 23, pp. 13853–13861, Dec. 2015, doi: 10.1021/acs.est.5b01360.

[111] J. Castro-Jiménez *et al.*, “Atmospheric input of POPs into Lake Maggiore (Northern Italy): PCDD/F and dioxin-like PCB profiles and fluxes in the atmosphere and aquatic system,” *Chemosphere*, vol. 73, no. 1, pp. S122–S130, Aug. 2008, doi: 10.1016/j.chemosphere.2007.06.097.

[112] S. Lee *et al.*, “Measured and predicted affinities of binding and relative potencies to activate the AhR of PAHs and their alkylated analogues,” *Chemosphere*, vol. 139, pp. 23–29, 2015, doi: 10.1016/j.chemosphere.2015.05.033.

[113] M. M. Lam, R. Bülow, M. Engwall, J. P. Giesy, and M. Larsson, “Methylated PACs are more potent than their parent compounds: A study of aryl hydrocarbon receptor–mediated activity, degradability, and mixture interactions in the H4IIE- luc assay,” *Environ. Toxicol. Chem.*, vol. 37, no. 5, pp. 1409–1419, May 2018, doi: 10.1002/etc.4087.

[114] R. Lille-Langøy, K. B. Jørgensen, A. Goksøyr, D. M. Pampanin, M. O. Sydnes, and O. A. Karlsen, “Substituted Two- to Five-Ring Polycyclic Aromatic Compounds Are Potent Agonists of Atlantic Cod (Gadus morhua) Aryl Hydrocarbon Receptors Ahr1a and Ahr2a,” *Environ. Sci. Technol.*, vol. 55, no. 22, pp. 15123–15135, Nov. 2021, doi: 10.1021/acs.est.1c02946.

[115] A. R. Johnsen, L. Y. Wick, and H. Harms, “Principles of microbial PAH-degradation in soil,” *Environ. Pollut.*, vol. 133, no. 1, pp. 71–84, Jan. 2005, doi: 10.1016/j.envpol.2004.04.015.

[116] L. Goedtke *et al.*, “Polycyclic aromatic hydrocarbons activate the aryl hydrocarbon receptor and the constitutive androstane receptor to regulate xenobiotic metabolism in human liver cells,” *Int. J. Mol. Sci.*, vol. 22, no. 1, pp. 1–17, 2021, doi: 10.3390/ijms22010372.

[117] S. Giani Tagliabue, S. C. Faber, S. Motta, M. S. Denison, and L. Bonati, “Modeling the binding of diverse ligands within the Ah receptor ligand binding domain,” *Sci. Rep.*, vol. 9, no. 1, p. 10693, Jul. 2019, doi: 10.1038/s41598-019-47138-z.

[118] S. C. Monteriolo, A. di Domenico, V. Silano, G. Viviano, and G. Zapponi, “2,3,7,8-TCDD LEVELS AND DISTRIBUTION IN THE ENVIRONMENT AT SEVESO AFTER THE ICMESA ACCIDENT ON JULY 10th, 1976,” in *Chlorinated Dioxins & Related Compounds*, Elsevier, 1982, pp. 127–136.

[119] A. R. Batterman, P. M. Cook, K. B. Lodge, D. B. Lothenbach, and B. C. Butterworth, “Methodology used for a laboratory determination of relative contributions of water, sediment and food chain routes of uptake for 2,3,7,8-TCDD bioaccumulation by lake trout in Lake Ontario,” *Chemosphere*, vol. 19, no. 1–6, pp. 451–458, Jan. 1989, doi: 10.1016/0045-6535(89)90351-2.

[120] B. S. Giri, I. . Karimi, and M. . Ray, “Modeling and Monte Carlo simulation of TCDD transport in a river,” *Water Res.*, vol. 35, no. 5, pp. 1263–1279, Apr. 2001, doi: 10.1016/S0043-1354(00)00379-1.

[121] J. K. H. de Schepper, T. Slootweg, P. Behnisch, E. Felzel, and C. J. Houtman, “Beyond the Drinking Water Directive: The use of reporter gene assays as an added tool for effect-based monitoring of polycyclic aromatic hydrocarbons and polychlorinated biphenyls in drinking water sources,” *Sci. Total Environ.*, vol. 941, no. May, p. 173366, Sep. 2024, doi: 10.1016/j.scitotenv.2024.173366.

[122] P. Y. Kunz *et al.*, “Effect-based tools for monitoring estrogenic mixtures: Evaluation of five in vitro bioassays,” *Water Res.*, vol. 110, pp. 378–388, Mar. 2017, doi: 10.1016/j.watres.2016.10.062.

[123] L. Gómez *et al.*, “Estrogenicity of chemical mixtures revealed by a panel of bioassays,” *Sci. Total Environ.*, vol. 785, p. 147284, Sep. 2021, doi: 10.1016/j.scitotenv.2021.147284.

[124] E. B. Reiter, B. I. Escher, U. Siebert, and A. Jahnke, “Activation of the xenobiotic metabolism and oxidative stress response by mixtures of organic pollutants extracted with in-tissue passive sampling from liver, kidney, brain and blubber of marine mammals,” *Environ. Int.*, vol. 165, no. June, p. 107337, 2022, doi: 10.1016/j.envint.2022.107337.

[125] M. Brinkmann *et al.*, “Multiple lines of evidence to assess risk from dioxins and dioxin-like chemicals in sediment and fish from waterbodies along a large prairie river,” *J. Soils Sediments*, no. Kondolf 1997, Aug. 2023, doi: 10.1007/s11368-023-03620-w.

[126] K. Hilscherova *et al.*, “Characterization of dioxin-like activity of sediments from a Czech River Basin,” *Environ. Toxicol. Chem.*, vol. 20, no. 12, pp. 2768–2777, Dec. 2001, doi: 10.1002/etc.5620201216.

[127] S. Keiter *et al.*, “Activities and identification of aryl hydrocarbon receptor agonists in sediments from the Danube river,” *Anal. Bioanal. Chem.*, vol. 390, no. 8, pp. 2009–2019, Apr. 2008, doi: 10.1007/s00216-007-1652-x.

[128] G. Liberatori *et al.*, “Occurrence and spatial distribution of dioxin and dioxin-like compounds in topsoil of Taranto (Apulia, Italy) by GC-MS analysis and DR-CALUX® bioassay,” *Chemosphere*, vol. 279, p. 130576, Sep. 2021, doi: 10.1016/j.chemosphere.2021.130576.

[129] C. Budin *et al.*, “Detection of high PBDD/Fs levels and dioxin-like activity in toys using a combination of GC-HRMS, rat-based and human-based DR CALUX® reporter gene assays,” *Chemosphere*, vol. 251, p. 126579, Jul. 2020, doi: 10.1016/j.chemosphere.2020.126579.

[130] P. A. Behnisch, “Screening of Dioxin-Like Toxicity Equivalents for Various Matrices with Wildtype and Recombinant Rat Hepatoma H4IIE Cells,” *Toxicol. Sci.*, vol. 69, no. 1, pp. 125–130, Sep. 2002, doi: 10.1093/toxsci/69.1.125.

[131] D. Delistraty, “Toxic equivalency factor approach for risk assessment of polycyclic aromatic hydrocarbons,” *Toxicol. Environ. Chem.*, vol. 64, no. 1–4, pp. 81–108, Nov. 1997, doi: 10.1080/02772249709358542.

[132] S. P. Bhavsar, E. J. Reiner, A. Hayton, R. Fletcher, and K. MacPherson, “Converting Toxic Equivalents (TEQ) of dioxins and dioxin-like compounds in fish from one Toxic Equivalency Factor (TEF) scheme to another,” *Environ. Int.*, vol. 34, no. 7, pp. 915–921, Oct. 2008, doi: 10.1016/j.envint.2008.02.001.

[133] B. I. Escher, N. Bramaz, J. F. Mueller, P. Quayle, S. Rutishauser, and E. L. M. Vermeirssen, “Toxic equivalent concentrations (TEQs) for baseline toxicity and specific modes of action as a tool to improve interpretation of ecotoxicity testing of environmental samples,” *J. Environ. Monit.*, vol. 10, no. 5, p. 612, 2008, doi: 10.1039/b800949j.

[134] J. A. Doering, D. E. Tillitt, and S. Wiseman, “Reevaluation of 2,3,7,8-Tetrachlorodibenzo- p

-Dioxin Equivalency Factors for Dioxin-Like Polychlorinated Dibenzo- p -Dioxins, Polychlorinated Dibenzofurans, and Polychlorinated Biphenyls for Fishes," *Environ. Toxicol. Chem.*, vol. 42, no. 10, pp. 2215–2228, Oct. 2023, doi: 10.1002/etc.5690.

[135] M. B. M. van Duursen, K. I. van Ede, and M. van den Berg, "One TEF concept does not fit all: The case for human risk assessment of polychlorinated biphenyls," *Curr. Opin. Toxicol.*, vol. 2, pp. 103–108, Feb. 2017, doi: 10.1016/j.cotox.2017.01.005.

[136] W. H. Bisson *et al.*, "Modeling of the Aryl Hydrocarbon Receptor (AhR) Ligand Binding Domain and Its Utility in Virtual Ligand Screening to Predict New AhR Ligands," *J. Med. Chem.*, vol. 52, no. 18, pp. 5635–5641, Sep. 2009, doi: 10.1021/jm900199u.

[137] A. P. Plonski and S. M. Reed, "Assessing protein homology models with docking reproducibility," *J. Mol. Graph. Model.*, vol. 121, no. February, p. 108430, Jun. 2023, doi: 10.1016/j.jmgm.2023.108430.

[138] G. ZeRuth and R. S. Pollenz, "Functional analysis of cis-regulatory regions within the dioxin-inducible CYP1A promoter/enhancer region from zebrafish (Danio rerio)," *Chem. Biol. Interact.*, vol. 170, no. 2, pp. 100–113, Nov. 2007, doi: 10.1016/j.cbi.2007.07.003.

[139] J. V. Schmidt and C. A. Bradfield, "AH RECEPTOR SIGNALING PATHWAYS," *Annu. Rev. Cell Dev. Biol.*, vol. 12, no. 1, pp. 55–89, Nov. 1996, doi: 10.1146/annurev.cellbio.12.1.55.

[140] S. O. Simmons, C.-Y. Fan, and R. Ramabhadran, "Cellular Stress Response Pathway System as a Sentinel Ensemble in Toxicological Screening," *Toxicol. Sci.*, vol. 111, no. 2, pp. 202–225, Oct. 2009, doi: 10.1093/toxsci/kfp140.

[141] L. A. Van Tiem and R. T. Di Giulio, "AHR2 knockdown prevents PAH-mediated cardiac toxicity and XRE- and ARE-associated gene induction in zebrafish (Danio rerio)," *Toxicol. Appl. Pharmacol.*, vol. 254, no. 3, pp. 280–287, Aug. 2011, doi: 10.1016/j.taap.2011.05.002.

[142] A. Ulin *et al.*, "Developmental Regulation of Nuclear Factor Erythroid-2 Related Factors (nrfs) by AHR1b in Zebrafish (Danio rerio)," *Toxicol. Sci.*, vol. 167, no. 2, pp. 536–545, Feb. 2019, doi: 10.1093/toxsci/kfy257.

[143] S. Shin *et al.*, "NRF2 Modulates Aryl Hydrocarbon Receptor Signaling: Influence on Adipogenesis," *Mol. Cell. Biol.*, vol. 27, no. 20, pp. 7188–7197, Oct. 2007, doi: 10.1128/MCB.00915-07.

[144] S. Kalthoff, U. Ehmer, N. Freiberg, M. P. Manns, and C. P. Strassburg, "Interaction between Oxidative Stress Sensor Nrf2 and Xenobiotic-activated Aryl Hydrocarbon Receptor in the Regulation of the Human Phase II Detoxifying UDP-glucuronosyltransferase 1A10," *J. Biol. Chem.*, vol. 285, no. 9, pp. 5993–6002, Feb. 2010, doi: 10.1074/jbc.M109.075770.

[145] M. S. Denison and S. C. Faber, "And now for something completely different: Diversity in ligand-dependent activation of Ah receptor responses," *Curr. Opin. Toxicol.*, vol. 2, pp. 124–131, Feb. 2017, doi: 10.1016/j.cotox.2017.01.006.

[146] A. A. Soshilov and M. S. Denison, "Ligand Promiscuity of Aryl Hydrocarbon Receptor Agonists and Antagonists Revealed by Site-Directed Mutagenesis," *Mol. Cell. Biol.*, vol. 34, no. 9, pp. 1707–1719, May 2014, doi: 10.1128/MCB.01183-13.

[147] N. Josyula, M. E. Andersen, N. E. Kaminski, E. Dere, T. R. Zacharewski, and S. Bhattacharya, "Gene co-regulation and co-expression in the aryl hydrocarbon receptor-mediated transcriptional regulatory network in the mouse liver," *Arch. Toxicol.*, vol. 94, no. 1, pp. 113–126, Jan. 2020, doi: 10.1007/s00204-019-02620-5.

[148] E. Vazquez-Rivera *et al.*, “The aryl hydrocarbon receptor as a model PAS sensor,” *Toxicol. Reports*, vol. 9, no. June 2021, pp. 1–11, 2022, doi: 10.1016/j.toxrep.2021.11.017.

[149] W. Zhang *et al.*, “The aryl hydrocarbon receptor: A predominant mediator for the toxicity of emerging dioxin-like compounds,” *J. Hazard. Mater.*, vol. 426, no. November 2021, p. 128084, Mar. 2022, doi: 10.1016/j.jhazmat.2021.128084.

[150] F. D. L. Leusch and S. A. Snyder, “Bioanalytical tools: half a century of application for potable reuse,” *Environ. Sci. Water Res. Technol.*, vol. 1, no. 5, pp. 606–621, 2015, doi: 10.1039/C5EW00115C.

[151] G. L. Warren *et al.*, “A Critical Assessment of Docking Programs and Scoring Functions,” *J. Med. Chem.*, vol. 49, no. 20, pp. 5912–5931, Oct. 2006, doi: 10.1021/jm050362n.

[152] S. Coecke *et al.*, “Metabolism: A Bottleneck in In Vitro Toxicological Test Development,” *Altern. to Lab. Anim.*, vol. 34, no. 1, pp. 49–84, Feb. 2006, doi: 10.1177/026119290603400113.

[153] S. Lungu-Mitea *et al.*, “On the Utilization and Characterization of External Biotransformation Systems in In Vitro Toxicology: A Critical Review of the Scientific Literature with Guidance Recommendations,” *ACS Environ. Au*, vol. 6, no. 1, pp. 21–45, Jan. 2026, doi: 10.1021/acsenvironau.5c00096.

[154] M. Eide, M. Rusten, R. Male, K. H. M. Jensen, and A. Goksøyr, “A characterization of the ZFL cell line and primary hepatocytes as in vitro liver cell models for the zebrafish (Danio rerio),” *Aquat. Toxicol.*, vol. 147, pp. 7–17, 2014, doi: 10.1016/j.aquatox.2013.11.023.

[155] V. Le Fol *et al.*, “Cell-Specific Biotransformation of Benzophenone-2 and Bisphenol-S in Zebrafish and Human in Vitro Models Used for Toxicity and Estrogenicity Screening,” *Environ. Sci. Technol.*, vol. 49, no. 6, pp. 3860–3868, Mar. 2015, doi: 10.1021/es505302c.

[156] G. Williamson *et al.*, “Induction of glutathione S-transferase activity in hepG2 cells by extracts from fruits and vegetables,” *Food Chem.*, vol. 60, no. 2, pp. 157–160, 1997, doi: 10.1016/S0308-8146(95)00255-3.

[157] A. Gronert, O. Zierau, D. Thieme, and A. M. Keiler, “Effect of HepG2 cell 3D cultivation on the metabolism of the anabolic androgenic steroid metandienone,” *Drug Test. Anal.*, vol. 15, no. 11–12, pp. 1319–1328, Nov. 2023, doi: 10.1002/dta.3455.

[158] E. L. Schaefer and J. K. Selkirk, “Metabolism of benzo(a)pyrene by variant mouse hepatoma cells.,” *Cancer Res.*, vol. 45, no. 8, pp. 3487–92, Aug. 1985, [Online]. Available: http://www.ncbi.nlm.nih.gov/pubmed/4016732.

[159] E. B. Froyen and F. M. Steinberg, “Soy isoflavones increase quinone reductase in hepa-1c1c7 cells via estrogen receptor beta and nuclear factor erythroid 2-related factor 2 binding to the antioxidant response element,” *J. Nutr. Biochem.*, vol. 22, no. 9, pp. 843–848, Sep. 2011, doi: 10.1016/j.jnutbio.2010.07.008.

[160] S. Wilkening, F. Stahl, and A. Bader, “COMPARISON OF PRIMARY HUMAN HEPATOCYTES AND HEPATOMA CELL LINE HEPG2 WITH REGARD TO THEIR BIOTRANSFORMATION PROPERTIES,” *Drug Metab. Dispos.*, vol. 31, no. 8, pp. 1035–1042, Aug. 2003, doi: 10.1124/dmd.31.8.1035.

[161] M. J. Gómez-Lechón, L. Tolosa, I. Conde, and M. T. Donato, “Competency of different cell models to predict human hepatotoxic drugs,” *Expert Opin. Drug Metab. Toxicol.*, vol. 10, no. 11, pp. 1553–1568, Nov. 2014, doi: 10.1517/17425255.2014.967680.

[162] J. A. Doering, R. Farmahin, S. Wiseman, S. W. Kennedy, J. P. Giesy, and M. Hecker, “Functionality of Aryl Hydrocarbon Receptors (AhR1 and AhR2) of White Sturgeon (Acipenser

transmontanus) and Implications for the Risk Assessment of Dioxin-like Compounds," *Environ. Sci. Technol.*, vol. 48, no. 14, pp. 8219–8226, Jul. 2014, doi: 10.1021/es502054h.

[163] R. L. Tanguay, C. C. Abnet, W. Heideman, and R. E. Peterson, "Cloning and characterization of the zebrafish (Danio rerio) aryl hydrocarbon receptor," *Biochim. Biophys. Acta - Gene Struct. Expr.*, vol. 1444, no. 1, pp. 35–48, Jan. 1999, doi: 10.1016/S0167-4781(98)00252-8.

[164] R. L. Tanguay, E. Andreasen, W. Heideman, and R. E. Peterson, "Identification and expression of alternatively spliced aryl hydrocarbon nuclear translocator 2 (ARNT2) cDNAs from zebrafish with distinct functions," *Biochim. Biophys. Acta - Gene Struct. Expr.*, vol. 1494, no. 1–2, pp. 117–128, Nov. 2000, doi: 10.1016/S0167-4781(00)00225-6.

[165] D. W. Cowing, J. C. A. Bardwell, E. A. Craig, C. Woolford, R. W. Hendrix, and C. A. Gross, "Consensus sequence for Escherichia coli heat shock gene promoters.," *Proc. Natl. Acad. Sci.*, vol. 82, no. 9, pp. 2679–2683, May 1985, doi: 10.1073/pnas.82.9.2679.

[166] M. Dreano *et al.*, "High-level, heat-regulated synthesis of proteins in eukaryotic cells," *Gene*, vol. 49, no. 1, pp. 1–8, Jan. 1986, doi: 10.1016/0378-1119(86)90380-X.

[167] A. A. Stepanenko and H. H. Heng, "Transient and stable vector transfection: Pitfalls, off-target effects, artifacts," *Mutat. Res. Mutat. Res.*, vol. 773, pp. 91–103, Jul. 2017, doi: 10.1016/j.mrrev.2017.05.002.

[168] M. J. Carvan 3rd, W. A. Solis, L. Gedamu, and D. W. Nebert, "Activation of transcription factors in zebrafish cell cultures by environmental pollutants," *Arch Biochem Biophys*, vol. 376, no. 2, pp. 320–327, 2000, doi: 10.1006/abbi.2000.1727.
